# Xylariaides A and B, novel cytochalasans with a unique 5/6/5/3 ring system from a soil fungus *Xylaria* sp. Y01

**DOI:** 10.1007/s13659-025-00507-w

**Published:** 2025-04-07

**Authors:** Yi-Yun Yuan, Yan Li, Wen-Yu Lu, Ai-Lin Liang, Jing Li, Wen-Xuan Wang

**Affiliations:** 1 Xiangya School of Pharmaceutical Sciences, Central South University, Changsha, 410008 Hunan People’s Republic of China; 2 Department of Pharmacy, National Clinical Research Center for Geriatric Disorder, Xiangya Hospital, Central South University, Changsha, 410008 Hunan People’s Republic of China; 3 Hunan Research Center for Drug Safety Evaluation, Hunan Key Laboratory of Pharmacodynamics and Safety Evaluation of New Drugs, Hunan Prima Drug Research Center Co., Ltd, Changsha, 410331 Hunan People’s Republic of China

**Keywords:** Cytochalasins, *Xylaria* sp., Coupling constant calculation, ECD calculation, GIAO ^13^C NMR calculation

## Abstract

Two new cytochalasans, xylariaides A (**1**) and B (**2**), were isolated and identified from a soil fungus *Xylaria* sp. Y01. Their structures were unambiguously determined by extensive spectroscopic methods including high resolution electrospray ionization mass spectrometry, ultraviolet radiation, infrared spectroscopy, and 1D/2D NMR, as well as in-depth quantum chemical calculations of gauge-including atomic orbital (GIAO) ^13^C NMR chemical shifts, electronic circular dichroism (ECD), and spin–spin coupling constants. The unprecedented core structure with a 5/6/5/3 fused tetracyclic ring system further enriches the scaffold types of cytochalasans.

**Graphical Abstract:**

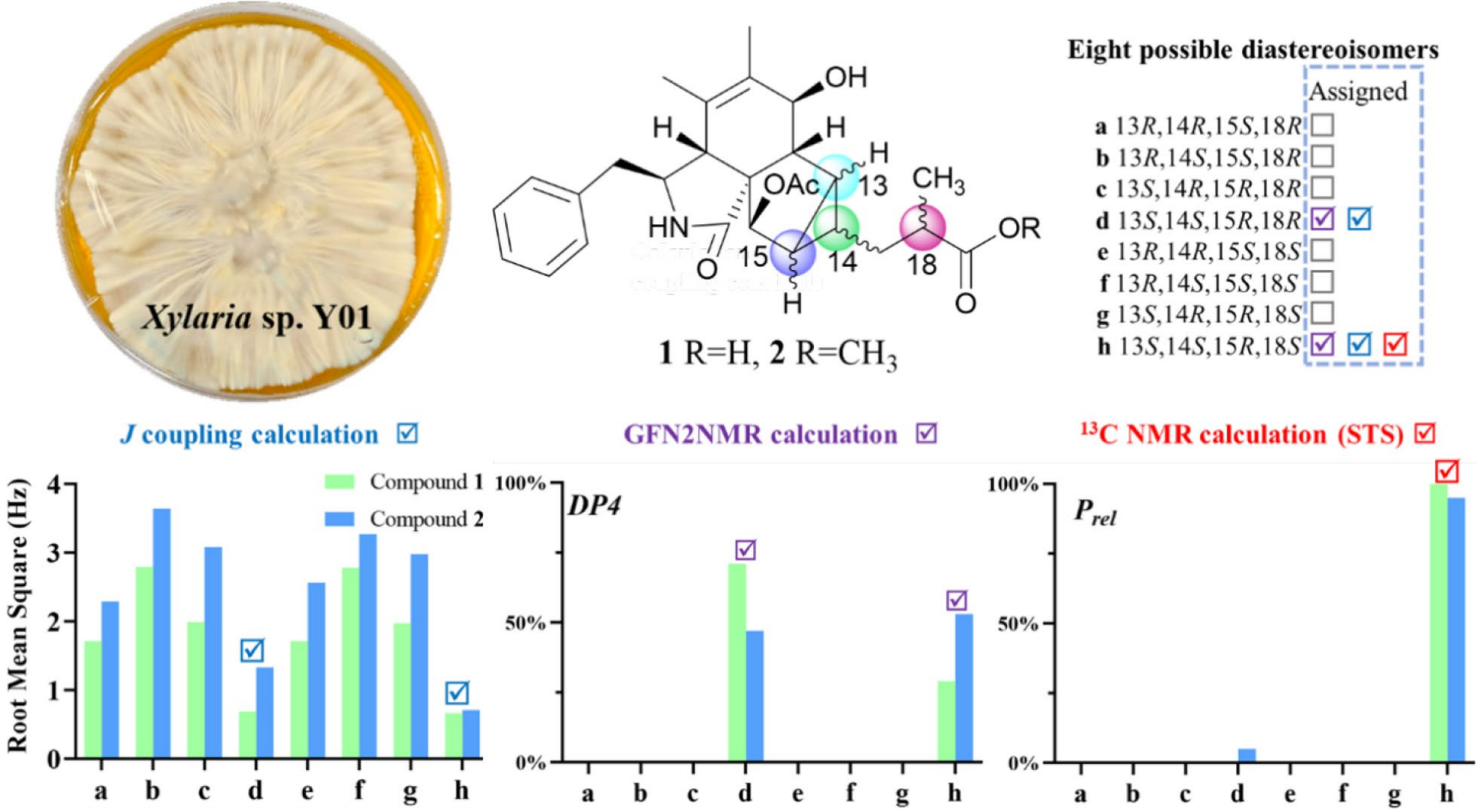

**Supplementary Information:**

The online version contains supplementary material available at 10.1007/s13659-025-00507-w.

## Introduction

The fungal genus *Xylaria*, a member of the Xylariaceae family, is widely distributed in both marine and terrestrial habitats [1]. Most *Xylaria* species are saprophytic fungi that decompose decaying wood, bark, dung, and other organic resources [1, 2]. The capacity of produceing complex, unique, and potent bioactive secondary metabolites is their well-known characteristic. As of 2020, 245 compounds (118 new ones) with a variety of bioactivities have been discovered from *Xylaria* species, including terpenoids, alkaloids, polyketides, cytochalasans, aromatic compounds, and sesquiterpenoids [1]. Many of them have shown tremendous potential to be developed as lead compounds [3].

**Fig. 1:**
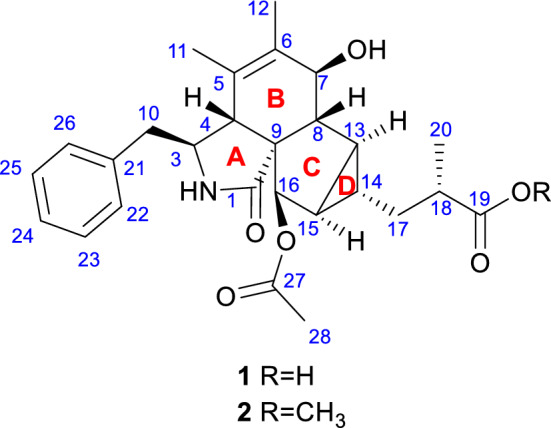
The structures of compounds **1** and **2**

Cytochalasans are a group of fungal secondary metabolites derived from hybrid pathways of polyketide synthase–non-ribosomal peptide synthetase (PKS–NRPS) with certain amino acids, typically with a tricyclic core scaffold formed by intramolecular Diels-Alder (IMDA) cyclization [4]. In recent years, there has been a surge of novel cytochalasans from *Xylaria* species, including newly discovered carbon skeleton types possessing 5/6/6/6 tetracyclic ring system (curtachalasin A) [5], bridged 6/6/6/6 ring system (curtachalasin C) [6], 6/7/5/6/6/6 polycyclic system (xylarichalasin A) [7], and open-chain merocytochalasans (perochalasins A−C) [8]. Moreover, these compounds also show a broad range of bioactivities, placing them at the forefront of research in natural medicinal chemistry [9]. Attracted by the active metabolites of soil-inhabiting fungi [10], we have conducted continuous research [11, 12] and identified a strain of fungus, *Xylaria* sp. Y01, from which a series of cytochalasans with cytotoxic effects were discovered [13]. In subsequent in-depth studies, we further discovered two new cytochalasans (**1** and **2**) with an unprecedented skeleton (Fig. 1). Herein, we report their isolation, structural elucidation, and preliminary bioactivity screening results.

## Results and discussion

### Planar configuration elucidation

Compound **1** was obtained as a yellow oil with the molecular formula C_27_H_33_NO_6_ determined by HRESIMS at *m/z* 468.2383 [M + H]^+^ (calcd. for C_27_H_34_NO_6_^+^, 468.2381), corresponding to 12 degrees of unsaturation. The infrared absorption spectrum (IR) peaks at 3443.9, 1683.3, and 1628.6 cm^−1^ correspond to the stretching vibrations of the hydroxyl, carboxylic carbonyl, and amide carbonyl groups, respectively. Its ultraviolet (UV) spectrum exhibited an absorption maximum at 257 nm, indicating the existence of conjugated systems.

The ^1^H NMR data (Table 1) revealed characteristic signals for a monosubstituted phenyl at *δ*_H_ 7.11 (2H, d, *J* = 7.6 Hz, H-22/H-26), 7.31 (2H, t, *J* = 7.6 Hz, H-23/H-25), and 7.24 (1H, t,* J* = 7.6 Hz, H-24), three singlet methyl groups at *δ*_H_ 1.40 (3H, s, H_3_-11), 1.64 (3H, s, H_3_-12), and 2.17 (3H, s, H_3_-28), a doublet methyl group at *δ*_H_ 1.16 (3H, d, *J* = 7.0 Hz, H_3_-20), and one amide NH proton at *δ*_H_ 6.09. The spectroscopic data also indicated two distinct groups of isolated and geminally coupled protons, one methylene at *δ*_H_ 2.61 (1H, dd, *J* = 13.3, 7.5 Hz) and 2.76 (1H, dd, *J* = 13.3, 7.8 Hz), the other at *δ*_H_ 1.65 (1H, m) and 1.47 (1H, ddd, *J* = 14.4, 8.7, 6.0 Hz). The ^13^C NMR, DEPT, and HSQC data of **1** displayed 27 carbon signals, including a carboxylic acid carbon at *δ*_C_ 179.6 (C-19), an amide carbonyl at *δ*_C_ 177.4 (C-1), an ester carbonyl at *δ*_C_ 170.0 (C-27), a double bond at *δ*_C_ 127.3 (C-5) and 134.8 (C-6), one nonprotonated carbon at *δ*_C_ 63.3 (C-9), two methylene carbons at *δ*_C_ 36.3 (C-17) and 42.7 (C-10), nine *sp*^3^ methine carbons at *δ*_C_ 28.9 (C-13), 29.8 (C-14), 32.0 (C-15), 38.6 (C-18), 47.5 (C-4), 52.4 (C-8), 58.1 (C-3), 70.8 (C-7), and 78.1 (C-16), two of which are oxygenated. These findings suggest that compound **1** contains four double bonds and one phenyl group, inferring the presence of four rings based on the 12 degrees of unsaturation derived from the molecular formula.

**Table 1:** The ^1^H NMR data (600 MHz) and ^13^C NMR data (150 MHz) of compounds **1** and **2** in CDCl_3_ (*δ* in ppm)

No	**1**		**2**	
	*δ*_C_, type	*δ*_H_	*δ*_C_, type	*δ*_H_
1	177.4, C	–	176.7, C	–
3	58.1, CH	3.39 (1H, ddd, 7.8, 7.5, 1.8)	57.8, CH	3.39 (1H, ddd, 7.8, 7.5, 1.8)
4	47.5, CH	2.67 (1H, br s)	47.7, CH	2.69 (1H, br s)
5	127.3, C	–	127.3, C	–
6	134.8, C	–	134.7, C	–
7	70.8, CH	4.26 (1H, d, 10.5)	70.7, CH	4.28 (1H, d, 10.5)
8	52.4, CH	1.60 (1H, dd, 10.5, 4.2)	52.5, CH	1.61 (1H, dd, 10.5, 4.3)
9	63.3, C	–	63.1, C	–
10	42.7, CH_2_	2.61 (1H, dd, 13.3, 7.5)	42.8, CH_2_	2.65 (1H, dd, 13.4, 7.5)
		2.76 (1H, dd, 13.3, 7.8)		2.72 (1H, dd, 13.4, 7.8)
11	17.9, CH_3_	1.40 (3H, s)	17.9, CH_3_	1.47 (3H, s)
12	14.4, CH_3_	1.64 (3H, s)	14.3, CH_3_	1.67 (3H, s)
13	28.9, CH	1.80 (1H, ddd, 7.7, 4.2, 3.8)	28.8, CH	1.72 (1H, ddd, 7.7, 4.3, 3.4)
14	29.8, CH	1.13 (1H, m)	30.3, CH	1.09 (1H, dddd, 7.2, 7.2, 3.4, 3.4)
15	32.0, CH	1.87 (1H, ddd, 7.7, 5.6, 3.3)	32.2, CH	1.87 (1H, ddd, 7.7, 5.6, 3.4)
16	78.1, CH	5.39 (1H, d, 5.6)	77.8, CH	5.40 (1H, d, 5.6)
17	36.3, CH_2_	1.65 (1H, m)	36.1, CH_2_	1.58 (1H, overlapped)
		1.47 (1H, ddd, 14.4, 8.7, 6.0)		1.48 (1H, ddd, 14.4, 7.2, 6.8)
18	38.6, CH	2.60 (1H, m)	39.0, CH	2.57 (1H, m)
19	179.6, C	–	177.0, C	-
20	15.9, CH_3_	1.16 (3H, d, 7.0)	16.2, CH_3_	1.16 (3H, d, 7.0)
21	137.3, C	–	137.3, C	-
22/26	129.0, CH_2_	7.11 (2H, d, 7.6)	129.0, CH_2_	7.12 (2H, d, 7.3)
23/25	128.8, CH_2_	7.31 (2H, t, 7.6)	128.8, CH_2_	7.31 (2H, t, 7.3)
24	127.0, CH	7.24 (1H, t, 7.6)	127.0, CH	7.25 (1H, t, 7.3)
27	170.0, C	–	169.7, C	–
28	21.0, CH_3_	2.17 (3H, s)	21.0, CH_3_	2.17 (3H, s)
19-OCH_3_	–	–	51.7, CH_3_	3.68 (3H, s)
NH		6.09 (1H, br s)		5.42 (1H, br s)

**Fig. 2:**
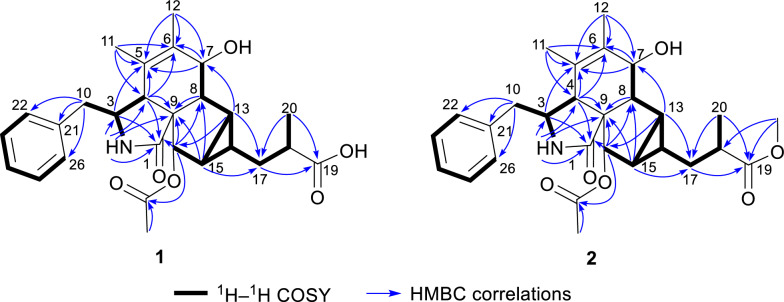
^1^H–.^1^H COSY and key HMBC correlations of compounds **1** and **2**

The ^1^H–^1^H COSY spectrum of **1** indicated three proton spin–spin systems, namely H_2_-10/H-3/H-4/2-NH, H-7/H-8/H-13/H-14(/H-15/H-16)/H_2_-17/H-18/H_3_-20, and H-22/H-23/H-24/H-25/H-26 (Fig. 2). The HMBC correlations (Fig. 2) between NH and C-1/C-3/C-4/C-9, H-4 and C-1/C-6/C-10, H-7 and C-5, H_3_-11 and C-4/C-5/C-6, H_3_-12 and C-5/C-6/C-7, as well as H-8 and C-4/C-6 established the structural connectivity between the five-membered lactam ring (A ring) and the six-membered ring (B ring). A proposed ring C fused with a tricyclic ring D was deduced by the HMBC correlations between H-13 and C-7/C-16/C-17, H-15 and C-9/C-8/C-17, H-16 and C-1/C-8/C-14, as well as H-14 and C-8/C-16, along with the ^1^H–^1^H COSY correlations of H-13/H-15/H-14. Additionally, the spin–spin coupling of H-14/H_2_-17/H-18/H_3_-20, and the HMBC correlations between H_3_-20 and C-17/C-18/C-19, H_2_-17 and C-13/C-15/C-19 suggested the presence of a 2-methylpropanoic acid moiety attached to C-14 of the tricyclic ring (D ring). Further HMBC correlation between H-16 and C-27 verified the attachment of the acetyl group at C-16. Thus, the planar structure of compound **1** was defined as a novel cytochalasan skeleton with a unique bicyclo[3.1.0]hexane moiety.

**Fig. 3:**
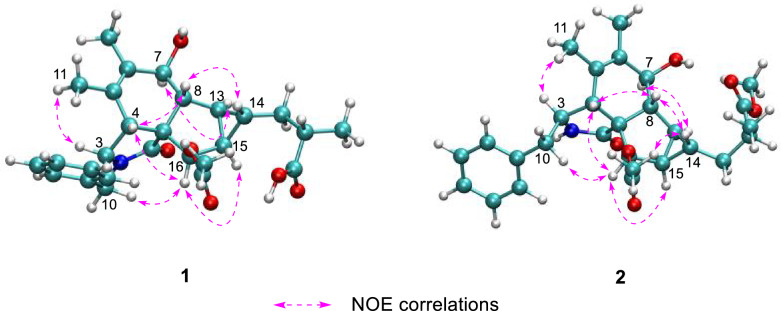
Key NOE correlations of compounds **1** and **2**, depicted with their optimal conformations (coordinates are provided in Tables S14 and S22)

Nuclear Overhauser effects (NOEs) between H-4 and H-16, as well as between H_2_-10 and H-16, suggest that these protons are on the same side of the five-membered lactam ring. The NOESY spectrum showed correlations of H-4/H-8/H-14 and of H-7/H-13, suggesting that H-4, H-8, and H-14 are cofacial, while H-7 and H-13 are cofacial (Fig. 3). The equatorial position of H-7 was inferred from the coupling constant between H-7 and H-8 (*J* = 10.5 Hz) (Table 1). Previous studies have indicated that the essential elements of most cytochalasan skeletons share the same stereochemistry, with configurations at C-3, C-4, C-7, C-8, C-9, and C-16 assigned as 3*S*, 4*R*, 7*S*, 8*R*, 9*R*, and 16*R*, respectively [5, 13], which is consistent with the aforementioned NOE analyses. However, the relative configuration of ring D could not be fully elucidated by NOESY experiments. Fortunately, coupling constants between vicinal protons in ring D provide valuable information for determining their relative orientations. H-13 exhibited coupling constants of 7.7, 4.2, and 3.8 Hz with H-15, H-8, and H-14, respectively. The small coupling constant between H-13 and H-14 (*J* = 3.8 Hz) and the large coupling constant between H-13 and H-15 (*J* = 7.7 Hz) suggest that H-13 and H-15 are coplanar and both diaxial to H-14.

**Fig. 4:**
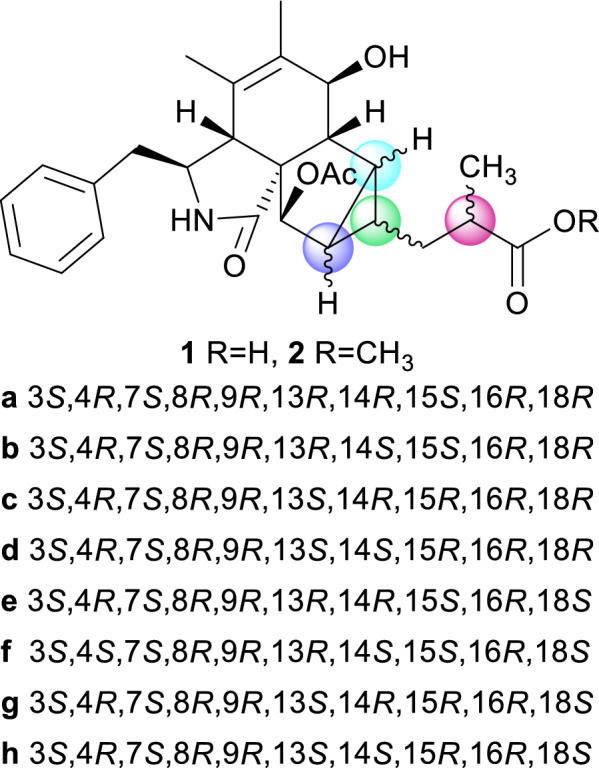
Eight possible epimers of compounds** 1** and **2**

To confirm the relative configuration of ring D, the *J* coupling constants for different possible conformations of ring D were calculated [14]. The fusion of rings C and D is restricted to a *cis* configuration due to strain that inhibits the *trans* configuration from achieving a stable geometric structure. Therefore, the relative configuration of **1** is one of the eight possible relative diastereoisomers (**1a**–**1h**), as shown in Fig. 4. The *J* coupling constants of protons on rigid rings for these eight possible epimers were correlated with the experimental coupling constants to calculate the mean absolute error (MAE) and root mean square error (RMS). The results indicate that the relative configurations of **1d** and **1h** align more closely with the experimental values, as they exhibit lower MAE and RMS compared to the other epimers (Table 2). These findings are consistent with the previous analysis.

**Table 2:** Experimental *J* coupling constants (CDCl_3_) for compounds **1** and **2**, and theoretical values for eight possible epimers (detailed data are provided in Table S5 and S6; the values in bold font are the optimal values in their respective columns)

Structures	Compound **1**		Compound **2**	
	MAE	RMS	MAE	RMS
**a**	1.05	1.82	0.98	1.66
**b**	1.92	2.77	1.95	2.81
**c**	1.32	2.24	1.24	2.01
**d**	**0.47**	0.68	0.43	0.55
**e**	1.01	1.73	0.97	1.65
**f**	1.98	2.87	1.91	2.81
**g**	1.26	2.05	1.22	2.00
**h**	0.49	**0.66**	**0.38**	**0.51**

To discern the chirality at key positions from an alternative perspective, ^13^C NMR calculations were utilized by analyzing chemical shift differences. The GFN2NMR method, a semiempirical approach integrated into a deep graph convolutional network, facilitates rapid and precise ^13^C NMR chemical shift calculations, noted for its high accuracy and low computational cost [15]. It is capable of distinguishing diastereomers based on the rules learned from large datasets. The most likely structures of **1** were swiftly identified from eight possible isomers using the GFN2NMR method. Since their *P*_mean_ values fall within the confidence interval and *P*_rel_ exceeds 5% (**1d** at 71%, **1h** at 29%), both **1d** and **1h** are assigned as plausible configurations (Table 3).

**Table 3:** Experimental ^13^C NMR chemical shifts (CDCl_3_) of compounds **1** and **2**, and the calculated chemical shifts for the eight possible epimers predicted by GFN2NMR (detailed data are provided in Table S1 and S2; the values in bold font are the optimal values in their respective columns)

Structures	Compound **1**				Compound **2**			
	MAE	RMS	*P*_*mean*_	DP4	MAE	RMS	*P*_*mean*_	DP4
**a**	2.12	2.97	26%	0%	2.12	3.32	22%	0%
**b**	2.62	3.63	16%	0%	2.14	3.33	22%	0%
**c**	2.18	2.99	25%	0%	1.90	2.58	33%	0%
**d**	**1.43**	**1.72**	51%	**71%**	**1.56**	1.95	**46%**	47%
**e**	2.30	3.24	22%	0%	2.21	3.26	22%	0%
**f**	2.41	3.30	21%	0%	2.17	3.22	23%	0%
**g**	2.17	3.23	23%	0%	1.74	2.35	38%	0%
**h**	1.45	1.82	**49%**	29%	1.58	**1.92**	**46%**	**53%**

**Fig. 5:**
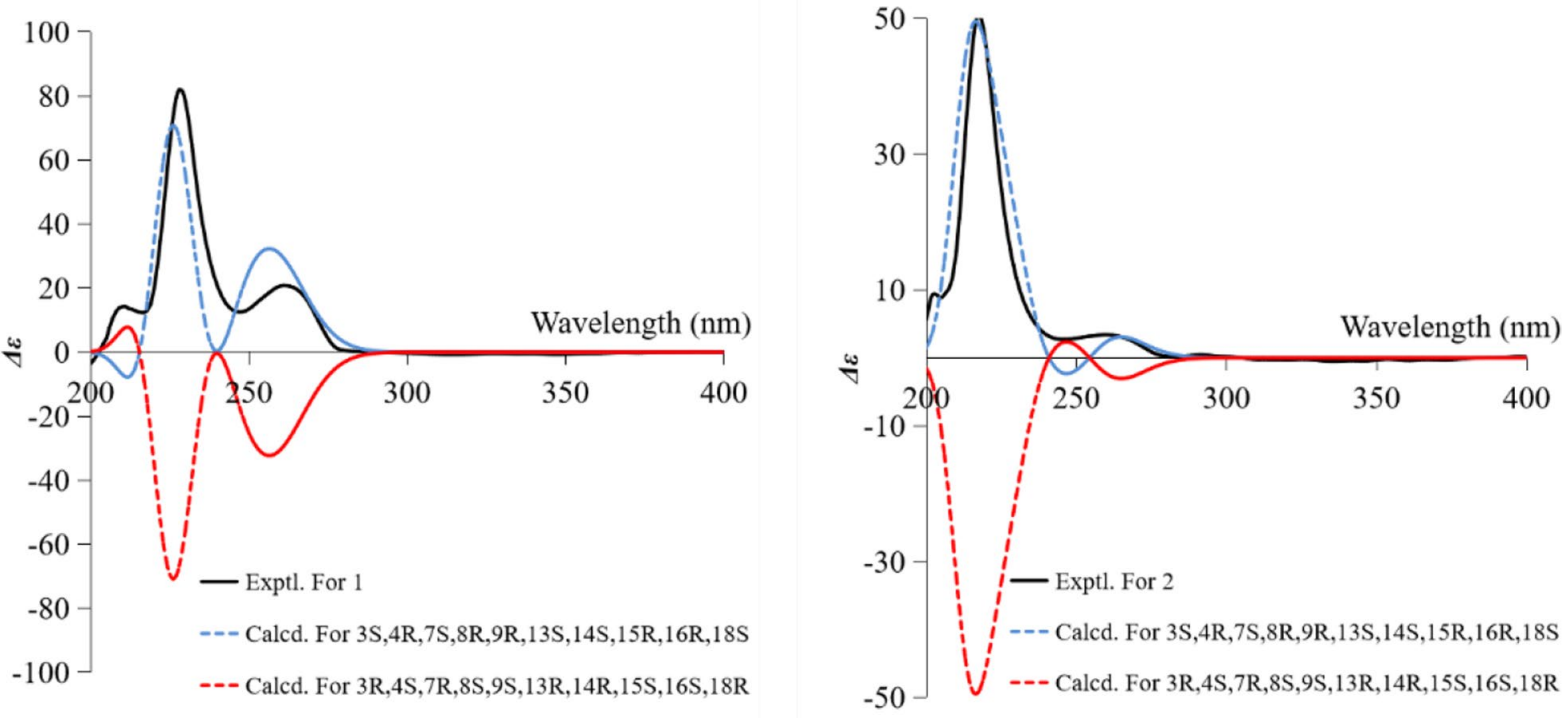
The experimental and calculated ECD curves of **1** and **2** and their enantiomers

The GIAO ^13^C NMR calculations with the sorted training sets (STS) protocol showed significantly enhanced accuracy and reliability for structural determination [16] (e. g., pestalopyrones A–D [12]), which provided a tool based on quantum chemistry. The calculated chemical shifts by STS for the eight possible epimers** 1a**–**1h** were correlated with the experimental ^13^C NMR chemical shifts to determine MAE, RMS, and the statistical parameters *P*_mean_ and *P*_rel_ (Table 4). The ^13^C NMR calculations for compound **1h** closely matched the experimental data, with both the MAE and RMS values significantly lower than those for **1b**. Furthermore, the *P*_rel_ probability calculations assigned a 100% likelihood to **1h**. This result not only established the chirality at C-18 but also validated the conformation of the D ring, consistent with the possible structures inferred from *J* coupling constants calculations and GFN2NMR analysis. An ECD calculation for **1h** was conducted to determine the absolute configuration of **1**, indicating a configuration of 3*S*,4*R*,7*S*,8*R*,9*R*,13*S*,14*S*,15*R*,16*R*,18*S*, which matched well with the experimental ECD data as shown in Fig. 5. These findings confirm that **1h** is the correct structure of xylariaide A.

**Table 4:** ^13^C NMR chemical shifts calculation (CDCl_3_) of structures **a**–**h** fitting to the experimental data of compounds **1** and **2** using GIAO ^13^C NMR calculations with the STS protocol (detailed data are provided in Table S3 and S4; the values in bold font are the optimal values in their respective columns)

Structures	Compound 1				Compound 2			
	MAE	RMS	*P*_*mean*_	*P*_*rel*_	MAE	RMS	*P*_*mean*_	*P*_*rel*_
**a**	2.69	3.70	0%	0%	2.55	3.65	0%	0%
**b**	2.59	3.29	1%	0%	2.51	3.22	1%	0%
**c**	2.10	2.85	2%	0%	2.08	2.69	3%	0%
**d**	1.46	1.84	20%	0%	1.03	1.24	42%	5%
**e**	2.67	3.51	0%	0%	2.55	3.46	0%	0%
**f**	2.65	3.31	1%	0%	2.58	3.36	1%	0%
**g**	2.13	2.95	2%	0%	2.05	2.60	4%	0%
**h**	**1.19**	**1.55**	**33%**	**100%**	**0.95**	**1.17**	**47%**	**95%**

Compound** 2** was obtained as an amorphous powder, with a molecular formula of C_28_H_35_NO_6_, as determined by HRESIMS at *m/z* 482.2538 [M + H]^+^ (calcd. for C_28_H_36_NO_6_^+^, 482.2538). The one-dimensional spectrum data of compound **2** exhibited an additional methoxyl group at *δ*_H_ 3.68 (3H, s) and *δ*_C_ 51.7, with all other signals closely resembling those of **1** except for the active proton signal of 2-NH (Table 1). The HMBC correlation between -OCH_3_ and C-19 confirmed the attachment of the methoxyl group at C-19. NOE signals indicate that H-4, H_2_-10, and H-16 are on the same side of the five-membered lactam ring, while H_3_-11 and H-3 are on the opposite side. The optimal conformations of compounds **1** and **2**, as illustrated in Fig. 3, differ significantly in the configuration of the 2-methylpropionic acid moiety, leading to the absence of a distinct NOE signal between H-14 and H-18 for **2**. The remaining NOE signals for **2** are consistent with those of **1**. Similarly, the *J* coupling constants and ^13^C NMR calculations of **2** were also performed, aligning with the results for **1** and confirming the relative configuration of **2**. The experimental ECD spectrum of **2** displayed two positive Cotton effects at 215 nm (Δ*ε* + 52.6) and 258 nm (Δ*ε* + 3.5), similar to the spectrum of **1**. Absolute confirmation was determined by ECD calculation, with the experimental values closely matching those for the configuration 3*S*,4*R*,7*S*,8*R*,9*R*,13*S*,14*S*,15*R*,16*R*,18*S*, namely xylariaide B.

**Fig. 6:**
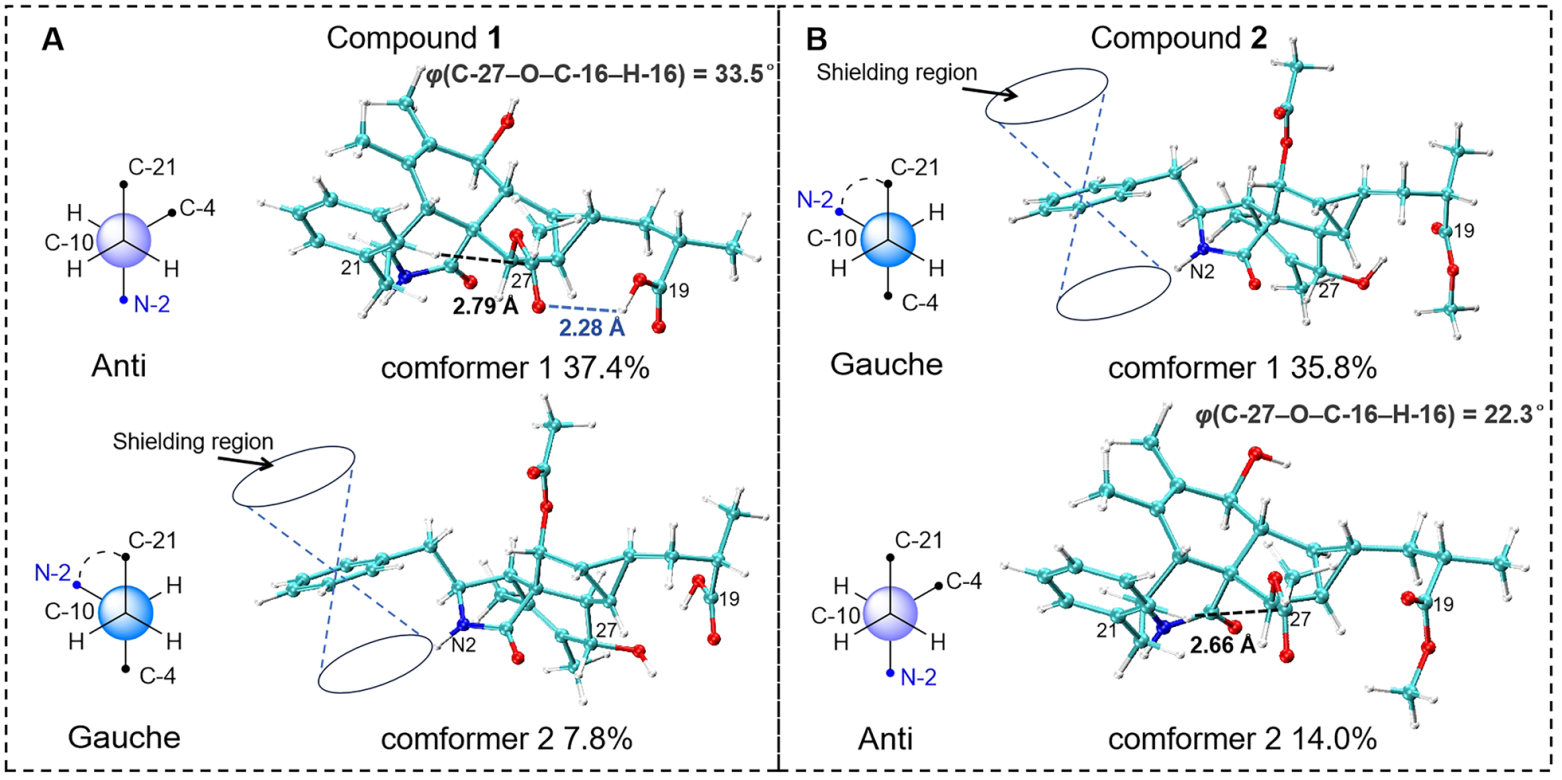
Analysis of the magnetic anisotropic effect of the benzene ring on the 2-NH proton in compounds **1** and **2**. Conformer 1 of compounds **1** and **2** represents the global minimum conformation, respectively

Upon comparing the structures of compounds **1** and **2**, it is evident that their only difference lies in the methoxy substitution on the 19-COOH group at the side chain. In the 1D NMR spectra, most of their signals are remarkably similar. However, the chemical shift of the 2-NH proton, which is distant from the side chain, shows a significant difference of 0.6 ppm. To investigate the cause of this discrepancy and further validate the structural assignments, we analyzed the low-energy conformations of compounds **1** and **2**, as shown in Fig. 6. It revealed that the most dominant conformation of compound **1** features the 10-phenyl ring and the N-2 group adopting an anti-conformation. In contrast, for compound **2**, the gauche conformation is energetically more favorable and predominant. In this conformation, the 2-NH proton lies within the shielding region of the 10-phenyl ring, which explains why the chemical shift of 2-NH in compound **2** appears at a higher field in the ^1^H NMR spectrum. Further examination of their geometric structures revealed that in the lowest-energy conformation of compound **1**, a weak hydrogen bond (bond length: 2.28 Å) forms between the 19-COOH and 27-CO groups. This interaction pulls the 16-acetyl group away from the 10-phenyl ring (the distance between 22-H and C-27 is 2.79 Å, and the dihedral angle between C-27 and H-16 is 33.5º), allowing the phenyl ring to adopt the energetically favorable anti-conformation. In contrast, in the anti-conformation of compound **2**, the 16-acetyl group is closer to the 10-phenyl ring (the distance between 22-H and C-27 is 2.66 Å, and the dihedral angle between C-27 and H-16 is 22.3º). The resulting steric hindrance likely increases the energy of the anti-conformation, making the gauche conformation the lowest-energy state. This analysis not only provides a clear explanation for why a simple methoxy substitution on the 19-COOH group can lead to a significant change in the chemical shift of the distant 2-NH proton, but also further corroborates the validity of the structural assignments.

### Proposed biosynthetic pathway

**Scheme 1:**
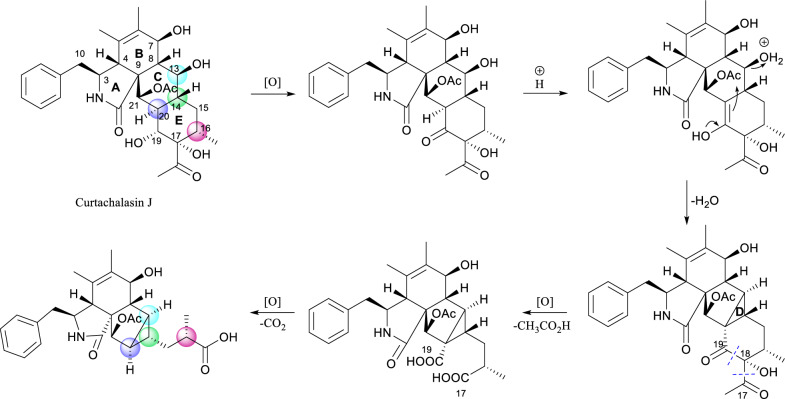
Proposed Biosynthetic Pathway of compounds **1** and **2**

Compounds **1** and **2** contain the same rings A and B as curtachalasin J [17], a co-isolated known cytochalasan identified from this strain (NMR data provided in Figs. S22 and S23), and also feature a benzyl group at C-3. This observation suggests that the 5/6/5/3 ring system common to compounds **1** and **2** may be biogenetically derived from the known compound curtachalasin J. Intramolecular cyclization between C-13 and C-20 in curtachalasin J could result in the bicyclo[3.1.0]hexane moiety. Besides, inspired by the C–C bond cleavage reactions on α-hydroxyketones [18], we propose that the carbon bonds between C-17 and C-18 and between C-17 and C-19 within ring E undergo oxidative cleavage, and both C-17 and C-19 are transformed into carboxylic acids. The following decarboxylation at C-19 leads to the formation of compound **1**. The stereochemistry of C-8, C-13, C-14, C-16, C-20, and C-21 in curtachalasin J corresponds to that of C-8, C-13, C-14, C-18, C-15, and C-16 in compound **1**, respectively, further supporting the proposed biogenetic pathway (Scheme 1).

### Preliminary bioactivity screening results

The cytochalasans, a well-known family of fungal metabolites, are recognized for their ability to inhibit the polymerization of filamentous (F)-actin [19]. These compounds have shown significant anticancer properties in numerous in *vitro* and in *vivo* studies [20]. To evaluate the potential anticancer properties of compounds **1** and **2**, a cytotoxicity assessment was conducted on three cancer cell lines (HCT116, 4T1, and MHCC97H) and one normal cell line (GES-1). Compounds **1** and **2** did not exhibit significant cytotoxic effects against any of the cell lines tested.

To assess the antimicrobial activity of compounds **1** and **2**, a screen for antibacterial activity against *Bacillus subtilis*, *Escherichia coli*, *Staphylococcus aureus*, *Acinetobacter baumannii*, and *Pseudomonas aeruginosa*, as well as for antifungal activity against *Saccharomyces cerevisiae* and *Canidia albicans*, was conducted*.* Both compounds showed MIC values above 50 *μ*M, indicating no significant antimicrobial activity against the bacterial strains tested.

The anti-inflammatory activity of compounds **1** and **2** was evaluated by assessing their inhibitory effects on nitric oxide (NO) production in lipopolysaccharide (LPS)-stimulated RAW264.7 macrophages. Compounds **1** and **2** demonstrated no significant inhibitory activity on NO production at a concentration of 30 *μ*M.

## Experimental

### General

UV spectra were measured by Nano-300 Microvolume Spectrophotometer (Hangzhou Allsheng Instruments) with a 1.0 mm pathlength. CD spectra were recorded using a Chirascan™-plus Circular Dichroism spectrometer (Applied Photophysics Ltd.). Optical rotations were measured using a Autopol IV automatic polarimeter (Rudolph Research Analytical). IR spectra were recorded on a Shimadzu Fourier Transform Infrared Spectrometer using KBr pellets. HRESIMS spectra were obtained with an Agilent 6500 series Q-TOF mass spectrometer (Agilent Technologies, Singapore). NMR spectra were obtained at 600 MHz for ^1^H and 150 MHz for ^13^C on Bruker spectrometers (Bruker BioSpin, Rheinstetten, Germany). Column chromatography (CC) was performed with macroporous adsorbent resin D101 (Tianjin Haoju Resin Technology Co., LTD, Tianjin, China), and silica gel (200 − 300 mesh and 300 − 400 mesh, Qingdao Marine Chemical, Qingdao, China). Thin Layer Chromatography (TLC) separations were executed on precoated silica gel GF254 plates (Qingdao Marine Chemical), with visualization under UV light or by spraying with 5–15% H_2_SO_4_ in EtOH followed by heating. HPLC separation was performed using a Ruihe Tech prep-HPLC system (xgt6515-10 s) with SinoChrom ODS-AP (Elite, 5 *μ*m, 10 × 250 mm) and a DAD detector (Ruihe tech, LC2050).

### Fungi culture, extraction and isolation

A soil sample from Yunnan, China, yielded a strain of *Xylaria* designated as Y01. The methods for sample processing and fungal species identification followed those outlined in our previous publication [13]. *Xylaria* sp. Y01 hyphae were cultivated in SDA liquid medium at a constant temperature of 25 °C for 10 days. These cultures were then transferred to rice medium in 500 mL Erlenmeyer flasks and incubated at 25 °C for 40 days. A total of 10 kg of rice was used for the cultures. The fermented rice medium was extracted with 30 L of ethyl acetate, repeated ten times at room temperature, yielding a crude extract of 200 g. This extract was processed through 300 g of macroporous resin D101, followed by sequential elution with water, 80% aqueous methanol, and pure methanol. The fraction eluted with the 80% methanolic solution was collected and concentrated to yield a residue of 100 g. This methanol eluate between 80 and 90% was further subjected to column chromatography on silica gel, using a gradient of petroleum ether and ethyl acetate in ratios ranging from 5:1 to 0:1, to separate it into five distinct subfractions (Fr. A to E). The remaining Fr. B was purified by a semipreparative HPLC system successively to yield **1** (5.11 mg, 25% methanol, 3.0 mL/min, *t*_R_ = 25.0 min, SinoChrom ODS-AP: 10.0 mm × 250 mm, 5 *μ*m), and **2** (0.45 mg, 60% methanol, 2.5 mL/min, *t*_R_ = 14.0 min, SinoChrom ODS-AP: 10.0 mm × 250 mm, 5 *μ*m).

### Compound characterization

*Xylariaide A* (**1**): Yellow oily. $[\alpha]^{25}_{\text{D}}+52.00$ (*c* 0.511, MeOH). IR (KBr): *υ*_max_ 3443, 1683, 1628, 1379.9, 1125, 854 cm^−1^. UV (CH_3_CN) *λ*_max_ (log *ε*) 257 (2.5) nm. ^1^H NMR (600 MHz in CDCl_3_) and ^13^C NMR (150 MHz in CDCl_3_) data, see Table 1. HRESIMS *m/z* 468.2383 [M + H]^+^ (calcd. for C_27_H_34_NO_6_^+^, 468.2381, Δ + 0.6 ppm), *m/z* 450.2279 [M + H − H_2_O]^+^ (calcd. for C_27_H_32_NO_5_^+^, 450.2275, Δ + 0.9 ppm).

*Xylariaide B* (**2**): White powder. $[\alpha]^{25}_{\text{D}}+142.00$ (*c* 0.066, MeOH). UV (CH_3_CN) *λ*_max_ (log *ε*) 257 (2.4) nm. ^1^H NMR (600 MHz in CDCl_3_) and ^13^C NMR (150 MHz in CDCl_3_) data, see Table 1. HRESIMS *m/z* 482.2538 [M + H]^+^ (calcd. for C_28_H_36_NO_6_^+^, 482.2538, Δ 0.0 ppm), *m/z* 464.2431 [M + H − H_2_O]^+^ (calcd. for C_28_H_34_NO_5_^+^, 464.2432, Δ − 0.2 ppm). The IR spectrum was not measured because of its low sample amount.

### Assay of antimicrobial activity

The antimicrobial activity of the two cytochalasans (**1** and **2**) against *B. subtilis* ATCC 6051, *E. coli* DH5α, *S. aureus* ATCC25923, *A. baumannii* ATCC19606, and *P. aeruginosa* ATCC27853 as well as *S. cerevisiae* ATCC9763 and *Canidia albicans* ATCC10231 was evaluated. Samples were prepared by dissolving them in an 80% ethanol aqueous solution to achieve an initial concentration of 1 mM. The samples were then diluted to a final concentration of 7.5 × 10^5^ CFU/mL, and bacterial solutions were added to each well of a 96-well culture plate. The plate was incubated at 37 °C for 24 h, after which the wells were visually inspected to evaluate growth inhibition or clearing of the bacterial cultures. The experiment included media blank, bacterial control, streptomycin-positive bacterial control, and fluconazole-positive fungal controls for comparative analysis.

### Assay of cytotoxic activity

The cell culture and cytotoxic activity assays were conducted as previously described [13].

### Assay of anti-inflammatory activity

RAW264.7 cells were cultured in DMEM medium supplemented with 10% fetal bovine serum, 2 mM L-glutamine, 1 mM sodium pyruvate, and 2 mg/mL gentamicin, and maintained in a humidified atmosphere at 37 °C with 5% CO_2_. For the assay, cells were seeded into 96-well plates at a density of 5 × 10^5^ cells per well and incubated with the two isolates (**1** and **2**) at a concentration of 30.0 *μ*M for an additional 24 h under the same conditions. After 24 h of incubation, 10 *μ*L of CCK-8 reagent was added to the cell medium and incubated at 37 °C for 4 h. The absorbance was measured at 450 nm using a microplate reader. Absorbance values from untreated cells were taken as 100% viability. No significant cytotoxic effect was observed, permitting the use of concentrations below 30 *μ*M of **1** and **2** in subsequent experiments.

Nitric oxide (NO) production was determined by measuring the nitrite content in the cell culture supernatant using Griess reagent. RAW264.7 cells were treated with LPS (1 *µ*g/mL) in the presence or absence of **1** and **2** (30 *µ*M) for 24 h. 100 *µ*L of the cell culture supernatant was mixed with 100 *µ*L of Griess reagent and incubated for 10 min at room temperature. The absorbance at 540 nm was measured.

### Quantum chemical calculation

The software Crest [21] was employed to explore the conformational space of potential structures within the GFN0 [22, 23] theoretical framework. Subsequently, structures were optimized at the GFN2-xTB [22] level, with a threshold of 4 kcal/mol to exclude high-energy conformers. Optimization and frequency calculation of each conformer were performed on B3LYP-D3(BJ)/TZVP (IEFPCM) level of theory. DFT GIAO ^13^C NMR calculations were performed on the *ω*B97x-D/6-31G* (IEFPCM, chloroform) level, aligning with the reported STS protocol [16]. ECD calculations for **1** and **2** were executed on the B3LYP/TZVP (IEFPCM, methanol) level. The calculated shielding tensors and ECD curves of conformers were Boltzmann-averaged based on Gibbs free energy. SpecDis V1.71 [24] was used to Boltzmann-average and simulate the ECD curves of all conformers. For both 1 and 2, sigma/gamma values were set to 0.3 eV, and the calculated curves were red-shifted by 25 nm. The *J* coupling constants calculations for protons in rigid rings were conducted using B3LYP/PCSSEG-2 (CPCM, chloroform) with the ORCA program [25]. GIAO ^13^C NMR and ECD calculations were executed using the Gaussian 16 software package [26]. Supplementary material includes DFT-optimized geometry data, relative energies, and conformational populations of all calculated structures. The 3D structures of **1** and **2** were generated using VMD (1.9.4) [27].

## Conclusions

This study reports the discovery of xylariaides A and B, which feature a novel cytochalasan skeleton. Although efforts to obtain single crystals for X-ray diffraction analysis were unsuccessful, the structure was reliably determined using a combination of spectroscopic methods, ^13^C NMR calculations with the STS protocol, and ECD calculations. These three distinct validation techniques each effectively confirmed the conformation of the tricyclic ring system. Despite the lack of positive results from recent bioactivity assays, these findings enrich the scaffolds of cytochalasans and lay a foundation for future research.

## Supplementary Information

Additional file 1.

## Data Availability

All data generated or analyzed during this study are included in this published article and its supplementary information files.
