# Supplementary material for: Xylariaides A and B, novel cytochalasans with a unique 5/6/5/3 ring system from a soil fungus Xylaria sp. Y01
Source: Nat Prod Bioprospect. 2025 Apr 7;15(1):23. doi: 10.1007/s13659-025-00507-w (PMC11977066; doi:10.1007/s13659-025-00507-w)
Supplement: Supplementary file 1 — Additional file 1. [file 13659_2025_507_MOESM1_ESM.docx]

**Supplementary Material**

Xylariaides A and B, novel cytochalasans with a unique 5/6/5/3 ring system a soil fungus *Xylaria* sp. Y01

Yi-Yun Yuan^1^, Yan Li^1^, Wen-Yu Lu^1^, Ai-Lin Liang^1^, Jing Li^1,2,3^* and Wen-Xuan Wang^1,3^*

^1^Xiangya School of Pharmaceutical Sciences, Central South University, Changsha, Hunan 410008, PR China.

^2^Department of Pharmacy, National Clinical Research Center for Geriatric Disorder, Xiangya Hospital, Central South University, Changsha, Hunan 410008, PR China.

^3^Hunan Prima Drug Research Center Co., Ltd, Hunan Research Center for Drug Safety Evaluation, Hunan Key Laboratory of Pharmacodynamics and Safety Evaluation of New Drugs, Changsha, Hunan 410331, PR China.

Corresponding authors: [lijingliyun@csu.edu.cn](mailto:lijingliyun@csu.edu.cn) (Jing Li); [wangwenxuan@csu.edu.cn](mailto:wangwenxuan@csu.edu.cn) (Wen-Xuan Wang)

**Table of contents**

[Fig. S1. ^1^H NMR (CDCl_3_, 600 MHz) of **1**. 4](#_Toc190804085)

[Fig. S2. ^13^C NMR (CDCl_3_, 150 MHz) of **1**. 4](#_Toc190804086)

[Fig. S3. DEPT 135 spectra of **1**. 5](#_Toc190804087)

[Fig. S4. HSQC of **1**. 5](#_Toc190804088)

[Fig. S5. ^1^H−^1^H COSY of **1**. 6](#_Toc190804089)

[Fig. S6. HMBC of **1**. 6](#_Toc190804090)

[Fig. S7. NOESY of **1**. 7](#_Toc190804091)

[Fig. S8. HRESIMS and MS/MS spectrum (collision-induced dissociation energy: 20 eV) of **1**. 7](#_Toc190804092)

[Fig. S9. CD spectrum(methanol) of **1**. 8](#_Toc190804093)

[Fig. S10. UV spectrum (acetonitrile) of **1**. 8](#_Toc190804094)

[Fig. S11. IR spectrum of **1**. 8](#_Toc190804095)

[Fig. S12. ^1^H NMR (CDCl_3_, 600 MHz) of **2**. 9](#_Toc190804096)

[Fig. S13. ^13^C NMR (CDCl_3_, 150 MHz) of **2**. 9](#_Toc190804097)

[Fig. S14. DEPT 135 spectra of **2**. 10](#_Toc190804098)

[Fig. S15. HSQC of **2**. 10](#_Toc190804099)

[Fig. S16. ^1^H−^1^H COSY of **2**. 11](#_Toc190804100)

[Fig. S17. HMBC of **2**. 11](#_Toc190804101)

[Fig. S18. NOESY of **2**. 12](#_Toc190804102)

[Fig. S19. HRESIMS and MS/MS spectrum (collision-induced dissociation energy: 20 eV) of **2**. 12](#_Toc190804103)

[Fig. S20. CD spectrum(methanol) of **2**. 13](#_Toc190804104)

[Fig. S21. UV spectrum (acetonitrile) of **2**. 13](#_Toc190804105)

[Fig. S22. ^1^H NMR (CDCl_3_, 600 MHz) of curtachalasin J. 13](#_Toc190804106)

[Fig. S23. ^13^C NMR (CDCl_3_, 600 MHz) of curtachalasin J. 14](#_Toc190804107)

[Fig. S24. LC-MS analysis of the raw EtOAc extraction of the strain Y01 and the standard sample of compound **1**. The presence of compound **1** in crude extraction was validated by comparison of MS/MS data and retention time. Chromatogram condition: Supersil ODS2, 2.1 × 150 mm, 3 μm, 5%−100% acetonitrile (0−30 min), flow rate: 0.2 mL/min. 14](#_Toc190804108)

[Fig. S25. LC-MS analysis of the raw EtOAc extraction of the strain Y01 and the standard sample of compound **2**. The presence of compound **2** in crude extraction was validated by comparison of MS/MS data and retention time. Chromatogram condition: Supersil ODS2, 2.1 × 150 mm, 3 μm, 5%−100% acetonitrile (0−30 min), flow rate: 0.2 mL/min. 15](#_Toc190804109)

[Table S1. Experimental ^13^C NMR chemical shifts (CDCl_3_) of **1** and the predicted values by GFN2NMR for isomers **1a**–**1h** 16](#_Toc190804110)

[Table S2. Experimental ^13^C NMR chemical shifts (CDCl_3_) of **2** and predicted values by GFN2NMR for isomers **2a**–**2h** 17](#_Toc190804111)

[Table S3. Experimental ^13^C NMR chemical shifts (CDCl_3_) of **1** and theoretical values for eight possible epimers from GIAO ^13^C NMR calculations using the STS protocol. 18](#_Toc190804112)

[Table S4. Experimental ^13^C NMR chemical shifts (CDCl_3_) of **2** and theoretical values for eight possible epimers from GIAO ^13^C NMR calculations using the STS protocol. 19](#_Toc190804113)

[Table S5. Calculated *J* coupling constant for eight possible epimers of compound **1**. 21](#_Toc190804114)

[Table S6. Calculated *J* coupling constant for eight possible epimers of compound **2**. 21](#_Toc190804115)

[Table S7. Geometry data of conformers of structure **1a**. 23](#_Toc190804116)

[Table S8. Geometry data of conformers of structure **1b**. 28](#_Toc190804117)

[Table S9. Geometry data of conformers of structure **1c**. 35](#_Toc190804118)

[Table S10. Geometry data of conformers of structure **1d**. 38](#_Toc190804119)

[Table S11. Geometry data of conformers of structure **1e**. 42](#_Toc190804120)

[Table S12. Geometry data of conformers of structure **1f**. 48](#_Toc190804121)

[Table S13. Geometry data of conformers of structure **1g**. 55](#_Toc190804122)

[Table S14. Geometry data of conformers of structure **1h**. 59](#_Toc190804123)

[Table S15. Geometry data of conformers of structure **2a**. 65](#_Toc190804124)

[Table S16. Geometry data of conformers of structure **2b**. 68](#_Toc190804125)

[Table S17. Geometry data of conformers of structure **2c**. 69](#_Toc190804126)

[Table S18. Geometry data of conformers of structure **2d**. 71](#_Toc190804127)

[Table S19. Geometry data of conformers of structure **2e**. 78](#_Toc190804128)

[Table S20. Geometry data of conformers of structure **2f**. 81](#_Toc190804129)

[Table S21. Geometry data of conformers of structure **2g**. 84](#_Toc190804130)

[Table S22. Geometry data of conformers of structure **2h**. 85](#_Toc190804131)

### Fig. S1. ^1^H NMR (CDCl_3_, 600 MHz) of 1.

### Fig. S2. ^13^C NMR (CDCl_3_, 150 MHz) of 1.

### Fig. S3. DEPT 135 spectra of 1.

### Fig. S4. HSQC of 1.

Fig. S5. ^1^H−^1^H COSY of 1.

### Fig. S6. HMBC of 1.

### Fig. S7. NOESY of 1.


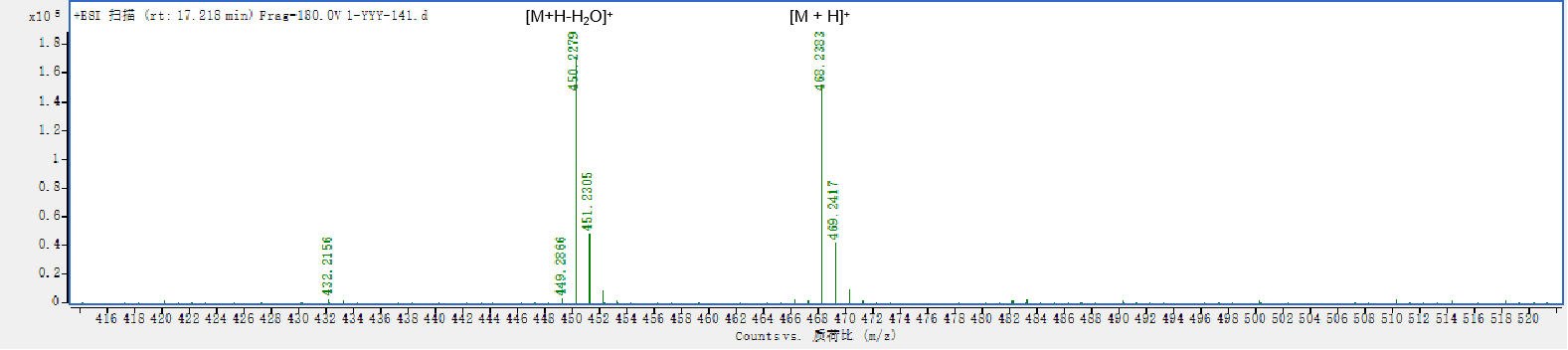


### Fig. S8. HRESIMS and MS/MS spectrum (collision-induced dissociation energy: 20 eV) of 1.


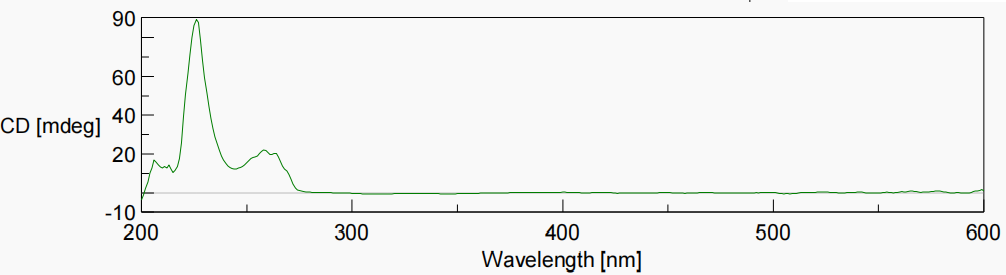


### Fig. S9. CD spectrum(methanol) of 1.


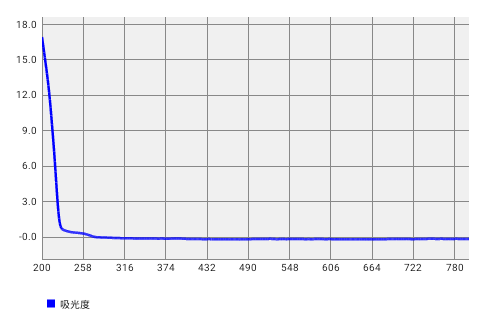


### Fig. S10. UV spectrum (acetonitrile) of 1.


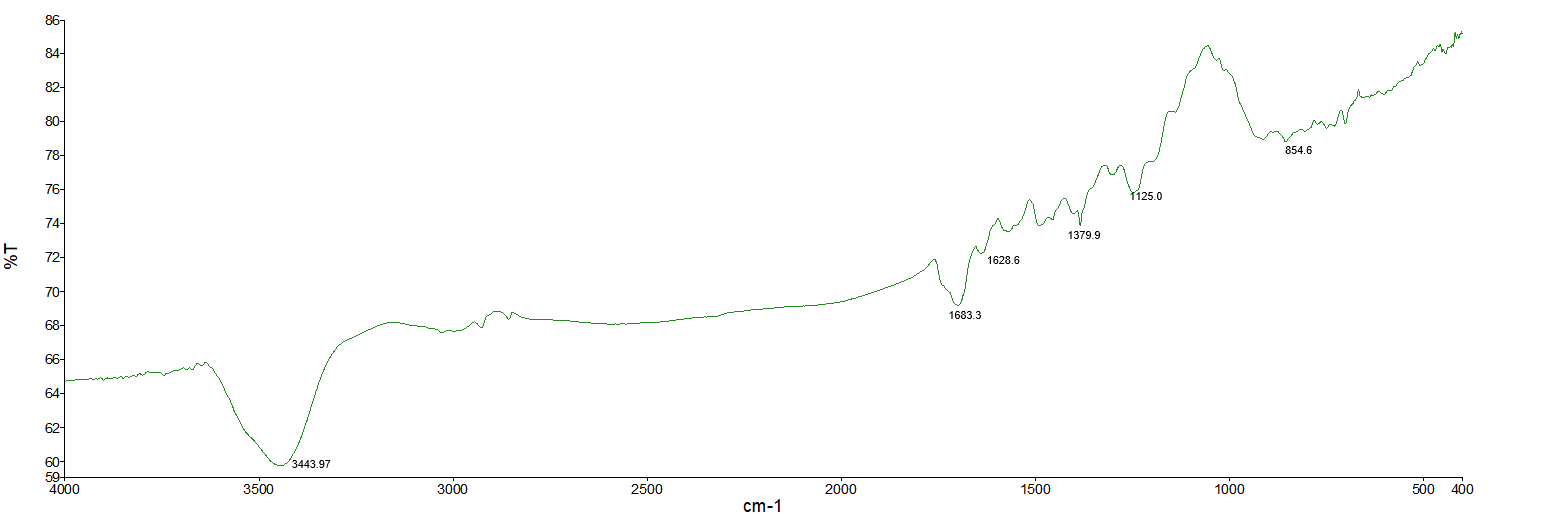


### Fig. S11. IR spectrum of 1.

### Fig. S12. ^1^H NMR (CDCl_3_, 600 MHz) of 2.

### Fig. S13. ^13^C NMR (CDCl_3_, 150 MHz) of 2.

### Fig. S14. DEPT 135 spectra of 2.

### Fig. S15. HSQC of 2.

### Fig. S16. ^1^H−^1^H COSY of 2.

### Fig. S17. HMBC of 2.

### Fig. S18. NOESY of 2.


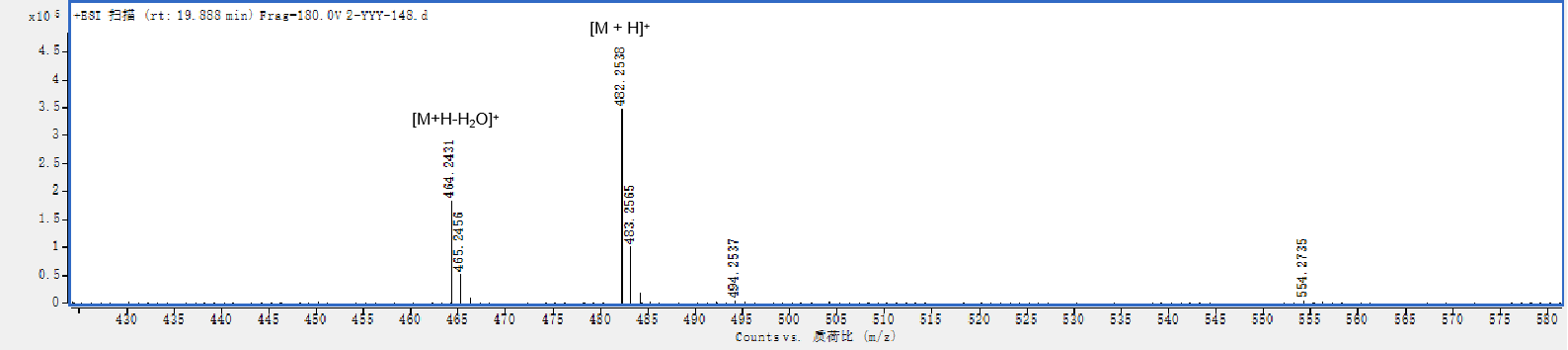


### Fig. S19. HRESIMS and MS/MS spectrum (collision-induced dissociation energy: 20 eV) of 2.


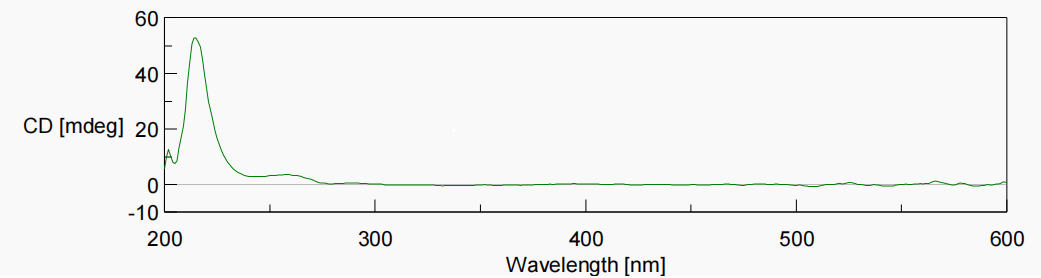


### Fig. S20. CD spectrum(methanol) of 2.


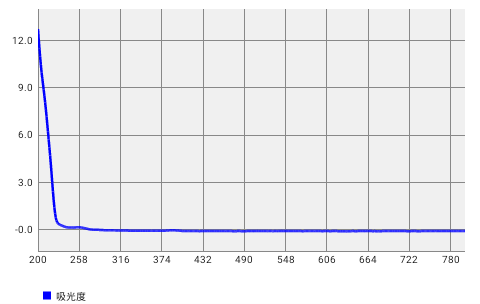


### Fig. S21. UV spectrum (acetonitrile) of 2.

### Fig. S22. ^1^H NMR (CDCl_3_, 600 MHz) of curtachalasin J.

### Fig. S23. ^13^C NMR (CDCl_3_, 600 MHz) of curtachalasin J.


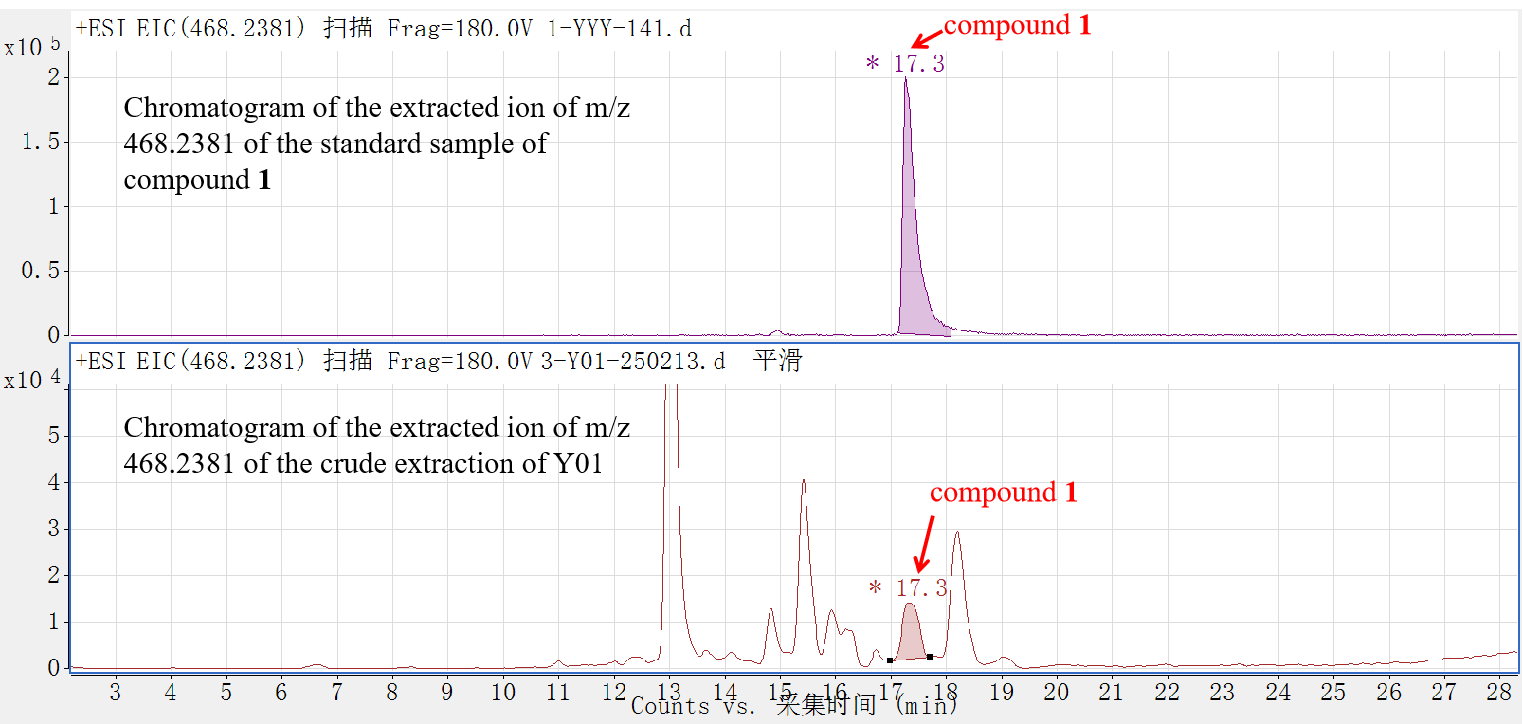


### Fig. S24. LC-MS analysis of the raw EtOAc extraction of the strain Y01 and the standard sample of compound 1. The presence of compound 1 in crude extraction was validated by comparison of MS/MS data and retention time. Chromatogram condition: Supersil ODS2, 2.1 × 150 mm, 3 μm, 5%−100% acetonitrile (0−30 min), flow rate: 0.2 mL/min.


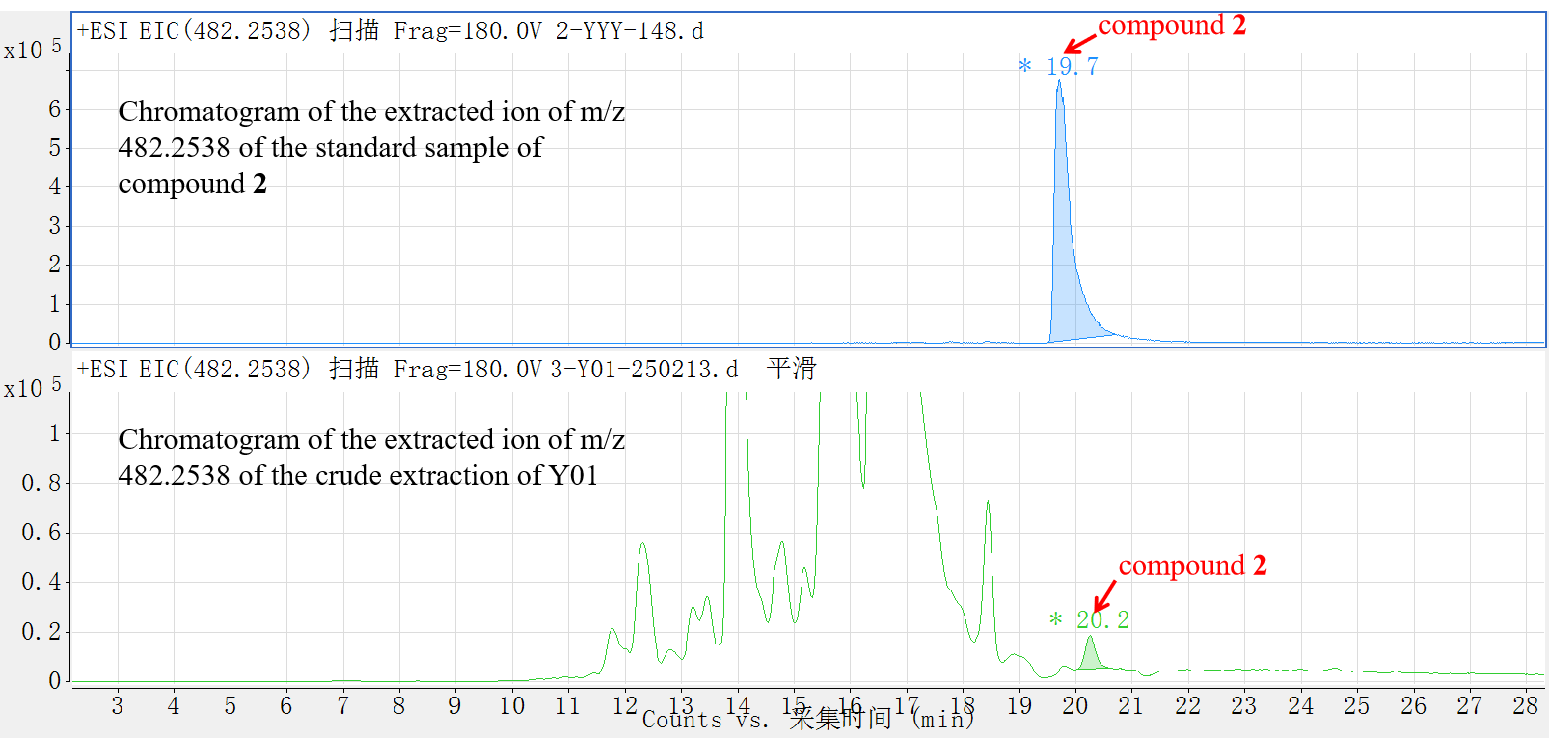


### Fig. S25. LC-MS analysis of the raw EtOAc extraction of the strain Y01 and the standard sample of compound 2. The presence of compound 2 in crude extraction was validated by comparison of MS/MS data and retention time. Chromatogram condition: Supersil ODS2, 2.1 × 150 mm, 3 μm, 5%−100% acetonitrile (0−30 min), flow rate: 0.2 mL/min.

### Table S1. Experimental ^13^C NMR chemical shifts (CDCl_3_) of 1 and the predicted values by GFN2NMR for isomers 1a–1h

|  |  | **MAE** | 2.12 | **MAE** | 2.62 | **MAE** | 2.18 | **MAE** | **1.43** | **MAE** | 2.30 | **MAE** | 2.41 | **MAE** | 2.17 | **MAE** | **1.45** |
| --- | --- | --- | --- | --- | --- | --- | --- | --- | --- | --- | --- | --- | --- | --- | --- | --- | --- |
|  |  | **RMS** | 2.97 | **RMS** | 3.63 | **RMS** | 2.99 | **RMS** | **1.72** | **RMS** | 3.24 | **RMS** | 3.30 | **RMS** | 3.23 | **RMS** | **1.82** |
|  |  | **P_mean_** | 26% | **P_mean_** | 17% | **P_mean_** | 26% | **P_mean_** | **51%** | **P_mean_** | 22% | **P_mean_** | 21% | **P_mean_** | 23% | **P_mean_** | **49%** |
|  |  | **DP4** | 0% | **DP4** | 0% | **DP4** | 0% | **DP4** | **71%** | **DP4** | 0% | **DP4** | 0% | **DP4** | 0% | **DP4** | **29%** |
| **No.** | **Exptl.** | **Calcd. 1a** | **Deviation** | **Calcd. 1b** | **Deviation** | **Calcd. 1c** | **Deviation** | **Calcd. 1d** | **Deviation** | **Calcd. 1e** | **Deviation** | **Calcd. 1f** | **Deviation** | **Calcd. 1g** | **Deviation** | **Calcd. 1h** | **Deviation** |
| 1 | 177.4 | 178.7 | 1.3 | 177.7 | 0.3 | 179.3 | 1.9 | 178.5 | 1.1 | 177.6 | 0.2 | 177.4 | 0.0 | 176.9 | 0.5 | 177.6 | 0.2 |
| 3 | 58.1 | 61.5 | 3.4 | 63.8 | 5.7 | 63.1 | 5.0 | 61.2 | 3.1 | 61.2 | 3.1 | 61.8 | 3.7 | 63.4 | 5.3 | 60.3 | 2.2 |
| 4 | 47.5 | 52.2 | 4.7 | 53.3 | 5.8 | 51.8 | 4.3 | 46.5 | 1.0 | 53.6 | 6.1 | 53.3 | 5.8 | 50.0 | 2.5 | 48.4 | 0.9 |
| 5 | 127.3 | 132.1 | 4.8 | 132.8 | 5.5 | 129.6 | 2.3 | 130.0 | 2.7 | 130.5 | 3.2 | 130.4 | 3.1 | 128.3 | 1.0 | 128.1 | 0.8 |
| 6 | 134.8 | 136.1 | 1.3 | 137.3 | 2.5 | 137.4 | 2.6 | 137.4 | 2.6 | 135.9 | 1.1 | 139.0 | 4.2 | 135.6 | 0.8 | 137.5 | 2.7 |
| 7 | 70.8 | 70.0 | 0.8 | 70.3 | 0.5 | 71.0 | 0.2 | 71.6 | 0.8 | 70.4 | 0.4 | 70.3 | 0.5 | 71.9 | 1.1 | 71.3 | 0.5 |
| 8 | 52.4 | 52.3 | 0.1 | 53.2 | 0.8 | 50.0 | 2.4 | 53.6 | 1.2 | 51.8 | 0.6 | 53.6 | 1.2 | 48.5 | 3.9 | 55.4 | 3.0 |
| 9 | 63.3 | 57.8 | 5.5 | 57.5 | 5.8 | 62.2 | 1.1 | 60.3 | 3.0 | 56.2 | 7.1 | 56.6 | 6.7 | 63.2 | 0.1 | 60.4 | 2.9 |
| 10 | 42.7 | 41.7 | 1.0 | 41.4 | 1.3 | 42.3 | 0.4 | 41.7 | 1.0 | 42.3 | 0.4 | 40.7 | 2.0 | 42.2 | 0.5 | 41.0 | 1.7 |
| 11 | 17.9 | 19.6 | 1.7 | 19.8 | 1.9 | 19.5 | 1.6 | 17.1 | 0.8 | 19.8 | 1.9 | 19.1 | 1.2 | 20.2 | 2.3 | 18.4 | 0.5 |
| 12 | 14.3 | 16.7 | 2.4 | 17.1 | 2.8 | 16.8 | 2.5 | 16.4 | 2.1 | 17.0 | 2.7 | 16.1 | 1.8 | 17.4 | 3.1 | 16.2 | 1.9 |
| 13 | 28.9 | 25.8 | 3.1 | 29.6 | 0.7 | 27.3 | 1.6 | 28.8 | 0.1 | 25.8 | 3.1 | 29.9 | 1.0 | 27.0 | 1.9 | 27.2 | 1.7 |
| 14 | 29.8 | 20.1 | 9.7 | 20.1 | 9.7 | 19.7 | 10.1 | 26.2 | 3.6 | 19.6 | 10.2 | 21.8 | 8.0 | 18.5 | 11.3 | 24.9 | 4.9 |
| 15 | 32.0 | 33.0 | 1.0 | 34.7 | 2.7 | 32.7 | 0.7 | 31.0 | 1.0 | 34.7 | 2.7 | 33.5 | 1.5 | 33.3 | 1.3 | 30.7 | 1.3 |
| 16 | 78.1 | 79.4 | 1.3 | 79.1 | 1.0 | 79.2 | 1.1 | 79.0 | 0.9 | 79.9 | 1.8 | 79.2 | 1.1 | 79.4 | 1.3 | 78.2 | 0.1 |
| 17 | 36.3 | 35.7 | 0.6 | 28.5 | 7.8 | 32.2 | 4.1 | 34.5 | 1.8 | 33.3 | 3.0 | 29.1 | 7.2 | 30.0 | 6.3 | 35.0 | 1.3 |
| 18 | 38.6 | 40.0 | 1.4 | 39.5 | 0.9 | 41.6 | 3.0 | 38.7 | 0.1 | 41.3 | 2.7 | 41.3 | 2.7 | 42.1 | 3.5 | 41.0 | 2.4 |
| 19 | 179.6 | 177.4 | 2.2 | 174.6 | 5.0 | 175.0 | 4.6 | 177.2 | 2.4 | 177.0 | 2.6 | 176.2 | 3.4 | 176.9 | 2.7 | 177.2 | 2.4 |
| 20 | 15.9 | 19.6 | 3.7 | 18.7 | 2.8 | 18.4 | 2.5 | 18.4 | 2.5 | 18.3 | 2.4 | 20.5 | 4.6 | 19.2 | 3.3 | 16.5 | 0.6 |
| 21 | 137.3 | 136.9 | 0.4 | 138.6 | 1.3 | 137.0 | 0.3 | 137.5 | 0.2 | 138.5 | 1.2 | 137.8 | 0.5 | 138.3 | 1.0 | 138.0 | 0.7 |
| 22 | 129.0 | 129.3 | 0.3 | 128.1 | 0.9 | 128.3 | 0.7 | 128.2 | 0.8 | 128.4 | 0.6 | 128.6 | 0.4 | 129.2 | 0.2 | 128.3 | 0.7 |
| 23 | 128.8 | 127.9 | 0.9 | 128.3 | 0.5 | 127.7 | 1.1 | 128.2 | 0.6 | 128.5 | 0.3 | 127.9 | 0.9 | 128.3 | 0.5 | 128.1 | 0.7 |
| 24 | 127.0 | 126.0 | 1.0 | 126.0 | 1.0 | 125.8 | 1.2 | 125.8 | 1.2 | 126.1 | 0.9 | 126.2 | 0.8 | 126.0 | 1.0 | 126.2 | 0.8 |
| 25 | 128.8 | 128.2 | 0.6 | 128.1 | 0.7 | 127.6 | 1.2 | 128.0 | 0.8 | 128.6 | 0.2 | 127.9 | 0.9 | 128.3 | 0.5 | 127.9 | 0.9 |
| 26 | 129.0 | 128.2 | 0.8 | 127.3 | 1.7 | 128.4 | 0.6 | 127.8 | 1.2 | 128.5 | 0.5 | 128.1 | 0.9 | 128.8 | 0.2 | 128.8 | 0.2 |
| 27 | 170.0 | 167.8 | 1.9 | 169.1 | 0.6 | 170.5 | 0.8 | 170.0 | 0.3 | 168.6 | 1.1 | 168.9 | 0.8 | 170.0 | 0.3 | 170.6 | 0.9 |
| 28 | 21.0 | 22.3 | 1.3 | 21.7 | 0.7 | 21.9 | 0.9 | 22.6 | 1.6 | 23.0 | 2.0 | 20.9 | 0.1 | 23.3 | 2.3 | 23.0 | 2.0 |

### Table S2. Experimental ^13^C NMR chemical shifts (CDCl_3_) of 2 and predicted values by GFN2NMR for isomers 2a–2h

|  |  | **MAE** | 2.12 | **MAE** | 2.14 | **MAE** | 1.90 | **MAE** | **1.56** | **MAE** | 2.21 | **MAE** | 2.17 | **MAE** | 1.74 | **MAE** | **1.58** |
| --- | --- | --- | --- | --- | --- | --- | --- | --- | --- | --- | --- | --- | --- | --- | --- | --- | --- |
|  |  | **RMS** | 3.32 | **RMS** | 3.33 | **RMS** | 2.58 | **RMS** | **1.95** | **RMS** | 3.26 | **RMS** | 3.22 | **RMS** | 2.35 | **RMS** | **1.92** |
|  |  | **P_mean_** | 22% | **P_mean_** | 22% | **P_mean_** | 33% | **P_mean_** | **46%** | **P_mean_** | 22% | **P_mean_** | 23% | **P_mean_** | 38% | **P_mean_** | **46%** |
|  |  | **DP4** | 0% | **DP4** | 0% | **DP4** | 0% | **DP4** | **47%** | **DP4** | 0% | **DP4** | 0% | **DP4** | 0% | **DP4** | **53%** |
| **No.** | **Exptl δ** | **Calcd. 2a** | **Deviation** | **Calcd. 2b** | **Deviation** | **Calcd. 2c** | **Deviation** | **Calcd. 2d** | **Deviation** | **Calcd. 2e** | **Deviation** | **Calcd. 2f** | **Deviation** | **Calcd. 2g** | **Deviation** | **Calcd. 2h** | **Deviation** |
| 1 | 176.7 | 176.5 | 0.2 | 176.5 | 0.2 | 177.7 | 1.0 | 179.9 | 3.2 | 176.4 | 0.3 | 176.3 | 0.4 | 178.4 | 1.7 | 178.7 | 2.0 |
| 3 | 57.8 | 61.7 | 3.9 | 61.8 | 4.0 | 62.6 | 4.8 | 61.6 | 3.8 | 61.7 | 3.9 | 61.7 | 3.9 | 62.9 | 5.1 | 61.7 | 3.9 |
| 4 | 47.7 | 52.8 | 5.1 | 52.9 | 5.2 | 49.1 | 1.4 | 47.9 | 0.2 | 53.1 | 5.4 | 53.0 | 5.3 | 49.1 | 1.4 | 47.9 | 0.2 |
| 5 | 127.3 | 128.8 | 1.5 | 128.9 | 1.6 | 128.8 | 1.5 | 128.8 | 1.5 | 129.0 | 1.7 | 129.0 | 1.7 | 129.0 | 1.7 | 128.2 | 0.9 |
| 6 | 134.7 | 139.2 | 4.5 | 139.1 | 4.4 | 136.5 | 1.8 | 136.4 | 1.7 | 139.2 | 4.5 | 139.2 | 4.5 | 135.9 | 1.2 | 136.8 | 2.1 |
| 7 | 70.7 | 69.6 | 1.1 | 69.7 | 1.0 | 69.8 | 0.9 | 68.7 | 2.0 | 69.9 | 0.8 | 69.8 | 0.9 | 69.8 | 0.9 | 69.3 | 1.4 |
| 8 | 52.5 | 51.7 | 0.8 | 51.6 | 0.9 | 51.8 | 0.7 | 55.0 | 2.5 | 51.6 | 0.9 | 51.6 | 0.9 | 50.8 | 1.7 | 55.2 | 2.7 |
| 9 | 63.1 | 55.6 | 7.5 | 55.7 | 7.4 | 60.3 | 2.8 | 58.6 | 4.5 | 55.5 | 7.6 | 55.4 | 7.7 | 60.2 | 2.9 | 60.2 | 2.9 |
| 10 | 42.8 | 42.7 | 0.1 | 42.8 | 0.0 | 41.4 | 1.4 | 41.3 | 1.5 | 43.0 | 0.2 | 43.0 | 0.2 | 42.2 | 0.6 | 41.8 | 1.0 |
| 11 | 17.9 | 19.8 | 1.9 | 19.9 | 2.0 | 20.0 | 2.1 | 18.2 | 0.3 | 19.9 | 2.0 | 20.0 | 2.1 | 19.3 | 1.4 | 18.9 | 1.0 |
| 12 | 14.3 | 16.9 | 2.6 | 17.0 | 2.7 | 17.4 | 3.1 | 16.0 | 1.7 | 17.2 | 2.9 | 17.2 | 2.9 | 17.0 | 2.7 | 16.7 | 2.4 |
| 13 | 28.8 | 26.8 | 2.0 | 26.6 | 2.2 | 27.8 | 1.0 | 28.2 | 0.6 | 26.4 | 2.4 | 26.5 | 2.3 | 29.7 | 0.9 | 26.3 | 2.5 |
| 14 | 30.3 | 18.7 | 11.6 | 18.6 | 11.7 | 21.1 | 9.2 | 27.1 | 3.2 | 19.6 | 10.7 | 19.8 | 10.5 | 22.4 | 7.9 | 26.3 | 4.0 |
| 15 | 32.2 | 32.4 | 0.2 | 32.5 | 0.3 | 29.2 | 3.0 | 29.2 | 3.0 | 32.1 | 0.1 | 32.1 | 0.1 | 29.5 | 2.7 | 30.4 | 1.8 |
| 16 | 77.8 | 79.6 | 1.8 | 79.8 | 2.0 | 78.8 | 1.0 | 78.6 | 0.8 | 79.9 | 2.1 | 79.8 | 2.0 | 78.7 | 0.9 | 79.6 | 1.8 |
| 17 | 36.1 | 36.0 | 0.1 | 36.0 | 0.1 | 34.8 | 1.3 | 36.5 | 0.4 | 34.3 | 1.8 | 34.6 | 1.5 | 34.3 | 1.8 | 33.8 | 2.3 |
| 18 | 39.0 | 39.5 | 0.5 | 39.5 | 0.5 | 41.7 | 2.7 | 39.8 | 0.8 | 39.9 | 0.9 | 40.0 | 1.0 | 40.5 | 1.5 | 39.4 | 0.4 |
| 19 | 177.0 | 173.5 | 3.5 | 173.5 | 3.5 | 174.8 | 2.2 | 174.9 | 2.1 | 173.6 | 3.4 | 173.8 | 3.2 | 174.3 | 2.7 | 174.1 | 2.9 |
| 20 | 16.2 | 19.1 | 2.9 | 19.2 | 3.0 | 19.6 | 3.4 | 19.3 | 3.1 | 18.9 | 2.7 | 18.9 | 2.7 | 19.0 | 2.8 | 18.7 | 2.5 |
| 21 | 137.3 | 137.4 | 0.1 | 137.5 | 0.2 | 138.4 | 1.1 | 137.8 | 0.5 | 137.4 | 0.1 | 137.4 | 0.1 | 138.2 | 0.9 | 137.9 | 0.6 |
| 22 | 129.0 | 128.3 | 0.7 | 128.5 | 0.5 | 128.5 | 0.5 | 128.1 | 0.9 | 128.6 | 0.4 | 128.5 | 0.5 | 128.4 | 0.6 | 128.5 | 0.5 |
| 23 | 128.8 | 128.6 | 0.2 | 128.7 | 0.1 | 128.4 | 0.4 | 128.0 | 0.8 | 128.7 | 0.1 | 128.6 | 0.2 | 128.5 | 0.3 | 128.3 | 0.5 |
| 24 | 127.0 | 126.4 | 0.6 | 126.5 | 0.5 | 125.9 | 1.1 | 125.9 | 1.1 | 126.5 | 0.5 | 126.5 | 0.5 | 126.2 | 0.8 | 126.3 | 0.7 |
| 25 | 128.8 | 128.7 | 0.1 | 128.6 | 0.2 | 128.1 | 0.7 | 128.2 | 0.6 | 128.6 | 0.2 | 128.6 | 0.2 | 128.4 | 0.4 | 128.3 | 0.5 |
| 26 | 129.0 | 128.6 | 0.4 | 128.4 | 0.6 | 127.8 | 1.2 | 128.1 | 0.9 | 128.5 | 0.5 | 128.6 | 0.4 | 128.2 | 0.8 | 128.5 | 0.5 |
| 19-OCH_3_ | 51.7 | 54.0 | 2.3 | 54.0 | 2.3 | 52.6 | 0.9 | 51.9 | 0.2 | 53.8 | 2.1 | 53.8 | 2.1 | 52.2 | 0.5 | 52.2 | 0.5 |
| 27 | 169.7 | 168.9 | 0.8 | 168.8 | 0.9 | 169.5 | 0.2 | 169.1 | 0.6 | 168.7 | 1.0 | 168.8 | 0.9 | 169.5 | 0.2 | 169.2 | 0.5 |
| 28 | 21.0 | 23.2 | 2.2 | 23.0 | 2.0 | 22.7 | 1.7 | 22.3 | 1.3 | 23.3 | 2.3 | 23.1 | 2.1 | 22.7 | 1.7 | 22.1 | 1.1 |

### Table S3. Experimental ^13^C NMR chemical shifts (CDCl_3_) of 1 and theoretical values for eight possible epimers from GIAO ^13^C NMR calculations using the STS protocol.

| **No.** | **Exptl.** | **Calcd. 1a** | **Dev.** | **Calcd. 1b** | **Dev.** | **Calcd. 1c** | **Dev.** | **Calcd. 1d** | **Dev.** | **Calcd. 1e** | **Dev.** | **Calcd. 1f** | **Dev.** | **Calcd. 1g** | **Dev.** | **Calcd. 1h** | **Dev.** |
| --- | --- | --- | --- | --- | --- | --- | --- | --- | --- | --- | --- | --- | --- | --- | --- | --- | --- |
| 1 | 177.4 | 173.8 | 3.6 | 173.9 | 3.6 | 173.9 | 3.5 | 174.4 | 3.0 | 173.7 | 3.8 | 173.8 | 3.6 | 174.2 | 3.2 | 174.3 | 3.1 |
| 3 | 58.1 | 59.9 | 1.8 | 59.0 | 0.9 | 59.6 | 1.5 | 57.6 | 0.5 | 59.4 | 1.3 | 59.2 | 1.1 | 59.1 | 1.0 | 57.9 | 0.2 |
| 4 | 47.5 | 53.5 | 6.0 | 55.3 | 7.8 | 48.2 | 0.7 | 45.8 | 1.7 | 53.0 | 5.5 | 54.7 | 7.2 | 46.9 | 0.6 | 46.6 | 1.0 |
| 5 | 127.3 | 129.3 | 2.0 | 129.7 | 2.4 | 132.1 | 4.8 | 131.9 | 4.6 | 130.3 | 3.0 | 129.5 | 2.2 | 132.1 | 4.8 | 131.4 | 4.1 |
| 6 | 134.8 | 139.6 | 4.8 | 140.8 | 6.0 | 134.8 | 0.0 | 135.6 | 0.8 | 139.2 | 4.4 | 140.1 | 5.3 | 134.8 | 0.0 | 136.5 | 1.7 |
| 7 | 70.8 | 70.7 | 0.1 | 69.5 | 1.3 | 72.6 | 1.8 | 71.9 | 1.1 | 71.1 | 0.3 | 70.4 | 0.4 | 72.0 | 1.2 | 71.0 | 0.2 |
| 8 | 52.4 | 52.6 | 0.2 | 51.6 | 0.9 | 44.3 | 8.1 | 51.7 | 0.8 | 51.9 | 0.5 | 51.0 | 1.4 | 43.7 | 8.8 | 52.1 | 0.3 |
| 9 | 63.3 | 56.5 | 6.8 | 57.8 | 5.5 | 66.1 | 2.8 | 62.7 | 0.6 | 56.3 | 7.0 | 57.4 | 5.9 | 65.4 | 2.1 | 63.2 | 0.1 |
| 10 | 42.7 | 43.8 | 1.1 | 43.0 | 0.3 | 43.6 | 0.9 | 41.5 | 1.2 | 43.2 | 0.5 | 43.1 | 0.4 | 42.6 | 0.1 | 41.4 | 1.3 |
| 11 | 17.9 | 21.4 | 3.5 | 20.0 | 2.1 | 20.5 | 2.6 | 18.0 | 0.1 | 20.6 | 2.7 | 20.0 | 2.1 | 19.4 | 1.5 | 18.3 | 0.4 |
| 12 | 14.4 | 18.9 | 4.6 | 17.3 | 3.0 | 17.6 | 3.3 | 15.2 | 0.9 | 17.9 | 3.6 | 17.2 | 2.9 | 16.6 | 2.3 | 15.3 | 1.0 |
| 13 | 28.9 | 26.3 | 2.7 | 27.0 | 1.9 | 28.9 | 0.0 | 30.0 | 1.1 | 25.4 | 3.5 | 26.7 | 2.2 | 28.5 | 0.5 | 28.9 | 0.0 |
| 14 | 29.8 | 17.3 | 12.5 | 26.3 | 3.5 | 29.4 | 0.4 | 32.0 | 2.2 | 19.2 | 10.6 | 25.1 | 4.7 | 32.1 | 2.3 | 30.8 | 1.0 |
| 15 | 32.0 | 29.9 | 2.1 | 30.4 | 1.6 | 30.1 | 1.9 | 30.7 | 1.3 | 29.8 | 2.2 | 31.2 | 0.8 | 29.3 | 2.7 | 31.8 | 0.2 |
| **16** | **78.1** | **80.2** | **2.1** | **79.2** | **1.1** | **80.6** | **2.5** | **79.1** | **1.0** | **79.6** | **1.5** | **79.7** | **1.6** | **80.2** | **2.1** | **78.6** | **0.5** |
| 17 | 36.3 | 37.2 | 0.9 | 29.3 | 7.1 | 29.4 | 6.9 | 35.8 | 0.5 | 37.7 | 1.4 | 29.8 | 6.5 | 30.7 | 5.6 | 35.4 | 0.9 |
| 18 | 38.6 | 40.0 | 3.7 | 41.8 | 5.5 | 39.4 | 3.1 | 39.7 | 3.4 | 41.4 | 5.1 | 42.8 | 6.5 | 42.4 | 6.1 | 38.4 | 2.1 |
| 19 | 179.6 | 181.9 | 2.3 | 182.5 | 2.9 | 182.4 | 2.8 | 183.6 | 4.0 | 182.1 | 2.5 | 182.4 | 2.8 | 183.0 | 3.4 | 182.0 | 2.4 |
| 20 | 15.9 | 17.0 | 1.1 | 17.8 | 1.9 | 15.1 | 0.8 | 15.5 | 0.4 | 18.7 | 2.8 | 17.8 | 1.9 | 17.1 | 1.2 | 16.1 | 0.2 |
| 21 | 137.3 | 136.4 | 1.0 | 136.9 | 0.4 | 136.4 | 0.9 | 136.1 | 1.2 | 136.4 | 1.0 | 136.6 | 0.7 | 136.2 | 1.1 | 135.9 | 1.4 |
| 22 | 129.0 | 128.2 | 0.8 | 127.9 | 1.2 | 128.7 | 0.4 | 127.2 | 1.8 | 128.2 | 0.8 | 127.0 | 2.0 | 128.7 | 0.3 | 127.8 | 1.2 |
| 23 | 128.8 | 127.1 | 1.7 | 126.8 | 2.0 | 127.3 | 1.5 | 127.1 | 1.7 | 127.1 | 1.7 | 126.9 | 2.0 | 127.2 | 1.6 | 127.0 | 1.8 |
| 24 | 127.0 | 125.3 | 1.7 | 125.0 | 2.1 | 125.6 | 1.4 | 125.2 | 1.8 | 125.4 | 1.7 | 124.9 | 2.1 | 125.5 | 1.5 | 125.4 | 1.6 |
| 25 | 128.8 | 127.2 | 1.6 | 127.0 | 1.8 | 127.4 | 1.4 | 126.8 | 2.0 | 127.2 | 1.6 | 126.8 | 2.0 | 127.3 | 1.5 | 126.9 | 1.9 |
| 26 | 129.0 | 127.3 | 1.7 | 126.7 | 2.3 | 127.9 | 1.1 | 128.9 | 0.1 | 127.2 | 1.8 | 127.5 | 1.5 | 127.7 | 1.3 | 128.3 | 0.7 |
| 27 | 170.0 | 170.0 | 0.3 | 170.1 | 0.4 | 169.8 | 0.1 | 170.2 | 0.5 | 170.1 | 0.4 | 171.0 | 1.3 | 169.9 | 0.2 | 172.1 | 2.4 |
| 28 | 21.0 | 23.0 | 2.0 | 21.7 | 0.7 | 22.4 | 1.4 | 20.0 | 1.0 | 22.2 | 1.2 | 21.7 | 0.7 | 21.5 | 0.5 | 20.5 | 0.5 |
|  |  | **MAE** | 2.69 | **MAE** | 2.59 | **MAE** | 2.10 | **MAE** | 1.46 | **MAE** | 2.67 | **MAE** | 2.65 | **MAE** | 2.13 | **MAE** | **1.19** |
|  |  | **RMS** | 3.70 | **RMS** | 3.29 | **RMS** | 2.85 | **RMS** | 1.84 | **RMS** | 3.51 | **RMS** | 3.31 | **RMS** | 2.95 | **RMS** | **1.55** |
|  |  | **Pmean** | 0.00% | **Pmean** | 0.91% | **Pmean** | 2.37% | **Pmean** | 19.60% | **Pmean** | 0.00% | **Pmean** | 0.80% | **Pmean** | 1.96% | **Pmean** | **33.41%** |
|  |  | **Prel** | 0.00% | **Prel** | 0.00% | **Prel** | 0.00% | **Prel** | 0.00% | **Prel** | 0.00% | **Prel** | 0.00% | **Prel** | 0.00% | **Prel** | **100.00%** |

### Table S4. Experimental ^13^C NMR chemical shifts (CDCl_3_) of 2 and theoretical values for eight possible epimers from GIAO ^13^C NMR calculations using the STS protocol.

| **No.** | **Exptl.** | **Calcd. 2a** | **Deviation** | **Calcd. 2b** | **Deviation** | **Calcd. 2c** | **Deviation** | **Calcd. 2d** | **Deviation** | **Calcd. 2e** | **Deviation** | **Calcd. 2f** | **Deviation** | **Calcd. 2g** | **Deviation** | **Calcd. 2h** | **Deviation** |
| --- | --- | --- | --- | --- | --- | --- | --- | --- | --- | --- | --- | --- | --- | --- | --- | --- | --- |
| 1 | 176.7 | 174.0 | 2.7 | 173.8 | 2.9 | 174.2 | 2.5 | 175.0 | 1.7 | 174.0 | 2.7 | 174.0 | 2.7 | 174.5 | 2.2 | 174.8 | 1.9 |
| 3 | 57.8 | 60.0 | 2.2 | 58.9 | 1.1 | 59.7 | 1.9 | 58.0 | 0.2 | 59.3 | 1.5 | 59.1 | 1.3 | 59.2 | 1.4 | 58.3 | 0.5 |
| 4 | 47.7 | 53.4 | 5.7 | 55.9 | 8.2 | 49.9 | 2.2 | 46.7 | 1.0 | 52.9 | 5.2 | 55.6 | 7.9 | 47.0 | 0.7 | 47.2 | 0.5 |
| 5 | 127.3 | 128.8 | 1.5 | 129.0 | 1.7 | 128.9 | 1.6 | 129.4 | 2.1 | 129.0 | 1.7 | 128.9 | 1.6 | 129.1 | 1.8 | 129.6 | 2.3 |
| 6 | 134.7 | 140.2 | 5.5 | 141.7 | 7.0 | 139.0 | 4.3 | 138.2 | 3.5 | 139.9 | 5.2 | 141.0 | 6.3 | 139.1 | 4.4 | 138.0 | 3.3 |
| 7 | 70.7 | 70.1 | 0.6 | 68.7 | 2.0 | 70.5 | 0.2 | 69.9 | 0.8 | 69.7 | 1.0 | 69.8 | 0.9 | 70.0 | 0.7 | 70.2 | 0.5 |
| 8 | 52.5 | 52.6 | 0.1 | 51.9 | 0.6 | 45.4 | 7.1 | 51.8 | 0.7 | 52.1 | 0.4 | 51.6 | 0.9 | 45.2 | 7.3 | 52.4 | 0.1 |
| 9 | 63.1 | 56.5 | 6.6 | 57.9 | 5.2 | 66.3 | 3.2 | 63.3 | 0.2 | 56.0 | 7.1 | 58.1 | 5.0 | 65.2 | 2.1 | 63.6 | 0.5 |
| 10 | 42.8 | 43.8 | 1.0 | 43.2 | 0.4 | 44.0 | 1.2 | 41.7 | 1.1 | 43.2 | 0.4 | 43.4 | 0.6 | 42.8 | 0.0 | 42.0 | 0.8 |
| 11 | 17.9 | 21.2 | 3.3 | 20.1 | 2.2 | 20.5 | 2.6 | 18.0 | 0.1 | 20.5 | 2.6 | 20.5 | 2.6 | 19.5 | 1.6 | 18.5 | 0.6 |
| 12 | 14.3 | 18.8 | 4.5 | 17.6 | 3.3 | 17.8 | 3.5 | 15.3 | 1.0 | 18.0 | 3.7 | 17.8 | 3.5 | 17.2 | 2.9 | 15.8 | 1.5 |
| 13 | 28.8 | 26.0 | 2.8 | 26.3 | 2.5 | 28.2 | 0.6 | 29.4 | 0.6 | 26.5 | 2.3 | 26.7 | 2.1 | 27.3 | 1.5 | 27.9 | 0.9 |
| 14 | 30.3 | 17.1 | 13.2 | 26.3 | 4.0 | 28.0 | 2.3 | 31.0 | 0.7 | 18.9 | 11.4 | 23.6 | 6.7 | 30.9 | 0.6 | 30.0 | 0.3 |
| 15 | 32.2 | 30.6 | 1.6 | 30.7 | 1.5 | 29.6 | 2.6 | 31.2 | 1.0 | 29.5 | 2.7 | 31.6 | 0.6 | 29.2 | 3.0 | 32.7 | 0.5 |
| 16 | 77.8 | 80.0 | 2.2 | 79.1 | 1.3 | 80.8 | 3.0 | 78.6 | 0.8 | 79.7 | 1.9 | 79.6 | 1.8 | 80.8 | 3.0 | 78.2 | 0.4 |
| 17 | 36.1 | 37.0 | 0.9 | 29.4 | 6.7 | 29.4 | 6.7 | 35.5 | 0.6 | 37.3 | 1.2 | 28.5 | 7.6 | 30.7 | 5.4 | 35.4 | 0.7 |
| 18 | 39.0 | 41.3 | 2.3 | 42.7 | 3.7 | 40.2 | 1.2 | 41.0 | 2.0 | 43.4 | 4.4 | 43.2 | 4.2 | 42.1 | 3.1 | 39.5 | 0.5 |
| 19 | 177.0 | 177.8 | 0.8 | 179.0 | 2.0 | 177.5 | 0.5 | 178.0 | 1.0 | 178.3 | 1.3 | 178.6 | 1.6 | 179.0 | 2.0 | 177.6 | 0.6 |
| 20 | 16.2 | 17.3 | 1.1 | 18.2 | 2.0 | 15.7 | 0.5 | 16.4 | 0.2 | 19.0 | 2.8 | 17.9 | 1.7 | 19.1 | 2.9 | 15.5 | 0.7 |
| 21 | 137.3 | 136.6 | 0.7 | 136.9 | 0.4 | 136.7 | 0.6 | 136.6 | 0.7 | 136.6 | 0.7 | 136.6 | 0.7 | 136.2 | 1.1 | 136.6 | 0.7 |
| 22 | 129.0 | 127.1 | 1.9 | 127.5 | 1.5 | 127.3 | 1.7 | 128.5 | 0.5 | 128.6 | 0.4 | 128.3 | 0.7 | 127.3 | 1.7 | 128.3 | 0.7 |
| 23 | 128.8 | 127.0 | 1.8 | 126.9 | 1.9 | 127.3 | 1.5 | 127.3 | 1.5 | 127.4 | 1.4 | 127.1 | 1.7 | 126.9 | 1.9 | 127.4 | 1.4 |
| 24 | 127.0 | 125.3 | 1.7 | 124.9 | 2.1 | 125.6 | 1.4 | 125.6 | 1.4 | 125.4 | 1.6 | 125.0 | 2.0 | 125.3 | 1.7 | 125.6 | 1.4 |
| 25 | 128.8 | 127.3 | 1.5 | 126.8 | 2.0 | 127.5 | 1.3 | 127.3 | 1.5 | 127.0 | 1.8 | 126.8 | 2.0 | 127.2 | 1.6 | 127.3 | 1.5 |
| 26 | 129.0 | 128.4 | 0.6 | 126.9 | 2.1 | 128.9 | 0.1 | 127.9 | 1.1 | 127.0 | 2.0 | 126.6 | 2.4 | 129.2 | 0.2 | 128.1 | 0.9 |
| 19-OCH_3_ | 51.7 | 54.4 | 2.7 | 53.2 | 1.5 | 54.5 | 2.8 | 52.6 | 0.9 | 53.8 | 2.1 | 53.6 | 1.9 | 53.8 | 2.1 | 52.7 | 1.0 |
| 27 | 169.7 | 169.8 | 0.1 | 169.9 | 0.2 | 169.9 | 0.2 | 170.9 | 1.2 | 170.2 | 0.5 | 169.8 | 0.1 | 169.7 | 0.0 | 171.3 | 1.6 |
| 28 | 21.0 | 22.9 | 1.9 | 21.6 | 0.6 | 22.0 | 1.0 | 20.2 | 0.8 | 22.1 | 1.1 | 22.0 | 1.0 | 21.7 | 0.7 | 20.6 | 0.4 |
|  |  | **MAE** | 2.55 | **MAE** | 2.51 | **MAE** | 2.08 | **MAE** | 1.03 | **MAE** | 2.55 | **MAE** | 2.58 | **MAE** | 2.05 | **MAE** | 0.95 |
|  |  | **RMS** | 3.65 | **RMS** | 3.22 | **RMS** | 2.69 | **RMS** | 1.24 | **RMS** | 3.46 | **RMS** | 3.36 | **RMS** | 2.60 | **RMS** | 1.17 |
|  |  | **Pmean** | 0% | **Pmean** | 1% | **Pmean** | 3% | **Pmean** | 42% | **Pmean** | 0% | **Pmean** | 1% | **Pmean** | 4% | **Pmean** | 47% |
|  |  | **Prel** | 0% | **Prel** | 0% | **Prel** | 0% | **Prel** | 5% | **Prel** | 0% | **Prel** | 0% | **Prel** | 0% | **Prel** | 95% |

### Table S5. Calculated *J* coupling constant for eight possible epimers of compound 1.

| **No.** | **Exptl.** | **Calcd. 1a** | **Dev.** | **Calcd. 1b** | **Dev.** | **Calcd. 1c** | **Dev.** | **Calcd. 1d** | **Dev.** | **Calcd. 1e** | **Dev.** | **Calcd. 1f** | **Dev.** | **Calcd. 1g** | **Dev.** | **Calcd. 1h** | **Dev.** |
| --- | --- | --- | --- | --- | --- | --- | --- | --- | --- | --- | --- | --- | --- | --- | --- | --- | --- |
| H-3/H-4 | 1.8 | 0.48 | 1.32 | 0.48 | 1.32 | 0.56 | 1.24 | 0.56 | 1.25 | 0.48 | 1.32 | 0.48 | 1.32 | 0.57 | 1.23 | 0.71 | 1.09 |
| H-7/H-8 | 10.5 | 10.40 | 0.10 | 11.12 | 0.62 | 9.96 | 0.55 | 10.54 | 0.04 | 10.66 | 0.16 | 10.90 | 0.40 | 10.02 | 0.48 | 10.67 | 0.17 |
| H-8/H-13 | 4.2 | 4.66 | 0.46 | 6.08 | 1.88 | 4.91 | 0.71 | 4.09 | 0.11 | 4.80 | 0.60 | 5.72 | 1.52 | 4.83 | 0.63 | 4.21 | 0.01 |
| H-13/H-14 | 3.8 | 3.56 | 0.24 | 8.52 | 4.72 | 7.58 | 3.78 | 3.34 | 0.47 | 3.46 | 0.35 | 8.93 | 5.13 | 7.63 | 3.83 | 3.27 | 0.54 |
| H-13/H-15 | 7.7 | 5.92 | 1.78 | 6.43 | 1.27 | 7.39 | 0.31 | 7.43 | 0.27 | 5.95 | 1.75 | 6.50 | 1.20 | 7.40 | 0.30 | 7.45 | 0.25 |
| H-14/H-15 | 3.3 | 3.14 | 0.16 | 8.91 | 5.61 | 8.52 | 5.22 | 3.50 | 0.20 | 3.09 | 0.21 | 8.60 | 5.30 | 8.42 | 5.12 | 3.48 | 0.18 |
| H-15/H-16 | 5.6 | 0.44 | 5.16 | 0.22 | 5.38 | 5.47 | 0.13 | 4.99 | 0.61 | 0.46 | 5.14 | 0.20 | 5.40 | 5.47 | 0.13 | 4.97 | 0.64 |
| H-18/H-20 | 7.0 | 7.00 | 0.10 | 6.88 | 0.02 | 7.09 | 0.19 | 6.79 | 0.11 | 6.89 | 0.01 | 6.84 | 0.06 | 6.83 | 0.07 | 7.10 | 0.20 |
| H-22/H-23 | 7.6 | 6.75 | 0.85 | 6.72 | 0.89 | 6.76 | 0.84 | 6.81 | 0.79 | 6.76 | 0.85 | 6.77 | 0.83 | 6.78 | 0.82 | 6.79 | 0.81 |
| H-23/H-24 | 7.6 | 6.61 | 0.99 | 6.64 | 0.96 | 6.63 | 0.97 | 6.57 | 1.03 | 6.63 | 0.97 | 6.59 | 1.01 | 6.63 | 0.97 | 6.63 | 0.97 |
| H-24/H-25 | 7.6 | 6.60 | 1.00 | 6.58 | 1.02 | 6.58 | 1.02 | 6.62 | 0.98 | 6.60 | 1.00 | 6.63 | 0.97 | 6.58 | 1.02 | 6.61 | 0.99 |
| H-25/H-26 | 7.6 | 6.79 | 0.81 | 6.80 | 0.80 | 6.78 | 0.82 | 6.76 | 0.84 | 6.80 | 0.80 | 6.73 | 0.87 | 6.81 | 0.79 | 6.78 | 0.82 |
|  |  | **MAE** | 1.08 | **MAE** | 2.04 | **MAE** | 1.31 | **MAE** | **0.56** | **MAE** | 1.10 | **MAE** | 2.00 | **MAE** | 1.28 | **MAE** | **0.56** |
|  |  | **RMS** | 1.71 | **RMS** | 2.79 | **RMS** | 1.99 | **RMS** | **0.69** | **RMS** | 1.71 | **RMS** | 2.78 | **RMS** | 1.97 | **RMS** | **0.66** |

### Table S6. Calculated *J* coupling constant for eight possible epimers of compound 2.

| **No.** | **Exptl** | **Calcd. 2a** | **Dev.** | **Calcd. 2b** | **Dev.** | **Calcd. 2c** | **Dev.** | **Calcd. 2d** | **Dev.** | **Calcd. 2e** | **Dev.** | **Calcd. 2f** | **Dev.** | **Calcd. 2g** | **Dev.** | **Calcd. 2h** | **Dev.** |
| --- | --- | --- | --- | --- | --- | --- | --- | --- | --- | --- | --- | --- | --- | --- | --- | --- | --- |
| H-3/H-4 | 1.8 | 0.48 | 1.32 | 0.48 | 1.32 | 0.56 | 1.24 | 0.56 | 1.25 | 0.48 | 1.32 | 0.48 | 1.32 | 0.57 | 1.23 | 0.71 | 1.09 |
| H-7/H-8 | 10.5 | 10.40 | 0.30 | 11.12 | 0.42 | 9.96 | 0.74 | 10.54 | 0.16 | 10.66 | 0.04 | 10.90 | 0.20 | 10.02 | 0.68 | 10.67 | 0.03 |
| H-8/H-13 | 4.3 | 4.66 | 0.36 | 6.08 | 1.78 | 4.91 | 0.61 | 4.09 | 0.21 | 4.80 | 0.50 | 5.72 | 1.42 | 4.83 | 0.53 | 4.21 | 0.09 |
| H-13/H-14 | 3.4 | 3.56 | 0.26 | 8.52 | 5.22 | 7.58 | 4.28 | 3.34 | 0.04 | 3.46 | 0.16 | 8.93 | 5.63 | 7.63 | 4.33 | 3.27 | 0.03 |
| H-13/H-15 | 7.7 | 5.92 | 1.68 | 6.43 | 1.17 | 7.39 | 0.21 | 7.43 | 0.17 | 5.95 | 1.65 | 6.50 | 1.10 | 7.40 | 0.20 | 7.45 | 0.15 |
| H-14/H-15 | 3.4 | 3.14 | 0.26 | 8.91 | 5.51 | 8.52 | 5.12 | 3.50 | 0.10 | 3.09 | 0.31 | 8.60 | 5.20 | 8.42 | 5.02 | 3.48 | 0.08 |
| H-15/H-16 | 5.6 | 0.44 | 5.16 | 0.22 | 5.38 | 5.47 | 0.13 | 4.99 | 0.61 | 0.46 | 5.14 | 0.20 | 5.40 | 5.47 | 0.13 | 4.97 | 0.64 |
| H-18/H-20 | 7.0 | 7.00 | 0.00 | 6.88 | 0.12 | 7.09 | 0.09 | 6.79 | 0.21 | 6.89 | 0.11 | 6.84 | 0.16 | 6.83 | 0.17 | 7.10 | 0.10 |
| H-22/H-23 | 7.3 | 6.75 | 0.55 | 6.72 | 0.59 | 6.76 | 0.54 | 6.81 | 0.49 | 6.76 | 0.55 | 6.77 | 0.53 | 6.78 | 0.52 | 6.79 | 0.51 |
| H-23/H-24 | 7.3 | 6.61 | 0.69 | 6.64 | 0.66 | 6.63 | 0.67 | 6.57 | 0.73 | 6.63 | 0.67 | 6.59 | 0.71 | 6.63 | 0.67 | 6.63 | 0.67 |
| H-24/H-25 | 7.3 | 6.60 | 0.70 | 6.58 | 0.72 | 6.58 | 0.72 | 6.62 | 0.68 | 6.60 | 0.70 | 6.63 | 0.67 | 6.58 | 0.72 | 6.61 | 0.69 |
| H-25/H-26 | 7.3 | 6.79 | 0.51 | 6.80 | 0.50 | 6.78 | 0.52 | 6.76 | 0.54 | 6.80 | 0.50 | 6.73 | 0.57 | 6.81 | 0.49 | 6.78 | 0.52 |
|  |  | **MAE** | 0.98 | **MAE** | 1.95 | **MAE** | 1.24 | **MAE** | **0.43** | **MAE** | 0.97 | **MAE** | 1.91 | **MAE** | 1.22 | **MAE** | **0.38** |
|  |  | **RMS** | 1.66 | **RMS** | 2.81 | **RMS** | 2.01 | **RMS** | **0.55** | **RMS** | 1.65 | **RMS** | 2.81 | **RMS** | 2.00 | **RMS** | **0.51** |

### Table S7. Geometry data of conformers of structure 1a.

YYY-141a-c4 , delta G = 0.0000 kcal/mol, population = 65.11 %

C -0.402196 -0.003471 -1.167403

C -2.405342 0.426909 0.035967

C -1.217696 1.091806 0.777526

C -1.107219 2.560298 0.369646

C -0.034456 3.104095 -0.229703

C 1.273859 2.350505 -0.407533

C 1.266223 1.172996 0.542546

C 0.017989 0.310978 0.298040

C -3.080596 -0.708113 0.823796

C -2.349197 3.373148 0.662092

C -0.021462 4.516819 -0.748850

C 2.444285 0.239496 0.666101

C 2.618440 -0.873547 -0.324578

C 1.885011 -1.134513 0.966099

C 0.371348 -1.038594 0.992923

C 3.999059 -1.450916 -0.516369

C 4.853250 -0.632626 -1.520944

C 5.049474 0.768796 -0.989125

C 4.255959 -0.613015 -2.925484

C -4.134900 -1.412723 0.012947

C -3.833977 -2.591956 -0.670418

C -4.788657 -3.218088 -1.465933

C -6.061758 -2.671027 -1.589537

C -6.373995 -1.495762 -0.911330

C -5.416741 -0.872995 -0.117952

C -0.315792 -2.088790 3.037815

C -0.771299 -1.809421 4.441507

H 5.957477 1.719704 0.372307

N -1.751950 -0.025355 -1.182313

O 0.304008 -0.237666 -2.138751

H -1.353615 1.012828 1.855952

H 1.130744 1.604775 1.540801

O 2.360372 3.242617 -0.123183

O 4.511211 1.771742 -1.408540

O 5.897964 0.802515 0.058483

H 3.310574 0.592574 1.208829

H 2.341639 -1.789328 1.696193

O -0.056669 -0.941085 2.379715

O -0.206736 -3.185587 2.539716

H -7.364728 -1.066778 -0.997185

H -6.806973 -3.158418 -2.205480

H -4.538552 -4.134389 -1.986149

H -2.845016 -3.023859 -0.574380

H -5.668176 0.039559 0.411059

H -3.521891 -0.267852 1.721274

H -2.328873 -1.425554 1.149221

H -3.171073 1.156883 -0.223244

H 1.366956 2.004846 -1.442937

H -0.128586 -1.895642 0.547030

H -2.252729 -0.257699 -2.026278

H -2.999924 3.472099 -0.212196

H -2.939090 2.901028 1.450205

H -2.101304 4.379717 0.996919

H 0.540777 4.562718 -1.683580

H -1.023532 4.898430 -0.934604

H 0.482367 5.192578 -0.053097

H 3.147629 2.874025 -0.552780

H 2.021885 -0.813858 -1.221518

H 3.929727 -2.471211 -0.902098

H 4.524306 -1.505648 0.439835

H 5.842928 -1.095461 -1.548547

H 4.915727 -0.097553 -3.624253

H 4.115342 -1.635802 -3.278128

H 3.291077 -0.108661 -2.940926

H -0.049146 -1.171392 4.951438

H -1.722290 -1.274257 4.413712

H -0.893756 -2.744115 4.981455

YYY-141a-c3 , delta G = 0.7367 kcal/mol, population = 18.75 %

C 0.196415 -0.183581 -1.883997

C -2.132581 -0.224856 -1.407022

C -1.386041 0.418014 -0.208147

C -1.450625 1.938688 -0.329963

C -0.379095 2.736649 -0.469420

C 1.043725 2.222856 -0.324396

C 0.985608 0.900935 0.410079

C 0.066286 -0.078731 -0.337006

C -2.859841 -1.541693 -1.085052

C -2.859659 2.491670 -0.323236

C -0.494384 4.212811 -0.738701

C 2.232745 0.147119 0.798972

C 2.920018 -0.732203 -0.203296

C 1.894003 -1.326378 0.726343

C 0.451440 -1.460424 0.271760

C 4.378171 -1.054982 0.012522

C 5.322155 0.042984 -0.545804

C 5.062171 1.344385 0.177834

C 5.199002 0.215860 -2.057272

C -4.084501 -1.293151 -0.243695

C -5.238086 -0.752788 -0.817793

C -6.351393 -0.458268 -0.039318

C -6.329903 -0.700944 1.331668

C -5.189267 -1.243204 1.913854

C -4.077626 -1.534493 1.129411

C -0.462848 -2.916121 1.940652

C -1.227934 -2.963238 3.234236

H 5.299516 2.172404 1.862607

N -1.049512 -0.388671 -2.361826

O 1.201676 -0.138929 -2.580069

H -1.837845 0.105565 0.731736

H 0.471817 1.116573 1.353637

O 1.809277 3.189382 0.408221

O 4.488890 2.311036 -0.279012

O 5.523637 1.324841 1.444707

H 2.802503 0.522222 1.638405

H 2.229131 -2.025241 1.480761

O -0.370268 -1.660728 1.459031

O 0.024742 -3.879899 1.396236

H -5.163256 -1.439898 2.978533

H -7.196765 -0.472603 1.938781

H -7.237235 -0.041449 -0.502320

H -5.264934 -0.562848 -1.884881

H -3.192458 -1.952274 1.587640

H -2.179384 -2.227778 -0.579950

H -3.145415 -2.010630 -2.030069

H -2.866104 0.466111 -1.820307

H 1.499575 2.092798 -1.312141

H 0.274705 -2.294684 -0.403803

H -1.200569 -0.497510 -3.353384

H -3.555476 1.767314 0.102934

H -2.926265 3.404195 0.268973

H -3.220513 2.731090 -1.328152

H -1.462622 4.481141 -1.156983

H -0.337310 4.797800 0.171004

H 0.281165 4.524017 -1.441628

H 2.743267 3.013575 0.215361

H 2.628685 -0.603874 -1.233645

H 4.634644 -1.991610 -0.489030

H 4.582174 -1.195663 1.076371

H 6.342426 -0.255208 -0.291914

H 5.379845 -0.739670 -2.551653

H 4.207197 0.566188 -2.338586

H 5.927598 0.937152 -2.428979

H -1.968786 -3.760626 3.186384

H -0.528372 -3.208752 4.035540

H -1.705736 -2.013559 3.460877

YYY-141a-c4-gfn0 , delta G = 1.5098 kcal/mol, population = 5.08 %

C -0.305276 0.241601 -1.134049

C -2.388159 0.323712 0.007105

C -1.273712 0.843482 0.951568

C -1.211015 2.368546 0.886015

C -0.139544 3.066480 0.471465

C 1.206747 2.408705 0.206552

C 1.216955 1.057894 0.885687

C 0.020294 0.231749 0.387646

C -3.054556 -0.978767 0.480295

C -2.498824 3.056986 1.278845

C -0.170356 4.557606 0.259606

C 2.427098 0.158868 0.879534

C 2.715587 -0.703728 -0.313240

C 1.915940 -1.264643 0.835421

C 0.400300 -1.226475 0.785951

C 4.140411 -1.169919 -0.511272

C 5.012825 -0.137915 -1.277960

C 4.926041 1.173036 -0.518936

C 4.628365 -0.036702 -2.753418

C -4.023873 -1.520370 -0.536066

C -5.318324 -1.004832 -0.636694

C -6.194740 -1.467077 -1.612316

C -5.787444 -2.454258 -2.505765

C -4.501102 -2.975916 -2.413950

C -3.627447 -2.510885 -1.435857

C -0.348525 -2.731046 2.498365

C -0.880583 -2.790406 3.901505

H 3.797084 2.706570 -0.388444

N -1.649735 0.176460 -1.237541

O 0.465120 0.261863 -2.083265

H -1.463771 0.520244 1.974710

H 1.005912 1.252624 1.945731

O 2.301802 3.242916 0.648077

O 5.505663 1.381762 0.526917

O 4.082946 2.057480 -1.069223

H 3.246155 0.416923 1.535436

H 2.357272 -2.047063 1.438379

O -0.106647 -1.455900 2.129853

O -0.168238 -3.681900 1.773607

H -4.177658 -3.747529 -3.101486

H -6.469803 -2.816990 -3.264091

H -7.196772 -1.060647 -1.672804

H -5.643981 -0.239532 0.059103

H -2.627918 -2.922930 -1.366105

H -3.569032 -0.762417 1.419727

H -2.290694 -1.725325 0.691593

H -3.170208 1.068954 -0.132171

H 1.381093 2.303391 -0.864798

H -0.035657 -1.976426 0.129758

H -2.093931 0.120563 -2.141241

H -3.107461 2.403889 1.906936

H -2.310122 3.971414 1.840114

H -3.106467 3.328272 0.410320

H 0.243261 5.099899 1.114635

H 0.444644 4.823604 -0.602044

H -1.179128 4.925858 0.084162

H 2.285218 3.305961 1.611747

H 2.178446 -0.466491 -1.218628

H 4.158277 -2.106985 -1.074045

H 4.602188 -1.367876 0.458936

H 6.050677 -0.466615 -1.194325

H 3.586045 0.253919 -2.877579

H 5.245231 0.697107 -3.273984

H 4.774599 -1.005333 -3.235103

H -0.190660 -2.293700 4.584430

H -1.833604 -2.261013 3.952633

H -1.019639 -3.826048 4.198477

YYY-141a-c2-gfn0 , delta G = 1.7476 kcal/mol, population = 3.40 %

C 0.275241 0.403732 -1.803642

C -2.058175 0.119559 -1.447027

C -1.370919 0.394560 -0.082333

C -1.505969 1.875851 0.258899

C -0.470161 2.722256 0.387708

C 0.972187 2.239539 0.415112

C 0.968538 0.760240 0.731210

C 0.105933 0.020149 -0.305273

C -2.728369 -1.259637 -1.566252

C -2.937024 2.348586 0.389705

C -0.651819 4.208371 0.554372

C 2.237833 -0.031800 0.916366

C 2.992255 -0.540680 -0.276197

C 1.968320 -1.424451 0.388818

C 0.543462 -1.465199 -0.134035

C 4.460234 -0.852325 -0.090705

C 5.370349 0.393571 -0.273832

C 4.875070 1.456654 0.689200

C 5.442259 0.850451 -1.730063

C -3.985471 -1.325526 -0.738592

C -5.138936 -0.655035 -1.153983

C -6.286302 -0.656701 -0.369571

C -6.299733 -1.332441 0.847980

C -5.159567 -2.007827 1.269686

C -4.013522 -2.001379 0.480427

C -0.345720 -3.393368 0.976373

C -1.120581 -3.860213 2.177059

H 3.555183 2.832700 0.781495

N -0.947286 0.298593 -2.366875

O 1.291646 0.712432 -2.408908

H -1.828688 -0.207336 0.700848

H 0.422537 0.657364 1.678183

O 1.764255 2.991732 1.362705

O 5.072712 1.420843 1.886354

O 4.126519 2.403057 0.106160

H 2.775578 0.096599 1.844900

H 2.312770 -2.306618 0.911479

O -0.292081 -2.047462 0.908548

O 0.182112 -4.125060 0.171376

H -5.160915 -2.541118 2.212275

H -7.193293 -1.335673 1.459266

H -7.171601 -0.133457 -0.709024

H -5.138757 -0.128834 -2.101925

H -3.129641 -2.526997 0.813558

H -2.029550 -2.040643 -1.265868

H -2.965632 -1.429753 -2.619454

H -2.814410 0.874712 -1.657064

H 1.467468 2.429451 -0.537710

H 0.416919 -2.057531 -1.037615

H -1.071901 0.494961 -3.348648

H -3.608267 1.505055 0.556773

H -3.053191 3.038698 1.225334

H -3.287982 2.866396 -0.507933

H 0.148549 4.742836 0.039662

H -1.604040 4.550170 0.153193

H -0.601872 4.511092 1.604373

H 1.451315 2.805701 2.257297

H 2.728629 -0.114033 -1.231315

H 4.781467 -1.609802 -0.810599

H 4.626860 -1.265188 0.907054

H 6.368898 0.116597 0.070192

H 4.458190 1.110983 -2.116833

H 6.087358 1.723312 -1.839614

H 5.851971 0.046648 -2.344583

H -1.677142 -3.051097 2.643224

H -1.790971 -4.667010 1.884056

H -0.411673 -4.263652 2.903036

YYY-141a-c1 , delta G = 1.8612 kcal/mol, population = 2.80 %

C -0.445709 0.783502 -1.276365

C -2.355570 0.596517 0.122311

C -1.270459 1.426885 0.859488

C -1.541403 2.912663 0.644144

C -0.704960 3.750105 0.008323

C 0.722751 3.369160 -0.361663

C 1.098318 2.117240 0.398252

C 0.046831 1.018579 0.180069

C -2.684580 -0.754593 0.777707

C -2.886488 3.378598 1.154752

C -1.091064 5.165763 -0.337604

C 2.466073 1.476764 0.302646

C 2.781199 0.451806 -0.750455

C 2.242462 0.008790 0.582800

C 0.762672 -0.282959 0.658192

C 4.211874 0.090496 -1.071618

C 4.368219 -1.378702 -1.538996

C 3.860570 -2.279855 -0.430820

C 5.820343 -1.698561 -1.890107

C -3.662179 -1.551403 -0.044371

C -3.214066 -2.540381 -0.921534

C -4.110373 -3.240117 -1.723894

C -5.471237 -2.958724 -1.659742

C -5.929738 -1.974421 -0.788099

C -5.030766 -1.277084 0.011016

C 0.590140 -1.819053 2.473823

C 0.125007 -1.984464 3.889808

H 2.237426 -3.058147 0.188384

N -1.753918 0.461232 -1.194659

O 0.198224 0.833313 -2.314297

H -1.271598 1.193400 1.924039

H 1.000239 2.386819 1.459438

O 1.632827 4.443410 -0.072134

O 4.472633 -2.526832 0.585487

O 2.614376 -2.735133 -0.654934

H 3.289484 1.995792 0.775653

H 2.888705 -0.544368 1.247888

O 0.388938 -0.564422 2.036012

O 1.069853 -2.702599 1.792479

H -6.988205 -1.753061 -0.728502

H -6.170209 -3.504103 -2.281215

H -3.745330 -4.006313 -2.396512

H -2.155156 -2.764425 -0.974068

H -5.395136 -0.514548 0.690267

H -3.097172 -0.550973 1.768685

H -1.769500 -1.327250 0.917081

H -3.284209 1.158078 0.031119

H 0.819055 3.220633 -1.438721

H 0.478502 -1.148451 0.062900

H -2.278331 0.215235 -2.020220

H -2.822628 4.370326 1.601991

H -3.638181 3.429558 0.361205

H -3.267533 2.696010 1.916693

H -2.165443 5.271736 -0.478913

H -0.783953 5.875258 0.436354

H -0.592028 5.474825 -1.257101

H 1.596556 4.616022 0.877543

H 2.103959 0.394269 -1.590735

H 4.850591 0.247298 -0.197389

H 4.591609 0.737116 -1.866705

H 3.729315 -1.529924 -2.410677

H 5.926670 -2.724989 -2.245152

H 6.169961 -1.028507 -2.677053

H 6.462855 -1.572320 -1.017822

H -0.955922 -1.838630 3.933648

H 0.375850 -2.979430 4.245808

H 0.586137 -1.228706 4.526017

YYY-141a-c2 , delta G = 2.0174 kcal/mol, population = 2.15 %

C -0.450354 0.761939 -1.278655

C -2.362800 0.597265 0.119339

C -1.272924 1.419568 0.857634

C -1.540658 2.908822 0.660271

C -0.706136 3.754038 0.033774

C 0.699268 3.355615 -0.380716

C 1.088844 2.112844 0.388504

C 0.042326 1.008900 0.175460

C -2.699010 -0.752217 0.774479

C -2.879396 3.367608 1.194954

C -1.075588 5.180027 -0.277396

C 2.460541 1.477418 0.293858

C 2.783471 0.446185 -0.753474

C 2.242238 0.009674 0.579972

C 0.763226 -0.288268 0.657476

C 4.216007 0.086004 -1.068558

C 4.376958 -1.385629 -1.526649

C 3.867607 -2.281177 -0.414446

C 5.830838 -1.704639 -1.871069

C -3.676532 -1.546290 -0.050289

C -3.229581 -2.540596 -0.922013

C -4.125517 -3.236875 -1.727838

C -5.484887 -2.946559 -1.672736

C -5.942264 -1.956915 -0.806542

C -5.043694 -1.263138 -0.003919

C 0.597015 -1.820383 2.476945

C 0.133513 -1.982741 3.893694

H 2.242906 -3.056961 0.204567

N -1.762031 0.457257 -1.197401

O 0.197192 0.788775 -2.316065

H -1.272311 1.178612 1.920512

H 0.996051 2.396750 1.443525

O 1.574123 4.454532 -0.062396

O 4.477515 -2.520284 0.604967

O 2.623258 -2.739835 -0.639784

H 3.280291 1.994504 0.777040

H 2.890522 -0.533777 1.251081

O 0.391495 -0.568028 2.035342

O 1.079975 -2.704476 1.798287

H -6.999608 -1.728511 -0.753996

H -6.183597 -3.489102 -2.296983

H -3.761354 -4.007262 -2.396141

H -2.171816 -2.771560 -0.967639

H -5.407197 -0.496239 0.670845

H -3.114133 -0.546001 1.763849

H -1.785860 -1.327492 0.917761

H -3.287811 1.164808 0.027669

H 0.737919 3.177653 -1.461052

H 0.483410 -1.156577 0.064145

H -2.285384 0.200205 -2.020325

H -2.807454 4.346456 1.668103

H -3.639756 3.440078 0.411509

H -3.254158 2.667141 1.943657

H -0.650563 5.871104 0.454879

H -0.665940 5.467794 -1.247556

H -2.153119 5.329227 -0.305322

H 2.419243 4.284547 -0.493401

H 2.107303 0.381758 -1.594227

H 4.851869 0.249221 -0.193400

H 4.597430 0.727892 -1.866950

H 3.741105 -1.543516 -2.399369

H 5.939967 -2.732814 -2.220047

H 6.182229 -1.038542 -2.660612

H 6.470088 -1.572802 -0.997231

H -0.946678 -1.832643 3.939789

H 0.381889 -2.978022 4.250508

H 0.598639 -1.227656 4.527850

YYY-141a-c17-gfn0 , delta G = 2.3180 kcal/mol, population = 1.30 %

C -0.455967 1.027015 -1.092042

C -2.387829 0.450056 0.162618

C -1.364569 1.141733 1.102947

C -1.706701 2.623817 1.217953

C -0.895515 3.616809 0.820308

C 0.556765 3.388490 0.414971

C 0.976956 2.021438 0.891777

C -0.011982 0.955599 0.396750

C -2.665197 -1.025984 0.490543

C -3.084689 2.895882 1.780790

C -1.328951 5.058937 0.800301

C 2.377261 1.479818 0.702965

C 2.770508 0.729286 -0.538951

C 2.221162 -0.021668 0.643521

C 0.756743 -0.387320 0.599698

C 4.225115 0.509606 -0.880255

C 4.466545 -0.811209 -1.654169

C 3.975836 -1.956937 -0.791325

C 5.941348 -0.982752 -2.014384

C -3.582672 -1.660425 -0.520446

C -3.066114 -2.409840 -1.578680

C -3.907796 -2.950527 -2.546187

C -5.281858 -2.747767 -2.468192

C -5.808465 -2.002107 -1.416737

C -4.963931 -1.462954 -0.452855

C 0.617687 -2.295389 2.022860

C 0.129887 -2.791446 3.351281

H 2.375974 -2.918334 -0.415984

N -1.747694 0.637389 -1.129683

O 0.209171 1.333469 -2.071415

H -1.378366 0.675977 2.088122

H 0.842986 2.050595 1.980142

O 1.424285 4.339671 1.061389

O 4.573976 -2.398272 0.165681

O 2.759258 -2.402649 -1.154307

H 3.159345 1.916457 1.309565

H 2.877468 -0.679861 1.193078

O 0.363668 -0.985455 1.866476

O 1.156831 -2.983984 1.179755

H -6.877569 -1.844228 -1.345943

H -5.938355 -3.170191 -3.218398

H -3.489891 -3.532079 -3.358528

H -1.996636 -2.572123 -1.642944

H -5.381128 -0.886658 0.365333

H -3.110609 -1.066249 1.487382

H -1.726459 -1.575480 0.530673

H -3.340876 0.977207 0.164934

H 0.674552 3.479755 -0.669613

H 0.531145 -1.109763 -0.182245

H -2.237789 0.555628 -2.007281

H -3.442485 2.036587 2.351082

H -3.081999 3.755250 2.450632

H -3.823826 3.094279 0.998875

H -2.411006 5.167583 0.782152

H -0.947624 5.606546 1.666582

H -0.936780 5.557368 -0.091355

H 1.352154 5.184272 0.604380

H 2.118454 0.829144 -1.395069

H 4.832447 0.495730 0.029543

H 4.593300 1.331900 -1.499155

H 3.858625 -0.792416 -2.560288

H 6.554001 -1.025763 -1.113037

H 6.107833 -1.899360 -2.582765

H 6.277538 -0.140506 -2.621141

H 0.543814 -2.179944 4.153380

H -0.957035 -2.700355 3.393815

H 0.417092 -3.830393 3.484406

YYY-141a-c1-gfn0 , delta G = 2.7491 kcal/mol, population = 0.63 %

C 0.171904 1.207948 -1.621970

C -2.038565 0.373526 -1.366259

C -1.499391 0.811377 0.024126

C -1.996727 2.219628 0.328599

C -1.196627 3.286494 0.490024

C 0.315443 3.168792 0.629567

C 0.660512 1.725059 0.919462

C 0.030893 0.795233 -0.129918

C -2.367890 -1.124157 -1.490235

C -3.503236 2.346547 0.377975

C -1.741255 4.687223 0.610473

C 2.078547 1.255882 1.164220

C 2.969855 0.831289 0.031462

C 2.105540 -0.188377 0.722889

C 0.797215 -0.551095 0.058362

C 4.463013 0.718226 0.225453

C 5.102456 -0.366954 -0.676775

C 4.457845 -1.696096 -0.336474

C 6.617858 -0.423976 -0.490852

C -3.631385 -1.457288 -0.739648

C -4.877707 -1.198587 -1.315303

C -6.052432 -1.430131 -0.608982

C -5.999368 -1.925915 0.690834

C -4.764686 -2.189297 1.274450

C -3.592062 -1.954812 0.563025

C 0.406264 -2.730456 0.935457

C -0.393185 -3.550595 1.905286

H 2.944001 -2.746830 -0.808864

N -0.958309 0.801817 -2.240146

O 1.111892 1.756185 -2.178091

H -1.838211 0.124256 0.797298

H 0.131726 1.486282 1.853073

O 0.804881 4.035526 1.666794

O 4.713812 -2.346685 0.653384

O 3.508439 -2.060081 -1.217604

H 2.539074 1.540737 2.101235

H 2.582205 -0.950853 1.320686

O 0.043929 -1.435103 0.940901

O 1.269390 -3.186120 0.213084

H -4.712992 -2.576806 2.284448

H -6.913150 -2.108030 1.242201

H -7.009390 -1.226490 -1.073214

H -4.927147 -0.814205 -2.327811

H -2.635505 -2.153579 1.025518

H -1.530253 -1.718951 -1.127191

H -2.493474 -1.356585 -2.550239

H -2.939501 0.929295 -1.623006

H 0.819731 3.520416 -0.272385

H 0.938094 -1.078602 -0.882556

H -1.084831 0.966472 -3.227520

H -3.969245 1.372014 0.531189

H -3.819456 3.000557 1.190757

H -3.916600 2.759807 -0.547014

H -1.024977 5.404073 0.206741

H -2.682349 4.802804 0.074638

H -1.914933 4.972820 1.652325

H 0.394942 3.764832 2.498440

H 2.668360 1.130683 -0.961750

H 4.696271 0.485521 1.268618

H 4.942960 1.672336 -0.006495

H 4.860492 -0.134240 -1.715186

H 6.868437 -0.685960 0.537696

H 7.071461 -1.165469 -1.150713

H 7.058418 0.548036 -0.717888

H -1.145537 -4.115129 1.350384

H 0.267262 -4.266281 2.391591

H -0.888850 -2.928567 2.646311

YYY-141a-c5-gfn0 , delta G = 2.9167 kcal/mol, population = 0.47 %

C 0.171741 1.194531 -1.635408

C -2.045666 0.380040 -1.372923

C -1.500263 0.808945 0.017387

C -2.000595 2.212595 0.341910

C -1.205656 3.280327 0.517724

C 0.305986 3.163542 0.602209

C 0.656463 1.723676 0.905539

C 0.029741 0.790144 -0.141299

C -2.379546 -1.116247 -1.502288

C -3.508664 2.322721 0.410075

C -1.743675 4.676005 0.685522

C 2.076234 1.257526 1.153480

C 2.973149 0.827556 0.024677

C 2.105160 -0.187831 0.715975

C 0.797181 -0.554880 0.051893

C 4.465617 0.712501 0.224326

C 5.106619 -0.378084 -0.670115

C 4.459151 -1.704664 -0.324494

C 6.621340 -0.435825 -0.478783

C -3.640707 -1.451258 -0.748769

C -3.596997 -1.955785 0.551035

C -4.767157 -2.191595 1.265998

C -6.003633 -1.922989 0.688639

C -6.061049 -1.420697 -0.608502

C -4.888753 -1.187610 -1.318313

C 0.408145 -2.731070 0.937785

C -0.394045 -3.547199 1.908555

H 2.943991 -2.754622 -0.795789

N -0.965981 0.805551 -2.248820

O 1.119967 1.723645 -2.197764

H -1.835645 0.115643 0.786523

H 0.131408 1.499260 1.841536

O 0.751608 4.034141 1.659348

O 4.711311 -2.349327 0.670106

O 3.512386 -2.072044 -1.206658

H 2.530569 1.534421 2.096741

H 2.579644 -0.945752 1.321281

O 0.044793 -1.436206 0.936312

O 1.274201 -3.189188 0.220224

H -7.019513 -1.212864 -1.067761

H -6.915518 -2.105803 1.242913

H -4.712065 -2.583723 2.274018

H -2.638885 -2.158434 1.008664

H -4.941401 -0.797690 -2.328550

H -1.541452 -1.713703 -1.144277

H -2.509009 -1.344448 -2.562796

H -2.944776 0.941092 -1.624860

H 0.765141 3.489789 -0.337622

H 0.940563 -1.085211 -0.887185

H -1.090838 0.962206 -3.237723

H -3.936845 2.759136 -0.497210

H -3.961758 1.338578 0.537711

H -3.824311 2.944773 1.247538

H -2.758455 4.773565 0.305038

H -1.737981 4.982311 1.734561

H -1.108886 5.387186 0.152675

H 1.711087 4.098402 1.594299

H 2.674013 1.123926 -0.970268

H 4.694670 0.484239 1.269419

H 4.949133 1.664287 -0.010824

H 4.868364 -0.150707 -1.710563

H 6.868139 -0.692233 0.552077

H 7.076037 -1.181823 -1.132726

H 7.064191 0.534153 -0.710208

H 0.265684 -4.258340 2.402499

H -0.894666 -2.921685 2.643272

H -1.142231 -4.116951 1.353334

YYY-141a-c15-gfn0 , delta G = 3.1608 kcal/mol, population = 0.31 %

C 0.171326 1.194641 -1.640296

C -2.039020 0.362056 -1.376581

C -1.500310 0.813144 0.009446

C -2.002887 2.221184 0.306205

C -1.203576 3.287506 0.466554

C 0.312004 3.172004 0.593114

C 0.658252 1.736330 0.898424

C 0.029817 0.798567 -0.143838

C -2.366586 -1.137118 -1.487404

C -3.511590 2.333604 0.362831

C -1.738950 4.688165 0.601633

C 2.077099 1.271381 1.146130

C 2.969522 0.835220 0.018028

C 2.104199 -0.176856 0.719193

C 0.796398 -0.545975 0.057542

C 4.462398 0.722353 0.214426

C 5.102076 -0.372645 -0.675887

C 4.455916 -1.697754 -0.322852

C 6.617187 -0.429091 -0.487543

C -3.630495 -1.465464 -0.735421

C -3.592333 -1.944043 0.574398

C -4.765734 -2.173383 1.286137

C -6.000073 -1.924240 0.695582

C -6.052011 -1.447631 -0.611411

C -4.876455 -1.220873 -1.317952

C 0.404988 -2.716520 0.955861

C -0.394405 -3.526341 1.934371

H 2.938326 -2.748739 -0.783624

N -0.958945 0.782924 -2.254379

O 1.111543 1.736046 -2.203643

H -1.836562 0.131920 0.788833

H 0.132769 1.513929 1.835208

O 0.795245 3.959655 1.698036

O 4.712108 -2.339519 0.672637

O 3.505499 -2.068625 -1.200001

H 2.535282 1.565479 2.081027

H 2.579915 -0.933278 1.325396

O 0.042883 -1.421649 0.947896

O 1.267906 -3.180263 0.237957

H -7.008677 -1.255095 -1.080979

H -6.914430 -2.102274 1.247332

H -4.714834 -2.545380 2.301968

H -2.636230 -2.131394 1.042507

H -4.924875 -0.851204 -2.335980

H -1.528457 -1.727349 -1.118044

H -2.490728 -1.379504 -2.545368

H -2.940378 0.914814 -1.638687

H 0.805831 3.506932 -0.324829

H 0.937367 -1.082451 -0.878317

H -1.084170 0.934265 -3.244029

H -3.965445 1.352414 0.509360

H -3.832481 2.973111 1.184979

H -3.934468 2.749355 -0.556475

H -1.717860 5.028723 1.640501

H -1.124039 5.384912 0.023222

H -2.760154 4.778910 0.238541

H 0.806846 4.886159 1.435743

H 2.669080 1.123452 -0.978832

H 4.694163 0.500060 1.260180

H 4.944379 1.673345 -0.026576

H 4.861513 -0.150444 -1.716958

H 6.866183 -0.680597 0.543991

H 7.071023 -1.177866 -1.138991

H 7.059061 0.540089 -0.724077

H -1.147196 -4.096307 1.385628

H 0.266012 -4.237089 2.427901

H -0.889422 -2.896291 2.669006

### Table S8. Geometry data of conformers of structure 1b.

YYY-141b-c3 , delta G = 0.0000 kcal/mol, population = 34.24 %

C -0.120467 0.441173 -1.007353

C -2.075603 0.999905 0.219207

C -0.853755 0.979977 1.173296

C -0.291840 2.398565 1.297046

C 0.953938 2.757130 0.940506

C 2.026786 1.742539 0.568244

C 1.558336 0.397221 1.083255

C 0.181431 0.071345 0.475911

C -3.164462 -0.045166 0.509225

C -1.285018 3.412303 1.821631

C 1.412406 4.190490 0.927816

C 2.408810 -0.859873 1.160162

C 2.659018 -1.846909 0.050949

C 1.447972 -2.016810 0.935979

C 0.056257 -1.474166 0.648661

C 2.772910 -1.576110 -1.426571

C 4.247155 -1.386625 -1.852810

C 4.902199 -0.360225 -0.958693

C 4.344602 -0.955365 -3.318498

C -4.307962 0.066218 -0.464111

C -4.376322 -0.761936 -1.585377

C -5.401469 -0.617391 -2.515831

C -6.375147 0.359661 -2.336149

C -6.318437 1.190037 -1.219813

C -5.291990 1.043268 -0.293919

C -1.414917 -2.901726 1.901298

C -2.186025 -3.003110 3.186190

H 6.170162 -0.185321 0.434456

N -1.422971 0.797324 -1.063078

O 0.616938 0.408708 -1.981071

H -1.137948 0.598511 2.153135

H 1.347010 0.580104 2.142989

O 3.266755 2.134115 1.171988

O 4.654108 0.828294 -0.957132

O 5.808431 -0.897318 -0.119265

H 3.095635 -0.882859 1.996004

H 1.451749 -2.887128 1.578648

O -0.747246 -1.731875 1.834394

O -1.391382 -3.737279 1.027432

H -7.076352 1.948896 -1.069247

H -7.174782 0.471073 -3.057587

H -5.440388 -1.270118 -3.379120

H -3.621734 -1.526326 -1.727979

H -5.255362 1.690633 0.575162

H -3.519506 0.113824 1.530217

H -2.736032 -1.043823 0.459372

H -2.551192 1.979630 0.222642

H 2.162715 1.722339 -0.516027

H -0.435173 -1.953459 -0.195509

H -1.838167 1.066401 -1.941917

H -2.077526 2.916962 2.386053

H -0.808463 4.131262 2.487229

H -1.766835 3.981436 1.020693

H 2.096176 4.353634 0.092740

H 0.582490 4.888106 0.831254

H 1.968724 4.439489 1.835237

H 3.960653 1.807851 0.579645

H 3.410671 -2.567141 0.366585

H 2.199931 -0.703857 -1.717870

H 2.381616 -2.425294 -1.994473

H 4.782228 -2.326820 -1.708314

H 3.842558 -1.689770 -3.949795

H 3.861806 0.011000 -3.464250

H 5.382969 -0.877537 -3.645361

H -2.715612 -3.950865 3.223519

H -1.504717 -2.922732 4.034226

H -2.896348 -2.177997 3.255945

YYY-141b-c1 , delta G = 0.0113 kcal/mol, population = 33.59 %

C 0.013032 0.402904 -0.912684

C -1.984391 0.987119 0.227595

C -0.803951 0.950165 1.233307

C -0.233100 2.362352 1.385797

C 1.028803 2.711941 1.081503

C 2.103380 1.688908 0.744045

C 1.603766 0.343949 1.225147

C 0.246396 0.028547 0.577870

C -3.101596 -0.037398 0.477065

C -1.237442 3.381077 1.879576

C 1.504896 4.139117 1.097463

C 2.443539 -0.914602 1.308851

C 2.698502 -1.934291 0.232673

C 1.477287 -2.070542 1.110990

C 0.102263 -1.518236 0.755949

C 2.789773 -1.799906 -1.264753

C 4.212405 -1.402823 -1.740120

C 4.499322 0.021722 -1.330564

C 4.374561 -1.641769 -3.241783

C -4.205336 0.092072 -0.539195

C -4.245815 -0.735751 -1.662060

C -5.232932 -0.574458 -2.630218

C -6.195784 0.419362 -2.487307

C -6.166486 1.249709 -1.369903

C -5.177919 1.086234 -0.406366

C -1.441909 -2.928900 1.933677

C -2.288001 -3.016333 3.171479

H 4.365675 1.812992 -1.917096

N -1.282223 0.770872 -1.027025

O 0.800589 0.373166 -1.846948

H -1.134169 0.571479 2.199581

H 1.364815 0.518480 2.280609

O 3.316048 2.069646 1.405156

O 4.950230 0.352499 -0.250576

O 4.180401 0.928048 -2.270658

H 3.137119 -0.927857 2.139030

H 1.451472 -2.917075 1.784150

O -0.764932 -1.762639 1.897046

O -1.372102 -3.770819 1.068586

H -6.916120 2.021832 -1.248059

H -6.965759 0.543912 -3.238248

H -5.250816 -1.227179 -3.494199

H -3.499619 -1.513113 -1.775802

H -5.162493 1.733871 0.463074

H -3.492258 0.132049 1.483238

H -2.690232 -1.044060 0.447371

H -2.443291 1.974528 0.208718

H 2.273611 1.679938 -0.337564

H -0.348356 -1.997877 -0.110811

H -1.652248 1.055891 -1.920909

H -2.060485 2.887607 2.400011

H -0.783352 4.084105 2.577045

H -1.675318 3.967387 1.066125

H 2.178938 4.314745 0.256246

H 0.684101 4.850134 1.029466

H 2.078657 4.355561 2.001848

H 4.024724 1.547408 0.996481

H 3.458690 -2.627658 0.585963

H 2.070341 -1.094395 -1.664697

H 2.577120 -2.777288 -1.707780

H 4.937194 -2.008931 -1.191578

H 5.357210 -1.326749 -3.595895

H 4.262809 -2.706343 -3.452454

H 3.617797 -1.099260 -3.808183

H -2.815318 -3.965918 3.189917

H -1.659947 -2.920460 4.058064

H -3.004981 -2.194054 3.185816

YYY-141b-c10-gfn0 , delta G = 0.7066 kcal/mol, population = 10.37 %

C -0.187898 0.124276 0.992132

C -2.070078 -0.822534 -0.102192

C -0.767560 -1.355602 -0.755293

C -0.391656 -2.686383 -0.100323

C 0.748346 -2.904582 0.578597

C 1.908003 -1.918177 0.572033

C 1.683254 -0.959727 -0.577457

C 0.303138 -0.294471 -0.423154

C -2.972844 0.021006 -1.015610

C -1.451706 -3.758560 -0.213140

C 0.993260 -4.167359 1.364062

C 2.691220 0.052386 -1.087345

C 2.951254 1.435917 -0.564894

C 1.878602 1.224935 -1.611407

C 0.403914 0.977079 -1.324696

C 2.866870 1.997579 0.831778

C 4.141039 1.710730 1.673501

C 4.211216 0.222036 1.934631

C 4.116619 2.498813 2.980042

C -4.201509 0.498014 -0.287053

C -4.249304 1.776050 0.271663

C -5.361162 2.194879 0.996737

C -6.443313 1.338635 1.172489

C -6.407655 0.062150 0.617450

C -5.294488 -0.352725 -0.104622

C -0.733547 1.699469 -3.313281

C -1.357430 1.208088 -4.588047

H 4.545740 -1.381750 0.966411

N -1.525701 -0.068912 1.014398

O 0.455649 0.571091 1.927599

H -0.899756 -1.484415 -1.828726

H 1.560886 -1.611652 -1.452810

O 3.173546 -2.611297 0.461296

O 3.698578 -0.338426 2.878642

O 4.851359 -0.447566 0.957491

H 3.477424 -0.374126 -1.695711

H 2.065001 1.656702 -2.585758

O -0.227326 0.671922 -2.599840

O -0.694521 2.851035 -2.947545

H -7.248679 -0.608242 0.744690

H -7.309767 1.664114 1.734268

H -5.382160 3.190632 1.421950

H -3.409801 2.447311 0.135169

H -5.274653 -1.346848 -0.537077

H -3.255225 -0.597063 -1.871192

H -2.417339 0.876005 -1.394422

H -2.671635 -1.643394 0.285225

H 1.981177 -1.399803 1.526732

H -0.110844 1.834581 -0.896320

H -2.057514 0.152199 1.842257

H -1.009139 -4.742168 -0.368029

H -2.076143 -3.822509 0.683085

H -2.115624 -3.557421 -1.055861

H 1.516694 -4.923301 0.770983

H 1.627271 -3.952810 2.225162

H 0.065337 -4.609377 1.722900

H 3.205561 -3.068633 -0.389102

H 3.835904 1.827766 -1.062343

H 2.002172 1.631064 1.373083

H 2.774875 3.085229 0.756207

H 5.017730 1.986836 1.082016

H 5.012250 2.311129 3.574753

H 4.062584 3.568390 2.770857

H 3.249203 2.217286 3.577317

H -2.196696 0.551055 -4.353509

H -1.706527 2.052433 -5.175659

H -0.633555 0.626388 -5.159551

YYY-141b-c9-gfn0 , delta G = 1.0316 kcal/mol, population = 5.99 %

C -0.265381 0.090570 1.052787

C -2.120919 -0.853616 -0.089348

C -0.804376 -1.353334 -0.740202

C -0.420884 -2.699659 -0.123453

C 0.711402 -2.922640 0.566786

C 1.847680 -1.907966 0.623849

C 1.640346 -0.933756 -0.516873

C 0.249496 -0.289550 -0.366519

C -3.024355 0.002180 -0.991086

C -1.464023 -3.780927 -0.297099

C 0.969491 -4.206943 1.310391

C 2.645296 0.098632 -1.000741

C 2.877203 1.478553 -0.446093

C 1.815723 1.273525 -1.499118

C 0.342817 1.002160 -1.236636

C 2.799931 1.952297 0.983337

C 4.182252 1.843386 1.674385

C 4.739047 0.458273 1.426338

C 4.085020 2.187444 3.162016

C -4.272478 0.435238 -0.268419

C -4.353375 1.695323 0.326055

C -5.484699 2.072227 1.044052

C -6.553299 1.191580 1.176521

C -6.484574 -0.067318 0.585463

C -5.352134 -0.440337 -0.129310

C -0.777399 1.757713 -3.222749

C -1.364262 1.290749 -4.523860

H 4.238441 -1.358843 1.684312

N -1.600083 -0.120418 1.052224

O 0.358773 0.522188 2.009467

H -0.922726 -1.451310 -1.818406

H 1.538941 -1.571691 -1.404730

O 3.136098 -2.553597 0.573541

O 5.635022 0.201658 0.651064

O 4.087126 -0.493402 2.116736

H 3.434242 -0.305625 -1.620953

H 2.007366 1.731877 -2.460213

O -0.268983 0.719577 -2.526222

O -0.763163 2.899760 -2.826687

H -7.315138 -0.756127 0.678351

H -7.435133 1.484874 1.732044

H -5.531696 3.054802 1.496946

H -3.524804 2.385830 0.222640

H -5.306286 -1.420546 -0.590592

H -3.282496 -0.593194 -1.870092

H -2.477069 0.877644 -1.333560

H -2.715232 -1.692032 0.271073

H 1.854238 -1.394102 1.583468

H -0.186589 1.843779 -0.794983

H -2.147082 0.078713 1.875815

H -2.107294 -3.884784 0.581838

H -2.112234 -3.557187 -1.146370

H -1.006388 -4.752787 -0.478928

H 0.046317 -4.719072 1.574486

H 1.584845 -4.899131 0.728448

H 1.520722 -4.000537 2.229381

H 3.262221 -2.951886 -0.296735

H 3.750503 1.907247 -0.932443

H 2.066830 1.392655 1.551801

H 2.503265 3.005198 1.008146

H 4.878052 2.527215 1.184286

H 3.408798 1.503343 3.674375

H 5.061015 2.138101 3.648358

H 3.698919 3.201561 3.279716

H -1.753920 2.140433 -5.077244

H -0.601979 0.779136 -5.112735

H -2.165546 0.575913 -4.330340

YYY-141b-c25-gfn0 , delta G = 1.2726 kcal/mol, population = 3.99 %

C -0.037441 -0.838699 1.243996

C -1.957355 -1.121551 -0.115633

C -0.712050 -1.702521 -0.842596

C -0.604472 -3.195120 -0.535315

C 0.429899 -3.760981 0.108979

C 1.733201 -3.023662 0.407487

C 1.756631 -1.765656 -0.422308

C 0.478909 -0.953797 -0.216140

C -2.629461 0.076052 -0.804858

C -1.804310 -4.005321 -0.976009

C 0.423861 -5.205610 0.534837

C 2.935902 -0.820116 -0.506016

C 3.247417 0.444589 0.270187

C 2.362445 0.515553 -0.944823

C 0.844168 0.456343 -0.788786

C 2.893125 0.967546 1.642936

C 3.266927 2.470667 1.790008

C 2.684495 3.231177 0.617223

C 4.765976 2.715382 1.930089

C -3.796410 0.592177 -0.005526

C -3.633131 1.656763 0.882375

C -4.695691 2.101945 1.662865

C -5.939656 1.486656 1.565229

C -6.114302 0.424353 0.682350

C -5.049477 -0.018311 -0.094668

C 0.011835 1.838740 -2.560159

C -0.532446 1.817133 -3.957193

H 0.997654 3.487507 -0.233802

N -1.386884 -0.782168 1.179836

O 0.612781 -0.815630 2.277092

H -0.783396 -1.541607 -1.917574

H 1.687671 -2.131957 -1.454779

O 2.866300 -3.815077 -0.000645

O 3.318250 3.726431 -0.286762

O 1.336138 3.259570 0.655772

H 3.784649 -1.227249 -1.038974

H 2.711549 1.045797 -1.820393

O 0.262997 0.597465 -2.115173

O 0.206309 2.846080 -1.909992

H -7.081046 -0.056178 0.596134

H -6.768309 1.834564 2.169036

H -4.552422 2.931586 2.344023

H -2.666506 2.139970 0.960139

H -5.192981 -0.843366 -0.783485

H -2.959141 -0.244928 -1.795766

H -1.902884 0.873503 -0.941470

H -2.715985 -1.890507 0.024192

H 1.817198 -2.800009 1.474244

H 0.444961 1.252651 -0.168323

H -1.936959 -0.725325 2.023092

H -2.359188 -3.477331 -1.753592

H -1.506836 -4.970996 -1.383309

H -2.502204 -4.198461 -0.155760

H 0.875779 -5.307119 1.526294

H -0.580810 -5.618243 0.592620

H 1.007433 -5.829084 -0.148144

H 3.034026 -4.483103 0.672305

H 4.257322 0.741242 0.003534

H 3.434810 0.409511 2.410319

H 1.842860 0.844732 1.864197

H 2.757900 2.831975 2.687544

H 4.980544 3.777055 2.058335

H 5.147041 2.180813 2.801584

H 5.314161 2.375992 1.051184

H 0.276457 1.570098 -4.648044

H -1.298872 1.049283 -4.056329

H -0.934749 2.794026 -4.210142

YYY-141b-c2 , delta G = 1.4841 kcal/mol, population = 2.79 %

C 0.456106 0.163813 -1.571529

C -1.830600 0.708911 -1.238231

C -1.068839 0.803331 0.112462

C -0.649968 2.256941 0.342443

C 0.622182 2.676764 0.450808

C 1.791336 1.711416 0.576762

C 1.217970 0.367065 0.970676

C 0.179645 -0.089338 -0.065084

C -2.979912 -0.310986 -1.285707

C -1.812880 3.224536 0.401053

C 0.995617 4.132928 0.516598

C 2.041242 -0.815818 1.437739

C 2.702482 -1.877793 0.601645

C 1.263181 -2.051361 1.022736

C 0.068343 -1.632329 0.172722

C 3.294543 -1.833463 -0.782699

C 4.773627 -1.366393 -0.782113

C 4.837372 0.093115 -0.400624

C 5.445185 -1.670185 -2.121809

C -4.175815 0.191140 -0.518146

C -4.333737 -0.080314 0.841896

C -5.401968 0.450853 1.557576

C -6.332262 1.266623 0.922019

C -6.187015 1.544466 -0.434086

C -5.117318 1.011031 -1.144566

C -1.583030 -3.183229 0.935810

C -2.796018 -3.353976 1.805808

H 4.842871 1.840215 -1.119768

N -0.742991 0.396927 -2.152723

O 1.517849 0.147682 -2.175810

H -1.696256 0.466382 0.934370

H 0.626136 0.589838 1.866017

O 2.686239 2.213900 1.576507

O 4.864249 0.506342 0.742750

O 4.829555 0.928776 -1.453974

H 2.407456 -0.725418 2.451691

H 1.053414 -2.854449 1.715673

O -1.131723 -1.908929 0.950940

O -1.075107 -4.061671 0.278942

H -6.908310 2.174957 -0.939139

H -7.164758 1.680161 1.477111

H -5.506794 0.226949 2.612005

H -3.611623 -0.706951 1.346299

H -5.011496 1.231329 -2.200865

H -2.638016 -1.268841 -0.898624

H -3.252261 -0.461469 -2.332736

H -2.247073 1.678139 -1.509798

H 2.322585 1.645139 -0.378508

H -0.014499 -2.186504 -0.760372

H -0.782913 0.621467 -3.135815

H -1.665670 3.974455 1.177844

H -1.963107 3.759041 -0.541787

H -2.741573 2.696188 0.621888

H 1.215187 4.441703 1.541620

H 1.906650 4.309376 -0.059548

H 0.213133 4.778189 0.122052

H 3.513966 1.716341 1.479910

H 3.329150 -2.487098 1.249211

H 2.724883 -1.208506 -1.460726

H 3.290706 -2.850213 -1.186683

H 5.297043 -1.893603 0.018986

H 6.474805 -1.310489 -2.146559

H 5.457758 -2.749102 -2.283478

H 4.902380 -1.207106 -2.945756

H -2.854964 -4.387021 2.140136

H -2.771989 -2.678285 2.658329

H -3.688329 -3.132388 1.216360

YYY-141b-c4 , delta G = 1.5449 kcal/mol, population = 2.52 %

C 0.320947 0.154388 -1.720848

C -1.950256 0.694283 -1.276722

C -1.122241 0.809916 0.031955

C -0.700779 2.268201 0.221998

C 0.573746 2.693108 0.261047

C 1.753308 1.733964 0.348585

C 1.200048 0.397650 0.800476

C 0.124013 -0.075719 -0.195303

C -3.097730 -0.328429 -1.251044

C -1.862318 3.233489 0.323558

C 0.941074 4.152294 0.284683

C 2.035421 -0.779923 1.272666

C 2.703006 -1.810945 0.401953

C 1.273212 -2.020307 0.840453

C 0.035245 -1.614970 0.052803

C 3.301518 -1.620795 -0.967920

C 4.823700 -1.359607 -0.887171

C 5.087977 -0.239991 0.091366

C 5.393465 -1.008725 -2.264113

C -4.256412 0.187389 -0.437059

C -4.364076 -0.083324 0.927841

C -5.397097 0.460117 1.685039

C -6.341100 1.288087 1.086561

C -6.245458 1.565657 -0.274105

C -5.211412 1.019449 -1.026108

C -1.565317 -3.168328 0.918478

C -2.725567 -3.338192 1.858191

H 5.779269 0.103743 1.817628

N -0.909736 0.372000 -2.239446

O 1.342612 0.120870 -2.389153

H -1.705525 0.477699 0.887846

H 0.635967 0.641249 1.707905

O 2.703369 2.252542 1.288222

O 4.809645 0.928066 -0.089761

O 5.664159 -0.663468 1.232247

H 2.394214 -0.692853 2.289789

H 1.109665 -2.846200 1.518819

O -1.117585 -1.893506 0.900882

O -1.094046 -4.049766 0.238447

H -6.977902 2.205828 -0.750104

H -7.145833 1.711803 1.674010

H -5.463463 0.236891 2.742732

H -3.629644 -0.718728 1.402937

H -5.144229 1.239285 -2.085657

H -2.736475 -1.277855 -0.861201

H -3.419206 -0.499697 -2.280913

H -2.382872 1.658654 -1.540326

H 2.241934 1.646975 -0.625245

H -0.095830 -2.176772 -0.869882

H -1.003642 0.570892 -3.224316

H -1.676395 3.999533 1.076095

H -2.065183 3.749036 -0.620119

H -2.776201 2.707390 0.604016

H 1.160679 4.492514 1.300018

H 1.849389 4.316351 -0.298411

H 0.152774 4.781490 -0.124948

H 3.569767 1.937402 0.989172

H 3.338616 -2.449404 1.011473

H 2.827447 -0.805443 -1.501101

H 3.161830 -2.524301 -1.568483

H 5.317986 -2.251223 -0.497732

H 5.173659 -1.813521 -2.966894

H 4.940752 -0.090873 -2.639289

H 6.475766 -0.873058 -2.227927

H -3.653769 -3.172114 1.306992

H -2.733398 -4.357756 2.236791

H -2.680602 -2.627191 2.680388

YYY-141b-c3-gfn0 , delta G = 1.6911 kcal/mol, population = 1.97 %

C 0.038383 -0.786497 1.230530

C -1.894897 -1.057282 -0.111425

C -0.665220 -1.672165 -0.835917

C -0.575994 -3.162650 -0.508004

C 0.460979 -3.743435 0.119959

C 1.768804 -3.019496 0.396269

C 1.801467 -1.775814 -0.462491

C 0.544863 -0.931676 -0.233142

C -2.552226 0.136600 -0.820785

C -1.797663 -3.957421 -0.912740

C 0.438182 -5.182242 0.563545

C 3.003878 -0.862123 -0.614014

C 3.387094 0.380392 0.167070

C 2.439072 0.495244 -0.996164

C 0.922805 0.463087 -0.827763

C 3.159356 0.799435 1.599728

C 3.200157 2.341329 1.804434

C 2.030567 2.931578 1.046696

C 4.550904 2.970191 1.467368

C -3.697412 0.694014 -0.017880

C -4.965119 0.111143 -0.081597

C -6.008973 0.589914 0.702581

C -5.797998 1.661040 1.566668

C -4.539176 2.249414 1.637933

C -3.497337 1.768639 0.850560

C 0.114241 1.808176 -2.642511

C -0.556012 1.755914 -3.983180

H 1.547125 3.535928 -0.686673

N -1.308070 -0.701989 1.171196

O 0.687500 -0.767546 2.264935

H -0.742829 -1.527909 -1.912727

H 1.688433 -2.162517 -1.482795

O 2.839596 -3.917264 0.042663

O 0.880767 2.862355 1.431585

O 2.358080 3.463949 -0.141176

H 3.801603 -1.283771 -1.212651

H 2.780265 1.037054 -1.867352

O 0.339371 0.577775 -2.159637

O 0.390121 2.832715 -2.050523

H -4.367142 3.084967 2.305023

H -6.610008 2.036088 2.176879

H -6.987456 0.130234 0.637116

H -5.136513 -0.721204 -0.755248

H -2.518721 2.229946 0.909575

H -2.901480 -0.202898 -1.798798

H -1.812956 0.916778 -0.985700

H -2.665523 -1.809961 0.049851

H 1.848053 -2.774384 1.459425

H 0.522523 1.275577 -0.229177

H -1.849913 -0.604428 2.016070

H -2.349252 -3.438742 -1.699102

H -1.526858 -4.940938 -1.295132

H -2.490422 -4.111067 -0.079855

H -0.574868 -5.555403 0.700287

H 0.950449 -5.829370 -0.152928

H 0.970763 -5.286984 1.510706

H 3.653887 -3.550419 0.404822

H 4.377636 0.680494 -0.161830

H 3.955612 0.370486 2.215686

H 2.225507 0.416424 1.989713

H 2.978268 2.512346 2.859704

H 4.801931 2.854031 0.414240

H 4.555443 4.037812 1.692475

H 5.333597 2.496091 2.062857

H -0.116080 0.977286 -4.604428

H -1.610483 1.510858 -3.836492

H -0.481346 2.724206 -4.470306

YYY-141b-c13-gfn0 , delta G = 1.7231 kcal/mol, population = 1.86 %

C 0.462409 -0.636888 1.735963

C -1.850429 -0.572559 1.219095

C -1.062787 -1.168120 0.016197

C -1.089889 -2.692258 0.114787

C -0.001768 -3.466795 0.261581

C 1.412160 -2.925898 0.131231

C 1.329656 -1.581506 -0.557527

C 0.377532 -0.645264 0.188090

C -2.659082 0.700309 0.921369

C -2.482707 -3.283600 0.079537

C -0.092586 -4.948438 0.513827

C 2.528974 -0.795030 -1.044238

C 3.338960 0.287044 -0.360978

C 2.078118 0.653617 -1.099344

C 0.745681 0.775467 -0.359838

C 3.649915 0.615918 1.079995

C 4.332969 2.007509 1.210170

C 3.512922 3.017219 0.435086

C 5.793434 2.012082 0.770095

C -3.896577 0.371494 0.126612

C -5.056528 -0.048453 0.781844

C -6.184176 -0.431492 0.064597

C -6.169924 -0.401172 -1.327014

C -5.021850 0.017112 -1.991608

C -3.895792 0.398340 -1.268661

C -0.345027 2.531398 -1.562934

C -1.361267 2.848277 -2.620403

H 1.712245 3.629277 0.337658

N -0.784818 -0.371765 2.190798

O 1.437309 -0.821982 2.446617

H -1.502687 -0.851827 -0.927202

H 0.813279 -1.804347 -1.499271

O 2.160537 -3.864963 -0.665274

O 3.847067 3.550829 -0.598425

O 2.308130 3.227798 1.002415

H 3.007385 -1.221937 -1.916797

H 2.158889 1.252761 -1.995877

O -0.252300 1.210579 -1.328404

O 0.300407 3.372108 -0.971034

H -5.001164 0.046600 -3.074005

H -7.047084 -0.698419 -1.887713

H -7.074653 -0.751558 0.591301

H -5.075975 -0.073770 1.865601

H -3.005051 0.713565 -1.794264

H -2.036269 1.423445 0.398365

H -2.942860 1.148841 1.875977

H -2.546489 -1.310032 1.617061

H 1.881870 -2.843792 1.116101

H 0.767199 1.510902 0.438598

H -0.960457 -0.372396 3.184730

H -2.845450 -3.558292 1.074583

H -3.194538 -2.569090 -0.336132

H -2.515734 -4.182884 -0.535336

H 0.707714 -5.262607 1.186520

H -1.044378 -5.230422 0.960291

H 0.035505 -5.519798 -0.409212

H 3.092312 -3.631892 -0.586177

H 4.199132 0.493828 -0.990722

H 4.329385 -0.131901 1.496603

H 2.763244 0.597276 1.696815

H 4.273214 2.288870 2.264800

H 6.237398 3.000889 0.891153

H 6.363210 1.304000 1.373866

H 5.899213 1.733535 -0.278456

H -2.310588 3.084488 -2.134441

H -1.038125 3.727437 -3.173495

H -1.513298 2.008283 -3.293923

YYY-141b-c6-gfn0 , delta G = 1.9158 kcal/mol, population = 1.34 %

C 0.468683 -0.651872 1.727598

C -1.843918 -0.567617 1.215338

C -1.061179 -1.170270 0.012099

C -1.088858 -2.692758 0.125071

C 0.001640 -3.462179 0.285791

C 1.421039 -2.933583 0.111922

C 1.334357 -1.585606 -0.568678

C 0.381114 -0.651835 0.179616

C -2.649684 0.706530 0.916242

C -2.479795 -3.288238 0.104418

C -0.092632 -4.938568 0.574806

C 2.532110 -0.796877 -1.051508

C 3.338156 0.280713 -0.360592

C 2.080857 0.651900 -1.103720

C 0.748013 0.770293 -0.365214

C 3.643690 0.605120 1.082401

C 4.328863 1.995062 1.218914

C 3.511932 3.008777 0.446020

C 5.790302 1.999104 0.782141

C -3.888977 0.377615 0.124253

C -5.047664 -0.041656 0.782177

C -6.176898 -0.424951 0.067573

C -6.165621 -0.395540 -1.324094

C -5.018842 0.022032 -1.991373

C -3.891099 0.403323 -1.271031

C -0.343797 2.526868 -1.566021

C -1.357093 2.845886 -2.625826

H 1.712745 3.624336 0.346321

N -0.775190 -0.370005 2.184624

O 1.440630 -0.856683 2.435491

H -1.503138 -0.858095 -0.931738

H 0.810940 -1.796766 -1.512546

O 2.216951 -3.852115 -0.655847

O 3.848931 3.547473 -0.583954

O 2.305203 3.217734 1.010665

H 3.019905 -1.226154 -1.916859

H 2.164136 1.257800 -1.995530

O -0.249896 1.205365 -1.334808

O 0.298478 3.366739 -0.969792

H -5.000742 0.051463 -3.073834

H -7.044192 -0.692537 -1.882707

H -7.066383 -0.744249 0.596395

H -5.064936 -0.065982 1.865982

H -3.001497 0.718530 -1.798572

H -2.026718 1.427601 0.390914

H -2.931003 1.157151 1.870548

H -2.542232 -1.301213 1.616272

H 1.935763 -2.866732 1.070810

H 0.766351 1.504308 0.434543

H -0.949456 -0.378826 3.178787

H -2.510396 -4.202491 -0.488380

H -2.839554 -3.541472 1.106223

H -3.194801 -2.585686 -0.326102

H 0.747118 -5.251758 1.197130

H -1.016323 -5.193995 1.091619

H -0.043768 -5.538809 -0.338417

H 1.800962 -3.956196 -1.521445

H 4.201706 0.487988 -0.985400

H 4.318737 -0.146248 1.499172

H 2.754854 0.588218 1.695974

H 4.267241 2.272706 2.274458

H 6.235620 2.986662 0.908670

H 6.357291 1.287123 1.383835

H 5.898025 1.724821 -0.267288

H -1.513846 2.004769 -3.296807

H -2.305174 3.090501 -2.141663

H -1.027708 3.721008 -3.181740

YYY-141b-c16-gfn0 , delta G = 2.3525 kcal/mol, population = 0.64 %

C 0.285338 0.569552 1.547268

C -2.013656 0.119775 1.152146

C -1.147366 -0.634033 0.105537

C -0.913833 -2.061020 0.603186

C 0.292901 -2.577456 0.893577

C 1.590608 -1.877139 0.515931

C 1.251863 -0.820793 -0.514963

C 0.191376 0.135930 0.059005

C -3.013228 1.137145 0.578244

C -2.181856 -2.858812 0.812667

C 0.459821 -3.918421 1.560723

C 2.264054 -0.043427 -1.333476

C 3.004136 1.203531 -0.941993

C 1.640656 1.316984 -1.587563

C 0.320229 1.414709 -0.832516

C 3.493278 1.684569 0.400687

C 4.857063 1.060065 0.807689

C 4.646603 -0.417713 1.052295

C 5.424085 1.753452 2.043599

C -4.184054 0.433825 -0.058729

C -4.177795 0.090777 -1.411624

C -5.229784 -0.626753 -1.972127

C -6.308168 -1.015699 -1.184332

C -6.327508 -0.678759 0.166080

C -5.273608 0.038642 0.721021

C -1.017573 2.582794 -2.433874

C -2.098016 2.439676 -3.468127

H 4.265340 -1.986134 0.043324

N -0.986103 0.721772 1.986152

O 1.275588 0.778861 2.228041

H -1.641677 -0.653892 -0.863099

H 0.710411 -1.370096 -1.296731

O 2.561060 -2.827666 0.017052

O 4.334178 -0.905422 2.116588

O 4.772711 -1.151551 -0.069505

H 2.692228 -0.602884 -2.154461

H 1.613049 1.754178 -2.576109

O -0.743432 1.404622 -1.828958

O -0.467164 3.620445 -2.149803

H -7.164882 -0.972680 0.786745

H -7.128186 -1.573420 -1.618725

H -5.206649 -0.881209 -3.024600

H -3.340551 0.382749 -2.030056

H -5.296476 0.298260 1.773402

H -2.509770 1.787425 -0.134357

H -3.363389 1.764474 1.400948

H -2.583329 -0.586429 1.754797

H 2.075250 -1.448573 1.391699

H 0.215971 2.330375 -0.253957

H -1.151612 0.975767 2.948728

H -2.056352 -3.892491 0.490592

H -2.491672 -2.882986 1.861701

H -3.007109 -2.428817 0.243291

H 1.350338 -3.913386 2.190645

H -0.397902 -4.172463 2.181300

H 0.595719 -4.724291 0.833099

H 2.214210 -3.234524 -0.787596

H 3.757308 1.406613 -1.700177

H 2.776084 1.497747 1.191577

H 3.645601 2.766267 0.339261

H 5.549474 1.162154 -0.031842

H 6.381518 1.320442 2.338405

H 5.577455 2.814245 1.838968

H 4.737352 1.659371 2.885159

H -3.067109 2.602843 -2.991665

H -1.963310 3.199767 -4.234160

H -2.095778 1.446612 -3.912570

YYY-141b-c12-gfn0 , delta G = 2.5307 kcal/mol, population = 0.48 %

C 0.215162 0.519214 1.646608

C -2.072001 0.048440 1.205448

C -1.183096 -0.650664 0.139746

C -0.941653 -2.096085 0.574315

C 0.266439 -2.609234 0.864314

C 1.560195 -1.863211 0.559540

C 1.229122 -0.785235 -0.452348

C 0.146579 0.136354 0.142005

C -3.082082 1.070767 0.660007

C -2.205571 -2.916310 0.713060

C 0.443456 -3.979476 1.463954

C 2.238222 0.031260 -1.241627

C 2.954219 1.278332 -0.796447

C 1.594449 1.393983 -1.440663

C 0.266747 1.447695 -0.698482

C 3.447798 1.648102 0.579771

C 4.914567 1.191450 0.780259

C 5.024434 -0.261764 0.372911

C 5.380524 1.443078 2.215757

C -4.236721 0.373489 -0.012243

C -4.220226 0.092420 -1.379172

C -5.256760 -0.619112 -1.975160

C -6.329133 -1.064295 -1.209269

C -6.358152 -0.789897 0.155100

C -5.319982 -0.077993 0.745408

C -1.071655 2.661196 -2.265271

C -2.136744 2.544691 -3.318622

H 4.209162 -1.929768 0.785928

N -1.063478 0.634807 2.073310

O 1.191952 0.721757 2.350051

H -1.664901 -0.632217 -0.835159

H 0.704987 -1.319721 -1.255661

O 2.588838 -2.761143 0.095412

O 5.541610 -0.657144 -0.649859

O 4.420356 -1.089388 1.243202

H 2.668664 -0.490120 -2.086258

H 1.572032 1.870452 -2.411040

O -0.786197 1.463460 -1.705827

O -0.541453 3.694647 -1.931732

H -7.191068 -1.128128 0.758983

H -7.137052 -1.617543 -1.671205

H -5.226046 -0.825215 -3.037956

H -3.386900 0.427907 -1.980704

H -5.350455 0.133144 1.808412

H -2.582631 1.752899 -0.024959

H -3.448861 1.663302 1.501162

H -2.635261 -0.690040 1.774790

H 1.983297 -1.433980 1.465913

H 0.147295 2.340243 -0.087638

H -1.245866 0.857948 3.040463

H -3.021265 -2.465564 0.145943

H -2.062416 -3.930964 0.341932

H -2.541092 -2.995908 1.751354

H 1.280276 -3.974946 2.164612

H -0.446491 -4.309350 1.996656

H 0.678762 -4.730586 0.704316

H 2.342563 -3.114280 -0.768546

H 3.701157 1.538902 -1.542821

H 2.828071 1.216371 1.356634

H 3.421850 2.735098 0.702096

H 5.555216 1.738131 0.085630

H 6.419060 1.138547 2.357875

H 5.305274 2.507630 2.444828

H 4.760002 0.896607 2.925887

H -2.130526 1.562526 -3.786389

H -3.112303 2.701402 -2.853169

H -1.988288 3.322086 -4.064660

YYY-141b-c2-gfn0 , delta G = 3.2656 kcal/mol, population = 0.14 %

C 0.577500 -0.520780 1.706637

C -1.749294 -0.412127 1.268999

C -1.024567 -1.122795 0.089746

C -1.074277 -2.632116 0.319030

C 0.008871 -3.411685 0.481708

C 1.426948 -2.929207 0.196782

C 1.327351 -1.631930 -0.574172

C 0.434986 -0.626416 0.163412

C -2.533700 0.858148 0.904462

C -2.474122 -3.199047 0.411724

C -0.098656 -4.861509 0.880615

C 2.508548 -0.911082 -1.192187

C 3.402890 0.150997 -0.595638

C 2.107312 0.552326 -1.256612

C 0.801875 0.746160 -0.489022

C 3.859105 0.411301 0.819185

C 4.302207 1.882479 1.062329

C 3.084353 2.754222 0.850347

C 5.525928 2.296505 0.247638

C -3.811138 0.506849 0.185918

C -3.864100 0.414923 -1.205627

C -5.029971 0.016559 -1.852127

C -6.164846 -0.301153 -1.113304

C -6.125514 -0.213825 0.275391

C -4.958172 0.186381 0.915917

C -0.279341 2.402386 -1.836376

C -1.346550 2.652294 -2.861532

H 2.127148 3.641224 -0.525413

N -0.641303 -0.173089 2.181372

O 1.562007 -0.707064 2.402418

H -1.494530 -0.873902 -0.859304

H 0.739867 -1.896920 -1.464970

O 2.162964 -3.918677 -0.542067

O 2.171549 2.833645 1.646900

O 3.049808 3.368627 -0.343215

H 2.901644 -1.393604 -2.077620

H 2.178506 1.128781 -2.168350

O -0.220402 1.111359 -1.468937

O 0.431945 3.272004 -1.375957

H -7.005042 -0.454869 0.859464

H -7.073153 -0.610890 -1.614538

H -5.051216 -0.045047 -2.933234

H -2.984733 0.652079 -1.788103

H -4.936113 0.254893 1.997720

H -1.912742 1.521161 0.305524

H -2.766677 1.385006 1.832274

H -2.450245 -1.094633 1.747992

H 1.993022 -2.805208 1.120564

H 0.831949 1.540831 0.249971

H -0.776923 -0.088705 3.177861

H -3.198302 -2.518928 -0.039174

H -2.548594 -4.156807 -0.103521

H -2.787866 -3.365053 1.446768

H -0.129954 -5.524039 0.010494

H 0.775280 -5.155359 1.463631

H -0.989396 -5.054275 1.476649

H 1.698661 -4.075538 -1.374433

H 4.214581 0.328914 -1.294910

H 4.723248 -0.227623 1.024640

H 3.098229 0.148648 1.541844

H 4.535962 1.958707 2.126173

H 5.333533 2.255213 -0.823296

H 5.834669 3.314674 0.489561

H 6.358539 1.627596 0.473922

H -2.259899 2.961104 -2.348265

H -1.033959 3.465812 -3.512436

H -1.559518 1.758585 -3.443321

YYY-141b-c23-gfn0 , delta G = 3.4864 kcal/mol, population = 0.09 %

C 0.573928 -0.513406 1.715336

C -1.751765 -0.404409 1.271910

C -1.025231 -1.123402 0.099167

C -1.077886 -2.631413 0.335710

C 0.005511 -3.409218 0.496256

C 1.425409 -2.924856 0.214678

C 1.327000 -1.635952 -0.561419

C 0.434498 -0.628592 0.172599

C -2.532674 0.865537 0.898726

C -2.481627 -3.191891 0.423060

C -0.093941 -4.860138 0.888029

C 2.510156 -0.917526 -1.178075

C 3.403482 0.147465 -0.583552

C 2.109206 0.545256 -1.248900

C 0.801687 0.741196 -0.485699

C 3.856332 0.416267 0.830889

C 4.295645 1.889709 1.067818

C 3.075874 2.757472 0.850182

C 5.519366 2.302742 0.252716

C -3.808953 0.513952 0.178269

C -3.856601 0.409851 -1.212626

C -5.021677 0.011458 -1.860431

C -6.161263 -0.293851 -1.123621

C -6.127337 -0.194231 0.264373

C -4.960621 0.205773 0.906241

C -0.276964 2.388479 -1.845662

C -1.338327 2.630927 -2.878690

H 2.118250 3.634354 -0.532207

N -0.645388 -0.161793 2.185562

O 1.556468 -0.696231 2.415626

H -1.491856 -0.880361 -0.852938

H 0.746218 -1.910985 -1.450812

O 2.123353 -3.866307 -0.624077

O 2.161800 2.837639 1.645314

O 3.041719 3.366936 -0.345641

H 2.904004 -1.404374 -2.060213

H 2.181817 1.117920 -2.162891

O -0.217673 1.100157 -1.470950

O 0.429582 3.262603 -1.385686

H -7.010526 -0.425628 0.846827

H -7.068900 -0.603779 -1.625941

H -5.038366 -0.060357 -2.940961

H -2.973587 0.636787 -1.793520

H -4.942743 0.283896 1.987473

H -1.908587 1.524143 0.298229

H -2.767090 1.397719 1.823148

H -2.455094 -1.082753 1.753381

H 1.984649 -2.790445 1.144781

H 0.827565 1.539624 0.249313

H -0.782992 -0.070240 3.181114

H -2.560546 -4.148539 -0.093056

H -2.803744 -3.352720 1.456222

H -3.198377 -2.507873 -0.033751

H 0.705763 -5.118174 1.589056

H -1.038986 -5.094426 1.373090

H 0.013484 -5.519521 0.022228

H 2.406327 -4.610256 -0.082028

H 4.216596 0.322813 -1.281823

H 4.721964 -0.219182 1.041678

H 3.094605 0.155600 1.553422

H 4.527849 1.971804 2.131594

H 6.353325 1.636983 0.483354

H 5.328350 2.255594 -0.818189

H 5.825360 3.322838 0.489964

H -1.021583 3.439372 -3.533921

H -1.548230 1.732931 -3.454910

H -2.254375 2.944090 -2.372943

### Table S9. Geometry data of conformers of structure 1c.

YYY-141c-c4-gfn0 , delta G = 0.0000 kcal/mol, population = 53.25 %

C 0.177260 1.090905 2.386897

C 2.250280 1.131236 1.222061

C 1.117081 0.881857 0.185512

C 0.797753 2.178169 -0.541691

C -0.374680 2.824918 -0.456802

C -1.580634 2.241435 0.254028

C -1.415848 0.741822 0.392385

C -0.065184 0.399199 1.025573

C 3.221347 -0.049732 1.400914

C 1.952711 2.727197 -1.350990

C -0.598841 4.181367 -1.072158

C -2.433015 -0.058516 1.201140

C -2.809205 -1.434350 0.715181

C -1.681969 -1.272291 1.714075

C -0.214721 -1.113352 1.330130

C -2.714816 -1.919278 -0.715203

C -3.967161 -1.557782 -1.563064

C -4.106443 -0.052596 -1.545235

C -5.240574 -2.255997 -1.098352

C 4.039638 -0.291286 0.160219

C 3.769199 -1.373635 -0.676695

C 4.481543 -1.561704 -1.856752

C 5.480457 -0.663956 -2.217951

C 5.765734 0.416474 -1.387336

C 5.051039 0.598884 -0.208996

C 0.330146 -3.176998 0.259713

C 0.635687 -3.812700 -1.067204

H -3.071231 1.474254 -1.965900

N 1.488801 1.414459 2.429983

O -0.636432 1.284388 3.277153

H 1.431153 0.130779 -0.537786

H -1.372294 0.359455 -0.628167

O -2.746349 2.574227 -0.541995

O -4.901606 0.568321 -0.870443

O -3.175324 0.551754 -2.303738

H -3.112728 0.475159 1.850653

H -1.862201 -1.651214 2.709495

O 0.179088 -1.843327 0.139324

O 0.211574 -3.769495 1.306614

H 6.546858 1.116328 -1.657106

H 6.036180 -0.806632 -3.136062

H 4.256318 -2.409219 -2.492301

H 2.993093 -2.074622 -0.399791

H 5.282656 1.442070 0.431848

H 2.660966 -0.947571 1.664250

H 3.876344 0.177220 2.246089

H 2.832627 2.011426 0.952132

H -1.705993 2.710919 1.235712

H 0.424613 -1.446527 2.144546

H 1.892959 1.835367 3.253092

H 2.703477 1.955193 -1.522597

H 1.621335 3.091763 -2.323341

H 2.454577 3.558700 -0.847233

H 0.336926 4.704698 -1.256646

H -1.142227 4.109379 -2.017705

H -1.210702 4.797881 -0.410596

H -3.539915 2.304184 -0.058579

H -3.693534 -1.816968 1.212928

H -2.620340 -3.008281 -0.719350

H -1.831914 -1.526748 -1.212431

H -3.742519 -1.850610 -2.591442

H -6.071665 -2.035772 -1.769640

H -5.531139 -1.932981 -0.099761

H -5.090092 -3.336754 -1.084537

H 1.181098 -4.740459 -0.912290

H -0.309417 -4.044638 -1.563929

H 1.195820 -3.138759 -1.712338

YYY-141c-c6-gfn0 , delta G = 0.2353 kcal/mol, population = 35.78 %

C 0.799453 1.011911 1.394801

C 2.239890 0.808754 -0.484305

C 0.773157 0.659693 -0.977554

C 0.291777 1.974693 -1.571383

C -0.708078 2.713102 -1.065754

C -1.589383 2.232265 0.070413

C -1.474591 0.728426 0.208162

C -0.008633 0.299583 0.285899

C 3.069739 -0.479025 -0.627652

C 1.083015 2.433051 -2.776421

C -1.051294 4.082652 -1.591233

C -2.148098 0.025548 1.382645

C -2.756485 -1.332618 1.144174

C -1.328541 -1.223873 1.638182

C -0.109948 -1.185061 0.723046

C -3.230589 -1.861056 -0.192367

C -4.686953 -1.435787 -0.533469

C -4.728939 0.075149 -0.537641

C -5.732272 -2.036037 0.400550

C 4.435519 -0.342338 -0.010016

C 5.484755 0.240714 -0.724387

C 6.731571 0.423868 -0.136641

C 6.947471 0.027622 1.180429

C 5.909743 -0.553643 1.902099

C 4.664059 -0.736634 1.309635

C -0.073321 -3.327775 -0.325591

C -0.279889 -4.040105 -1.631417

H -3.849981 1.517588 -1.390837

N 2.040686 1.213635 0.900561

O 0.417601 1.305085 2.517635

H 0.711315 -0.127846 -1.729282

H -1.852753 0.321666 -0.730143

O -2.947327 2.632425 -0.247782

O -5.176132 0.765475 0.354620

O -4.121715 0.595884 -1.618728

H -2.501364 0.623437 2.211224

H -1.140462 -1.563191 2.646367

O -0.223461 -1.995970 -0.473379

O 0.183119 -3.854981 0.731510

H 6.069973 -0.868372 2.925817

H 7.918308 0.167335 1.638860

H 7.535588 0.872497 -0.706897

H 5.324028 0.549555 -1.751235

H 3.859571 -1.190484 1.876001

H 3.157209 -0.696632 -1.694701

H 2.533930 -1.311967 -0.171278

H 2.757763 1.606078 -1.016018

H -1.307530 2.731249 1.003836

H 0.770788 -1.524293 1.263609

H 2.776825 1.595164 1.474944

H 0.435725 2.856449 -3.544005

H 1.825234 3.195054 -2.520701

H 1.621186 1.595941 -3.224093

H -1.902850 4.049142 -2.275615

H -1.342102 4.737532 -0.767793

H -0.213855 4.542615 -2.111551

H -3.514392 2.435991 0.511250

H -3.409065 -1.638594 1.954470

H -3.204502 -2.953769 -0.175864

H -2.576036 -1.545961 -1.000602

H -4.876961 -1.768094 -1.556825

H -6.740055 -1.775965 0.074002

H -5.614525 -1.672790 1.420578

H -5.645763 -3.123821 0.406905

H 0.011177 -5.082092 -1.532275

H -1.335021 -3.982973 -1.906567

H 0.293068 -3.559313 -2.423993

YYY-141c-c1 , delta G = 1.4276 kcal/mol, population = 4.77 %

C -0.173428 1.094546 -2.376996

C -2.250319 1.116589 -1.218813

C -1.114177 0.888156 -0.180089

C -0.795018 2.202181 0.512832

C 0.378242 2.845278 0.408173

C 1.608609 2.232804 -0.243775

C 1.423043 0.734354 -0.385619

C 0.067974 0.400209 -1.016158

C -3.214029 -0.072236 -1.383772

C -1.948160 2.783074 1.300970

C 0.585195 4.228725 0.972114

C 2.429125 -0.068757 -1.205121

C 2.806224 -1.447005 -0.734162

C 1.670059 -1.275304 -1.722421

C 0.207260 -1.112035 -1.325695

C 2.725578 -1.951144 0.690087

C 3.967183 -1.575715 1.550669

C 3.967774 -0.069968 1.659418

C 5.273663 -2.173413 1.032816

C -4.029742 -0.304397 -0.139439

C -3.757438 -1.380463 0.704886

C -4.468242 -1.560871 1.887058

C -5.467462 -0.661440 2.243128

C -5.754444 0.412983 1.405327

C -5.041302 0.587665 0.224856

C -0.338018 -3.175760 -0.254767

C -0.631834 -3.814056 1.073624

H 4.397605 1.463688 0.644841

N -1.490787 1.394523 -2.429342

O 0.643225 1.305467 -3.260095

H -1.423286 0.149248 0.557833

H 1.377131 0.345467 0.634622

O 2.784459 2.546685 0.537049

O 3.249462 0.537984 2.434546

O 4.737052 0.548466 0.756395

H 3.108247 0.471821 -1.848349

H 1.840087 -1.648916 -2.721713

O -0.180296 -1.843170 -0.132929

O -0.232987 -3.766602 -1.304136

H -6.535795 1.114127 1.671086

H -6.022005 -0.798093 3.162872

H -4.241531 -2.403629 2.528361

H -2.981090 -2.082691 0.432100

H -5.274622 1.425993 -0.421723

H -2.649812 -0.970167 -1.637562

H -3.871159 0.141825 -2.230666

H -2.839488 1.994903 -0.958164

H 1.799593 2.693489 -1.215426

H -0.440475 -1.440402 -2.135264

H -1.893888 1.820794 -3.250117

H -1.612572 3.193280 2.253623

H -2.453984 3.590544 0.762783

H -2.696633 2.018066 1.510954

H -0.346060 4.791470 1.011705

H 1.001067 4.201837 1.983567

H 1.295951 4.783694 0.358089

H 2.652659 2.176978 1.427262

H 3.685219 -1.825922 -1.243892

H 2.656143 -3.042204 0.680252

H 1.834861 -1.586184 1.195969

H 3.771716 -1.947615 2.557775

H 6.101864 -1.940332 1.703592

H 5.529501 -1.794570 0.045061

H 5.182181 -3.259433 0.973398

H 0.317539 -4.075012 1.547323

H -1.160749 -3.131959 1.736108

H -1.203471 -4.726773 0.923170

YYY-141c-c3 , delta G = 1.5355 kcal/mol, population = 3.98 %

C -0.763216 0.917149 -1.470773

C -2.237905 0.845050 0.391152

C -0.778483 0.742442 0.917775

C -0.311455 2.109025 1.392631

C 0.692615 2.800520 0.831196

C 1.628910 2.212317 -0.213602

C 1.492410 0.702712 -0.235296

C 0.022441 0.281924 -0.299679

C -3.069229 -0.428873 0.622271

C -1.124858 2.683920 2.530728

C 1.005003 4.222577 1.224387

C 2.167584 -0.091971 -1.348344

C 2.760396 -1.433630 -1.011509

C 1.336834 -1.347358 -1.524781

C 0.111365 -1.231239 -0.626443

C 3.219335 -1.877647 0.360282

C 4.664298 -1.417081 0.710774

C 4.632393 0.091365 0.763655

C 5.731598 -1.994403 -0.216403

C -4.426018 -0.339606 -0.023337

C -4.635357 -0.829754 -1.313771

C -5.871679 -0.690184 -1.936829

C -6.919336 -0.057110 -1.275577

C -6.722762 0.434403 0.012057

C -5.485150 0.294435 0.630398

C 0.039338 -3.292291 0.573244

C 0.229106 -3.912546 1.927697

H 4.604473 1.590403 -0.385794

N -2.016439 1.142034 -1.017490

O -0.358716 1.138984 -2.601700

H -0.725271 0.023850 1.736326

H 1.853226 0.360916 0.737581

O 2.989106 2.615137 0.065735

O 4.214198 0.712589 1.725685

O 4.996845 0.690429 -0.375798

H 2.539913 0.448067 -2.206833

H 1.153846 -1.756488 -2.507769

O 0.204948 -1.955313 0.626695

O -0.216536 -3.890848 -0.445410

H -7.534585 0.924001 0.535659

H -7.882851 0.049236 -1.757814

H -6.016874 -1.078864 -2.937127

H -3.823081 -1.323951 -1.833273

H -5.339383 0.677926 1.634058

H -3.171716 -0.562791 1.701720

H -2.528265 -1.294620 0.239458

H -2.766310 1.679527 0.850444

H 1.418591 2.634864 -1.198332

H -0.768170 -1.599765 -1.149381

H -2.739770 1.488773 -1.628835

H -0.490633 3.184271 3.262353

H -1.861249 3.416102 2.185987

H -1.672000 1.895834 3.050797

H 1.392191 4.773424 0.366027

H 0.123926 4.743255 1.595554

H 1.774944 4.272963 1.999656

H 3.216393 2.281687 0.950964

H 3.415623 -1.800881 -1.793672

H 3.207867 -2.969951 0.405740

H 2.547869 -1.527379 1.140599

H 4.856319 -1.746601 1.733200

H 6.730718 -1.701402 0.109370

H 5.604238 -1.655599 -1.242859

H 5.678027 -3.084447 -0.203129

H -0.333367 -3.363648 2.682550

H -0.084075 -4.952578 1.903218

H 1.285275 -3.858878 2.199771

YYY-141c-c7-gfn0 , delta G = 1.9308 kcal/mol, population = 2.04 %

C 0.735890 1.046597 2.003078

C 2.317570 2.039016 0.516521

C 1.115630 1.519845 -0.339648

C 0.334093 2.739934 -0.805408

C -0.863065 3.099633 -0.320042

C -1.662323 2.189536 0.591650

C -1.218487 0.760301 0.363983

C 0.294723 0.611753 0.587936

C 3.706081 1.455026 0.198658

C 1.082431 3.602623 -1.798667

C -1.517110 4.415430 -0.649996

C -1.839361 -0.368485 1.177249

C -2.120518 -1.676425 0.481058

C -0.797837 -1.467789 1.188214

C 0.463899 -0.929905 0.516306

C -2.358510 -1.858640 -1.001796

C -3.842027 -1.642504 -1.413351

C -4.223279 -0.240051 -0.998772

C -4.791900 -2.689600 -0.842602

C 3.857665 -0.036591 0.317171

C 3.989081 -0.647159 1.567290

C 4.134949 -2.025663 1.675760

C 4.165194 -2.817213 0.532174

C 4.048780 -2.220565 -0.717957

C 3.891429 -0.842931 -0.821678

C 0.828557 -2.572748 -1.176843

C 0.966587 -2.766967 -2.660198

H -3.612577 1.530222 -1.262285

N 1.863486 1.776306 1.869856

O 0.193500 0.775846 3.064423

H 1.481210 0.965679 -1.203184

H -1.379627 0.575480 -0.698147

O -3.061029 2.373807 0.263126

O -4.893295 0.045930 -0.028188

O -3.652499 0.691883 -1.782552

H -2.382428 -0.122328 2.079191

H -0.629217 -2.057891 2.076801

O 0.603772 -1.278211 -0.884013

O 0.892528 -3.447437 -0.346345

H 4.074065 -2.829651 -1.612860

H 4.273969 -3.890745 0.614756

H 4.228350 -2.481237 2.653585

H 3.980480 -0.039306 2.463078

H 3.802644 -0.384625 -1.799731

H 4.422721 1.946506 0.863152

H 3.959258 1.771109 -0.815632

H 2.410433 3.117975 0.386543

H -1.528867 2.477538 1.640904

H 1.352942 -1.283876 1.030963

H 2.325193 2.153296 2.684128

H 0.421928 3.977789 -2.580094

H 1.552101 4.471018 -1.327606

H 1.875257 3.031858 -2.284369

H -1.976589 4.839943 0.245086

H -0.805733 5.138850 -1.041961

H -2.320833 4.294397 -1.380516

H -3.594098 1.831942 0.861663

H -2.758915 -2.323796 1.072344

H -2.084906 -2.878078 -1.285133

H -1.735987 -1.190206 -1.590226

H -3.871365 -1.677569 -2.505088

H -5.805067 -2.542988 -1.219337

H -4.835731 -2.639843 0.244549

H -4.461173 -3.688686 -1.131115

H 1.283120 -3.785003 -2.869745

H 0.005082 -2.578374 -3.141564

H 1.684359 -2.056068 -3.068743

YYY-141c-c4 , delta G = 3.3666 kcal/mol, population = 0.18 %

C -0.724148 1.084007 -1.977981

C -2.328181 2.026718 -0.483787

C -1.114378 1.522942 0.365899

C -0.343567 2.756100 0.811117

C 0.847284 3.120519 0.313046

C 1.689688 2.195838 -0.552450

C 1.227444 0.768719 -0.339025

C -0.287273 0.627784 -0.567226

C -3.708741 1.423863 -0.166565

C -1.092073 3.633365 1.790954

C 1.468118 4.461479 0.613833

C 1.839922 -0.349954 -1.171129

C 2.117579 -1.672497 -0.507118

C 0.793311 -1.443699 -1.206042

C -0.463570 -0.913553 -0.520811

C 2.361606 -1.894937 0.968554

C 3.842174 -1.669943 1.392359

C 4.138059 -0.213487 1.131224

C 4.819695 -2.643515 0.737829

C -3.854539 -0.066245 -0.308955

C -3.983639 -0.656862 -1.568916

C -4.130681 -2.033304 -1.699489

C -4.164538 -2.842816 -0.568683

C -4.050219 -2.266184 0.690961

C -3.891661 -0.890496 0.816829

C -0.827264 -2.583557 1.146575

C -0.966241 -2.801676 2.626654

H 4.547890 0.961844 -0.290177

N -1.871854 1.781615 -1.839639

O -0.166351 0.850623 -3.039920

H -1.466282 0.967447 1.234424

H 1.383404 0.568129 0.723395

O 3.087569 2.355549 -0.232319

O 3.786348 0.679788 1.882805

O 4.719785 0.027670 -0.049583

H 2.389847 -0.081280 -2.061072

H 0.619481 -2.015606 -2.105634

O -0.601215 -1.284876 0.874590

O -0.891811 -3.445205 0.302535

H -4.077851 -2.889337 1.576064

H -4.274527 -3.914774 -0.668531

H -4.222282 -2.473233 -2.684645

H -3.972201 -0.034779 -2.454820

H -3.805156 -0.447612 1.802179

H -4.433376 1.921024 -0.818131

H -3.957708 1.721891 0.854266

H -2.437526 3.103177 -0.346467

H 1.620520 2.472919 -1.607329

H -1.356292 -1.254881 -1.037522

H -2.329190 2.173873 -2.649114

H -1.885702 3.071569 2.285960

H -0.428908 4.020047 2.565103

H -1.559312 4.496490 1.307716

H 2.054318 4.803977 -0.239874

H 0.714957 5.215305 0.837071

H 2.154814 4.414961 1.464255

H 3.196948 2.149428 0.712553

H 2.750862 -2.308046 -1.116357

H 2.105005 -2.926428 1.223286

H 1.731570 -1.253363 1.579825

H 3.879077 -1.792689 2.476040

H 4.845229 -2.527528 -0.344113

H 4.523525 -3.668462 0.967851

H 5.832673 -2.491552 1.113443

H -0.005291 -2.621118 3.112223

H -1.684320 -2.097466 3.046025

H -1.283221 -3.822899 2.819363

### Table S10. Geometry data of conformers of structure 1d.

YYY-141d-c3 , delta G = 0.0000 kcal/mol, population = 56.52 %

C 0.178741 0.906569 2.314130

C 2.345306 1.033067 1.341901

C 1.309120 0.869923 0.194246

C 1.051045 2.220952 -0.462854

C -0.124016 2.871392 -0.437698

C -1.379600 2.248084 0.139969

C -1.218704 0.748825 0.161795

C 0.057808 0.327663 0.887085

C 3.292355 -0.168255 1.518867

C 2.273119 2.814017 -1.129724

C -0.298516 4.264622 -0.979761

C -2.306635 -0.116807 0.762299

C -2.546072 -1.415174 0.051614

C -1.644904 -1.377650 1.261495

C -0.138531 -1.206791 1.048812

C -3.938408 -1.994045 0.077503

C -4.810087 -1.407749 -1.068536

C -4.578410 0.091710 -1.119153

C -6.285729 -1.750170 -0.882839

C 4.194757 -0.350484 0.327332

C 5.233779 0.548264 0.074381

C 6.024629 0.420817 -1.061951

C 5.789380 -0.611977 -1.965673

C 4.763591 -1.518473 -1.719679

C 3.975198 -1.385546 -0.581379

C 0.440561 -3.173057 -0.173803

C 0.835369 -3.685998 -1.529872

H -3.323234 1.341208 -1.815819

N 1.478682 1.230615 2.495195

O -0.710766 1.027451 3.142838

H 1.688605 0.176568 -0.554678

H -1.086030 0.460160 -0.885167

O -2.515104 2.616456 -0.691211

O -5.101642 0.897871 -0.377203

O -3.664783 0.443512 -2.039315

H -3.110311 0.332806 1.326351

H -1.966245 -1.857906 2.174646

O 0.341495 -1.828922 -0.170729

O 0.219906 -3.859516 0.796382

H 4.576193 -2.329011 -2.413059

H 6.404324 -0.710929 -2.851199

H 6.825899 1.126742 -1.241957

H 5.425069 1.354818 0.772856

H 3.177264 -2.091766 -0.393596

H 2.705074 -1.072062 1.686803

H 3.885908 -0.000614 2.421499

H 2.950546 1.925917 1.190739

H -1.567254 2.640322 1.144562

H 0.422099 -1.625808 1.881046

H 1.808301 1.581679 3.381832

H 2.024484 3.270126 -2.087779

H 2.747370 3.586159 -0.516403

H 3.023345 2.043851 -1.312082

H -0.783543 4.254678 -1.959101

H -0.948020 4.844390 -0.320170

H 0.649062 4.790812 -1.071876

H -3.303993 2.624995 -0.132854

H -2.044769 -1.515247 -0.904714

H -4.415340 -1.767239 1.034656

H -3.924552 -3.081468 -0.028784

H -4.447123 -1.819914 -2.011761

H -6.410557 -2.831032 -0.798810

H -6.883629 -1.403300 -1.727292

H -6.675347 -1.286329 0.023930

H -0.068861 -3.796454 -2.133061

H 1.495100 -2.987762 -2.041695

H 1.307958 -4.660025 -1.430438

YYY-141d-c1 , delta G = 0.4800 kcal/mol, population = 25.11 %

C 0.182722 0.908513 2.320681

C 2.347727 1.031309 1.344145

C 1.307358 0.875061 0.199343

C 1.043305 2.232131 -0.442631

C -0.134654 2.877405 -0.409526

C -1.402368 2.238740 0.135042

C -1.221018 0.742381 0.172546

C 0.059433 0.328567 0.894301

C 3.294556 -0.171531 1.511302

C 2.261607 2.842622 -1.100234

C -0.305732 4.280727 -0.930483

C -2.301461 -0.120971 0.783844

C -2.553608 -1.423619 0.085249

C -1.638249 -1.378535 1.284938

C -0.133367 -1.207162 1.056615

C -3.955682 -1.978453 0.131053

C -4.804126 -1.429049 -1.051053

C -4.420934 0.014807 -1.315053

C -6.301987 -1.619201 -0.809430

C 4.190808 -0.348392 0.314267

C 5.228686 0.551351 0.060130

C 6.013354 0.429406 -1.081096

C 5.772978 -0.598790 -1.988697

C 4.748338 -1.506290 -1.741645

C 3.966126 -1.378850 -0.598451

C 0.433254 -3.169703 -0.178741

C 0.812442 -3.676453 -1.541515

H -4.370733 1.705952 -0.476075

N 1.484986 1.224959 2.501007

O -0.705343 1.033999 3.149817

H 1.684259 0.187811 -0.556559

H -1.087629 0.439988 -0.872732

O -2.557775 2.585628 -0.664841

O -3.702144 0.368454 -2.229905

O -4.896697 0.879911 -0.405004

H -3.098182 0.335629 1.350326

H -1.949793 -1.850426 2.205788

O 0.334211 -1.825482 -0.169439

O 0.224052 -3.859719 0.791404

H 4.556906 -2.313232 -2.438119

H 6.383020 -0.693362 -2.878085

H 6.813779 1.136080 -1.261931

H 5.424112 1.354339 0.761573

H 3.169015 -2.085844 -0.409995

H 2.707754 -1.075812 1.677857

H 3.892599 -0.008294 2.411757

H 2.953595 1.923987 1.195162

H -1.637400 2.635260 1.125418

H 0.436009 -1.627175 1.882306

H 1.816210 1.578787 3.385882

H 2.007378 3.321626 -2.045649

H 2.736390 3.601448 -0.470702

H 3.013251 2.079101 -1.304169

H 0.631755 4.833564 -0.929931

H -0.702330 4.289503 -1.949695

H -1.024382 4.824365 -0.314485

H -2.496350 2.112124 -1.509536

H -2.062648 -1.539097 -0.875111

H -4.430312 -1.696926 1.074195

H -3.965246 -3.069718 0.075942

H -4.509460 -1.963390 -1.955405

H -6.627655 -1.062246 0.069512

H -6.516588 -2.676383 -0.643750

H -6.889810 -1.284978 -1.665942

H 1.269112 -4.659089 -1.454013

H -0.095983 -3.764457 -2.142064

H 1.481588 -2.984147 -2.049356

YYY-141d-c5 , delta G = 1.0216 kcal/mol, population = 10.06 %

C 0.759454 0.869041 1.318734

C 2.412400 0.818120 -0.388192

C 1.015776 0.720832 -1.062193

C 0.600802 2.082967 -1.606273

C -0.458203 2.787612 -1.174332

C -1.449023 2.230101 -0.171301

C -1.347196 0.726150 -0.155862

C 0.090128 0.262170 0.065753

C 3.254401 -0.460155 -0.542322

C 1.530516 2.623876 -2.670588

C -0.754301 4.187297 -1.642356

C -2.167484 -0.070780 0.836496

C -2.691998 -1.376661 0.317510

C -1.412092 -1.347168 1.115191

C -0.080789 -1.254387 0.367235

C -3.994254 -1.891129 0.876854

C -5.210611 -1.296789 0.112844

C -4.970387 0.190278 -0.077088

C -6.520604 -1.572565 0.845371

C 4.538401 -0.389673 0.239845

C 4.628649 -0.951043 1.514444

C 5.795893 -0.834186 2.263235

C 6.892228 -0.152486 1.745044

C 6.814212 0.410600 0.474013

C 5.645472 0.293133 -0.269901

C 0.004223 -3.297677 -0.859165

C -0.088063 -3.889790 -2.236357

H -4.010687 1.366130 -1.225694

N 2.049949 1.103498 0.993873

O 0.245586 1.067300 2.409489

H 1.046500 -0.001460 -1.878567

H -1.621359 0.407815 -1.166316

O -2.792666 2.634687 -0.557792

O -5.169739 1.036715 0.769716

O -4.439430 0.479564 -1.277242

H -2.695295 0.432961 1.632742

H -1.395727 -1.786230 2.102526

O -0.081183 -1.952398 -0.903468

O 0.128933 -3.922340 0.168156

H 7.665188 0.938183 0.061220

H 7.802168 -0.063394 2.324961

H 5.849231 -1.278005 3.249595

H 3.778269 -1.485404 1.921126

H 5.591666 0.731674 -1.260095

H 3.465277 -0.588103 -1.606859

H 2.669519 -1.323546 -0.222914

H 2.987897 1.654135 -0.784135

H -1.252116 2.647911 0.820876

H 0.727530 -1.660738 0.970480

H 2.712635 1.426722 1.682199

H 2.128257 1.820681 -3.104422

H 0.980074 3.098874 -3.482275

H 2.227542 3.366985 -2.272131

H -1.558209 4.198919 -2.382493

H -1.098565 4.796654 -0.803759

H 0.118506 4.670434 -2.075974

H -3.313253 2.752505 0.247266

H -2.577589 -1.526188 -0.750338

H -4.074342 -1.618868 1.932643

H -4.055300 -2.980533 0.817211

H -5.235715 -1.746874 -0.881262

H -7.379191 -1.219797 0.271614

H -6.529557 -1.072255 1.814290

H -6.638533 -2.644956 1.009669

H 0.556488 -3.350229 -2.929745

H 0.185309 -4.940890 -2.203619

H -1.114600 -3.794769 -2.596619

YYY-141d-c2 , delta G = 1.6146 kcal/mol, population = 3.69 %

C 0.756497 0.864667 1.329593

C 2.411256 0.823783 -0.375996

C 1.015192 0.726218 -1.051207

C 0.596595 2.091729 -1.584620

C -0.465388 2.789557 -1.148168

C -1.475931 2.216827 -0.167073

C -1.348419 0.715263 -0.146077

C 0.090903 0.258822 0.074155

C 3.256752 -0.451544 -0.535483

C 1.527691 2.645992 -2.641099

C -0.759556 4.195098 -1.602484

C -2.160532 -0.082075 0.848764

C -2.693614 -1.387996 0.339218

C -1.404939 -1.356817 1.124960

C -0.077445 -1.260510 0.368642

C -3.999621 -1.875321 0.915942

C -5.205866 -1.308165 0.114117

C -4.893122 0.109189 -0.327490

C -6.510872 -1.418908 0.903198

C 4.540264 -0.380462 0.247371

C 4.630984 -0.944433 1.520793

C 5.797401 -0.826445 2.270668

C 6.892494 -0.141013 1.754734

C 6.814046 0.424599 0.484865

C 5.646091 0.305990 -0.260124

C 0.003061 -3.295082 -0.872933

C -0.094578 -3.876505 -2.254249

H -4.476004 1.815228 0.367256

N 2.047204 1.102113 1.006939

O 0.241055 1.059451 2.419979

H 1.048221 0.009152 -1.872182

H -1.616879 0.383599 -1.156004

O -2.827853 2.603649 -0.509288

O -4.548410 0.406398 -1.455134

O -4.967562 1.017001 0.659125

H -2.681023 0.424404 1.646608

H -1.380080 -1.791494 2.114052

O -0.085949 -1.949599 -0.906615

O 0.135577 -3.926954 0.148952

H 7.664027 0.955152 0.073833

H 7.801773 -0.050916 2.335530

H 5.851080 -1.272186 3.256141

H 3.781501 -1.481552 1.925673

H 5.591849 0.746677 -1.249323

H 3.468323 -0.574215 -1.600502

H 2.674209 -1.317908 -0.219852

H 2.985014 1.662838 -0.767879

H -1.322373 2.640263 0.828107

H 0.734404 -1.670504 0.964589

H 2.707470 1.428392 1.696093

H 2.124494 1.848107 -3.086085

H 0.978098 3.133432 -3.446007

H 2.225619 3.382628 -2.232238

H -1.535592 4.215856 -2.372425

H -1.139693 4.785812 -0.766411

H 0.122991 4.693811 -1.997647

H -3.097959 2.103350 -1.295580

H -2.589660 -1.548542 -0.728491

H -4.080118 -1.555432 1.957782

H -4.069371 -2.965713 0.905515

H -5.290847 -1.874716 -0.814252

H -7.363296 -1.074618 0.315283

H -6.462909 -0.827731 1.818033

H -6.687635 -2.460382 1.177542

H 0.559924 -3.341025 -2.941616

H 0.164820 -4.931302 -2.228284

H -1.118213 -3.764129 -2.617457

YYY-141d-c6 , delta G = 1.9265 kcal/mol, population = 2.18 %

C 0.732759 1.044336 1.871316

C 2.492776 1.998864 0.572413

C 1.366930 1.539625 -0.411240

C 0.681630 2.794541 -0.940275

C -0.552098 3.194610 -0.594169

C -1.469572 2.311365 0.227590

C -1.045330 0.876780 0.035617

C 0.423571 0.649396 0.411964

C 3.878331 1.353166 0.388827

C 1.568319 3.635183 -1.834622

C -1.124406 4.528015 -0.992190

C -1.793715 -0.237372 0.735517

C -2.000158 -1.475583 -0.087844

C -0.830425 -1.389712 0.857713

C 0.521804 -0.902418 0.325558

C -3.218364 -2.318230 0.193510

C -4.454726 -1.807541 -0.599951

C -4.477402 -0.292891 -0.513909

C -5.749212 -2.426543 -0.080461

C 3.934749 -0.146610 0.481819

C 3.915457 -0.787714 1.723141

C 3.963161 -2.174587 1.810242

C 4.042950 -2.944686 0.654401

C 4.076602 -2.318232 -0.586125

C 4.018933 -0.931537 -0.669416

C 0.889048 -2.524515 -1.386584

C 1.113469 -2.691803 -2.863239

H -3.671528 1.218870 -1.337954

N 1.891520 1.737327 1.867776

O 0.074616 0.776728 2.865942

H 1.799502 0.986144 -1.243641

H -1.109408 0.704819 -1.042540

O -2.834626 2.501120 -0.229095

O -4.894793 0.340763 0.434697

O -3.915132 0.295778 -1.582838

H -2.479764 -0.013894 1.538837

H -0.817098 -2.019931 1.735082

O 0.737733 -1.224568 -1.071237

O 0.843987 -3.420934 -0.578180

H 4.141229 -2.910826 -1.490096

H 4.073283 -4.024456 0.719476

H 3.939613 -2.653900 2.780851

H 3.864507 -0.196761 2.628726

H 4.042327 -0.450744 -1.640226

H 4.542326 1.789623 1.140390

H 4.255270 1.677839 -0.583623

H 2.643995 3.074323 0.471667

H -1.432720 2.600731 1.283874

H 1.350306 -1.315708 0.893623

H 2.279494 2.086836 2.731432

H 2.412494 3.048373 -2.199333

H 1.024173 4.003864 -2.704280

H 1.978936 4.506707 -1.316692

H -1.848821 4.426494 -1.804094

H -1.664983 4.968936 -0.151662

H -0.353340 5.228338 -1.305566

H -3.430481 2.224997 0.481551

H -1.766427 -1.373488 -1.141694

H -3.448561 -2.286219 1.261784

H -3.052934 -3.365179 -0.071812

H -4.311220 -2.066270 -1.650553

H -5.935050 -2.115015 0.947948

H -5.678318 -3.515251 -0.104107

H -6.605488 -2.127995 -0.687609

H 1.272997 -3.741369 -3.094155

H 0.247230 -2.319845 -3.412800

H 1.978207 -2.105300 -3.175880

YYY-141d-c2-gfn0 , delta G = 1.9641 kcal/mol, population = 2.05 %

C 0.144901 1.187142 2.337642

C 2.303695 0.967844 1.363178

C 1.273202 1.184803 0.217163

C 1.317528 2.636034 -0.235772

C 0.303268 3.503085 -0.089267

C -1.083853 3.081998 0.376730

C -1.241125 1.588566 0.206102

C -0.074379 0.832641 0.847636

C 2.985239 -0.412000 1.351230

C 2.647401 3.055740 -0.823550

C 0.443723 4.971075 -0.400426

C -2.490566 0.895322 0.735534

C -3.010643 -0.227375 -0.107139

C -2.071765 -0.524564 1.044504

C -0.568257 -0.636761 0.839765

C -4.453773 -0.667180 0.040818

C -4.823954 -1.927636 -0.786793

C -3.675401 -2.909597 -0.735725

C -5.187959 -1.582457 -2.227870

C 3.915532 -0.556789 0.175710

C 5.126961 0.138549 0.142224

C 5.961996 0.062456 -0.966381

C 5.598812 -0.714612 -2.063272

C 4.399080 -1.417602 -2.038547

C 3.566483 -1.336270 -0.926661

C -0.233971 -2.572550 -0.517957

C 0.207005 -3.071389 -1.860582

H -2.566947 -3.789258 0.516095

N 1.480688 1.178521 2.545094

O -0.709592 1.394678 3.184938

H 1.508282 0.534805 -0.624778

H -1.167475 1.402012 -0.872529

O -2.113693 3.791067 -0.332100

O -2.977140 -3.204619 -1.680957

O -3.460742 -3.394267 0.500287

H -3.158287 1.433367 1.393848

H -2.488080 -1.058931 1.885487

O -0.183765 -1.233885 -0.430323

O -0.601105 -3.284060 0.396324

H 4.110198 -2.030192 -2.883606

H 6.248661 -0.774793 -2.927101

H 6.897706 0.607584 -0.974161

H 5.419439 0.744646 0.992208

H 2.634155 -1.884533 -0.913909

H 2.227227 -1.195829 1.334482

H 3.540729 -0.523918 2.285895

H 3.082064 1.728917 1.329260

H -1.237895 3.370087 1.419346

H -0.120299 -1.239052 1.626503

H 1.864349 1.339978 3.464167

H 2.513747 3.682451 -1.705357

H 3.254448 3.623918 -0.112320

H 3.231881 2.183548 -1.118850

H 0.050491 5.218594 -1.390598

H -0.130730 5.561835 0.315573

H 1.480575 5.299456 -0.360996

H -2.050772 3.551677 -1.265600

H -2.599585 -0.296724 -1.108995

H -5.119918 0.138990 -0.276822

H -4.660082 -0.850029 1.096403

H -5.678812 -2.403378 -0.299369

H -4.366863 -1.067658 -2.728494

H -6.061546 -0.929333 -2.240327

H -5.418439 -2.477990 -2.805232

H 0.875272 -3.921028 -1.725587

H -0.683920 -3.417727 -2.385318

H 0.692490 -2.292332 -2.442900

YYY-141d-c6-gfn0 , delta G = 2.9424 kcal/mol, population = 0.39 %

C 0.714427 0.940158 1.940255

C 2.487035 1.962937 0.712622

C 1.366982 1.565574 -0.304214

C 0.684476 2.851842 -0.755741

C -0.551183 3.229221 -0.390866

C -1.494007 2.289751 0.344595

C -1.048379 0.873179 0.083269

C 0.417946 0.628712 0.458183

C 3.872553 1.325676 0.500882

C 1.574690 3.749918 -1.588388

C -1.113294 4.591123 -0.702150

C -1.798331 -0.277737 0.714275

C -2.002735 -1.470139 -0.174610

C -0.836587 -1.434188 0.779956

C 0.518597 -0.916502 0.284252

C -3.228752 -2.316379 0.061085

C -4.439749 -1.781900 -0.755704

C -4.389332 -0.267013 -0.780721

C -5.765223 -2.333384 -0.229978

C 3.927259 -0.176929 0.514370

C 4.017919 -0.900075 -0.676170

C 4.074226 -2.289305 -0.665797

C 4.032759 -2.980356 0.539704

C 3.946529 -2.272257 1.734098

C 3.900176 -0.882691 1.719998

C 0.897185 -2.438995 -1.514600

C 1.131840 -2.522465 -2.996722

H -4.423693 1.230886 0.369161

N 1.875448 1.628139 1.985924

O 0.047147 0.618634 2.912389

H 1.804485 1.063900 -1.166429

H -1.096241 0.754450 -1.005027

O -2.864453 2.475889 -0.074006

O -3.993889 0.382250 -1.730652

O -4.760758 0.309571 0.373311

H -2.488753 -0.093786 1.522428

H -0.827933 -2.110558 1.622287

O 0.743198 -1.158855 -1.127187

O 0.847496 -3.379604 -0.758402

H 3.916912 -2.802066 2.677932

H 4.062067 -4.062092 0.547876

H 4.143721 -2.833491 -1.599360

H 4.047311 -0.368754 -1.620111

H 3.844123 -0.340300 2.655192

H 4.533710 1.720527 1.277555

H 4.253924 1.701469 -0.451231

H 2.641936 3.041858 0.675958

H -1.509275 2.514017 1.414429

H 1.344028 -1.360546 0.833191

H 2.255842 1.928099 2.871282

H 1.026210 4.199817 -2.415999

H 2.006998 4.568216 -1.005073

H 2.404178 3.182201 -2.012680

H -0.330594 5.325648 -0.881572

H -1.765277 4.569666 -1.579883

H -1.727916 4.941507 0.129224

H -2.967516 2.103537 -0.965031

H -1.763896 -1.317012 -1.221594

H -3.481619 -2.305624 1.124191

H -3.062928 -3.358530 -0.222422

H -4.305135 -2.081801 -1.795916

H -5.742814 -3.424345 -0.249012

H -6.606497 -2.001695 -0.840797

H -5.938815 -2.012802 0.797524

H 2.002304 -1.924072 -3.268417

H 1.287846 -3.557914 -3.286347

H 0.272123 -2.114470 -3.530654

### Table S11. Geometry data of conformers of structure 1e.

YYY-141e-c2 , delta G = 0.0000 kcal/mol, population = 37.07 %

C -0.312223 -0.693737 1.016933

C -2.299524 -0.873590 -0.274239

C -1.079347 -0.821038 -1.229554

C -0.661860 -2.239394 -1.613321

C 0.536862 -2.779221 -1.332910

C 1.685771 -1.957774 -0.766011

C 1.390506 -0.495525 -1.014667

C 0.025599 -0.145695 -0.399513

C -3.243790 0.333824 -0.394904

C -1.745229 -3.035488 -2.305665

C 0.855409 -4.230868 -1.578516

C 2.355682 0.601410 -0.641939

C 2.423715 1.094712 0.772274

C 1.523494 1.792065 -0.217542

C 0.058502 1.409197 -0.312252

C 3.693612 1.784535 1.215937

C 4.791702 0.796400 1.682300

C 5.009549 -0.209793 0.570026

C 6.083060 1.529278 2.039103

C -4.331634 0.305487 0.645390

C -4.196488 1.025314 1.833500

C -5.172430 0.956380 2.823034

C -6.300087 0.163259 2.637181

C -6.446408 -0.558152 1.455343

C -5.468611 -0.486793 0.469310

C -0.992067 3.169665 -1.558554

C -1.486540 3.549645 -2.924690

H 4.252947 -1.849045 -0.053168

N -1.635613 -0.961808 1.017125

O 0.425910 -0.832669 1.981406

H -1.322742 -0.248323 -2.124155

H 1.245927 -0.397727 -2.098907

O 2.954946 -2.358535 -1.329918

O 5.624657 0.018270 -0.450231

O 4.383946 -1.374139 0.798448

H 3.217289 0.745819 -1.278039

H 1.776220 2.799477 -0.520331

O -0.467207 1.926745 -1.565964

O -1.053618 3.863255 -0.570089

H -7.324319 -1.173084 1.300214

H -7.061887 0.110670 3.404744

H -5.052701 1.524296 3.737290

H -3.321360 1.646488 1.982115

H -5.591108 -1.048169 -0.450279

H -3.676140 0.314089 -1.398292

H -2.673430 1.256893 -0.304230

H -2.888585 -1.776198 -0.431298

H 1.808357 -2.150578 0.300331

H -0.552049 1.804695 0.496386

H -2.095159 -1.284169 1.854959

H -2.290308 -3.684792 -1.614004

H -2.476594 -2.368621 -2.765905

H -1.337418 -3.669675 -3.092006

H 1.398176 -4.376089 -2.516951

H 1.503388 -4.608603 -0.785535

H -0.039955 -4.848343 -1.609128

H 2.978111 -2.121235 -2.265898

H 1.956060 0.474959 1.523468

H 3.482769 2.465774 2.044468

H 4.097489 2.389120 0.398403

H 4.416105 0.252425 2.550396

H 5.889464 2.274907 2.812029

H 6.489900 2.036624 1.163476

H 6.840229 0.838964 2.414891

H -2.229490 2.826228 -3.263432

H -1.923582 4.543687 -2.893941

H -0.659115 3.529283 -3.635385

YYY-141e-c8 , delta G = 0.1606 kcal/mol, population = 28.26 %

C -0.348907 -0.697228 1.023082

C -2.333805 -0.874930 -0.271581

C -1.113118 -0.828905 -1.225720

C -0.705441 -2.248935 -1.615115

C 0.493101 -2.795314 -1.349669

C 1.650239 -1.990365 -0.779824

C 1.355674 -0.524737 -1.014187

C -0.003353 -0.160349 -0.395865

C -3.272225 0.337361 -0.390222

C -1.798296 -3.032208 -2.308936

C 0.807242 -4.242072 -1.621143

C 2.333555 0.560985 -0.638680

C 2.395551 1.055219 0.775803

C 1.512209 1.762074 -0.220599

C 0.043126 1.394661 -0.315362

C 3.659068 1.732053 1.247212

C 4.719489 0.717883 1.730964

C 5.084280 -0.221574 0.605315

C 5.950005 1.421038 2.312334

C -4.360449 0.310772 0.649784

C -4.227439 1.033270 1.836529

C -5.203151 0.963525 2.826349

C -6.328400 0.166580 2.642398

C -6.472553 -0.557714 1.462023

C -5.495156 -0.485313 0.475773

C -0.993468 3.159914 -1.566775

C -1.495927 3.534347 -2.931759

H 6.003998 -0.287892 -1.045999

N -1.671915 -0.965978 1.020049

O 0.380705 -0.825086 1.996715

H -1.352591 -0.252325 -2.119040

H 1.217678 -0.425438 -2.096959

O 2.860048 -2.390947 -1.438184

O 4.749725 -1.386160 0.528983

O 5.821560 0.369452 -0.355016

H 3.196134 0.699468 -1.276458

H 1.778476 2.766911 -0.520366

O -0.478218 1.914076 -1.570085

O -1.044372 3.860788 -0.582336

H -7.348387 -1.176027 1.308448

H -7.089815 0.113068 3.410306

H -5.084887 1.533544 3.739522

H -3.353593 1.656567 1.984076

H -5.615491 -1.049780 -0.442171

H -3.704444 0.322129 -1.393752

H -2.697545 1.257565 -0.296877

H -2.927350 -1.774399 -0.429500

H 1.752404 -2.194066 0.291647

H -0.561949 1.801136 0.492120

H -2.134859 -1.280428 1.858975

H -1.398899 -3.663403 -3.101934

H -2.347833 -3.682205 -1.621196

H -2.525701 -2.356264 -2.762860

H -0.089088 -4.856020 -1.683169

H 1.371582 -4.359710 -2.549795

H 1.439901 -4.639631 -0.825144

H 3.591945 -2.154379 -0.847699

H 1.911833 0.441574 1.521877

H 3.436240 2.398030 2.085041

H 4.087726 2.349917 0.453375

H 4.264340 0.085355 2.494156

H 6.701261 0.702120 2.643161

H 5.652347 2.019804 3.174157

H 6.408105 2.082002 1.576205

H -1.903398 4.541151 -2.910363

H -0.681665 3.476641 -3.655257

H -2.265946 2.828370 -3.246519

YYY-141e-c1 , delta G = 0.6708 kcal/mol, population = 11.93 %

C 0.261216 -0.495796 1.852848

C -2.058613 -0.757513 1.402368

C -1.280565 -0.771989 0.058956

C -1.039996 -2.218257 -0.365385

C 0.174312 -2.780187 -0.490104

C 1.456019 -1.963263 -0.425083

C 1.102603 -0.511976 -0.663455

C 0.053614 -0.069060 0.371342

C -3.043796 0.411993 1.566894

C -2.307668 -3.014082 -0.585028

C 0.365552 -4.253264 -0.740397

C 2.146591 0.572851 -0.749516

C 2.725513 1.164473 0.500998

C 1.533884 1.820310 -0.150361

C 0.124669 1.485039 0.303921

C 4.074823 1.838252 0.397745

C 5.258873 0.846446 0.513600

C 5.053345 -0.239558 -0.523604

C 6.597989 1.556716 0.326959

C -4.242574 0.244616 0.669932

C -5.219318 -0.710191 0.964819

C -6.296892 -0.914869 0.110914

C -6.417109 -0.164179 -1.055706

C -5.454620 0.793218 -1.356905

C -4.377310 0.991936 -0.499055

C -1.154983 3.214761 -0.751060

C -1.965124 3.560701 -1.969127

H 4.114765 -1.893648 -0.703625

N -0.968077 -0.725727 2.362254

O 1.301486 -0.586476 2.488186

H -1.844143 -0.249675 -0.712305

H 0.577137 -0.485680 -1.626987

O 2.438358 -2.443464 -1.371254

O 5.251545 -0.098133 -1.711913

O 4.554311 -1.368017 0.002545

H 2.721664 0.640695 -1.661991

H 1.671268 2.793189 -0.602711

O -0.789410 1.916490 -0.746850

O -0.846364 3.999007 0.115944

H -5.541284 1.386476 -2.258813

H -7.256947 -0.321855 -1.720514

H -7.045366 -1.657807 0.357100

H -5.137075 -1.296759 1.872869

H -3.631043 1.735676 -0.739032

H -2.538185 1.355551 1.359752

H -3.360696 0.441039 2.612532

H -2.617555 -1.683011 1.533446

H 1.950076 -2.086424 0.539315

H -0.173800 1.970678 1.230403

H -1.073239 -1.007358 3.325323

H -3.159030 -2.349563 -0.738464

H -2.225714 -3.659536 -1.459464

H -2.549203 -3.655109 0.268138

H 0.527097 -4.470383 -1.800222

H 1.251975 -4.609702 -0.212755

H -0.489167 -4.838082 -0.405929

H 2.122763 -2.278707 -2.269052

H 2.558515 0.618874 1.417827

H 4.185178 2.585250 1.188183

H 4.158707 2.366215 -0.556863

H 5.216966 0.376350 1.497397

H 7.434392 0.868347 0.459586

H 6.700382 2.360925 1.057477

H 6.667086 1.987382 -0.672681

H -2.792631 4.208123 -1.683351

H -1.324310 4.120029 -2.654243

H -2.330896 2.674152 -2.481074

YYY-141e-c7 , delta G = 0.6984 kcal/mol, population = 11.39 %

C 0.226010 -0.499947 1.874907

C -2.091970 -0.764401 1.415591

C -1.309746 -0.785357 0.075497

C -1.074458 -2.233527 -0.347719

C 0.136654 -2.798401 -0.483873

C 1.423812 -1.992198 -0.420305

C 1.069928 -0.538104 -0.646271

C 0.026241 -0.085278 0.388559

C -3.072925 0.409736 1.575378

C -2.349032 -3.020448 -0.565430

C 0.322965 -4.267694 -0.750580

C 2.123636 0.538114 -0.732762

C 2.697746 1.126769 0.521605

C 1.517075 1.791457 -0.138642

C 0.104068 1.468131 0.312738

C 4.048701 1.795148 0.445981

C 5.209888 0.785879 0.587450

C 5.129291 -0.249406 -0.509928

C 6.569992 1.490597 0.610256

C -4.270975 0.245456 0.677008

C -5.256674 -0.698318 0.977330

C -6.333373 -0.901634 0.121973

C -6.443605 -0.160672 -1.051783

C -5.471950 0.785619 -1.358693

C -4.395681 0.983063 -0.499288

C -1.156372 3.201445 -0.758718

C -1.971170 3.542997 -1.975227

H 5.359863 -0.469828 -2.374341

N -1.004781 -0.732812 2.378718

O 1.260369 -0.575302 2.523615

H -1.868935 -0.262013 -0.698330

H 0.550284 -0.512255 -1.610591

O 2.319534 -2.473098 -1.432279

O 4.795132 -1.406810 -0.358144

O 5.450011 0.243613 -1.721918

H 2.697314 0.603609 -1.647330

H 1.667038 2.763529 -0.588628

O -0.805186 1.900437 -0.742025

O -0.834363 3.993000 0.097334

H -5.550199 1.370600 -2.266775

H -7.282370 -0.317705 -1.718124

H -7.088550 -1.636421 0.372203

H -5.181587 -1.277897 1.890482

H -3.641497 1.717138 -0.744244

H -2.562734 1.350475 1.366156

H -3.391876 0.443432 2.620329

H -2.654693 -1.687462 1.547489

H 1.901412 -2.121868 0.556919

H -0.192717 1.962258 1.235415

H -1.114230 -1.005364 3.343883

H -2.600338 -3.655422 0.289684

H -3.193916 -2.348598 -0.724348

H -2.272286 -3.669511 -1.437513

H -0.551049 -4.851967 -0.469181

H 0.540661 -4.457663 -1.804543

H 1.181024 -4.640969 -0.187692

H 3.214339 -2.219367 -1.157722

H 2.517218 0.582179 1.436661

H 4.150364 2.526806 1.251867

H 4.158928 2.339991 -0.495670

H 5.069361 0.230643 1.515661

H 7.389047 0.774993 0.697856

H 6.615855 2.163767 1.467382

H 6.722170 2.077085 -0.296143

H -2.801153 4.186469 -1.687211

H -1.335467 4.105808 -2.662127

H -2.334519 2.654520 -2.485459

YYY-141e-c12 , delta G = 0.8678 kcal/mol, population = 8.55 %

C -0.185924 -0.653891 0.897110

C -2.189519 -0.824543 -0.368881

C -0.992770 -0.683035 -1.342223

C -0.551827 -2.066005 -1.825461

C 0.663634 -2.596722 -1.605533

C 1.795612 -1.794016 -0.982658

C 1.465015 -0.329141 -1.166995

C 0.114787 -0.029914 -0.497047

C -3.162105 0.366643 -0.382234

C -1.634930 -2.828313 -2.557370

C 1.019020 -4.011980 -1.970291

C 2.424900 0.763777 -0.775664

C 2.525430 1.167398 0.661239

C 1.591539 1.916269 -0.259136

C 0.127863 1.518750 -0.323911

C 3.791392 1.821627 1.163008

C 5.002922 0.840721 1.258178

C 4.509443 -0.535207 1.644668

C 5.839184 0.780951 -0.018532

C -4.252290 0.207921 0.643586

C -4.144942 0.807995 1.899267

C -5.123450 0.609461 2.868967

C -6.225710 -0.193658 2.594723

C -6.344285 -0.795509 1.344736

C -5.363889 -0.595812 0.379485

C -1.011762 3.323055 -1.419234

C -1.607367 3.752406 -2.729487

H 3.741863 -1.470674 3.097131

N -1.495326 -0.980289 0.899670

O 0.564730 -0.797604 1.853454

H -1.268817 -0.063135 -2.194969

H 1.295817 -0.197192 -2.241335

O 3.037752 -2.135873 -1.610040

O 4.428216 -1.495145 0.907794

O 4.123912 -0.592821 2.932987

H 3.256836 0.957673 -1.436243

H 1.824802 2.943775 -0.504038

O -0.454362 2.099834 -1.522829

O -1.036560 3.962711 -0.393028

H -7.202412 -1.417289 1.121279

H -6.989084 -0.346358 3.347199

H -5.025557 1.084758 3.837121

H -3.289447 1.436651 2.116258

H -5.463054 -1.064942 -0.593000

H -3.591365 0.432362 -1.384942

H -2.614250 1.290152 -0.199645

H -2.762361 -1.727767 -0.574258

H 1.875877 -2.034290 0.081927

H -0.454198 1.859747 0.529680

H -1.935203 -1.341325 1.732343

H -1.231382 -3.393444 -3.396646

H -2.159761 -3.537250 -1.909640

H -2.384678 -2.141050 -2.954342

H 1.608096 -4.463852 -1.168956

H 0.140680 -4.631445 -2.138280

H 1.645115 -4.044648 -2.865335

H 3.725468 -1.936642 -0.956981

H 2.061610 0.501227 1.374266

H 3.601548 2.248938 2.149100

H 4.088076 2.645139 0.509499

H 5.635939 1.186045 2.078636

H 6.190399 1.781058 -0.275135

H 5.266457 0.390507 -0.857229

H 6.710429 0.138228 0.115513

H -2.457884 3.109565 -2.965019

H -1.939999 4.784429 -2.662036

H -0.878018 3.641084 -3.531817

YYY-141e-c9 , delta G = 2.0369 kcal/mol, population = 1.19 %

C -0.062558 -1.176397 0.934074

C -2.124295 -1.018247 -0.233156

C -0.991660 -1.298817 -1.255453

C -0.931032 -2.796214 -1.546537

C 0.128761 -3.578045 -1.283726

C 1.467950 -3.016376 -0.840017

C 1.500141 -1.540917 -1.168631

C 0.288530 -0.833231 -0.542147

C -2.791188 0.359743 -0.377690

C -2.209272 -3.364381 -2.123344

C 0.093345 -5.075057 -1.439058

C 2.718170 -0.670555 -0.937079

C 2.952392 0.008310 0.384851

C 2.176262 0.717843 -0.690747

C 0.669906 0.677982 -0.596757

C 4.309058 0.573052 0.735108

C 4.224577 1.807976 1.675594

C 3.422180 2.878584 0.959462

C 3.697303 1.451746 3.064732

C -3.812579 0.601646 0.701389

C -3.473910 1.308468 1.856441

C -4.399550 1.484116 2.880688

C -5.680118 0.953487 2.762950

C -6.029227 0.247056 1.615010

C -5.101029 0.072733 0.594665

C -0.013713 2.620720 -1.798884

C -0.678768 3.106485 -3.052012

H 1.625554 3.436310 0.676335

N -1.408642 -1.174541 1.023159

O 0.698237 -1.374415 1.871547

H -1.167977 -0.745949 -2.177966

H 1.333541 -1.490336 -2.251131

O 2.495879 -3.726939 -1.555979

O 3.868735 3.562783 0.063321

O 2.139495 2.953630 1.354373

H 3.557378 -0.801106 -1.608941

H 2.618014 1.583268 -1.161499

O 0.088821 1.280955 -1.786090

O 0.365401 3.334930 -0.892532

H -7.025771 -0.164592 1.513363

H -6.402361 1.092266 3.557505

H -4.120977 2.038134 3.768494

H -2.478270 1.725401 1.951912

H -5.380359 -0.475393 -0.298178

H -3.261317 0.400115 -1.362962

H -2.031554 1.139089 -0.346884

H -2.904262 -1.775752 -0.293107

H 1.608846 -3.182928 0.233798

H 0.293561 1.228984 0.262742

H -1.869095 -1.313044 1.909725

H -2.812978 -2.574429 -2.573915

H -2.005270 -4.103600 -2.897405

H -2.828942 -3.851221 -1.364200

H -0.920502 -5.468323 -1.401141

H 0.550444 -5.390090 -2.380460

H 0.672269 -5.545674 -0.641975

H 3.338186 -3.512425 -1.139576

H 2.384275 -0.371279 1.220664

H 4.840390 0.861949 -0.174997

H 4.916263 -0.185516 1.236175

H 5.235451 2.210645 1.764901

H 4.348904 0.705052 3.522278

H 2.688690 1.044110 3.021274

H 3.677262 2.327474 3.714912

H -0.608986 4.188904 -3.109839

H -0.218432 2.648294 -3.927025

H -1.729349 2.808890 -3.035974

YYY-141e-c11 , delta G = 2.6732 kcal/mol, population = 0.40 %

C 0.359248 -0.514677 1.696864

C -1.963960 -0.815576 1.286955

C -1.223001 -0.733313 -0.072789

C -0.968502 -2.145710 -0.599619

C 0.250524 -2.673497 -0.803942

C 1.518474 -1.841695 -0.692192

C 1.119562 -0.390218 -0.850943

C 0.104926 -0.019212 0.243527

C -2.971326 0.318957 1.542551

C -2.235345 -2.938019 -0.843241

C 0.465374 -4.112111 -1.186365

C 2.140912 0.715298 -0.925552

C 2.763211 1.218010 0.338106

C 1.544252 1.912839 -0.220056

C 0.155049 1.537056 0.265364

C 4.110379 1.900015 0.283316

C 5.290917 0.926543 -0.024213

C 5.023764 -0.410514 0.629102

C 5.570083 0.757513 -1.516315

C -4.198304 0.157358 0.683764

C -4.374757 0.914559 -0.473701

C -5.478484 0.716675 -1.297577

C -6.425743 -0.248699 -0.973670

C -6.263133 -1.009515 0.181185

C -5.158998 -0.806441 1.000798

C -1.172492 3.314693 -0.638230

C -2.070282 3.714114 -1.776388

H 4.911815 -1.222198 2.332455

N -0.849384 -0.810650 2.219280

O 1.413815 -0.595666 2.312870

H -1.817515 -0.176835 -0.795256

H 0.564845 -0.337704 -1.794047

O 2.454003 -2.241876 -1.701541

O 4.683876 -1.425737 0.059222

O 5.174961 -0.362026 1.965865

H 2.663829 0.848793 -1.860842

H 1.664580 2.916475 -0.604411

O -0.804216 2.020811 -0.719939

O -0.811358 4.059057 0.244140

H -6.998521 -1.759732 0.444240

H -7.285652 -0.405398 -1.612556

H -5.597315 1.316490 -2.191445

H -3.639619 1.663818 -0.732396

H -5.041642 -1.401980 1.899193

H -2.497887 1.283730 1.358557

H -3.249363 0.293723 2.599176

H -2.497869 -1.760408 1.379986

H 1.984706 -2.002862 0.284771

H -0.110305 1.964565 1.230024

H -0.925848 -1.134174 3.171902

H -2.171810 -3.519508 -1.762784

H -2.454819 -3.638213 -0.031508

H -3.094522 -2.271070 -0.931136

H 1.313815 -4.520690 -0.632714

H -0.406402 -4.729748 -0.980765

H 0.716550 -4.207974 -2.245597

H 3.328439 -1.996865 -1.363017

H 2.611037 0.608292 1.216475

H 4.295882 2.391739 1.239788

H 4.120000 2.678347 -0.483349

H 6.181627 1.336570 0.457097

H 6.449853 0.133423 -1.679523

H 5.755266 1.732604 -1.968442

H 4.733568 0.290596 -2.031877

H -1.537791 4.439325 -2.394226

H -2.354547 2.863625 -2.390602

H -2.956701 4.207912 -1.378817

YYY-141e-c10-gfn0 , delta G = 2.7962 kcal/mol, population = 0.33 %

C 0.010504 1.181119 -0.849224

C -2.089489 1.033901 0.248917

C -0.980775 1.269854 1.309534

C -0.884178 2.759580 1.622252

C 0.206751 3.510860 1.397510

C 1.557540 2.916290 1.019196

C 1.525945 1.430793 1.299412

C 0.306110 0.781722 0.624850

C -2.796994 -0.327123 0.361016

C -2.159607 3.365719 2.162796

C 0.197813 5.009962 1.560164

C 2.719550 0.525447 1.072709

C 2.956176 -0.115073 -0.264158

C 2.131295 -0.832403 0.773263

C 0.631560 -0.742933 0.658112

C 4.277504 -0.762940 -0.606388

C 4.127135 -1.801545 -1.756267

C 2.879029 -2.631462 -1.517461

C 4.081086 -1.131522 -3.127054

C -3.764730 -0.551893 -0.769616

C -3.370154 -1.246256 -1.914778

C -4.244846 -1.408756 -2.984732

C -5.529459 -0.877815 -2.923895

C -5.933801 -0.183863 -1.786699

C -5.056163 -0.022286 -0.720268

C -0.020773 -2.681038 1.889178

C -0.751293 -3.160676 3.109213

H 2.121909 -3.785575 -0.220673

N -1.332786 1.190393 -0.983106

O 0.804369 1.419749 -1.747281

H -1.199178 0.707527 2.217158

H 1.323537 1.346695 2.376443

O 2.621966 3.558273 1.741539

O 1.846576 -2.532532 -2.139339

O 3.014602 -3.487710 -0.481696

H 3.548584 0.614052 1.762722

H 2.541378 -1.730447 1.205532

O 0.010565 -1.337980 1.835071

O 0.463340 -3.401016 1.040615

H -6.933851 0.227894 -1.729056

H -6.212025 -1.006707 -3.754419

H -3.923075 -1.952546 -3.864188

H -2.371173 -1.662511 -1.967234

H -5.378393 0.515704 0.164174

H -3.320656 -0.348613 1.319856

H -2.057283 -1.125619 0.377963

H -2.851821 1.810408 0.296800

H 1.788322 3.114047 -0.029181

H 0.249371 -1.272193 -0.213226

H -1.761861 1.374545 -1.876909

H -2.798565 2.595067 2.598097

H -1.953882 4.102518 2.938915

H -2.742870 3.868711 1.385491

H 0.906577 5.465781 0.867680

H -0.787133 5.433855 1.370314

H 0.503956 5.315226 2.565245

H 2.472901 3.410735 2.684383

H 2.427231 0.315069 -1.101976

H 4.685886 -1.255219 0.278714

H 5.007683 -0.015315 -0.925994

H 4.983102 -2.479161 -1.707275

H 3.247507 -0.432392 -3.194817

H 3.963186 -1.867022 -3.923551

H 5.007695 -0.582276 -3.300102

H -0.600614 -4.229551 3.232013

H -0.410365 -2.623591 3.993848

H -1.817410 -2.957345 2.987537

YYY-141e-c3 , delta G = 2.9330 kcal/mol, population = 0.26 %

C 0.558820 -1.131809 1.533034

C -1.798160 -0.897701 1.337256

C -1.196324 -1.146090 -0.073949

C -1.318428 -2.627696 -0.411213

C -0.272970 -3.445612 -0.617382

C 1.152807 -2.935085 -0.779533

C 1.104071 -1.445011 -1.032563

C 0.280718 -0.736303 0.055404

C -2.500725 0.459630 1.513487

C -2.741579 -3.139693 -0.438620

C -0.436679 -4.936532 -0.769452

C 2.345697 -0.618399 -1.289574

C 3.125772 -0.013548 -0.156941

C 2.011456 0.769772 -0.797945

C 0.667840 0.766294 -0.106500

C 4.530804 0.494930 -0.379069

C 4.900132 1.684926 0.550327

C 3.925852 2.811971 0.263026

C 4.976236 1.278104 2.021129

C -3.823896 0.475668 0.792688

C -4.949940 -0.108156 1.377962

C -6.160194 -0.165265 0.696240

C -6.263948 0.362055 -0.587995

C -5.150095 0.946835 -1.181198

C -3.941149 1.000466 -0.494426

C -0.283387 2.785842 -0.938270

C -1.288169 3.386671 -1.877477

H 2.200264 3.434107 0.758515

N -0.623777 -1.052955 2.180224

O 1.622576 -1.423893 2.059374

H -1.720148 -0.552731 -0.821190

H 0.507617 -1.329317 -1.948543

O 1.824943 -3.619594 -1.850199

O 3.995671 3.523842 -0.716481

O 2.923640 2.907740 1.153764

H 2.838154 -0.747689 -2.244544

H 2.264700 1.645688 -1.375976

O -0.306106 1.441333 -0.957604

O 0.453184 3.437711 -0.226225

H -5.220912 1.361053 -2.179354

H -7.206005 0.319618 -1.119859

H -7.023231 -0.618733 1.167722

H -4.877776 -0.519268 2.378591

H -3.076553 1.448316 -0.963906

H -1.854816 1.261742 1.157546

H -2.657719 0.622395 2.582259

H -2.518258 -1.674557 1.591098

H 1.753327 -3.164855 0.102631

H 0.684558 1.292710 0.845521

H -0.679814 -1.265237 3.165046

H -2.897655 -3.833680 -1.264587

H -3.011616 -3.667359 0.481065

H -3.446771 -2.315471 -0.555459

H 0.443125 -5.451399 -0.380676

H -1.312618 -5.304735 -0.237505

H -0.533884 -5.234652 -1.817582

H 1.336059 -3.448669 -2.665558

H 2.934079 -0.418833 0.824519

H 4.652325 0.812714 -1.417520

H 5.253527 -0.305404 -0.200349

H 5.876068 2.051642 0.225996

H 5.727847 0.496355 2.144876

H 4.023610 0.895329 2.383812

H 5.259867 2.123229 2.649979

H -0.846368 4.257255 -2.358827

H -1.621484 2.669672 -2.623630

H -2.149202 3.726951 -1.298329

YYY-141e-c8-gfn0 , delta G = 2.9361 kcal/mol, population = 0.26 %

C 0.546180 1.083923 -1.574252

C -1.811156 0.867498 -1.359049

C -1.202611 1.140788 0.044128

C -1.334361 2.625472 0.367364

C -0.297049 3.454999 0.564179

C 1.132671 2.955761 0.676552

C 1.096640 1.474598 0.980797

C 0.274924 0.731684 -0.084567

C -2.511865 -0.494322 -1.507129

C -2.765295 3.116737 0.407943

C -0.465496 4.941422 0.731183

C 2.344365 0.661849 1.258144

C 3.128338 0.026058 0.142793

C 2.016269 -0.740875 0.805183

C 0.670416 -0.763876 0.117183

C 4.534827 -0.472409 0.379189

C 4.906825 -1.687911 -0.515347

C 3.936301 -2.808976 -0.193062

C 4.979371 -1.324178 -1.997511

C -3.831839 -0.501769 -0.780597

C -3.941595 -1.003032 0.516507

C -5.147766 -0.940934 1.207351

C -6.266336 -0.371605 0.608035

C -6.170126 0.131860 -0.686321

C -4.962536 0.066608 -1.371997

C -0.268735 -2.764662 1.006453

C -1.271846 -3.342818 1.961367

H 2.211255 -3.449374 -0.668023

N -0.641222 1.005753 -2.210803

O 1.610681 1.344656 -2.117097

H -1.719971 0.555951 0.802458

H 0.510238 1.392485 1.903535

O 1.765963 3.684707 1.744750

O 4.009482 -3.489760 0.807961

O 2.934147 -2.934660 -1.079685

H 2.830525 0.809961 2.214446

H 2.272970 -1.596560 1.411287

O -0.297504 -1.420512 0.987878

O 0.471606 -3.433142 0.313711

H -7.036869 0.573274 -1.162427

H -7.206176 -0.322422 1.143224

H -5.212593 -1.336075 2.213594

H -3.073238 -1.438575 0.990664

H -4.895965 0.459356 -2.380362

H -1.861645 -1.287494 -1.138758

H -2.672837 -0.677879 -2.571979

H -2.532664 1.639611 -1.623582

H 1.677950 3.152247 -0.253363

H 0.689248 -1.316064 -0.820178

H -0.701017 1.184138 -3.202122

H -3.050170 3.648055 -0.505011

H -3.456557 2.280534 0.521935

H -2.927902 3.798600 1.242494

H -0.411038 5.233228 1.783040

H 0.343926 5.468705 0.222001

H -1.411244 5.295575 0.325756

H 2.710898 3.500731 1.698155

H 2.934032 0.402759 -0.849625

H 4.657419 -0.759836 1.426326

H 5.256396 0.323587 0.176669

H 5.884381 -2.041710 -0.181749

H 4.024884 -0.955514 -2.369790

H 5.264840 -2.186493 -2.601610

H 5.728451 -0.544148 -2.145579

H -2.129981 -3.704630 1.391007

H -0.826395 -4.196420 2.469068

H -1.610175 -2.605649 2.685238

YYY-141e-c15-gfn0 , delta G = 3.0001 kcal/mol, population = 0.23 %

C 0.031563 1.111537 -0.926612

C -2.088050 1.071197 0.142763

C -0.994590 1.354401 1.206934

C -0.886063 2.857849 1.444881

C 0.217362 3.586424 1.209670

C 1.551428 2.955980 0.849912

C 1.508205 1.493363 1.231899

C 0.297403 0.810382 0.575963

C -2.817132 -0.270604 0.325761

C -2.166324 3.495621 1.937609

C 0.237252 5.088415 1.313171

C 2.699972 0.566816 1.087833

C 2.960717 -0.162465 -0.200257

C 2.106464 -0.802901 0.862272

C 0.609404 -0.711249 0.712366

C 4.282073 -0.843587 -0.471078

C 4.144684 -1.952397 -1.555008

C 2.883533 -2.754332 -1.289320

C 4.132767 -1.371087 -2.966391

C -3.766514 -0.554356 -0.807069

C -5.050221 -0.004099 -0.817629

C -5.909949 -0.221660 -1.888658

C -5.495142 -0.993481 -2.970581

C -4.218087 -1.545688 -2.971588

C -3.361324 -1.326888 -1.897130

C -0.080246 -2.563458 2.050054

C -0.837808 -2.959770 3.283250

H 2.088012 -3.811557 0.066174

N -1.308375 1.139689 -1.083000

O 0.843543 1.267606 -1.827583

H -1.235927 0.846432 2.140446

H 1.292175 1.490637 2.306745

O 2.574527 3.651084 1.588149

O 1.865815 -2.687487 -1.939020

O 2.988933 -3.540592 -0.196559

H 3.510283 0.684816 1.796597

H 2.500937 -1.671853 1.363116

O -0.038418 -1.226947 1.911013

O 0.415959 -3.338958 1.259124

H -3.888346 -2.149855 -3.807627

H -6.163884 -1.166014 -3.804431

H -6.904335 0.207344 -1.877494

H -5.380541 0.594632 0.023738

H -2.368157 -1.760065 -1.902755

H -3.358841 -0.224598 1.273630

H -2.088679 -1.075653 0.407935

H -2.838679 1.860376 0.128941

H 1.748084 3.076418 -0.221184

H 0.241090 -1.292225 -0.131666

H -1.720112 1.263454 -1.995221

H -1.969544 4.258172 2.690317

H -2.732351 3.971959 1.131429

H -2.818326 2.746221 2.390209

H 0.683029 5.417951 2.255000

H 0.854006 5.508471 0.515903

H -0.758564 5.519895 1.234956

H 3.424980 3.387287 1.219409

H 2.452741 0.217032 -1.074898

H 4.666090 -1.281651 0.452554

H 5.027778 -0.125836 -0.822481

H 4.992683 -2.632833 -1.445064

H 5.068399 -0.842712 -3.155494

H 3.307730 -0.670430 -3.095587

H 4.022918 -2.154935 -3.716497

H -1.899349 -2.754355 3.129461

H -0.700253 -4.020243 3.474923

H -0.507359 -2.371614 4.138924

YYY-141e-c14-gfn0 , delta G = 3.3578 kcal/mol, population = 0.13 %

C 0.625344 1.073180 -1.452205

C -1.740788 0.894011 -1.328523

C -1.177041 1.114431 0.102353

C -1.281886 2.591903 0.465688

C -0.231325 3.384933 0.731441

C 1.179090 2.843212 0.884596

C 1.094939 1.353785 1.133037

C 0.293343 0.671417 0.012437

C -2.466550 -0.446854 -1.537354

C -2.699606 3.121507 0.462964

C -0.365813 4.870060 0.937328

C 2.313276 0.498932 1.421777

C 3.115209 -0.102039 0.302264

C 1.965014 -0.872472 0.895257

C 0.638472 -0.840907 0.176814

C 4.487123 -0.689109 0.540178

C 4.880463 -1.700335 -0.574595

C 3.693782 -2.600531 -0.867365

C 5.357761 -0.997138 -1.842465

C -3.817399 -0.434447 -0.870354

C -3.995028 -0.945348 0.415663

C -5.229317 -0.864948 1.053002

C -6.308708 -0.267619 0.410594

C -6.144782 0.245180 -0.873319

C -4.909349 0.161805 -1.505384

C -0.323263 -2.827202 1.081446

C -1.418839 -3.384453 1.943822

H 2.538334 -3.816864 0.012078

N -0.538519 1.029872 -2.134433

O 1.710716 1.348668 -1.942962

H -1.734718 0.522171 0.825246

H 0.474222 1.253588 2.031088

O 1.785393 3.517251 2.004122

O 3.003466 -2.544297 -1.858421

O 3.441333 -3.467271 0.137327

H 2.766815 0.593220 2.400751

H 2.200989 -1.760120 1.458725

O -0.375709 -1.484807 1.005811

O 0.499639 -3.502633 0.499169

H -6.980473 0.708330 -1.383213

H -7.270333 -0.203952 0.903954

H -5.346487 -1.268228 2.051283

H -3.157621 -1.403405 0.923689

H -4.790319 0.562465 -2.505832

H -1.854319 -1.264097 -1.156791

H -2.584023 -0.603950 -2.611954

H -2.435520 1.689131 -1.596524

H 1.767315 3.058192 -0.014447

H 0.664533 -1.372728 -0.772811

H -0.559208 1.243165 -3.120332

H -3.416015 2.301600 0.531798

H -2.876653 3.789831 1.305347

H -2.935024 3.678852 -0.448583

H -0.343560 5.129596 1.998715

H 0.476619 5.388514 0.474636

H -1.284999 5.263032 0.507349

H 2.727477 3.314883 1.985457

H 2.964889 0.328619 -0.676402

H 4.510353 -1.189502 1.510507

H 5.248358 0.094974 0.562724

H 5.680321 -2.333339 -0.182287

H 5.628386 -1.716913 -2.615639

H 6.234612 -0.387921 -1.618038

H 4.581397 -0.349246 -2.249844

H -1.753975 -2.659237 2.681932

H -2.264519 -3.648698 1.304796

H -1.067854 -4.291474 2.431078

### Table S12. Geometry data of conformers of structure 1f.

YYY-141f-c14 , delta G = 0.0000 kcal/mol, population = 42.76 %

C -0.249862 -0.083677 -1.017805

C -2.026401 1.065515 0.058000

C -0.680451 1.469735 0.713570

C -0.173757 2.764873 0.072774

C 0.986859 2.884645 -0.595457

C 2.025999 1.774038 -0.631376

C 1.713091 0.840240 0.519144

C 0.280574 0.307313 0.388047

C -3.007347 0.320987 0.977434

C -1.126259 3.932056 0.216676

C 1.376689 4.134932 -1.335986

C 2.628083 -0.261037 1.010067

C 2.727300 -1.677801 0.506169

C 1.722918 -1.337533 1.581563

C 0.271796 -0.958570 1.306957

C 2.528340 -2.247791 -0.882436

C 3.280138 -1.464362 -1.978130

C 4.656533 -1.055949 -1.518638

C 3.324145 -2.248380 -3.292972

C -4.278645 -0.039647 0.255889

C -5.282186 0.913704 0.066340

C -6.434479 0.603485 -0.646697

C -6.600086 -0.669756 -1.185663

C -5.607793 -1.627434 -1.003100

C -4.456319 -1.313015 -0.286976

C -0.911386 -1.552932 3.309952

C -1.477707 -0.991115 4.582377

H 6.346402 -1.749527 -1.038060

N -1.558506 0.250627 -1.051336

O 0.348231 -0.617210 -1.940007

H -0.804944 1.607967 1.786780

H 1.678109 1.504001 1.390788

O 3.331224 2.345481 -0.506794

O 5.015659 0.085114 -1.301669

O 5.493620 -2.097136 -1.346138

H 3.474506 0.090332 1.585096

H 1.888259 -1.764094 2.561946

O -0.318225 -0.584620 2.581234

O -0.980254 -2.707628 2.958260

H -5.730494 -2.621454 -1.415031

H -7.497767 -0.914050 -1.739508

H -7.205150 1.352850 -0.778841

H -5.160729 1.905750 0.486624

H -3.687686 -2.063180 -0.143686

H -3.225997 0.969929 1.828843

H -2.534824 -0.580539 1.362425

H -2.542127 1.939135 -0.338064

H 1.961098 1.253899 -1.592275

H -0.320434 -1.772252 0.892620

H -2.112416 0.067190 -1.873903

H -1.747023 4.078626 -0.672477

H -1.802650 3.774150 1.058990

H -0.592517 4.864723 0.395840

H 1.882314 3.871463 -2.267115

H 0.517746 4.757496 -1.578225

H 2.086664 4.733039 -0.759044

H 3.960274 1.658030 -0.784191

H 3.591538 -2.140358 0.974304

H 1.486060 -2.270125 -1.179947

H 2.890381 -3.278747 -0.869905

H 2.740228 -0.536978 -2.141039

H 2.303021 -2.455051 -3.615572

H 3.847557 -3.197084 -3.168808

H 3.820168 -1.680016 -4.081729

H -2.249730 -0.257196 4.345050

H -1.903885 -1.791707 5.180296

H -0.697106 -0.477330 5.144307

YYY-141f-c6 , delta G = 0.8189 kcal/mol, population = 10.72 %

C 0.117172 0.827576 -1.215129

C -1.805410 1.267802 0.097492

C -0.537346 1.775144 0.840737

C -0.317846 3.248900 0.505244

C 0.762533 3.729378 -0.134908

C 2.019521 2.903234 -0.384645

C 1.937850 1.657868 0.469308

C 0.610177 0.932235 0.254087

C -2.575998 0.138759 0.799188

C -1.464313 4.154589 0.894284

C 0.845629 5.161707 -0.599797

C 3.045601 0.633389 0.592806

C 3.277964 -0.662081 -0.158469

C 2.372338 -0.651252 1.043033

C 0.864685 -0.488276 0.860038

C 2.914969 -1.182070 -1.529333

C 3.187126 -2.701478 -1.628732

C 2.518804 -3.408784 -0.468865

C 2.732306 -3.255589 -2.982077

C -3.755835 -0.318059 -0.016893

C -4.969304 0.371643 0.031653

C -6.042494 -0.018800 -0.761819

C -5.915801 -1.107368 -1.620479

C -4.711436 -1.801606 -1.677404

C -3.640530 -1.408514 -0.880670

C -0.097665 -1.770772 2.641545

C -0.659583 -1.680915 4.028992

H 0.806212 -3.550364 0.354830

N -1.234336 0.859699 -1.177632

O 0.785537 0.746689 -2.232957

H -0.643513 1.640748 1.916560

H 1.869378 2.042368 1.497557

O 3.199643 3.665694 -0.080746

O 3.101541 -3.949335 0.444826

O 1.173586 -3.339829 -0.526793

H 3.913846 0.986770 1.133397

H 2.669605 -1.189979 1.932443

O 0.255669 -0.560035 2.179872

O 0.026760 -2.800855 2.010929

H -4.605877 -2.652072 -2.339499

H -6.751164 -1.414462 -2.237027

H -6.978577 0.523140 -0.707289

H -5.075758 1.217629 0.701573

H -2.704728 -1.952623 -0.926819

H -2.906425 0.510297 1.772087

H -1.910952 -0.703213 0.976688

H -2.504242 2.085229 -0.075283

H 2.120605 2.657061 -1.441845

H 0.420172 -1.267901 0.249720

H -1.769473 0.828879 -2.031833

H -2.076098 3.692100 1.670957

H -1.105346 5.108867 1.279510

H -2.125065 4.375173 0.050314

H 1.271416 5.817370 0.165754

H 1.497095 5.235621 -1.471078

H -0.132550 5.558680 -0.868196

H 3.162635 3.913937 0.852070

H 4.260503 -1.031076 0.125765

H 3.515181 -0.679771 -2.291922

H 1.881062 -0.992350 -1.784228

H 4.255730 -2.883354 -1.501322

H 1.662691 -3.100556 -3.125202

H 2.940650 -4.323652 -3.066086

H 3.262060 -2.741876 -3.785960

H -1.389962 -0.874708 4.091748

H -1.113771 -2.628596 4.303897

H 0.149050 -1.450952 4.725827

YYY-141f-c1 , delta G = 0.8873 kcal/mol, population = 9.55 %

C 0.167498 0.725549 -1.196441

C -1.767902 1.187003 0.089830

C -0.510819 1.737519 0.818596

C -0.301743 3.199290 0.425113

C 0.785951 3.664831 -0.214190

C 2.058010 2.844145 -0.400686

C 1.968067 1.635939 0.502815

C 0.651977 0.884519 0.274004

C -2.527641 0.070679 0.823017

C -1.464701 4.107384 0.757007

C 0.865825 5.075928 -0.739498

C 3.089009 0.638132 0.720969

C 3.385329 -0.653892 -0.010079

C 2.408385 -0.654641 1.136303

C 0.904289 -0.510329 0.930514

C 3.165265 -1.100182 -1.435050

C 3.101830 -2.642575 -1.574521

C 1.881016 -3.121857 -0.822359

C 3.035989 -3.051979 -3.045928

C -3.706659 -0.410917 0.019612

C -3.584640 -1.513765 -0.827577

C -4.654058 -1.924340 -1.617696

C -5.862949 -1.237128 -1.569495

C -5.996201 -0.137375 -0.726158

C -4.924575 0.271552 0.060019

C -0.026496 -1.718932 2.780663

C -0.723515 -1.566186 4.100098

H 1.352419 -3.640282 0.926722

N -1.183227 0.743612 -1.166015

O 0.834670 0.623923 -2.213565

H -0.620527 1.646406 1.898336

H 1.853834 2.063924 1.509401

O 3.221825 3.633668 -0.101827

O 0.741375 -2.999614 -1.220893

O 2.169850 -3.633058 0.386534

H 3.908118 1.020293 1.316133

H 2.688323 -1.195044 2.029745

O 0.283075 -0.526084 2.250634

O 0.204010 -2.782785 2.241643

H -6.935629 0.399539 -0.679041

H -6.696438 -1.557531 -2.181879

H -4.542730 -2.782418 -2.268874

H -2.645285 -2.052124 -0.867826

H -5.034584 1.127495 0.716627

H -2.858490 0.463088 1.787398

H -1.856764 -0.763015 1.016150

H -2.477152 1.987834 -0.114627

H 2.187048 2.556207 -1.444247

H 0.453284 -1.312232 0.354194

H -1.712482 0.661647 -2.020448

H -2.122145 4.272431 -0.101942

H -2.075727 3.677214 1.552737

H -1.123169 5.085637 1.094182

H 1.485405 5.105584 -1.636985

H -0.116537 5.473499 -0.989186

H 1.329306 5.756298 -0.019054

H 3.156730 3.925763 0.816655

H 4.340386 -1.031166 0.348714

H 4.010527 -0.752203 -2.035885

H 2.274619 -0.666026 -1.872009

H 3.982400 -3.081956 -1.101420

H 3.913733 -2.678639 -3.575595

H 2.145362 -2.635617 -3.517216

H 3.006532 -4.137426 -3.156837

H -0.224102 -0.817512 4.713606

H -1.744617 -1.222556 3.920180

H -0.754259 -2.523134 4.613450

YYY-141f-c9 , delta G = 0.9212 kcal/mol, population = 9.02 %

C 0.099682 0.794737 -1.217744

C -1.817895 1.266156 0.093278

C -0.546776 1.766907 0.834411

C -0.325499 3.243672 0.509306

C 0.755633 3.732010 -0.122328

C 1.990870 2.893533 -0.407789

C 1.921078 1.657374 0.461155

C 0.598866 0.920795 0.247538

C -2.596747 0.146506 0.800869

C -1.471600 4.142204 0.918775

C 0.856341 5.168900 -0.561719

C 3.038743 0.644390 0.605474

C 3.296707 -0.655103 -0.132066

C 2.371040 -0.643789 1.054170

C 0.862861 -0.496137 0.858065

C 2.962387 -1.177278 -1.508741

C 3.230793 -2.697845 -1.602800

C 2.547039 -3.403044 -0.450719

C 2.792050 -3.251220 -2.961666

C -3.777962 -0.308401 -0.014266

C -3.669920 -1.408493 -0.866667

C -4.741458 -1.799234 -1.663792

C -5.939173 -1.092778 -1.618767

C -6.058681 0.005457 -0.771411

C -4.984965 0.393385 0.022507

C -0.107086 -1.789418 2.627317

C -0.680570 -1.706470 4.010324

H 0.823341 -3.550248 0.347259

N -1.250028 0.846526 -1.178758

O 0.762401 0.676497 -2.236769

H -0.652003 1.629766 1.910020

H 1.852253 2.059588 1.479465

O 3.141662 3.693246 -0.073367

O 3.118608 -3.936022 0.474436

O 1.202892 -3.341379 -0.529904

H 3.890298 1.007997 1.166886

H 2.664429 -1.170787 1.951788

O 0.242855 -0.575611 2.171493

O 0.029493 -2.817515 1.995675

H -6.989605 0.557055 -0.726168

H -6.774986 -1.397758 -2.235759

H -4.641522 -2.657229 -2.316996

H -2.739252 -1.962095 -0.903636

H -5.085796 1.247129 0.683363

H -2.926365 0.526735 1.770663

H -1.936458 -0.697793 0.985589

H -2.510266 2.088023 -0.084477

H 2.036378 2.634503 -1.469797

H 0.432727 -1.280094 0.242947

H -1.788933 0.793791 -2.029548

H -1.112175 5.087510 1.324030

H -2.136774 4.378645 0.082785

H -2.079087 3.662805 1.688594

H 1.434771 5.764660 0.149026

H 1.382099 5.230175 -1.516590

H -0.120982 5.632486 -0.681600

H 3.914851 3.256114 -0.447289

H 4.276275 -1.016164 0.172222

H 3.583834 -0.680109 -2.258232

H 1.935958 -0.978274 -1.786099

H 4.297218 -2.882388 -1.461376

H 3.333285 -2.739228 -3.759041

H 1.724800 -3.093370 -3.118382

H 2.998735 -4.319818 -3.042733

H -1.150644 -2.650536 4.270838

H 0.125972 -1.497373 4.716194

H -1.398776 -0.889874 4.076826

YYY-141f-c17 , delta G = 1.0737 kcal/mol, population = 6.97 %

C 0.108155 0.820516 -1.225876

C -1.814472 1.261472 0.086540

C -0.547520 1.776196 0.826244

C -0.335720 3.250939 0.490799

C 0.745032 3.733531 -0.145001

C 2.001558 2.905335 -0.403651

C 1.927725 1.667577 0.454266

C 0.603137 0.936858 0.241578

C -2.581353 0.134231 0.795105

C -1.487634 4.145825 0.893362

C 0.841721 5.165705 -0.601278

C 3.039844 0.647932 0.578154

C 3.276220 -0.651186 -0.166824

C 2.372182 -0.636563 1.035822

C 0.863796 -0.479930 0.854066

C 2.912655 -1.182297 -1.533411

C 3.189073 -2.701604 -1.622822

C 2.523954 -3.403335 -0.457664

C 2.734600 -3.266457 -2.971854

C -3.759788 -0.331656 -0.017806

C -3.641112 -1.427173 -0.874731

C -4.710823 -1.828807 -1.668822

C -5.917441 -1.138129 -1.616180

C -6.047554 -0.044599 -0.764350

C -4.975557 0.354319 0.026530

C -0.090381 -1.756379 2.644356

C -0.648568 -1.660668 4.032908

H 0.812670 -3.540458 0.370015

N -1.243249 0.847876 -1.186764

O 0.774247 0.735078 -2.245419

H -0.651093 1.643952 1.902530

H 1.865534 2.060695 1.477224

O 3.178181 3.629040 0.006340

O 3.109380 -3.937591 0.457982

O 1.178592 -3.337649 -0.514228

H 3.906362 1.009032 1.115565

H 2.672405 -1.168901 1.928092

O 0.256894 -0.547447 2.175067

O 0.035712 -2.790007 2.019368

H -6.985432 0.494557 -0.712993

H -6.751914 -1.451882 -2.230592

H -4.602616 -2.683213 -2.325411

H -2.703606 -1.968640 -0.917390

H -5.084742 1.204046 0.691246

H -2.912761 0.510850 1.765693

H -1.913327 -0.704262 0.977966

H -2.515637 2.076035 -0.090592

H 2.087454 2.651193 -1.463392

H 0.420649 -1.263907 0.248371

H -1.779620 0.807787 -2.039793

H -2.156029 4.369393 0.056492

H -2.090587 3.669029 1.668322

H -1.135034 5.097007 1.290690

H 1.307674 5.214512 -1.590261

H -0.131436 5.645436 -0.677942

H 1.461264 5.762496 0.073685

H 3.404161 4.264542 -0.680849

H 4.260456 -1.015126 0.118228

H 3.510453 -0.684320 -2.300948

H 1.877818 -0.997355 -1.788237

H 4.258326 -2.879568 -1.495233

H 1.664339 -3.115794 -3.114909

H 2.946140 -4.334436 -3.048776

H 3.261924 -2.756755 -3.779930

H -1.381512 -0.856558 4.093063

H -1.098684 -2.608280 4.314716

H 0.161263 -1.423425 4.725856

YYY-141f-c3 , delta G = 1.1076 kcal/mol, population = 6.58 %

C 0.158192 0.704674 -1.199075

C -1.773504 1.188766 0.085432

C -0.513916 1.733554 0.813033

C -0.301179 3.198118 0.429231

C 0.789412 3.670646 -0.198591

C 2.040527 2.835583 -0.418224

C 1.958943 1.633427 0.494284

C 0.646055 0.876535 0.268498

C -2.539504 0.079496 0.822944

C -1.463725 4.102495 0.775427

C 0.887480 5.088386 -0.695928

C 3.084857 0.641177 0.720229

C 3.390807 -0.655482 -0.001876

C 2.407826 -0.652401 1.138528

C 0.903054 -0.515823 0.928748

C 3.178058 -1.106827 -1.426462

C 3.119967 -2.649738 -1.562521

C 1.898599 -3.131589 -0.812452

C 3.059338 -3.062470 -3.033204

C -3.722792 -0.397492 0.023130

C -4.934807 0.295750 0.059756

C -6.010354 -0.108924 -0.723182

C -5.887212 -1.215270 -1.559477

C -4.684421 -1.913277 -1.603827

C -3.610992 -1.506820 -0.816931

C -0.027715 -1.727629 2.776663

C -0.731612 -1.576282 4.092438

H 1.367158 -3.641582 0.938513

N -1.191624 0.736529 -1.168393

O 0.822881 0.580382 -2.216121

H -0.623136 1.640334 1.892651

H 1.850879 2.074084 1.492971

O 3.173028 3.661381 -0.082279

O 0.760133 -3.017548 -1.216310

O 2.186027 -3.634326 0.400115

H 3.895083 1.027055 1.326157

H 2.687976 -1.183452 2.037427

O 0.277698 -0.534158 2.245982

O 0.211380 -2.791274 2.240704

H -4.580977 -2.776553 -2.249423

H -6.723866 -1.532352 -2.169270

H -6.945049 0.436433 -0.679025

H -5.036979 1.156808 0.710916

H -2.676465 -2.053773 -0.854051

H -2.866513 0.477269 1.786439

H -1.873017 -0.757398 1.018371

H -2.477603 1.993280 -0.122444

H 2.118283 2.538373 -1.468263

H 0.459327 -1.321264 0.351588

H -1.723001 0.643799 -2.020443

H -2.079107 3.654600 1.557805

H -1.121975 5.070798 1.139556

H -2.117049 4.289450 -0.082106

H 1.429418 5.113591 -1.643384

H -0.090897 5.538290 -0.851888

H 1.449437 5.717157 -0.000736

H 3.962418 3.210148 -0.401825

H 4.344862 -1.028785 0.363722

H 4.024819 -0.758905 -2.025695

H 2.286986 -0.676610 -1.867184

H 4.000597 -3.085298 -1.085991

H 3.938028 -2.689022 -3.561304

H 2.169301 -2.648908 -3.508035

H 3.032097 -4.148178 -3.141616

H -0.237907 -0.824966 4.707334

H -1.752989 -1.236408 3.907175

H -0.761180 -2.532807 4.606633

YYY-141f-c5 , delta G = 1.2224 kcal/mol, population = 5.42 %

C 0.160390 0.718218 -1.205173

C -1.771112 1.183320 0.085680

C -0.512532 1.739474 0.807031

C -0.308534 3.200758 0.409719

C 0.779428 3.664749 -0.227701

C 2.049657 2.840084 -0.422734

C 1.965906 1.640687 0.485485

C 0.650488 0.887291 0.262228

C -2.526898 0.069870 0.827120

C -1.473324 4.104566 0.750676

C 0.872940 5.076473 -0.744086

C 3.089183 0.645470 0.702555

C 3.385119 -0.650112 -0.023510

C 2.411742 -0.645936 1.125857

C 0.906898 -0.504452 0.924194

C 3.160109 -1.104947 -1.445075

C 3.097162 -2.648169 -1.576468

C 1.878846 -3.124302 -0.818207

C 3.027367 -3.065293 -3.045509

C -3.707779 -0.417947 0.030261

C -4.926980 0.262219 0.071619

C -6.000508 -0.152650 -0.708832

C -5.867974 -1.256145 -1.547400

C -4.657847 -1.941119 -1.596528

C -3.586524 -1.524608 -0.812086

C -0.015780 -1.705666 2.783215

C -0.705971 -1.547263 4.105591

H 1.356138 -3.630106 0.936572

N -1.190102 0.734072 -1.169839

O 0.822886 0.611227 -2.225377

H -0.617111 1.652520 1.887585

H 1.860799 2.078479 1.486380

O 3.209154 3.593665 -0.017445

O 0.738103 -3.005903 -1.214904

O 2.171435 -3.627718 0.392816

H 3.908356 1.033223 1.293248

H 2.694933 -1.181336 2.021292

O 0.290018 -0.515425 2.246365

O 0.212758 -2.772239 2.248203

H -4.547081 -2.802178 -2.243870

H -6.702999 -1.581236 -2.155218

H -6.940907 0.382503 -0.660885

H -5.036473 1.120998 0.724593

H -2.646246 -2.061337 -0.852894

H -2.855043 0.467029 1.790456

H -1.853988 -0.761615 1.022610

H -2.482100 1.982216 -0.120783

H 2.162870 2.542983 -1.468696

H 0.454420 -1.309221 0.353013

H -1.722618 0.644094 -2.021413

H -2.132601 4.276768 -0.105321

H -2.082299 3.664338 1.542378

H -1.134377 5.079014 1.100784

H 1.380374 5.088917 -1.713422

H -0.103952 5.533871 -0.883606

H 1.452394 5.714393 -0.070840

H 3.427452 4.220643 -0.715076

H 4.341756 -1.024700 0.333999

H 4.002976 -0.760257 -2.051438

H 2.267374 -0.673914 -1.880986

H 3.979402 -3.084444 -1.103615

H 2.998045 -4.151310 -3.150783

H 3.903519 -2.694455 -3.579638

H 2.135189 -2.651849 -3.516506

H -0.199033 -0.800575 4.715399

H -1.725800 -1.197591 3.930304

H -0.739513 -2.503242 4.620557

YYY-141f-c11 , delta G = 1.4125 kcal/mol, population = 3.93 %

C -0.246095 -0.044617 -1.024227

C -2.016272 1.016881 0.145670

C -0.658687 1.415011 0.783302

C -0.184348 2.733213 0.167146

C 0.958801 2.884128 -0.524006

C 2.024522 1.800463 -0.594574

C 1.740701 0.821364 0.523890

C 0.309272 0.278425 0.391404

C -2.968007 0.221849 1.052228

C -1.147953 3.884138 0.358330

C 1.307938 4.156838 -1.247402

C 2.676377 -0.285980 0.969264

C 2.791410 -1.684226 0.424887

C 1.782416 -1.389981 1.509350

C 0.325591 -1.023787 1.255592

C 2.564925 -2.231699 -0.959779

C 3.805293 -2.118470 -1.890112

C 4.057608 -0.669820 -2.230966

C 5.067235 -2.763424 -1.313583

C -4.249815 -0.122218 0.340498

C -4.420726 -1.371322 -0.258205

C -5.581564 -1.668940 -0.966170

C -6.590175 -0.718320 -1.084642

C -6.431457 0.530671 -0.490002

C -5.269643 0.824327 0.214803

C -0.822725 -1.730361 3.241533

C -1.384403 -1.241219 4.545668

H 3.677551 0.635660 -3.547050

N -1.562785 0.257515 -1.008290

O 0.339990 -0.499854 -1.995549

H -0.755825 1.516220 1.863445

H 1.708114 1.452712 1.419434

O 3.309162 2.416101 -0.443516

O 4.668804 0.120350 -1.539333

O 3.510452 -0.310854 -3.406851

H 3.519470 0.059054 1.553055

H 1.957663 -1.847456 2.474021

O -0.265708 -0.716081 2.548231

O -0.866510 -2.870500 2.840994

H -7.214816 1.274005 -0.572340

H -7.495047 -0.949486 -1.632450

H -5.698431 -2.644147 -1.422413

H -3.638765 -2.115718 -0.166417

H -5.153818 1.797740 0.678283

H -3.176196 0.830554 1.935373

H -2.479219 -0.690208 1.387192

H -2.553214 1.898447 -0.201550

H 1.979427 1.304969 -1.569670

H -0.255791 -1.827880 0.808728

H -2.126441 0.118761 -1.832991

H -1.785364 4.047415 -0.516018

H -1.808142 3.694876 1.206886

H -0.621400 4.817767 0.553577

H 1.817906 3.923396 -2.184156

H 0.428613 4.754886 -1.478533

H 1.999759 4.769895 -0.664340

H 3.959377 1.761925 -0.744567

H 3.649318 -2.163352 0.886400

H 1.720594 -1.761503 -1.449488

H 2.343819 -3.299984 -0.872616

H 3.538502 -2.620079 -2.821086

H 5.863768 -2.794324 -2.058843

H 4.851134 -3.787499 -1.004643

H 5.438420 -2.210674 -0.451886

H -0.604852 -0.745498 5.125150

H -2.169141 -0.507595 4.354185

H -1.792307 -2.077347 5.106564

YYY-141f-c16 , delta G = 1.7432 kcal/mol, population = 2.25 %

C 0.138637 -0.484070 -1.620360

C -2.080291 0.218433 -1.143702

C -1.114364 0.799062 -0.074386

C -0.738905 2.229783 -0.466795

C 0.508489 2.643159 -0.749139

C 1.726774 1.763406 -0.511681

C 1.304534 0.682214 0.461551

C 0.128383 -0.117953 -0.114074

C -3.173041 -0.718959 -0.603857

C -1.925641 3.166151 -0.551358

C 0.813060 4.017464 -1.279957

C 2.257698 -0.246167 1.181887

C 2.818469 -1.558966 0.698179

C 1.491958 -1.533714 1.419214

C 0.142332 -1.448198 0.711731

C 3.168829 -2.029065 -0.697939

C 4.006887 -1.009111 -1.495848

C 5.070017 -0.374313 -0.636418

C 4.596673 -1.640976 -2.760267

C -4.250863 0.064377 0.100274

C -4.190593 0.312659 1.472293

C -5.150646 1.101020 2.098856

C -6.189023 1.657245 1.359182

C -6.261164 1.416703 -0.009964

C -5.299305 0.627789 -0.630918

C -1.273624 -2.549083 2.292188

C -2.306529 -2.343813 3.363796

H 6.662719 -0.752604 0.305884

N -1.145745 -0.441889 -2.040905

O 1.084602 -0.793052 -2.328721

H -1.585539 0.800720 0.905956

H 0.868870 1.242552 1.296769

O 2.787547 2.562130 0.019287

O 5.093186 0.791722 -0.292847

O 6.030484 -1.236096 -0.250398

H 2.795214 0.217991 1.998091

H 1.453526 -2.006538 2.390909

O -0.882319 -1.377317 1.742969

O -0.851386 -3.624543 1.937289

H -7.068174 1.841823 -0.593809

H -6.937270 2.270769 1.844918

H -5.086409 1.280904 3.164906

H -3.382223 -0.108411 2.053730

H -5.362768 0.444188 -1.697614

H -2.727421 -1.456963 0.060785

H -3.603581 -1.259269 -1.450008

H -2.578044 1.021347 -1.686233

H 2.053296 1.333543 -1.463734

H -0.076295 -2.317258 0.093790

H -1.362668 -0.632061 -3.007910

H -1.685821 4.149242 -0.147074

H -2.272685 3.313607 -1.578487

H -2.768389 2.770460 0.017583

H -0.060212 4.491912 -1.723282

H 1.200338 4.670591 -0.493941

H 1.593635 3.956774 -2.041390

H 3.602507 2.040710 -0.077723

H 3.587337 -1.873709 1.398008

H 2.292592 -2.245079 -1.298818

H 3.737175 -2.957183 -0.599487

H 3.343241 -0.200423 -1.785524

H 5.157648 -0.912075 -3.347863

H 3.782146 -2.020024 -3.378562

H 5.260212 -2.470545 -2.513370

H -2.223600 -3.141288 4.098728

H -2.198401 -1.372676 3.841971

H -3.299108 -2.399283 2.911355

YYY-141f-c4 , delta G = 2.3958 kcal/mol, population = 0.75 %

C 0.654176 0.374155 -1.653649

C -1.681508 0.494180 -1.265008

C -0.920372 1.195576 -0.104230

C -0.840910 2.693545 -0.394170

C 0.303246 3.379607 -0.555207

C 1.666872 2.781146 -0.252079

C 1.448991 1.540358 0.585246

C 0.491953 0.573917 -0.121945

C -2.571265 -0.691487 -0.859603

C -2.190975 3.363924 -0.533069

C 0.327572 4.819713 -0.993779

C 2.556655 0.758324 1.266002

C 3.371128 -0.404122 0.742856

C 2.033951 -0.661930 1.390708

C 0.730742 -0.788389 0.606148

C 3.840673 -0.769574 -0.644112

C 4.160922 -2.278559 -0.789755

C 2.874768 -3.039715 -0.562598

C 4.743759 -2.579465 -2.170344

C -3.824274 -0.213390 -0.171884

C -4.942327 0.152038 -0.925163

C -6.082372 0.660446 -0.312404

C -6.122568 0.813513 1.070583

C -5.015775 0.453353 1.832192

C -3.877421 -0.053459 1.213501

C -0.493400 -2.277141 2.023897

C -1.595087 -2.386728 3.037235

H 1.849293 -3.777533 0.854452

N -0.581674 0.120348 -2.141604

O 1.666433 0.429123 -2.333403

H -1.426378 1.030364 0.844361

H 0.877090 1.905977 1.446686

O 2.417636 3.769746 0.479850

O 1.969860 -3.107659 -1.367476

O 2.789440 -3.577558 0.666288

H 2.963209 1.247114 2.142520

H 2.042117 -1.193469 2.331749

O -0.335126 -1.018698 1.580042

O 0.163589 -3.220993 1.634270

H -5.037321 0.566626 2.909081

H -7.009827 1.207667 1.549749

H -6.940330 0.934641 -0.913706

H -4.919728 0.032685 -2.002515

H -3.018491 -0.322170 1.812201

H -2.010501 -1.375090 -0.225120

H -2.835621 -1.237481 -1.767729

H -2.314580 1.209348 -1.788986

H 2.197137 2.549937 -1.180983

H 0.710483 -1.621194 -0.089936

H -0.705722 -0.006392 -3.135078

H -2.473563 3.522437 -1.578191

H -2.973167 2.754403 -0.078087

H -2.202075 4.338247 -0.044708

H 0.430465 5.495674 -0.140927

H 1.190397 4.999822 -1.637815

H -0.571213 5.096212 -1.541853

H 3.336315 3.479057 0.496735

H 4.151465 -0.627099 1.467040

H 4.764266 -0.219327 -0.848098

H 3.129125 -0.481889 -1.408373

H 4.867826 -2.571696 -0.010883

H 4.028512 -2.317478 -2.950365

H 4.993640 -3.636714 -2.275694

H 5.653347 -1.997147 -2.325753

H -1.733880 -1.453343 3.577967

H -2.525352 -2.630342 2.519367

H -1.368393 -3.197220 3.726102

YYY-141f-c2 , delta G = 2.4950 kcal/mol, population = 0.63 %

C 0.623110 0.444622 -1.648363

C -1.701512 0.552733 -1.198108

C -0.908155 1.208894 -0.031779

C -0.820878 2.713066 -0.280046

C 0.328179 3.387779 -0.458074

C 1.697821 2.780982 -0.172994

C 1.484933 1.507608 0.614990

C 0.499092 0.579875 -0.105844

C -2.589457 -0.641313 -0.814486

C -2.163515 3.404747 -0.373027

C 0.351519 4.835774 -0.876714

C 2.599673 0.687543 1.234171

C 3.386168 -0.457167 0.640029

C 2.065134 -0.731607 1.315430

C 0.741282 -0.812704 0.560990

C 3.814886 -0.769751 -0.772730

C 4.115959 -2.274395 -0.987313

C 2.828657 -3.032172 -0.756548

C 4.659175 -2.524549 -2.394007

C -3.822841 -0.179497 -0.081619

C -3.844115 -0.070759 1.309558

C -4.964427 0.421016 1.971870

C -6.084658 0.817621 1.248991

C -6.076095 0.716012 -0.139293

C -4.954082 0.222263 -0.795727

C -0.462048 -2.343557 1.951327

C -1.536244 -2.482947 2.990375

H 1.834981 -3.822872 0.654098

N -0.625122 0.206485 -2.114424

O 1.616823 0.534003 -2.350224

H -1.393314 1.016034 0.922535

H 0.931029 1.835179 1.506510

O 2.525076 3.708353 0.549460

O 1.900268 -3.056057 -1.537006

O 2.771535 -3.622871 0.449713

H 3.041827 1.140273 2.112167

H 2.093663 -1.305455 2.230912

O -0.301909 -1.069840 1.553587

O 0.172867 -3.277610 1.505910

H -6.944826 1.018636 -0.710745

H -6.958083 1.199781 1.762088

H -4.961742 0.493500 3.052504

H -2.974635 -0.368760 1.878458

H -4.956277 0.142724 -1.876977

H -2.019690 -1.351023 -0.218074

H -2.878395 -1.152360 -1.735336

H -2.343117 1.289507 -1.680074

H 2.244587 2.594306 -1.097758

H 0.691876 -1.616546 -0.166894

H -0.773837 0.125296 -3.109289

H -2.944723 2.798601 0.088271

H -2.147345 4.370641 0.132143

H -2.466834 3.588070 -1.408273

H 1.247697 5.041490 -1.463675

H -0.519300 5.099237 -1.475161

H 0.380678 5.510846 -0.016126

H 2.079386 3.920646 1.379664

H 4.184396 -0.716006 1.332055

H 4.737367 -0.218744 -0.978232

H 3.087222 -0.444870 -1.505941

H 4.840216 -2.605157 -0.240093

H 3.926354 -2.224645 -3.143479

H 4.895795 -3.578846 -2.548532

H 5.569916 -1.944252 -2.549919

H -1.655345 -1.568491 3.566918

H -2.481524 -2.704942 2.490297

H -1.295063 -3.317865 3.644338

YYY-141f-c12 , delta G = 2.5671 kcal/mol, population = 0.56 %

C 0.137405 -0.441514 -1.640357

C -2.079039 0.211059 -1.095749

C -1.093497 0.775362 -0.034959

C -0.728710 2.211214 -0.414355

C 0.514012 2.630259 -0.707692

C 1.743243 1.763698 -0.476778

C 1.335406 0.653415 0.468123

C 0.153579 -0.132660 -0.119910

C -3.158485 -0.743204 -0.559733

C -1.917342 3.146566 -0.475838

C 0.803156 4.010502 -1.232965

C 2.298953 -0.291838 1.157292

C 2.861098 -1.590599 0.644816

C 1.531620 -1.586059 1.361735

C 0.177696 -1.485010 0.667291

C 3.187910 -2.052746 -0.751011

C 4.599938 -1.624110 -1.239907

C 4.638651 -0.129725 -1.449617

C 5.731602 -2.066475 -0.310398

C -4.227727 0.019292 0.179673

C -4.138935 0.248479 1.553519

C -5.092359 1.017779 2.212938

C -6.152755 1.573854 1.504995

C -6.253435 1.352362 0.134470

C -5.297965 0.582414 -0.519426

C -1.211011 -2.626531 2.244592

C -2.222803 -2.448632 3.341237

H 4.404411 1.192143 -2.783597

N -1.158419 -0.421579 -2.027544

O 1.071678 -0.684482 -2.389427

H -1.542495 0.757977 0.955424

H 0.904663 1.187467 1.323021

O 2.773321 2.582501 0.089310

O 4.846816 0.697381 -0.584346

O 4.397207 0.222479 -2.725796

H 2.833723 0.153029 1.986017

H 1.498597 -2.084231 2.320864

O -0.836703 -1.441986 1.712272

O -0.790865 -3.692585 1.859597

H -7.077728 1.777368 -0.424788

H -6.895948 2.172469 2.016409

H -5.005860 1.182918 3.279796

H -3.313732 -0.173034 2.110349

H -5.383777 0.413281 -1.586956

H -2.700740 -1.496080 0.078865

H -3.600849 -1.263806 -1.412016

H -2.591639 1.022918 -1.610464

H 2.093832 1.356976 -1.430986

H -0.048300 -2.336995 0.028873

H -1.388770 -0.567715 -2.999163

H -2.275877 3.305009 -1.497356

H -2.753076 2.743178 0.098028

H -1.673811 4.125368 -0.063422

H 1.591110 3.963719 -1.987780

H -0.073314 4.471791 -1.684079

H 1.172201 4.668923 -0.442657

H 3.601162 2.086188 -0.008892

H 3.615909 -1.937052 1.344005

H 2.454318 -1.716354 -1.473276

H 3.180088 -3.147090 -0.758211

H 4.738465 -2.083922 -2.219228

H 6.705342 -1.879459 -0.766027

H 5.649288 -3.136205 -0.111123

H 5.699260 -1.532102 0.637817

H -2.096632 -1.494719 3.848971

H -3.224268 -2.479482 2.906471

H -2.133477 -3.270566 4.047811

YYY-141f-c10 , delta G = 2.6305 kcal/mol, population = 0.50 %

C 0.616383 0.493927 -1.693865

C -1.716236 0.598179 -1.284973

C -0.947664 1.240254 -0.093839

C -0.879122 2.751295 -0.308644

C 0.258742 3.449543 -0.460833

C 1.630500 2.842232 -0.222270

C 1.438907 1.561413 0.559663

C 0.464820 0.624987 -0.156619

C -2.610194 -0.601111 -0.930842

C -2.234217 3.421441 -0.386905

C 0.269155 4.909857 -0.827370

C 2.569526 0.747764 1.153810

C 3.337149 -0.428102 0.586962

C 2.030750 -0.664031 1.299520

C 0.722740 -0.766619 0.514864

C 3.685212 -0.891805 -0.807551

C 4.282012 -2.318055 -0.779567

C 3.370323 -3.223020 0.020726

C 4.522586 -2.843393 -2.197769

C -3.857555 -0.142780 -0.219751

C -4.962852 0.289264 -0.956520

C -6.093174 0.791966 -0.322045

C -6.136502 0.871512 1.067039

C -5.043484 0.442158 1.812403

C -3.914575 -0.059139 1.172039

C -0.522932 -2.350085 1.805948

C -1.599163 -2.515827 2.838266

H 1.537534 -3.733598 0.094125

N -0.623353 0.264508 -2.187491

O 1.630480 0.572462 -2.368333

H -1.446806 1.024178 0.848281

H 0.899441 1.884800 1.458401

O 2.397883 3.796010 0.538112

O 3.642915 -3.703703 1.098518

O 2.175288 -3.408183 -0.572824

H 3.041209 1.212858 2.010378

H 2.041965 -1.196295 2.240745

O -0.337217 -1.061201 1.470579

O 0.090611 -3.274581 1.313645

H -5.068027 0.497666 2.893701

H -7.015670 1.262348 1.563485

H -6.940678 1.119875 -0.911106

H -4.937005 0.229393 -2.038787

H -3.066397 -0.381763 1.759755

H -2.052826 -1.315613 -0.327983

H -2.880610 -1.104715 -1.861496

H -2.350042 1.340224 -1.768897

H 2.137711 2.658859 -1.174300

H 0.729521 -1.564314 -0.221399

H -0.755335 0.202152 -3.186212

H -3.007422 2.780673 0.039026

H -2.242422 4.362936 0.162137

H -2.530808 3.644374 -1.416207

H 1.108098 5.121544 -1.493197

H -0.648466 5.214120 -1.327305

H 0.405571 5.541972 0.053800

H 3.316041 3.503395 0.524405

H 4.162951 -0.638683 1.262369

H 4.428853 -0.223724 -1.250094

H 2.831005 -0.877135 -1.470958

H 5.225787 -2.301665 -0.231694

H 3.588409 -2.887236 -2.758170

H 4.964631 -3.841220 -2.184236

H 5.206190 -2.176514 -2.725942

H -2.539852 -2.739652 2.330213

H -1.352780 -3.360243 3.478264

H -1.728080 -1.613053 3.430413

YYY-141f-c7 , delta G = 2.8156 kcal/mol, population = 0.37 %

C 0.623541 0.509295 -1.687966

C -1.709906 0.594222 -1.282302

C -0.945770 1.243271 -0.091473

C -0.877116 2.751610 -0.319844

C 0.263474 3.443610 -0.484836

C 1.639612 2.850937 -0.205255

C 1.443928 1.566255 0.568740

C 0.468581 0.632323 -0.150164

C -2.600365 -0.607171 -0.926968

C -2.229135 3.425108 -0.412663

C 0.271474 4.896693 -0.885561

C 2.573030 0.749997 1.158749

C 3.336394 -0.422326 0.583640

C 2.033522 -0.661815 1.302324

C 0.724845 -0.760616 0.519025

C 3.678488 -0.883066 -0.813245

C 4.278119 -2.308117 -0.789297

C 3.369781 -3.216055 0.011281

C 4.516628 -2.830788 -2.208850

C -3.849319 -0.150387 -0.217676

C -4.955063 0.277716 -0.956103

C -6.087059 0.779049 -0.323500

C -6.131722 0.861168 1.065390

C -5.038260 0.435824 1.812436

C -3.907603 -0.063935 1.173912

C -0.520748 -2.342723 1.811499

C -1.593546 -2.508798 2.847486

H 1.538330 -3.729792 0.088771

N -0.613895 0.264375 -2.182756

O 1.636008 0.606668 -2.360980

H -1.446828 1.031370 0.850576

H 0.896560 1.878702 1.470009

O 2.456124 3.778703 0.529131

O 3.645525 -3.700304 1.086685

O 2.172651 -3.400385 -0.579054

H 3.055380 1.216930 2.007498

H 2.047283 -1.201254 2.239479

O -0.334836 -1.053571 1.476635

O 0.090082 -3.267231 1.316194

H -5.064148 0.492728 2.893645

H -7.012374 1.250490 1.560385

H -6.934926 1.103588 -0.913878

H -4.928342 0.215500 -2.038201

H -3.059326 -0.384000 1.762836

H -2.041920 -1.319746 -0.323092

H -2.868700 -1.112283 -1.857355

H -2.346529 1.332258 -1.768540

H 2.189840 2.681801 -1.131175

H 0.727728 -1.557424 -0.218136

H -0.743888 0.210162 -3.182234

H -2.521673 3.630257 -1.446813

H -3.006510 2.796070 0.023160

H -2.233605 4.377861 0.116997

H 0.324620 5.560521 -0.017695

H 1.151437 5.111386 -1.494020

H -0.614767 5.166958 -1.457394

H 2.010662 3.972431 1.364100

H 4.166040 -0.633936 1.253913

H 4.417468 -0.211694 -1.257896

H 2.821348 -0.871070 -1.472734

H 5.223044 -2.290915 -0.243498

H 4.960141 -3.828017 -2.197920

H 5.198249 -2.161889 -2.736934

H 3.581491 -2.874956 -2.767669

H -1.723139 -1.605254 3.438298

H -2.535124 -2.736474 2.342863

H -1.342925 -3.351193 3.488526

### Table S13. Geometry data of conformers of structure 1g.

YYY-141g-c5 , delta G = 0.0000 kcal/mol, population = 64.42 %

C 0.199316 1.353815 2.297855

C 2.226848 1.267737 1.057624

C 1.057304 0.895596 0.101872

C 0.695951 2.098310 -0.755415

C -0.479740 2.742681 -0.699142

C -1.650336 2.236137 0.121448

C -1.462465 0.762508 0.420001

C -0.086236 0.505540 1.037417

C 3.217846 0.120288 1.324281

C 1.813282 2.558863 -1.666426

C -0.742338 4.018515 -1.455385

C -2.440109 0.048088 1.348142

C -2.807307 -1.378283 1.031391

C -1.656341 -1.089435 1.972631

C -0.204275 -0.960975 1.525734

C -2.747195 -2.025852 -0.334543

C -4.031255 -1.777941 -1.160931

C -4.204011 -0.283724 -1.310901

C -3.975543 -2.509028 -2.503766

C 4.011504 -0.219245 0.090380

C 3.724723 -1.359816 -0.659421

C 4.414253 -1.634167 -1.836402

C 5.405310 -0.766163 -2.282150

C 5.706109 0.372194 -1.538999

C 5.014612 0.640642 -0.363504

C 0.334139 -3.134361 0.697812

C 0.608343 -3.926559 -0.549437

H -3.227163 1.216708 -1.922170

N 1.509909 1.681630 2.255370

O -0.584282 1.648664 3.187312

H 1.349973 0.069172 -0.544198

H -1.450155 0.269538 -0.552553

O -2.852360 2.470277 -0.657230

O -4.999962 0.384250 -0.682306

O -3.314834 0.261188 -2.157814

H -3.109447 0.643792 1.952733

H -1.801708 -1.354833 3.009598

O 0.164563 -1.826436 0.420720

O 0.252289 -3.595844 1.811986

H 6.481209 1.049801 -1.874835

H 5.942797 -0.976524 -3.198116

H 4.177008 -2.525745 -2.403594

H 2.953521 -2.037552 -0.317842

H 5.257482 1.528625 0.209015

H 2.677487 -0.757163 1.681271

H 3.888576 0.432002 2.129034

H 2.788332 2.115366 0.666853

H -1.741949 2.812497 1.048340

H 0.464827 -1.185038 2.353419

H 1.939280 2.204596 3.003802

H 1.441741 2.801760 -2.661954

H 2.320612 3.449321 -1.283564

H 2.568712 1.779831 -1.773257

H -1.325821 3.836347 -2.361401

H -1.330517 4.704360 -0.842253

H 0.179807 4.521070 -1.739188

H -3.621920 2.275444 -0.104462

H -3.679138 -1.712379 1.586432

H -2.638243 -3.106732 -0.211857

H -1.888544 -1.688571 -0.911224

H -4.892645 -2.124337 -0.587721

H -3.888629 -3.583717 -2.334808

H -3.116135 -2.182713 -3.090012

H -4.877743 -2.331274 -3.091227

H -0.341529 -4.313748 -0.925401

H 1.053682 -3.309626 -1.326834

H 1.250483 -4.771861 -0.312211

YYY-141g-c2 , delta G = 0.9821 kcal/mol, population = 12.26 %

C 0.202924 1.357102 2.294572

C 2.231626 1.246343 1.058623

C 1.055448 0.904216 0.099641

C 0.700502 2.132341 -0.722574

C -0.472382 2.780297 -0.644354

C -1.670040 2.243214 0.126089

C -1.467426 0.769740 0.421307

C -0.088291 0.511322 1.032914

C 3.207851 0.082541 1.307656

C 1.819459 2.621260 -1.616250

C -0.715653 4.088556 -1.353551

C -2.437593 0.054167 1.355468

C -2.810062 -1.369676 1.045300

C -1.651709 -1.081657 1.979490

C -0.202895 -0.954876 1.522811

C -2.757781 -2.028339 -0.315141

C -4.028681 -1.763230 -1.164243

C -4.066012 -0.278662 -1.431792

C -4.002596 -2.569750 -2.460392

C 3.998825 -0.244707 0.068559

C 5.009808 0.613282 -0.371376

C 5.699757 0.357242 -1.550538

C 5.389487 -0.766615 -2.311648

C 4.390352 -1.632556 -1.880188

C 3.702349 -1.370591 -0.699459

C 0.332375 -3.129457 0.695403

C 0.590844 -3.926309 -0.552341

H -4.531929 1.335184 -0.563148

N 1.521223 1.656372 2.261695

O -0.580361 1.669183 3.177948

H 1.336800 0.088644 -0.565134

H -1.455324 0.275394 -0.553025

O -2.883427 2.467463 -0.625398

O -3.399144 0.271011 -2.289837

O -4.827580 0.399710 -0.563795

H -3.105857 0.655823 1.954069

H -1.790378 -1.345988 3.017723

O 0.158292 -1.822691 0.416455

O 0.264936 -3.587887 1.811889

H 4.145325 -2.512766 -2.461657

H 5.925660 -0.967306 -3.230559

H 6.481048 1.033171 -1.875253

H 5.260326 1.489896 0.215216

H 2.924586 -2.046485 -0.369465

H 2.657571 -0.794665 1.649084

H 3.881081 0.372957 2.118238

H 2.804993 2.090606 0.678205

H -1.817445 2.807416 1.049569

H 0.471575 -1.178475 2.346093

H 1.953977 2.180423 3.007322

H 2.337091 3.488796 -1.195875

H 2.566769 1.840269 -1.761303

H 1.446329 2.913479 -2.597924

H -1.208683 3.944900 -2.319232

H -1.376628 4.721313 -0.758903

H 0.210701 4.632234 -1.530274

H -2.794761 1.999956 -1.473706

H -3.679690 -1.699546 1.606325

H -2.675204 -3.110154 -0.182492

H -1.887723 -1.718015 -0.891032

H -4.907067 -2.024125 -0.571580

H -3.131940 -2.303227 -3.060173

H -4.896480 -2.388345 -3.059063

H -3.954808 -3.635781 -2.233777

H 0.996158 -3.306221 -1.348718

H 1.262239 -4.752007 -0.326578

H -0.358980 -4.343853 -0.894408

YYY-141g-c6 , delta G = 1.0153 kcal/mol, population = 11.59 %

C 0.796665 1.080944 1.407407

C 2.192600 0.841342 -0.500894

C 0.717494 0.643226 -0.949926

C 0.190625 1.922751 -1.581878

C -0.812007 2.659449 -1.078801

C -1.645125 2.207432 0.104892

C -1.499353 0.711467 0.289812

C -0.023913 0.314120 0.345683

C 3.044522 -0.434235 -0.622421

C 0.938040 2.345874 -2.827323

C -1.204702 3.996103 -1.651211

C -2.130097 0.036196 1.503326

C -2.712558 -1.341913 1.324179

C -1.278259 -1.185374 1.785207

C -0.082788 -1.155157 0.839011

C -3.204080 -1.930808 0.020332

C -4.671442 -1.545431 -0.283825

C -4.753570 -0.037084 -0.334465

C -5.164443 -2.213005 -1.569233

C 4.418212 -0.255721 -0.033852

C 4.683991 -0.636344 1.282643

C 5.935481 -0.414861 1.849275

C 6.941458 0.192090 1.104232

C 6.688372 0.574678 -0.210320

C 5.435997 0.352726 -0.772266

C -0.020681 -3.332644 -0.135404

C -0.244839 -4.094088 -1.410249

H -3.940492 1.412983 -1.236914

N 2.020332 1.289654 0.874152

O 0.437002 1.405609 2.528947

H 0.654890 -0.173346 -1.670056

H -1.891624 0.266899 -0.625237

O -3.020746 2.575124 -0.175481

O -5.200220 0.658574 0.555082

O -4.191538 0.479416 -1.440369

H -2.480343 0.655177 2.317569

H -1.060292 -1.482953 2.800621

O -0.209356 -2.010928 -0.324164

O 0.280126 -3.816148 0.930801

H 7.467878 1.042754 -0.798671

H 7.916712 0.362279 1.542574

H 6.125029 -0.719169 2.871129

H 3.903995 -1.110442 1.866336

H 5.246242 0.650386 -1.797467

H 3.116311 -0.680893 -1.684384

H 2.532466 -1.263174 -0.132644

H 2.679187 1.630671 -1.072775

H -1.338504 2.743025 1.009569

H 0.818526 -1.455431 1.368754

H 2.764149 1.701854 1.416596

H 0.259113 2.709495 -3.598284

H 1.660044 3.143068 -2.627590

H 1.492812 1.505079 -3.246578

H -2.095095 3.917146 -2.280045

H -1.455880 4.689224 -0.845842

H -0.406616 4.438495 -2.243351

H -3.552515 2.405587 0.614679

H -3.352074 -1.630968 2.153043

H -3.155889 -3.021427 0.080663

H -2.580324 -1.637991 -0.821510

H -5.297283 -1.852686 0.555432

H -4.547787 -1.922324 -2.420098

H -6.198454 -1.941425 -1.787737

H -5.115139 -3.298037 -1.462965

H -1.300970 -4.038190 -1.681138

H 0.327063 -3.648916 -2.224429

H 0.040301 -5.133379 -1.273337

YYY-141g-c3 , delta G = 1.0793 kcal/mol, population = 10.40 %

C 0.803658 1.192355 1.311126

C 2.190060 0.793147 -0.577523

C 0.709302 0.586514 -1.004455

C 0.197896 1.838224 -1.699261

C -0.788986 2.615315 -1.226013

C -1.662402 2.225560 -0.042789

C -1.503486 0.745640 0.243548

C -0.026790 0.351664 0.313961

C 3.027094 -0.497148 -0.619305

C 0.943433 2.195926 -2.965394

C -1.143853 3.934885 -1.864539

C -2.116614 0.162178 1.511692

C -2.701750 -1.222521 1.450675

C -1.260194 -1.033428 1.877774

C -0.078657 -1.076721 0.915354

C -3.212006 -1.918282 0.208827

C -4.677104 -1.540576 -0.131588

C -4.680262 -0.067962 -0.460885

C -5.208729 -2.379031 -1.291870

C 4.408319 -0.292578 -0.057397

C 5.423100 0.257418 -0.844254

C 6.682923 0.505357 -0.310581

C 6.946729 0.207902 1.023751

C 5.943880 -0.340322 1.816978

C 4.684867 -0.588058 1.278545

C -0.025855 -3.320742 0.104057

C -0.270413 -4.175910 -1.106052

H -4.665543 1.610292 0.410681

N 2.031358 1.336199 0.764682

O 0.449417 1.613274 2.401679

H 0.629398 -0.270690 -1.673987

H -1.902642 0.232562 -0.634830

O -3.040400 2.564611 -0.313726

O -4.327702 0.386601 -1.534227

O -5.013624 0.708968 0.577864

H -2.462141 0.846568 2.272914

H -1.027647 -1.252749 2.909820

O -0.221443 -2.017181 -0.179282

O 0.294673 -3.723306 1.197892

H 6.141680 -0.578296 2.854762

H 7.927763 0.398455 1.440312

H 7.459826 0.927273 -0.936146

H 5.224939 0.488803 -1.884865

H 3.907217 -1.016010 1.899791

H 3.085958 -0.815579 -1.662858

H 2.512562 -1.285931 -0.069829

H 2.682811 1.535880 -1.203949

H -1.405483 2.819929 0.836634

H 0.829861 -1.338168 1.453177

H 2.778847 1.788496 1.268581

H 0.263774 2.541394 -3.744455

H 1.679811 2.988960 -2.803831

H 1.480785 1.329584 -3.354688

H -1.528987 4.625467 -1.113387

H -0.282560 4.396637 -2.344777

H -1.927976 3.825687 -2.619572

H -3.313425 2.069797 -1.105192

H -3.329404 -1.443063 2.309219

H -3.182785 -2.999169 0.369321

H -2.589436 -1.716565 -0.660788

H -5.292038 -1.690827 0.757350

H -5.174739 -3.437711 -1.030729

H -4.605589 -2.223339 -2.186874

H -6.241656 -2.119702 -1.528952

H 0.277280 -3.786703 -1.964284

H 0.028904 -5.199714 -0.899938

H -1.333216 -4.150254 -1.354775

YYY-141g-c4 , delta G = 2.6600 kcal/mol, population = 0.72 %

C 0.767877 1.299312 1.869618

C 2.273924 2.115683 0.208647

C 1.043153 1.486840 -0.524063

C 0.215327 2.640032 -1.070181

C -0.965061 3.026578 -0.564033

C -1.736080 2.189053 0.445795

C -1.242353 0.758242 0.369892

C 0.285220 0.679129 0.538537

C 3.656498 1.506079 -0.088945

C 0.893402 3.417748 -2.177343

C -1.644599 4.301837 -0.994958

C -1.782261 -0.277225 1.347000

C -2.051513 -1.669214 0.839617

C -0.704776 -1.336532 1.446278

C 0.502223 -0.853860 0.645357

C -2.356138 -2.056150 -0.589838

C -3.860278 -1.922850 -0.941236

C -4.195167 -0.456945 -0.828873

C -4.151619 -2.463993 -2.339013

C 3.844713 0.052050 0.245705

C 4.041478 -0.357131 1.567230

C 4.222358 -1.699951 1.879084

C 4.223595 -2.657081 0.869654

C 4.042519 -2.261320 -0.450688

C 3.849764 -0.918831 -0.757115

C 0.816590 -2.678232 -0.867514

C 0.899167 -3.034351 -2.325075

H -4.614001 0.838091 0.484993

N 1.875099 2.024073 1.600903

O 0.269722 1.161604 2.976933

H 1.375561 0.847601 -1.340984

H -1.441422 0.435802 -0.654857

O -3.152438 2.277488 0.192654

O -3.918688 0.367490 -1.681763

O -4.737572 -0.123482 0.349407

H -2.297371 0.072187 2.229629

H -0.472846 -1.803926 2.391955

O 0.577160 -1.363103 -0.711297

O 0.940963 -3.452577 0.051053

H 4.044618 -3.000730 -1.241648

H 4.360304 -3.703358 1.110011

H 4.366310 -1.998058 2.910017

H 4.056674 0.381932 2.357930

H 3.709101 -0.617538 -1.788458

H 4.394286 2.099709 0.458717

H 3.856013 1.670083 -1.150246

H 2.350012 3.169849 -0.060472

H -1.618328 2.588000 1.456529

H 1.429655 -1.120401 1.144688

H 2.355279 2.512995 2.341640

H 1.711879 2.842142 -2.611844

H 0.194337 3.654959 -2.980018

H 1.313292 4.364947 -1.826517

H -0.929749 5.041901 -1.351023

H -2.370150 4.129038 -1.795339

H -2.199508 4.733387 -0.160870

H -3.311770 1.952398 -0.710538

H -2.648778 -2.251028 1.535856

H -2.076135 -3.101182 -0.744712

H -1.785308 -1.467367 -1.305879

H -4.444479 -2.464076 -0.195292

H -3.874925 -3.517795 -2.393296

H -3.580789 -1.916910 -3.089968

H -5.210662 -2.376431 -2.586452

H -0.033027 -2.770364 -2.826509

H 1.699681 -2.464737 -2.798845

H 1.088442 -4.098686 -2.433138

YYY-141g-c7 , delta G = 2.7479 kcal/mol, population = 0.62 %

C 0.771957 1.265970 1.890559

C 2.259878 2.127949 0.236135

C 1.041806 1.484233 -0.503954

C 0.201382 2.622478 -1.064966

C -0.986228 2.999302 -0.569110

C -1.713873 2.176097 0.475866

C -1.235781 0.742317 0.384842

C 0.290446 0.661514 0.552656

C 3.650201 1.535818 -0.061181

C 0.877618 3.388819 -2.181382

C -1.699750 4.247160 -1.017752

C -1.780985 -0.305837 1.346324

C -2.049365 -1.690270 0.812036

C -0.704503 -1.368289 1.427305

C 0.505463 -0.873261 0.637276

C -2.350767 -2.046735 -0.626600

C -3.854673 -1.920359 -0.964372

C -4.265709 -0.492316 -0.691458

C -4.137337 -2.357839 -2.402955

C 3.843875 0.077996 0.253608

C 4.038525 -0.349121 1.569731

C 4.219785 -1.695987 1.863503

C 4.223249 -2.639205 0.841054

C 4.044827 -2.225416 -0.474123

C 3.851963 -0.878996 -0.762535

C 0.824857 -2.673922 -0.901324

C 0.908282 -3.008226 -2.363814

H -3.732971 1.260496 -1.160361

N 1.860162 2.020066 1.627145

O 0.283701 1.097629 2.998287

H 1.388359 0.849500 -1.318312

H -1.439319 0.434771 -0.641108

O -3.132908 2.286516 0.202747

O -4.902792 -0.130907 0.277224

O -3.769769 0.372517 -1.592408

H -2.288933 0.023774 2.241799

H -0.475329 -1.849687 2.366514

O 0.583219 -1.361361 -0.726160

O 0.950644 -3.460631 0.006371

H 4.049177 -2.953904 -1.275124

H 4.359792 -3.688623 1.067300

H 4.361958 -2.008123 2.890504

H 4.051775 0.378952 2.370600

H 3.713155 -0.563894 -1.789987

H 4.379538 2.126839 0.500334

H 3.855497 1.716582 -1.118623

H 2.321719 3.184953 -0.025541

H -1.541234 2.582639 1.479087

H 1.430997 -1.147842 1.135745

H 2.340700 2.499171 2.374111

H 1.304132 4.336159 -1.839607

H 1.692066 2.804776 -2.611986

H 0.179200 3.621069 -2.985464

H -1.031737 4.941480 -1.522555

H -2.527207 4.016129 -1.693192

H -2.137817 4.758781 -0.158214

H -3.618683 1.822289 0.898665

H -2.648244 -2.284676 1.496040

H -2.057610 -3.084411 -0.806274

H -1.786376 -1.433624 -1.326053

H -4.427242 -2.538257 -0.270984

H -5.197522 -2.268685 -2.645373

H -3.846504 -3.401553 -2.534027

H -3.572500 -1.752007 -3.112000

H 1.099020 -4.070541 -2.487822

H -0.024355 -2.737892 -2.860996

H 1.708104 -2.430380 -2.828792

### Table S14. Geometry data of conformers of structure 1h.

YYY-141h-c11-gfn0 , delta G = 0.0000 kcal/mol, population = 37.44 %

C 0.036891 1.337946 2.125856

C 2.264309 1.020804 1.354703

C 1.343429 1.212189 0.115758

C 1.459538 2.642457 -0.388647

C 0.454512 3.532317 -0.368577

C -0.979228 3.155529 -0.018322

C -1.150958 1.658688 -0.138971

C -0.061593 0.917607 0.640656

C 2.903382 -0.375657 1.464083

C 2.845078 3.015751 -0.868438

C 0.656250 4.984572 -0.718755

C -2.454957 1.010224 0.307144

C -2.923040 -0.162073 -0.499716

C -2.095658 -0.393532 0.746688

C -0.581743 -0.540905 0.645756

C -4.385053 -0.542548 -0.478706

C -4.673844 -1.983757 -0.943372

C -3.891867 -2.944230 -0.074063

C -6.170348 -2.297420 -0.880544

C 3.905742 -0.614491 0.365411

C 5.112820 0.089071 0.340856

C 6.013706 -0.076915 -0.704460

C 5.722952 -0.954289 -1.745900

C 4.529882 -1.668560 -1.727437

C 3.631469 -1.497153 -0.678885

C -0.047609 -2.494429 -0.637533

C 0.292490 -2.981015 -2.016160

H -2.323218 -4.015270 -0.103469

N 1.349156 1.306285 2.449857

O -0.880919 1.608872 2.884154

H 1.634421 0.522112 -0.675412

H -0.981879 1.421088 -1.196390

O -1.921169 3.852188 -0.850878

O -4.112549 -3.160359 1.094896

O -2.877054 -3.530899 -0.741391

H -3.170865 1.594298 0.868250

H -2.579149 -0.853395 1.595945

O -0.156269 -1.157156 -0.601047

O -0.226276 -3.209923 0.324901

H 4.297462 -2.360321 -2.527618

H 6.423908 -1.083903 -2.560749

H 6.944275 0.476981 -0.706117

H 5.350508 0.773691 1.147087

H 2.708318 -2.061121 -0.667127

H 2.125884 -1.140153 1.438849

H 3.389959 -0.449198 2.440292

H 3.064127 1.759964 1.356168

H -1.224350 3.491253 0.992194

H -0.189125 -1.138547 1.463579

H 1.656694 1.501521 3.390707

H 3.403108 3.586264 -0.119728

H 3.429534 2.122227 -1.091542

H 2.805800 3.623817 -1.772299

H 0.010066 5.611616 -0.102083

H 1.686588 5.300938 -0.566300

H 0.391357 5.196056 -1.758791

H -1.769306 3.578744 -1.764532

H -2.422771 -0.321857 -1.449509

H -4.944887 0.134084 -1.130337

H -4.783444 -0.413162 0.531022

H -4.316251 -2.097955 -1.968045

H -6.724591 -1.587620 -1.496491

H -6.533460 -2.222235 0.145018

H -6.381828 -3.303605 -1.246001

H 0.860838 -2.239495 -2.573654

H 0.840306 -3.918284 -1.949704

H -0.645391 -3.165826 -2.544861

YYY-141h-c4 , delta G = 0.6357 kcal/mol, population = 12.79 %

C 0.741820 1.070999 1.915326

C 2.463888 2.019411 0.562547

C 1.311637 1.552805 -0.386531

C 0.607340 2.804986 -0.897403

C -0.618124 3.200709 -0.517913

C -1.530465 2.308560 0.308548

C -1.084824 0.879504 0.129422

C 0.394891 0.664142 0.467584

C 3.844276 1.373091 0.343316

C 1.466632 3.652915 -1.811829

C -1.198513 4.536580 -0.900059

C -1.806404 -0.226991 0.863832

C -2.040064 -1.476474 0.065727

C -0.838180 -1.374791 0.969872

C 0.495064 -0.888993 0.389631

C -3.251444 -2.308991 0.406909

C -4.494414 -1.851252 -0.421309

C -4.446208 -0.342083 -0.560746

C -4.547150 -2.526920 -1.788839

C 3.903837 -0.125767 0.447855

C 3.918803 -0.757206 1.694246

C 3.967234 -2.143455 1.790858

C 4.012869 -2.922638 0.639245

C 4.012538 -2.305861 -0.606694

C 3.954523 -0.919803 -0.699070

C 0.809973 -2.520194 -1.326569

C 0.970835 -2.694008 -2.810719

H -4.445419 1.221963 0.499404

N 1.898817 1.765900 1.875251

O 0.112037 0.808759 2.929364

H 1.722828 0.997800 -1.228879

H -1.170462 0.685922 -0.945700

O -2.915061 2.458636 -0.077044

O -4.087144 0.248043 -1.560607

O -4.781648 0.302843 0.569230

H -2.464645 0.011443 1.684653

H -0.794583 -1.992014 1.855259

O 0.666492 -1.219323 -1.011575

O 0.799803 -3.413518 -0.513679

H 4.051328 -2.905503 -1.507489

H 4.043378 -4.001919 0.712008

H 3.970748 -2.615048 2.765538

H 3.893816 -0.159615 2.596502

H 3.953694 -0.445970 -1.673610

H 4.530912 1.817960 1.069373

H 4.189806 1.688148 -0.643903

H 2.611879 3.094089 0.450426

H -1.514428 2.604832 1.360684

H 1.341810 -1.295900 0.934590

H 2.308933 2.123130 2.725259

H 0.897895 4.020668 -2.666151

H 1.884207 4.526087 -1.302150

H 2.305601 3.073427 -2.199382

H -1.890814 4.451273 -1.742247

H -1.775095 4.946212 -0.068261

H -0.427764 5.256317 -1.168462

H -3.040768 2.035722 -0.941892

H -1.838210 -1.393776 -0.996390

H -3.466882 -2.212807 1.472354

H -3.081953 -3.368957 0.203604

H -5.391251 -2.102445 0.148536

H -4.632875 -3.606853 -1.662587

H -3.645597 -2.318026 -2.365651

H -5.401424 -2.179802 -2.371238

H 1.197858 -3.732279 -3.036333

H 0.046995 -2.403651 -3.314559

H 1.765069 -2.045561 -3.180908

YYY-141h-c1 , delta G = 0.7812 kcal/mol, population = 10.00 %

C 0.199639 1.070331 2.299164

C 2.336624 1.110687 1.255589

C 1.263868 0.885256 0.152863

C 0.997586 2.194288 -0.580259

C -0.171208 2.856537 -0.562027

C -1.428094 2.277967 0.069209

C -1.264747 0.785602 0.206315

C 0.030645 0.402481 0.917404

C 3.279366 -0.086358 1.477285

C 2.204102 2.733489 -1.318398

C -0.345377 4.216314 -1.185674

C -2.337520 -0.018608 0.905264

C -2.627547 -1.357940 0.296010

C -1.675520 -1.252860 1.462695

C -0.175701 -1.117201 1.183750

C -4.033803 -1.890523 0.423716

C -4.915297 -1.419257 -0.777358

C -4.513672 -0.003135 -1.145627

C -4.788173 -2.354451 -1.976988

C 4.148038 -0.342877 0.274554

C 3.887794 -1.415025 -0.578896

C 4.645564 -1.615489 -1.728103

C 5.680439 -0.740597 -2.041299

C 5.955465 0.329175 -1.193707

C 5.195474 0.524019 -0.045962

C 0.319955 -3.166009 0.065638

C 0.655691 -3.769518 -1.268708

H -4.430668 1.731724 -0.401538

N 1.508192 1.388140 2.420466

O -0.662699 1.254321 3.144605

H 1.612598 0.141084 -0.561501

H -1.165651 0.409016 -0.818575

O -2.606559 2.584261 -0.712338

O -3.811838 0.291198 -2.092574

O -4.961661 0.915920 -0.273658

H -3.109260 0.485225 1.466324

H -1.966473 -1.663449 2.418899

O 0.251864 -1.821602 -0.010330

O 0.115746 -3.786268 1.082731

H 6.763790 1.011243 -1.426393

H 6.271462 -0.892702 -2.935555

H 4.427251 -2.454360 -2.377423

H 3.082183 -2.096702 -0.339644

H 5.418926 1.359104 0.608269

H 2.689865 -0.973432 1.712551

H 3.898529 0.127754 2.352410

H 2.943867 1.986691 1.031046

H -1.622374 2.747129 1.036540

H 0.410291 -1.489838 2.020648

H 1.867094 1.794964 3.271073

H 1.932095 3.113010 -2.303405

H 2.689276 3.552455 -0.778929

H 2.952158 1.952177 -1.457127

H -1.007148 4.829155 -0.570214

H 0.601324 4.741418 -1.295793

H -0.813508 4.151529 -2.171824

H -2.585789 2.045070 -1.518996

H -2.165350 -1.536373 -0.668282

H -4.470058 -1.535786 1.358884

H -4.054137 -2.982514 0.449118

H -5.954090 -1.390697 -0.442193

H -5.379416 -1.999397 -2.821792

H -5.139642 -3.350773 -1.705898

H -3.751933 -2.432673 -2.307532

H 1.077289 -4.761452 -1.127348

H -0.265840 -3.859108 -1.848733

H 1.341111 -3.135944 -1.829018

YYY-141h-c7 , delta G = 0.9275 kcal/mol, population = 7.81 %

C 0.763606 0.876728 1.368689

C 2.364696 0.839022 -0.386687

C 0.948474 0.748365 -1.019360

C 0.517033 2.116251 -1.535846

C -0.529512 2.815012 -1.065615

C -1.490398 2.245672 -0.040112

C -1.386801 0.741915 -0.043436

C 0.057255 0.278533 0.131753

C 3.200462 -0.438745 -0.575394

C 1.414887 2.670789 -2.620241

C -0.840628 4.219031 -1.510804

C -2.178565 -0.067206 0.962415

C -2.710561 -1.369819 0.443557

C -1.411865 -1.343250 1.210188

C -0.101467 -1.240494 0.427316

C -3.997248 -1.895379 1.030582

C -5.237277 -1.312458 0.285755

C -5.009475 0.179265 0.107654

C -5.519208 -2.051671 -1.022404

C 4.500744 -0.380468 0.180273

C 4.615455 -0.953839 1.447657

C 5.798487 -0.848253 2.172946

C 6.886327 -0.165540 1.638364

C 6.783797 0.409703 0.374568

C 5.599524 0.303061 -0.346094

C -0.057640 -3.277138 -0.811090

C -0.196487 -3.863080 -2.187006

H -4.071447 1.395418 -1.015314

N 2.043775 1.114490 1.007377

O 0.282454 1.066735 2.475525

H 0.954111 0.034668 -1.843853

H -1.689228 0.435218 -1.049468

O -2.846043 2.651688 -0.383293

O -5.204979 1.000713 0.980774

O -4.495539 0.507734 -1.088109

H -2.688810 0.426998 1.775893

H -1.369607 -1.792219 2.192221

O -0.134876 -1.931037 -0.847088

O 0.092383 -3.907029 0.209579

H 7.627815 0.938527 -0.050757

H 7.808460 -0.084893 2.199953

H 5.870560 -1.301601 3.153753

H 3.771557 -1.488241 1.867777

H 5.526694 0.751408 -1.330645

H 3.388399 -0.553261 -1.645658

H 2.620836 -1.305306 -0.255357

H 2.929235 1.677480 -0.792953

H -1.266269 2.653760 0.950184

H 0.724182 -1.648085 1.005588

H 2.727017 1.431832 1.678133

H 0.841082 3.157653 -3.408329

H 2.124682 3.407541 -2.232522

H 1.998746 1.873447 -3.082970

H -1.652983 4.235138 -2.241762

H -1.179466 4.814605 -0.660377

H 0.023785 4.714150 -1.947802

H -3.335530 2.786522 0.437918

H -2.615981 -1.508721 -0.626894

H -4.051719 -1.622616 2.086284

H -4.048742 -2.985572 0.973601

H -6.098656 -1.410009 0.947998

H -6.370435 -1.617837 -1.548853

H -5.749307 -3.096522 -0.807701

H -4.661536 -2.019974 -1.693701

H 0.416717 -3.315815 -2.902270

H 0.084857 -4.912444 -2.169680

H -1.237015 -3.774524 -2.506786

YYY-141h-c3 , delta G = 0.9645 kcal/mol, population = 7.34 %

C 0.791392 0.981484 1.297095

C 2.373577 0.804041 -0.466856

C 0.949909 0.680371 -1.076902

C 0.522545 2.014059 -1.680218

C -0.515864 2.750000 -1.249477

C -1.491574 2.253657 -0.194427

C -1.374296 0.755960 -0.077790

C 0.068885 0.299487 0.114209

C 3.197456 -0.491108 -0.571452

C 1.417284 2.491273 -2.803609

C -0.815318 4.126938 -1.781621

C -2.153096 0.036005 0.999422

C -2.714359 -1.295011 0.598514

C -1.396824 -1.224849 1.331844

C -0.099762 -1.194153 0.519193

C -4.001716 -1.733529 1.252992

C -5.235763 -1.230512 0.437067

C -4.923327 0.148864 -0.113762

C -5.605137 -2.197248 -0.685023

C 4.504409 -0.390511 0.168015

C 5.604984 0.242352 -0.414879

C 6.795895 0.390116 0.287392

C 6.903443 -0.092413 1.588984

C 5.813901 -0.724082 2.179908

C 4.624233 -0.871092 1.472972

C -0.101703 -3.311735 -0.576125

C -0.277655 -3.989368 -1.904838

H -4.480204 1.889492 0.473410

N 2.069626 1.181896 0.906778

O 0.323156 1.255860 2.391445

H 0.941310 -0.088839 -1.849976

H -1.686089 0.361785 -1.052277

O -2.853463 2.628706 -0.508978

O -4.604449 0.377704 -1.263368

O -4.970328 1.113214 0.821157

H -2.638284 0.600390 1.780523

H -1.337763 -1.593283 2.345972

O -0.165668 -1.970349 -0.703340

O 0.064144 -3.871414 0.482305

H 5.889787 -1.105328 3.190652

H 7.830702 0.020297 2.136443

H 7.641127 0.878588 -0.181550

H 5.528221 0.618339 -1.428981

H 3.778956 -1.365241 1.937245

H 3.375391 -0.682453 -1.632426

H 2.613778 -1.327961 -0.186328

H 2.940521 1.606004 -0.938046

H -1.296127 2.741702 0.763133

H 0.731922 -1.570546 1.109745

H 2.761229 1.545138 1.544813

H 1.989656 1.661132 -3.220930

H 0.841961 2.935162 -3.615649

H 2.137298 3.243251 -2.467222

H -1.168382 4.770531 -0.973322

H 0.058295 4.594274 -2.231247

H -1.612800 4.105866 -2.529385

H -3.154749 2.082700 -1.252324

H -2.647484 -1.526999 -0.458528

H -4.045662 -1.334363 2.267597

H -4.068193 -2.821344 1.328959

H -6.076640 -1.127743 1.125907

H -6.443928 -1.822982 -1.272970

H -5.885372 -3.162475 -0.261404

H -4.765957 -2.350061 -1.364430

H -1.325053 -3.913832 -2.205002

H 0.321718 -3.497879 -2.670503

H -0.003602 -5.037549 -1.821951

YYY-141h-c6-gfn0 , delta G = 0.9883 kcal/mol, population = 7.05 %

C 0.752911 1.156118 1.423908

C 2.352695 1.059857 -0.330459

C 0.963992 1.375228 -0.955056

C 0.849275 2.868825 -1.221626

C -0.025812 3.679481 -0.605358

C -1.137677 3.164961 0.298925

C -1.355774 1.691674 0.040804

C -0.033799 0.924210 0.112050

C 2.912182 -0.315189 -0.735080

C 1.865301 3.400032 -2.208577

C 0.004888 5.177252 -0.771816

C -2.316250 0.902916 0.922312

C -3.103856 -0.159152 0.218832

C -1.807351 -0.521119 0.914262

C -0.484581 -0.558061 0.166104

C -4.387996 -0.677851 0.830734

C -4.976899 -1.914729 0.114249

C -3.864917 -2.838819 -0.325244

C -6.016449 -2.624390 0.985955

C 4.181342 -0.647561 0.002682

C 5.406824 -0.126479 -0.419500

C 6.572895 -0.381855 0.293346

C 6.530008 -1.163119 1.445033

C 5.315419 -1.687893 1.874687

C 4.150807 -1.430900 1.157485

C -0.591131 -2.388186 -1.362455

C -0.640140 -2.777613 -2.807850

H -2.364553 -3.767978 0.348867

N 2.062881 1.165276 1.092683

O 0.296937 1.262540 2.551895

H 0.840999 0.821729 -1.886361

H -1.695216 1.617051 -0.999902

O -2.353008 3.907951 0.110219

O -3.558446 -3.026587 -1.483600

O -3.202091 -3.405185 0.697657

H -2.697335 1.351994 1.829008

H -1.887297 -1.148329 1.789473

O -0.577924 -1.055528 -1.197328

O -0.574527 -3.171204 -0.433080

H 5.273637 -2.299654 2.767285

H 7.437293 -1.364319 2.000544

H 7.515453 0.025411 -0.051058

H 5.447339 0.480573 -1.317022

H 3.207596 -1.843539 1.495343

H 3.094090 -0.291588 -1.812253

H 2.162112 -1.084658 -0.550784

H 3.088275 1.816949 -0.599477

H -0.882773 3.338252 1.347124

H 0.225716 -1.192772 0.690110

H 2.780942 1.220185 1.798858

H 2.261079 2.592599 -2.827323

H 1.427436 4.142312 -2.875612

H 2.717223 3.873739 -1.711693

H -0.260938 5.663199 0.168683

H 0.987212 5.535019 -1.074519

H -0.721729 5.521387 -1.513302

H -2.650843 3.764909 -0.797239

H -3.090329 -0.121142 -0.865943

H -5.151851 0.104336 0.814671

H -4.210556 -0.923050 1.880793

H -5.446962 -1.586040 -0.813587

H -5.559327 -3.005153 1.899787

H -6.477392 -3.459578 0.456216

H -6.804726 -1.922589 1.263966

H -0.066943 -3.691012 -2.954581

H -1.683230 -2.983142 -3.053176

H -0.269332 -1.982744 -3.450754

YYY-141h-c2-gfn0 , delta G = 1.2205 kcal/mol, population = 4.76 %

C 0.116724 1.288976 2.231964

C 2.301711 0.997376 1.338507

C 1.314298 1.200984 0.152948

C 1.399482 2.637896 -0.337518

C 0.394924 3.524268 -0.252604

C -1.015548 3.138350 0.172955

C -1.191248 1.642787 0.042631

C -0.059373 0.890708 0.747547

C 2.959492 -0.393479 1.384761

C 2.756921 3.018627 -0.887494

C 0.570275 4.980603 -0.598828

C -2.468981 0.985550 0.550761

C -2.978414 -0.157466 -0.271224

C -2.082934 -0.428969 0.920831

C -0.575684 -0.570444 0.770042

C -4.429979 -0.575681 -0.159171

C -4.776573 -1.876505 -0.916815

C -3.651906 -2.876157 -0.781622

C -6.121395 -2.448694 -0.459173

C 3.932451 -0.576981 0.249719

C 5.158409 0.093380 0.254682

C 6.033596 -0.013242 -0.819831

C 5.696735 -0.796516 -1.920697

C 4.483067 -1.475414 -1.933639

C 3.610347 -1.363914 -0.855671

C -0.214615 -2.554446 -0.509445

C 0.251822 -3.102372 -1.824096

H -2.583052 -3.724710 0.523750

N 1.443989 1.254548 2.486204

O -0.761126 1.544366 3.041435

H 1.565266 0.523952 -0.662558

H -1.083414 1.420500 -1.026112

O -2.005403 3.841187 -0.596351

O -2.949679 -3.234744 -1.703279

O -3.463720 -3.304200 0.478181

H -3.148989 1.554495 1.169251

H -2.535701 -0.929385 1.763856

O -0.161818 -1.213604 -0.467975

O -0.602050 -3.232695 0.421468

H 4.214203 -2.092584 -2.781971

H 6.377520 -0.880201 -2.758350

H 6.979752 0.513143 -0.798050

H 5.430167 0.705027 1.107580

H 2.667300 -1.893398 -0.872722

H 2.190122 -1.165901 1.355379

H 3.476361 -0.492532 2.342708

H 3.094778 1.743560 1.311410

H -1.204280 3.460167 1.199898

H -0.163028 -1.153904 1.589480

H 1.800607 1.440518 3.411492

H 3.349518 3.592242 -0.168490

H 3.333827 2.128226 -1.140402

H 2.667724 3.625746 -1.788412

H -0.016102 5.600186 0.082413

H 1.610781 5.293311 -0.535590

H 0.211826 5.206996 -1.607041

H -1.903596 3.579938 -1.520519

H -2.538397 -0.269195 -1.257162

H -5.076559 0.213633 -0.552243

H -4.686150 -0.696543 0.896182

H -4.823224 -1.655058 -1.983893

H -6.087042 -2.720720 0.596429

H -6.394669 -3.333901 -1.035676

H -6.904738 -1.700891 -0.594535

H -0.625962 -3.487473 -2.343861

H 0.731851 -2.341068 -2.433816

H 0.932977 -3.933434 -1.644194

YYY-141h-c5 , delta G = 1.3046 kcal/mol, population = 4.13 %

C 0.188512 1.067591 2.286641

C 2.328239 1.108278 1.248949

C 1.259738 0.872906 0.144127

C 0.994862 2.174123 -0.603206

C -0.174253 2.835544 -0.596276

C -1.418078 2.268614 0.059852

C -1.268597 0.773764 0.189328

C 0.024329 0.393144 0.907420

C 3.273456 -0.084827 1.481134

C 2.202951 2.704102 -1.345053

C -0.352982 4.184960 -1.238579

C -2.345764 -0.033345 0.883344

C -2.618062 -1.376123 0.273330

C -1.680381 -1.263625 1.449588

C -0.179562 -1.124445 1.182187

C -4.014182 -1.936481 0.387330

C -4.917131 -1.439347 -0.783562

C -4.667161 0.048977 -0.958723

C -4.698315 -2.252619 -2.059517

C 4.149473 -0.344113 0.284355

C 3.893430 -1.417888 -0.568258

C 4.658058 -1.621648 -1.712303

C 5.695647 -0.748404 -2.021020

C 5.966422 0.323084 -1.174206

C 5.199550 0.521252 -0.031593

C 0.326517 -3.180515 0.082715

C 0.672537 -3.793771 -1.244637

H -3.420334 1.244418 -1.753106

N 1.495424 1.390316 2.409690

O -0.676906 1.253437 3.128828

H 1.612375 0.123534 -0.562796

H -1.168457 0.405499 -0.836249

O -2.574394 2.584079 -0.765518

O -5.170872 0.905418 -0.259354

O -3.771118 0.336868 -1.915323

H -3.126280 0.465727 1.438527

H -1.978557 -1.675773 2.402890

O 0.259545 -1.836756 -0.003195

O 0.113802 -3.793708 1.102342

H 6.776825 1.003873 -1.403367

H 6.292143 -0.903015 -2.911195

H 4.443245 -2.461786 -2.361118

H 3.085579 -2.098028 -0.332381

H 5.419453 1.357526 0.622300

H 2.685233 -0.972663 1.717030

H 3.887183 0.135077 2.358620

H 2.933791 1.984701 1.021146

H -1.575112 2.736815 1.036708

H 0.401335 -1.489283 2.026089

H 1.851608 1.799528 3.260355

H 1.934589 3.067129 -2.337171

H 2.684769 3.532034 -0.816455

H 2.952599 1.921828 -1.468258

H 0.595349 4.695457 -1.392058

H -0.859796 4.104253 -2.203489

H -0.983979 4.818404 -0.611212

H -3.344251 2.658836 -0.186147

H -2.143351 -1.546153 -0.685730

H -4.453072 -1.624858 1.337064

H -4.011763 -3.029211 0.373205

H -5.956494 -1.536608 -0.466814

H -5.307331 -1.874530 -2.881741

H -4.976858 -3.292425 -1.880898

H -3.656464 -2.226160 -2.378009

H 1.354670 -3.160131 -1.808761

H 1.101537 -4.780935 -1.092388

H -0.246309 -3.896832 -1.826715

YYY-141h-c10-gfn0 , delta G = 1.4019 kcal/mol, population = 3.50 %

C 0.755106 1.131711 1.434919

C 2.357258 1.061036 -0.318127

C 0.968049 1.370573 -0.943808

C 0.846163 2.862586 -1.216869

C -0.036929 3.672122 -0.612302

C -1.123908 3.155049 0.312016

C -1.349467 1.682984 0.051980

C -0.029689 0.912661 0.120116

C 2.925424 -0.308004 -0.730655

C 1.861456 3.388489 -2.208196

C -0.031068 5.166184 -0.796769

C -2.308346 0.891003 0.936374

C -3.104203 -0.162411 0.228008

C -1.805059 -0.534817 0.912057

C -0.483747 -0.569013 0.160787

C -4.385883 -0.686545 0.840231

C -4.976089 -1.917093 0.114076

C -3.864887 -2.837948 -0.334774

C -6.014728 -2.633833 0.980972

C 4.194164 -0.639809 0.007917

C 5.418140 -0.109088 -0.406558

C 6.583504 -0.364423 0.307435

C 6.541435 -1.155241 1.452627

C 5.328338 -1.689587 1.874644

C 4.164434 -1.432636 1.156264

C -0.598044 -2.385617 -1.382584

C -0.651320 -2.754508 -2.833117

H -2.365699 -3.775851 0.328707

N 2.064845 1.154803 1.105450

O 0.297787 1.219103 2.564650

H 0.849077 0.816083 -1.875055

H -1.698293 1.620967 -0.983534

O -2.318683 3.913523 0.046294

O -3.559433 -3.015766 -1.494654

O -3.201446 -3.413952 0.682721

H -2.675653 1.322197 1.858965

H -1.883689 -1.166997 1.783796

O -0.582220 -1.054266 -1.205510

O -0.579673 -3.176756 -0.460252

H 5.287243 -2.308865 2.762083

H 7.448235 -1.356471 2.008915

H 7.524940 0.050289 -0.031098

H 5.458017 0.505362 -1.299034

H 3.222285 -1.852763 1.487915

H 3.109781 -0.275439 -1.807171

H 2.178359 -1.082417 -0.554027

H 3.088184 1.824826 -0.580920

H -0.829475 3.319989 1.355905

H 0.225624 -1.210202 0.678306

H 2.781844 1.202072 1.813327

H 1.417026 4.106600 -2.896856

H 2.702488 3.886702 -1.716937

H 2.273995 2.572948 -2.804851

H 0.913194 5.528464 -1.197115

H -0.835789 5.488238 -1.461873

H -0.206798 5.660835 0.161227

H -2.965948 3.683107 0.722184

H -3.096718 -0.109798 -0.855959

H -5.150531 0.095146 0.832606

H -4.205148 -0.941158 1.887553

H -5.446957 -1.580022 -0.810278

H -6.476009 -3.464671 0.444801

H -6.803007 -1.934550 1.265314

H -5.556735 -3.022179 1.891184

H -1.700482 -2.766221 -3.132606

H -0.117915 -2.029762 -3.445116

H -0.240764 -3.752153 -2.969337

YYY-141h-c7-gfn0 , delta G = 1.7865 kcal/mol, population = 1.83 %

C 0.119167 1.270923 2.243836

C 2.304799 1.000196 1.345860

C 1.315432 1.196535 0.161064

C 1.396382 2.629520 -0.342680

C 0.390158 3.514210 -0.271622

C -1.004401 3.131491 0.189157

C -1.187475 1.636891 0.055858

C -0.056453 0.881644 0.757273

C 2.967934 -0.388134 1.394208

C 2.752489 3.002107 -0.902513

C 0.551325 4.962302 -0.650630

C -2.465438 0.978313 0.567994

C -2.980225 -0.157637 -0.261677

C -2.079156 -0.439809 0.923160

C -0.571883 -0.579963 0.769773

C -4.430353 -0.580058 -0.147922

C -4.775098 -1.875909 -0.914520

C -3.650088 -2.876344 -0.785925

C -6.119542 -2.452796 -0.461561

C 3.940701 -0.571315 0.259043

C 5.166467 0.099305 0.264127

C 6.041468 -0.006577 -0.810589

C 5.704470 -0.789331 -1.911764

C 4.490920 -1.468423 -1.924862

C 3.618461 -1.357820 -0.846629

C -0.211836 -2.554945 -0.522605

C 0.252492 -3.093179 -1.841777

H -2.577431 -3.729158 0.513514

N 1.446902 1.253460 2.494532

O -0.760151 1.508235 3.058171

H 1.567466 0.516216 -0.651315

H -1.089916 1.426616 -1.013580

O -1.947159 3.838360 -0.638989

O -2.951885 -3.233376 -1.710940

O -3.457885 -3.307626 0.472333

H -3.139467 1.532726 1.208064

H -2.530302 -0.944376 1.764504

O -0.160240 -1.214419 -0.472452

O -0.597585 -3.239522 0.404567

H 4.221720 -2.084657 -2.773739

H 6.384868 -0.872109 -2.749812

H 6.987374 0.520258 -0.788911

H 5.437828 0.710983 1.117122

H 2.675296 -1.887216 -0.864063

H 2.200650 -1.162794 1.366654

H 3.485684 -0.484081 2.352018

H 3.094175 1.750270 1.317198

H -1.150609 3.446270 1.229853

H -0.158977 -1.169113 1.585105

H 1.803166 1.432829 3.421291

H 3.345134 3.593311 -0.197996

H 3.330947 2.107441 -1.135592

H 2.661170 3.586334 -1.818109

H 0.029337 5.597742 0.068462

H 1.595498 5.266158 -0.678031

H 0.105187 5.170151 -1.626145

H -2.824080 3.701026 -0.263335

H -2.544685 -0.256010 -1.250753

H -5.080250 0.209948 -0.534396

H -4.683508 -0.709496 0.907194

H -4.821457 -1.647064 -1.979984

H -6.903944 -1.705347 -0.592816

H -6.085754 -2.731937 0.592222

H -6.391018 -3.334491 -1.044189

H -0.627837 -3.464553 -2.367381

H 0.739970 -2.329517 -2.442548

H 0.925531 -3.932322 -1.669469

YYY-141h-c17-gfn0 , delta G = 1.8286 kcal/mol, population = 1.70 %

C 0.740960 1.048132 1.513784

C 2.350801 1.094257 -0.233425

C 0.964052 1.449377 -0.839927

C 0.842967 2.957743 -0.996401

C -0.045537 3.711596 -0.331516

C -1.154176 3.121983 0.532425

C -1.364800 1.676488 0.161722

C -0.037917 0.914770 0.183566

C 2.920750 -0.242676 -0.738525

C 1.861486 3.561656 -1.939247

C -0.044363 5.215914 -0.397525

C -2.323015 0.816925 0.977661

C -3.100098 -0.191295 0.189766

C -1.805222 -0.599235 0.863130

C -0.478784 -0.570424 0.121964

C -4.384420 -0.763646 0.750814

C -4.959005 -1.945929 -0.062699

C -3.837489 -2.828187 -0.560076

C -6.002223 -2.725345 0.742529

C 4.187999 -0.623436 -0.021174

C 4.156342 -1.494022 1.069351

C 5.318701 -1.798834 1.771341

C 6.532213 -1.235144 1.390739

C 6.576259 -0.366804 0.303231

C 5.412420 -0.063935 -0.394509

C -0.564604 -2.280592 -1.540962

C -0.603257 -2.552032 -3.013472

H -2.337976 -3.797844 0.054866

N 2.052732 1.089164 1.191952

O 0.280011 1.064679 2.645033

H 0.848050 0.967267 -1.810945

H -1.704123 1.687750 -0.879259

O -2.406117 3.796333 0.306302

O -3.519327 -2.927494 -1.726023

O -3.180004 -3.466761 0.423618

H -2.711959 1.192487 1.913981

H -1.886323 -1.293381 1.686197

O -0.561115 -0.964368 -1.275328

O -0.546595 -3.131903 -0.673712

H 7.518065 0.071357 -0.003234

H 7.437803 -1.473491 1.934194

H 5.276050 -2.478281 2.613526

H 3.214016 -1.937388 1.368532

H 5.453896 0.610842 -1.242234

H 3.108049 -0.135697 -1.809706

H 2.174355 -1.028122 -0.617851

H 3.083402 1.873768 -0.439605

H -0.892359 3.210012 1.593049

H 0.233622 -1.237893 0.600502

H 2.766676 1.089269 1.904400

H 2.699685 4.020656 -1.406785

H 2.278051 2.796356 -2.596502

H 1.418628 4.330606 -2.571851

H 0.891962 5.615460 -0.779497

H -0.855818 5.589997 -1.027484

H -0.194766 5.638206 0.601297

H -2.388160 4.643703 0.763724

H -3.081011 -0.067601 -0.888354

H -5.153523 0.012642 0.787852

H -4.212966 -1.087393 1.780501

H -5.422376 -1.550283 -0.967447

H -5.551523 -3.171337 1.629700

H -6.452719 -3.520887 0.146843

H -6.797692 -2.050998 1.064581

H -1.649573 -2.547927 -3.322866

H -0.067792 -1.785701 -3.570618

H -0.186651 -3.536564 -3.212287

YYY-141h-c13-gfn0 , delta G = 2.0237 kcal/mol, population = 1.23 %

C 0.111917 1.300997 2.230329

C 2.298734 1.021020 1.337621

C 1.310019 1.212627 0.151544

C 1.386594 2.645782 -0.350746

C 0.373144 3.521599 -0.277981

C -1.032305 3.128686 0.162905

C -1.199220 1.636220 0.038210

C -0.061920 0.897025 0.747167

C 2.964234 -0.365955 1.391729

C 2.742248 3.024623 -0.908593

C 0.524587 4.971186 -0.655630

C -2.471279 0.972362 0.552310

C -2.972428 -0.175553 -0.266853

C -2.074958 -0.438150 0.926181

C -0.566927 -0.568198 0.775544

C -4.420816 -0.604041 -0.154692

C -4.757605 -1.907925 -0.910928

C -3.630141 -2.903382 -0.768492

C -6.102009 -2.485693 -0.458708

C 3.941042 -0.549450 0.260064

C 3.623196 -1.337190 -0.845985

C 4.499552 -1.448298 -1.920998

C 5.712698 -0.768552 -1.904239

C 6.045343 0.015466 -0.802631

C 5.166402 0.121917 0.268832

C -0.194288 -2.553861 -0.497738

C 0.271454 -3.102290 -1.812435

H -2.556939 -3.736781 0.542866

N 1.439509 1.278970 2.484116

O -0.766811 1.551878 3.040839

H 1.564094 0.532514 -0.660381

H -1.096039 1.418989 -1.029826

O -2.033124 3.727420 -0.682309

O -2.928918 -3.269780 -1.687701

O -3.439197 -3.319977 0.495076

H -3.155564 1.537926 1.169204

H -2.523944 -0.940814 1.769915

O -0.148093 -1.213105 -0.459788

O -0.576715 -3.232051 0.435573

H 6.991010 0.542625 -0.777979

H 6.396188 -0.851917 -2.739720

H 4.233748 -2.065549 -2.770214

H 2.680378 -1.866984 -0.866164

H 5.434521 0.734284 1.122383

H 2.199017 -1.142528 1.363826

H 3.479372 -0.458070 2.351320

H 3.087611 1.771653 1.306633

H -1.208179 3.449016 1.196195

H -0.149190 -1.144970 1.597184

H 1.794755 1.466552 3.409600

H 3.331383 3.617867 -0.202967

H 3.324737 2.132302 -1.140757

H 2.650413 3.607753 -1.824887

H 0.038723 5.609853 0.089217

H 1.565890 5.278387 -0.715815

H 0.051053 5.185845 -1.617318

H -2.130197 4.654036 -0.438555

H -2.532043 -0.284122 -1.252811

H -5.072125 0.180167 -0.550170

H -4.677262 -0.725242 0.900654

H -4.800251 -1.688955 -1.978650

H -6.071721 -2.755340 0.597675

H -6.368251 -3.373397 -1.034699

H -6.888191 -1.741896 -0.599676

H 0.751950 -2.341267 -2.422136

H 0.951138 -3.934622 -1.633090

H -0.607277 -3.485400 -2.332194

YYY-141h-c8 , delta G = 2.6362 kcal/mol, population = 0.44 %

C 0.734819 1.042074 1.925094

C 2.448398 2.015443 0.578945

C 1.300623 1.546077 -0.373776

C 0.590334 2.794907 -0.885191

C -0.637355 3.184310 -0.507283

C -1.522269 2.295122 0.342754

C -1.092201 0.863790 0.138444

C 0.387933 0.646947 0.474271

C 3.833778 1.381389 0.355403

C 1.446054 3.644279 -1.801941

C -1.234688 4.508223 -0.898784

C -1.814047 -0.256658 0.855671

C -2.030537 -1.493720 0.033161

C -0.839432 -1.401791 0.951106

C 0.495055 -0.904088 0.384414

C -3.237892 -2.345671 0.336512

C -4.486527 -1.853950 -0.459761

C -4.521094 -0.338588 -0.365984

C -4.491285 -2.381330 -1.894624

C 3.904285 -0.117869 0.448176

C 3.921631 -0.759056 1.689569

C 3.980291 -2.145614 1.775368

C 4.034066 -2.915381 0.617796

C 4.031873 -2.288823 -0.623234

C 3.963255 -0.902537 -0.704838

C 0.831704 -2.521476 -1.339841

C 1.001457 -2.685367 -2.824084

H -3.737371 1.200721 -1.155283

N 1.885140 1.747968 1.889798

O 0.109024 0.763624 2.937435

H 1.716395 0.996743 -1.217483

H -1.184676 0.692638 -0.937785

O -2.902994 2.472723 -0.071299

O -4.935731 0.269171 0.601804

O -3.975387 0.281937 -1.423227

H -2.482261 -0.038755 1.675575

H -0.799650 -2.033458 1.826420

O 0.677616 -1.223521 -1.017834

O 0.823505 -3.419603 -0.532256

H 4.077058 -2.880937 -1.528696

H 4.072718 -3.994929 0.682235

H 3.985543 -2.624774 2.746332

H 3.891155 -0.168922 2.596570

H 3.960846 -0.421331 -1.675739

H 4.516802 1.825691 1.085157

H 4.177527 1.706749 -0.629069

H 2.587503 3.092194 0.474093

H -1.456179 2.585866 1.396997

H 1.340359 -1.310861 0.931772

H 2.296196 2.096305 2.743076

H 0.889389 3.965264 -2.682953

H 1.818521 4.546532 -1.308846

H 2.314544 3.083128 -2.148474

H -1.742490 4.958759 -0.042890

H -0.482733 5.208212 -1.256042

H -1.991929 4.389072 -1.677527

H -3.473405 2.195127 0.659149

H -1.816439 -1.384199 -1.023387

H -3.452966 -2.302479 1.405854

H -3.063584 -3.393882 0.081279

H -5.376714 -2.210850 0.060111

H -5.344553 -1.999710 -2.456946

H -4.552480 -3.470646 -1.881058

H -3.585295 -2.096082 -2.429087

H 0.078843 -2.396823 -3.331176

H 1.794245 -2.030513 -3.185964

H 1.235229 -3.721020 -3.054788

### Table S15. Geometry data of conformers of structure 2a.

YYY-148a-c4 , delta G = 0.0000 kcal/mol, population = 53.69 %

C -5.391522 -0.899193 -2.889460

C -6.506506 -0.094255 -2.678777

C -6.618898 0.628523 -1.494024

C -5.620135 0.546700 -0.530307

C -4.495814 -0.257606 -0.731011

C -4.394718 -0.979312 -1.921551

C -3.389610 -0.296884 0.289057

C -2.418314 0.884993 0.133108

C -1.219453 0.854894 1.114570

C -0.114299 0.112152 0.345263

C 1.241556 0.465235 0.976228

C 1.588185 1.906570 0.670933

C 0.443952 2.776416 1.167606

C -0.776342 2.281731 1.435647

C -0.117841 -1.446300 0.344572

C 1.339677 -1.864305 0.303589

C 2.189611 -0.668170 0.676269

N -1.726995 0.895901 -1.146779

C -0.412340 0.589898 -1.105882

O 0.342787 0.651673 -2.066607

C -1.860026 3.133579 2.059316

C 0.796596 4.226388 1.362777

H -1.494303 0.334577 2.031845

H 1.077311 0.427735 2.059277

O 2.796085 2.305763 1.333070

C 2.261296 -1.236300 -0.710305

C 3.518743 -1.967612 -1.112307

C 4.626628 -1.009199 -1.621594

C 4.978932 -0.022020 -0.528532

C 4.233876 -0.290481 -2.909813

O 4.678232 1.155137 -0.517236

O 5.653900 -0.607187 0.471823

C 5.982792 0.224117 1.605135

H 3.035530 -0.804087 1.336124

H 1.567717 -2.859497 0.661436

O -0.683859 -1.883249 1.611180

O -1.327171 -3.844875 0.702951

C -1.263948 -3.099318 1.653315

C -1.830395 -3.374936 3.016794

H -5.298309 -1.468388 -3.806048

H -7.284380 -0.033673 -3.429459

H -7.486679 1.252587 -1.319559

H -5.715409 1.108835 0.391960

H -3.530396 -1.611206 -2.088069

H -3.804445 -0.251835 1.298895

H -2.842756 -1.234601 0.202378

H -2.991390 1.805564 0.235446

H 1.719764 2.046669 -0.407300

H -0.717439 -1.874306 -0.455766

H -2.160678 1.187552 -2.009301

H -2.373318 3.761931 1.324942

H -2.618236 2.504173 2.529396

H -1.460284 3.794077 2.827891

H 1.444868 4.561854 0.550626

H -0.082380 4.867443 1.384087

H 1.359127 4.378328 2.287343

H 3.531549 2.028947 0.763721

H 1.802630 -0.650796 -1.492051

H 3.302511 -2.678363 -1.914186

H 3.905150 -2.544020 -0.268688

H 5.519164 -1.614965 -1.796582

H 5.050970 0.331275 -3.277397

H 3.988750 -1.024230 -3.679205

H 3.366983 0.351075 -2.758247

H 6.588571 1.071960 1.289797

H 5.074105 0.584268 2.086141

H 6.541741 -0.415821 2.280614

H -1.074787 -3.200511 3.782850

H -2.658286 -2.689227 3.207516

H -2.188025 -4.399566 3.066407

YYY-148a-c3 , delta G = 0.4116 kcal/mol, population = 26.78 %

C -5.607333 -0.582288 1.312655

C -6.567273 0.355407 0.947454

C -6.443134 1.027744 -0.265526

C -5.364027 0.765355 -1.101575

C -4.390432 -0.170567 -0.743359

C -4.529011 -0.839774 0.471982

C -3.186332 -0.390342 -1.621750

C -2.171488 0.754953 -1.464914

C -1.393655 0.757736 -0.122218

C -0.077546 0.017990 -0.428100

C 0.974730 0.445623 0.607518

C 1.369758 1.889184 0.379783

C 0.105517 2.730740 0.441404

C -1.120163 2.199406 0.300656

C -0.045643 -1.537879 -0.345780

C 1.353579 -1.900632 0.118635

C 1.997172 -0.659440 0.699075

N -1.082128 0.687328 -2.424143

C 0.139698 0.421923 -1.914923

O 1.178617 0.467479 -2.559358

C -2.374204 3.019635 0.515298

C 0.331913 4.193539 0.712666

H -1.969736 0.252301 0.650884

H 0.452131 0.436195 1.570701

O 2.279769 2.344509 1.390396

C 2.550711 -1.277235 -0.551045

C 3.884253 -1.978552 -0.465362

C 5.081304 -1.003716 -0.610136

C 5.020979 0.033138 0.492108

C 5.141768 -0.345110 -1.985930

O 4.711101 1.199139 0.348134

O 5.326016 -0.493683 1.687208

C 5.233625 0.387051 2.827103

H 2.570342 -0.736586 1.612970

H 1.472227 -2.871825 0.579804

O -0.978982 -1.942297 0.698638

O -1.069744 -4.029769 -0.149371

C -1.378798 -3.229481 0.703248

C -2.237510 -3.544162 1.896842

H -5.696673 -1.114324 2.251744

H -7.407441 0.558752 1.599331

H -7.188779 1.755451 -0.560959

H -5.276345 1.292188 -2.045084

H -3.783874 -1.566449 0.762997

H -2.709145 -1.342842 -1.389710

H -3.491906 -0.430963 -2.670223

H -2.706710 1.693365 -1.603511

H 1.851094 2.004283 -0.597361

H -0.345791 -2.025556 -1.270796

H -1.180242 0.960905 -3.390263

H -2.601056 3.668820 -0.335814

H -3.238566 2.370268 0.662438

H -2.286381 3.658693 1.393784

H -0.524901 4.802644 0.431183

H 0.552381 4.373858 1.767793

H 1.201424 4.544863 0.153200

H 3.166685 2.062603 1.115410

H 2.372590 -0.741524 -1.470001

H 3.969433 -2.725555 -1.258825

H 3.969997 -2.509509 0.485410

H 5.990469 -1.586457 -0.442649

H 4.267829 0.278730 -2.167656

H 6.027584 0.283964 -2.080698

H 5.183061 -1.113844 -2.759153

H 5.896646 1.241174 2.700235

H 4.209474 0.736369 2.953319

H 5.538306 -0.211229 3.680025

H -1.686570 -4.230052 2.542955

H -2.493514 -2.652075 2.462600

H -3.140599 -4.056242 1.565882

YYY-148a-c2 , delta G = 0.7122 kcal/mol, population = 16.12 %

C -4.878955 -0.713297 -3.260578

C -5.975672 0.140685 -3.202964

C -6.235256 0.848474 -2.032405

C -5.400045 0.703812 -0.930106

C -4.295655 -0.150448 -0.976529

C -4.046585 -0.856304 -2.154617

C -3.356117 -0.259455 0.194628

C -2.339174 0.893114 0.223873

C -1.260640 0.762526 1.328829

C -0.097107 0.024452 0.642734

C 1.187311 0.303098 1.439352

C 1.600570 1.743658 1.235065

C 0.440086 2.621565 1.675461

C -0.814715 2.152570 1.784746

C -0.135397 -1.530664 0.544997

C 1.307758 -1.989213 0.651786

C 2.134369 -0.843009 1.193320

N -1.506500 0.953775 -0.967196

C -0.217050 0.593259 -0.801472

O 0.641892 0.678831 -1.670282

C -1.935413 2.999829 2.346622

C 0.813183 4.042598 1.999294

H -1.652468 0.200745 2.176579

H 0.901490 0.224934 2.494259

O 2.767886 2.066823 2.000213

C 2.357908 -1.327168 -0.205132

C 3.659042 -2.031138 -0.515761

C 4.876340 -1.090379 -0.355605

C 4.552347 0.284413 -0.901651

C 6.139355 -1.678296 -0.991087

O 4.469146 1.295295 -0.230171

O 4.335204 0.270303 -2.219954

C 3.878753 1.503680 -2.818696

H 2.900453 -1.027529 1.934583

H 1.468476 -3.011193 0.967827

O -0.861981 -2.025480 1.703139

O -1.418139 -3.915676 0.605892

C -1.461115 -3.228881 1.600578

C -2.193960 -3.571516 2.866027

H -4.672082 -1.271068 -4.165495

H -6.626129 0.251108 -4.061445

H -7.090524 1.510613 -1.977203

H -5.610189 1.255652 -0.020681

H -3.195422 -1.524990 -2.203338

H -3.913855 -0.231901 1.133819

H -2.830161 -1.212103 0.155755

H -2.892319 1.826772 0.317828

H 1.823453 1.929400 0.180038

H -0.641747 -1.891990 -0.347500

H -1.835616 1.295871 -1.857004

H -2.349389 3.691118 1.606124

H -2.756773 2.368325 2.691118

H -1.602016 3.595699 3.195461

H -0.046771 4.709178 1.997717

H 1.304715 4.111540 2.972848

H 1.533775 4.411398 1.266214

H 3.523325 1.810116 1.448455

H 2.004360 -0.682241 -0.995774

H 3.632908 -2.417558 -1.537140

H 3.812353 -2.886295 0.147722

H 5.050474 -0.930389 0.708703

H 7.004681 -1.033124 -0.829739

H 6.355836 -2.651197 -0.546147

H 6.006066 -1.814959 -2.064614

H 2.889646 1.747726 -2.434789

H 4.577567 2.310339 -2.605701

H 3.834086 1.305309 -3.885166

H -3.022022 -2.873504 3.003680

H -2.579005 -4.585545 2.805142

H -1.531255 -3.469656 3.725531

YYY-148a-c1 , delta G = 1.6315 kcal/mol, population = 3.41 %

C -5.785318 -0.582834 0.973003

C -6.678760 0.397520 0.554792

C -6.400123 1.136149 -0.592217

C -5.234639 0.895651 -1.310153

C -4.327674 -0.084092 -0.898422

C -4.619508 -0.818507 0.250536

C -3.035466 -0.281324 -1.646970

C -2.027022 0.829085 -1.305221

C -1.389413 0.714906 0.104278

C -0.056869 -0.022733 -0.125823

C 0.887567 0.309219 1.040323

C 1.320219 1.757167 0.944281

C 0.062881 2.611829 0.973580

C -1.147727 2.113388 0.670566

C -0.044264 -1.580640 -0.165330

C 1.295759 -1.999271 0.414409

C 1.882053 -0.817613 1.156416

N -0.847439 0.812397 -2.154346

C 0.313919 0.492372 -1.546969

O 1.414251 0.574961 -2.078076

C -2.413965 2.927213 0.832712

C 0.277179 4.039936 1.394079

H -2.046157 0.160710 0.772533

H 0.270737 0.246959 1.943784

O 2.175978 2.120271 2.033787

C 2.560251 -1.337903 -0.072412

C 3.897418 -2.023809 0.091501

C 4.983044 -1.054999 0.616471

C 4.842685 0.298128 -0.047304

C 6.391207 -1.634335 0.454003

O 4.527583 1.322391 0.528289

O 5.076271 0.247996 -1.361867

C 4.828309 1.456222 -2.114617

H 2.363997 -0.962487 2.114075

H 1.361312 -3.007144 0.801005

O -1.088281 -2.052225 0.735538

O -1.074993 -4.074636 -0.261477

C -1.485639 -3.333159 0.601437

C -2.486576 -3.722141 1.654006

H -5.994477 -1.165250 1.861791

H -7.586393 0.582947 1.115133

H -7.092543 1.898058 -0.928147

H -5.025409 1.474431 -2.202790

H -3.926793 -1.579165 0.583088

H -2.604347 -1.256667 -1.421096

H -3.220041 -0.252901 -2.723623

H -2.533456 1.785868 -1.425361

H 1.865468 1.929818 0.010955

H -0.245464 -1.988288 -1.153900

H -0.846449 1.157970 -3.102321

H -2.404031 3.501795 1.758591

H -2.565627 3.635734 0.012664

H -3.288520 2.274980 0.856664

H 0.454390 4.114197 2.469766

H 1.170852 4.439350 0.908999

H -0.565481 4.678375 1.137099

H 3.071479 1.862137 1.763957

H 2.480599 -0.725887 -0.957945

H 4.216604 -2.440223 -0.866564

H 3.828708 -2.855631 0.797276

H 4.792150 -0.864642 1.672934

H 6.457356 -2.589886 0.977289

H 6.622574 -1.803882 -0.598008

H 7.147713 -0.967479 0.871150

H 5.148018 1.233453 -3.127977

H 3.763914 1.683933 -2.092423

H 5.401595 2.284625 -1.702981

H -2.775070 -2.878167 2.275132

H -3.363609 -4.154406 1.172356

H -2.041021 -4.494994 2.282476

### Table S16. Geometry data of conformers of structure 2b.

YYY-148b-c3 , delta G = 0.0000 kcal/mol, population = 74.24 %

C 6.481128 1.345631 1.191108

C 6.541986 0.576812 2.350462

C 5.579217 -0.401163 2.577576

C 4.560798 -0.607478 1.651502

C 4.488160 0.158771 0.487292

C 5.461362 1.137230 0.269568

C 3.350439 -0.019678 -0.482827

C 2.246902 1.025370 -0.254413

C 1.023505 0.928849 -1.202265

C 0.004701 0.047627 -0.448026

C -1.379607 0.311412 -1.069432

C -1.868619 1.677956 -0.634741

C -0.812962 2.685887 -1.069859

C 0.437831 2.327812 -1.408694

C 0.157018 -1.502718 -0.525507

C -1.226061 -2.087045 -0.767461

C -2.207601 -0.963312 -1.063319

N 1.599685 0.891450 1.039530

C 0.302738 0.512614 1.009055

O -0.432478 0.529015 1.984670

C 1.412626 3.325038 -1.995344

C -1.294352 4.110157 -1.139963

H 1.312017 0.493694 -2.158203

H -1.174902 0.432021 -2.139355

O -3.117225 2.013404 -1.254184

C -2.439019 -1.882018 0.107190

C -2.562817 -1.519148 1.564204

C -4.041867 -1.312885 1.969912

C -4.696154 -0.365091 0.990843

C -4.149971 -0.774704 3.397700

O -4.482186 0.830544 0.932404

O -5.533371 -0.993821 0.154047

C -6.145277 -0.191299 -0.878129

H -2.892801 -1.053446 -1.896130

H -1.216922 -2.996343 -1.353677

O 0.955754 -1.818097 -1.700991

O 1.640729 -3.759866 -0.780267

C 1.643230 -2.978188 -1.702975

C 2.407721 -3.142969 -2.985255

H 7.230688 2.104587 1.003702

H 7.336483 0.736359 3.068536

H 5.621403 -1.006462 3.474608

H 3.814777 -1.372378 1.831359

H 5.421969 1.736872 -0.632995

H 3.707464 0.087179 -1.509920

H 2.934733 -1.019503 -0.379305

H 2.708054 2.009934 -0.318822

H -2.000728 1.719209 0.449282

H 0.664870 -1.920007 0.341545

H 2.012297 1.219790 1.899210

H 0.922960 3.995139 -2.701192

H 1.886417 3.948702 -1.231022

H 2.212221 2.810135 -2.531491

H -1.851656 4.298482 -2.061281

H -1.983046 4.309836 -0.317028

H -0.475768 4.825261 -1.081669

H -3.802284 1.730150 -0.629184

H -3.178099 -2.632963 -0.162468

H -1.998800 -0.625144 1.802045

H -2.166625 -2.327464 2.185907

H -4.566442 -2.266827 1.891612

H -3.639098 -1.450041 4.085608

H -3.683369 0.207836 3.470419

H -5.190773 -0.687874 3.714885

H -6.681480 0.648654 -0.439958

H -5.385084 0.176063 -1.566618

H -6.830943 -0.856582 -1.393480

H 3.111327 -2.317376 -3.102614

H 2.945008 -4.087052 -2.974495

H 1.721670 -3.112970 -3.832672

YYY-148b-c1 , delta G = 0.6263 kcal/mol, population = 25.76 %

C 5.182016 -0.257787 2.856694

C 6.133236 0.748817 2.724689

C 6.166621 1.515128 1.562698

C 5.252330 1.275597 0.542918

C 4.292771 0.267656 0.664058

C 4.269491 -0.494738 1.833048

C 3.267391 0.047505 -0.416127

C 2.093359 1.030767 -0.291519

C 0.960341 0.855193 -1.336422

C -0.061318 -0.081510 -0.656074

C -1.404020 0.099976 -1.378559

C -2.003078 1.438761 -1.009309

C -0.980591 2.504467 -1.377171

C 0.312436 2.216127 -1.607474

C 0.180219 -1.625081 -0.711490

C -1.146310 -2.286400 -1.065800

C -2.166010 -1.209321 -1.397088

N 1.348268 0.872046 0.946749

C 0.083544 0.417405 0.810432

O -0.741792 0.413356 1.712212

C 1.275036 3.260353 -2.130742

C -1.542464 3.894326 -1.504091

H 1.352191 0.432741 -2.260652

H -1.131050 0.213744 -2.433963

O -3.212903 1.698139 -1.728048

C -2.412232 -2.150959 -0.251964

C -2.578807 -1.904620 1.224518

C -4.042127 -1.575731 1.617107

C -4.427282 -0.206356 1.097635

C -4.248411 -1.730883 3.124136

O -4.814246 0.009551 -0.036939

O -4.291105 0.761990 2.009638

C -4.661330 2.098156 1.612029

H -2.819190 -1.328614 -2.251283

H -1.044399 -3.182436 -1.663367

O 1.096795 -1.896614 -1.808052

O 1.762692 -3.820960 -0.838261

C 1.823061 -3.031846 -1.752504

C 2.704846 -3.161363 -2.961316

H 5.151451 -0.861511 3.755266

H 6.845981 0.932621 3.518737

H 6.907860 2.296646 1.449533

H 5.286904 1.873477 -0.360989

H 3.532543 -1.281925 1.938995

H 3.718036 0.187205 -1.401751

H 2.896035 -0.973443 -0.364846

H 2.503184 2.039220 -0.329023

H -2.208072 1.483721 0.065313

H 0.632114 -2.010973 0.200326

H 1.663215 1.242388 1.830359

H 0.809946 3.886522 -2.891230

H 1.643903 3.924334 -1.343230

H 2.146259 2.784763 -2.585048

H -2.123355 4.005344 -2.422682

H -2.231222 4.092083 -0.679350

H -0.768108 4.658088 -1.487942

H -3.901872 1.156058 -1.309444

H -3.114105 -2.915608 -0.577854

H -1.926142 -1.121279 1.591984

H -2.315209 -2.826863 1.751259

H -4.699517 -2.270156 1.087412

H -5.263118 -1.462518 3.421322

H -4.073817 -2.769879 3.408258

H -3.553799 -1.102610 3.681512

H -4.520706 2.710127 2.497404

H -4.024874 2.450265 0.802846

H -5.701419 2.119732 1.290157

H 3.317674 -4.054194 -2.875110

H 2.089384 -3.219963 -3.860384

H 3.339885 -2.279619 -3.054430

### Table S17. Geometry data of conformers of structure 2c.

YYY-148c-c3 , delta G = 0.0000 kcal/mol, population = 52.21 %

C -6.096363 0.528092 1.929856

C -7.125285 -0.125597 1.259452

C -6.915163 -0.591763 -0.035548

C -5.682539 -0.406374 -0.651742

C -4.641840 0.248346 0.011354

C -4.864866 0.713051 1.308815

C -3.288372 0.385254 -0.632895

C -2.429174 -0.877282 -0.443652

C -0.972262 -0.720231 -0.960683

C -0.180343 -0.292613 0.276032

C 1.289901 -0.710581 0.199252

C 1.410109 -2.219782 0.164722

C 0.535536 -2.753669 -0.966492

C -0.471054 -2.049028 -1.506714

C -0.106408 1.208281 0.657807

C 1.140988 1.316256 1.526683

C 1.985336 0.076886 1.300479

N -2.206851 -1.214708 0.955330

C -0.965367 -0.964044 1.426588

O -0.571976 -1.185140 2.561884

C -1.250433 -2.554884 -2.701583

C 0.908478 -4.131446 -1.445917

H -0.935721 0.037093 -1.744556

H 1.652348 -0.372977 -0.775243

O 2.769942 -2.640093 0.019879

C 2.535649 1.441553 0.956158

C 2.898168 1.916356 -0.433113

C 4.392148 1.697932 -0.781049

C 4.706865 0.222668 -0.639361

C 5.330134 2.624419 -0.009365

O 4.240934 -0.630049 -1.375032

O 5.517162 -0.054854 0.381213

C 5.836222 -1.445796 0.617725

H 2.402079 -0.464782 2.137480

H 0.984656 1.707378 2.521468

O -0.065287 1.983563 -0.567587

O -0.480468 3.869468 0.591378

C -0.264258 3.311549 -0.459259

C -0.185947 3.977431 -1.803607

H -6.252243 0.897026 2.936020

H -8.084809 -0.267818 1.740437

H -7.712464 -1.097461 -0.566120

H -5.525720 -0.770471 -1.660945

H -4.067176 1.223261 1.835467

H -3.395138 0.551973 -1.707375

H -2.763948 1.248843 -0.222618

H -2.935129 -1.708457 -0.933945

H 1.089276 -2.652685 1.115751

H -0.977039 1.539443 1.219459

H -2.931930 -1.573362 1.557874

H -1.794940 -1.738772 -3.180118

H -0.594132 -2.995622 -3.451848

H -1.986405 -3.316688 -2.427092

H 1.190438 -4.758623 -0.597941

H 0.091910 -4.619179 -1.974657

H 1.777344 -4.103544 -2.108942

H 3.202968 -2.078437 -0.644749

H 3.221081 1.815781 1.708712

H 2.288534 1.431878 -1.191112

H 2.701849 2.988241 -0.515430

H 4.495354 1.904306 -1.849442

H 6.367228 2.475648 -0.310996

H 5.061953 3.662447 -0.213299

H 5.272863 2.461443 1.065838

H 6.263140 -1.890135 -0.279908

H 6.563355 -1.437982 1.423642

H 4.939068 -1.988316 0.908658

H 0.781690 3.772871 -2.263712

H -0.954707 3.570346 -2.462132

H -0.325321 5.048998 -1.692156

YYY-148c-c11-gfn0 , delta G = 0.3709 kcal/mol, population = 27.90 %

C -6.172666 0.617784 1.603028

C -7.138514 -0.105872 0.911162

C -6.823429 -0.668028 -0.323055

C -5.549704 -0.508427 -0.857186

C -4.571189 0.215055 -0.171091

C -4.899358 0.776183 1.064205

C -3.175099 0.329215 -0.721674

C -2.326593 -0.914511 -0.405749

C -0.838788 -0.779676 -0.834868

C -0.135978 -0.265778 0.420958

C 1.346431 -0.646545 0.460919

C 1.501885 -2.151092 0.515906

C 0.686995 -2.785173 -0.600259

C -0.296911 -2.140969 -1.245671

C -0.122383 1.252058 0.731426

C 1.043692 1.425022 1.696372

C 1.931996 0.199788 1.586436

N -2.193167 -1.173687 1.021087

C -0.983187 -0.894451 1.554925

O -0.658453 -1.060518 2.720328

C -1.008662 -2.754453 -2.431751

C 1.093813 -4.194467 -0.942717

H -0.751751 -0.079096 -1.666145

H 1.771730 -0.340968 -0.499811

O 2.865554 -2.561586 0.331008

C 2.483210 1.558812 1.247999

C 2.956304 2.021270 -0.110718

C 4.458006 1.753050 -0.387994

C 4.678242 0.268491 -0.578728

C 5.387269 2.313973 0.687342

O 4.924392 -0.522401 0.309998

O 4.535607 -0.088096 -1.862454

C 4.706030 -1.489609 -2.183751

H 2.280232 -0.302415 2.478241

H 0.793353 1.841500 2.661109

O 0.008167 1.968899 -0.522919

O -0.585181 3.889940 0.492652

C -0.240848 3.293279 -0.500852

C -0.022285 3.903496 -1.855773

H -6.410155 1.062104 2.561656

H -8.130402 -0.227740 1.327754

H -7.571333 -1.227870 -0.870893

H -5.312292 -0.946276 -1.820323

H -4.151958 1.343244 1.606246

H -3.204302 0.438101 -1.808494

H -2.686897 1.217552 -0.320489

H -2.798503 -1.774264 -0.879947

H 1.157318 -2.526208 1.487418

H -1.042777 1.590397 1.201595

H -2.950450 -1.514225 1.593626

H -0.313446 -3.261267 -3.100386

H -1.763243 -3.487704 -2.131595

H -1.521067 -1.984534 -3.011697

H 1.326348 -4.749314 -0.031498

H 0.311631 -4.730680 -1.475944

H 2.000268 -4.209094 -1.552573

H 3.466466 -1.882294 0.670392

H 3.087126 1.971698 2.047830

H 2.365017 1.587815 -0.914582

H 2.812309 3.103331 -0.175779

H 4.684042 2.228643 -1.343564

H 6.430054 2.236044 0.376267

H 5.162221 3.367484 0.862513

H 5.279216 1.774022 1.626604

H 5.704161 -1.816775 -1.895702

H 3.958353 -2.086833 -1.666369

H 4.582210 -1.550670 -3.260747

H -0.545242 3.329040 -2.620313

H -0.367126 4.933728 -1.855334

H 1.042733 3.876383 -2.094781

YYY-148c-c8-gfn0 , delta G = 0.7430 kcal/mol, population = 14.88 %

C -4.457558 1.440279 -2.099882

C -5.367359 0.494209 -2.559673

C -5.664400 -0.613097 -1.769343

C -5.050631 -0.773220 -0.532328

C -4.129119 0.166268 -0.063129

C -3.845679 1.273919 -0.861591

C -3.418900 -0.042497 1.248372

C -2.397322 -1.191561 1.169502

C -1.186685 -0.918816 0.232523

C -0.101818 -0.376319 1.162100

C 1.313325 -0.679640 0.657558

C 1.540511 -2.175625 0.606009

C 0.421655 -2.823839 -0.195790

C -0.754700 -2.220916 -0.424091

C -0.044522 1.139941 1.471774

C 1.378234 1.362756 1.971525

C 2.222430 0.182413 1.527654

N -1.734766 -1.435232 2.442183

C -0.438761 -1.057448 2.512048

O 0.293267 -1.193647 3.480040

C -1.805492 -2.828025 -1.329096

C 0.751553 -4.189815 -0.737022

H -1.462058 -0.196199 -0.534423

H 1.353939 -0.338885 -0.381099

O 2.778935 -2.515405 -0.035769

C 2.564707 1.569730 1.054316

C 2.522030 2.068061 -0.371617

C 3.842998 1.867113 -1.158222

C 4.038693 0.395172 -1.448236

C 5.066823 2.460431 -0.462324

O 4.616703 -0.389879 -0.723295

O 3.461919 0.042527 -2.604938

C 3.558905 -1.347312 -2.999283

H 2.880286 -0.310260 2.229839

H 1.463448 1.757198 2.973465

O -0.381627 1.857429 0.255343

O -0.603150 3.770784 1.422913

C -0.614361 3.179155 0.368596

C -0.862755 3.810605 -0.972806

H -4.223726 2.308455 -2.703657

H -5.845304 0.619697 -3.522984

H -6.376818 -1.351312 -2.116156

H -5.291176 -1.637941 0.075641

H -3.138347 2.011996 -0.508222

H -2.915831 0.875685 1.553468

H -4.140899 -0.285539 2.032653

H -2.925985 -2.093089 0.862115

H 1.557656 -2.584156 1.623862

H -0.759590 1.441775 2.233653

H -2.195770 -1.855043 3.235327

H -2.547093 -2.079801 -1.611939

H -1.365153 -3.222370 -2.244810

H -2.345415 -3.649914 -0.849344

H -0.137676 -4.738100 -1.040556

H 1.428267 -4.126743 -1.592403

H 1.271143 -4.778072 0.022838

H 3.429079 -1.810977 0.100130

H 3.391358 2.001638 1.606644

H 1.706409 1.619165 -0.933888

H 2.327022 3.144004 -0.355921

H 3.703085 2.359748 -2.121677

H 4.876110 3.501485 -0.195831

H 5.317557 1.909850 0.443036

H 5.936799 2.431930 -1.120113

H 3.067588 -1.980720 -2.263956

H 3.061822 -1.403715 -3.962962

H 4.605687 -1.633357 -3.092943

H -1.196002 3.079614 -1.705882

H -1.591501 4.611896 -0.868899

H 0.074233 4.248363 -1.324707

YYY-148c-c4 , delta G = 1.3855 kcal/mol, population = 5.02 %

C -4.238001 2.275195 -0.492086

C -4.367955 2.708850 0.822294

C -4.357326 1.777819 1.855811

C -4.216176 0.423511 1.574314

C -4.069475 -0.023997 0.258562

C -4.084607 0.921159 -0.768415

C -3.915234 -1.489040 -0.046094

C -2.530530 -2.106704 0.222819

C -1.318884 -1.493022 -0.552118

C -0.516079 -0.687418 0.481897

C 1.002555 -0.827909 0.286425

C 1.426532 -2.278436 0.394490

C 0.655456 -3.069309 -0.658862

C -0.523366 -2.649246 -1.141732

C -0.680890 0.852374 0.566403

C 0.570014 1.313355 1.311174

C 1.610814 0.217248 1.208812

N -2.097487 -1.995739 1.603073

C -0.990184 -1.259917 1.836734

O -0.486380 -1.078836 2.935561

C -1.240425 -3.372293 -2.262018

C 1.327723 -4.331624 -1.127902

H -1.676040 -0.848538 -1.354483

H 1.198355 -0.550569 -0.751948

O 2.838423 -2.437507 0.249599

C 1.896372 1.587263 0.638276

C 2.128218 1.906571 -0.821438

C 3.626702 1.917037 -1.212511

C 4.209892 0.551208 -0.915631

C 4.399725 3.085761 -0.604262

O 3.868116 -0.459123 -1.506159

O 5.111609 0.554866 0.064742

C 5.695215 -0.715683 0.436186

H 2.149839 -0.116629 2.083267

H 0.389331 1.819837 2.247663

O -0.796770 1.343359 -0.794199

O -1.106953 3.447698 -0.050334

C -1.027033 2.657498 -0.960867

C -1.150994 2.995409 -2.420004

H -4.249084 2.993450 -1.302307

H -4.472308 3.763897 1.039435

H -4.461847 2.105437 2.882557

H -4.222551 -0.293084 2.385636

H -3.982197 0.590568 -1.795286

H -4.643407 -2.059414 0.537967

H -4.148316 -1.674356 -1.096933

H -2.618861 -3.164038 -0.029984

H 1.208656 -2.669025 1.392938

H -1.578684 1.156075 1.097842

H -2.577840 -2.448523 2.366262

H -0.549526 -3.693782 -3.040733

H -1.772460 -4.262819 -1.913664

H -1.980002 -2.718599 -2.727827

H 1.748374 -4.868959 -0.275231

H 0.638965 -4.996807 -1.644321

H 2.165324 -4.117940 -1.797111

H 3.138776 -1.903889 -0.505656

H 2.529462 2.181263 1.288621

H 1.604877 1.208574 -1.469877

H 1.732288 2.900559 -1.042821

H 3.661134 2.003081 -2.301876

H 3.936087 4.024942 -0.910851

H 4.408272 3.048873 0.484099

H 5.436148 3.091599 -0.942498

H 4.930244 -1.368613 0.850956

H 6.153151 -1.185587 -0.432739

H 6.446670 -0.476533 1.182072

H -0.189836 2.838743 -2.913099

H -1.875827 2.337138 -2.898591

H -1.452577 4.033089 -2.533048

### Table S18. Geometry data of conformers of structure 2d.

YYY-148d-c1 , delta G = 0.0000 kcal/mol, population = 28.46 %

C -5.087266 1.329124 -1.790695

C -6.140486 0.431219 -1.929163

C -6.351873 -0.536256 -0.950343

C -5.510366 -0.606334 0.153668

C -4.444995 0.285030 0.300241

C -4.247657 1.254138 -0.683573

C -3.495592 0.149558 1.460834

C -2.541276 -1.043639 1.269190

C -1.500916 -0.851135 0.129853

C -0.249544 -0.333948 0.841957

C 1.035875 -0.734235 0.114270

C 1.186661 -2.235829 0.070176

C -0.074426 -2.832411 -0.546341

C -1.246920 -2.179381 -0.568813

C -0.065672 1.190717 1.061326

C 1.436014 1.362198 1.276715

C 2.122008 0.127548 0.736572

N -1.681648 -1.264324 2.422769

C -0.378519 -0.947424 2.254757

O 0.504220 -1.087285 3.087464

C -2.466922 -2.738391 -1.270125

C 0.104889 -4.206269 -1.133306

H -1.875334 -0.131605 -0.596663

H 0.905972 -0.422995 -0.928892

O 2.362727 -2.629613 -0.652324

C 2.332204 1.456795 0.070718

C 3.673988 2.137690 0.149531

C 4.593581 1.717737 -1.018751

C 4.662949 0.204537 -1.091916

C 5.972541 2.371854 -0.914279

O 4.046735 -0.474830 -1.890310

O 5.460463 -0.311755 -0.151060

C 5.579627 -1.752200 -0.104890

H 2.929188 -0.333732 1.287493

H 1.757213 1.826906 2.197854

O -0.553120 1.863815 -0.129460

O -0.418568 3.850525 0.923442

C -0.647288 3.206951 -0.074064

C -1.043325 3.781661 -1.404851

H -4.918205 2.088514 -2.544123

H -6.794535 0.486875 -2.790140

H -7.173627 -1.234943 -1.046936

H -5.681497 -1.362345 0.911664

H -3.426919 1.952395 -0.580627

H -2.916712 1.065316 1.586806

H -4.054547 -0.007307 2.386856

H -3.143137 -1.934854 1.095126

H 1.327837 -2.637987 1.077811

H -0.627299 1.571754 1.910965

H -2.017240 -1.633347 3.299798

H -2.211902 -3.163101 -2.240984

H -2.955898 -3.526789 -0.689936

H -3.208823 -1.955773 -1.434419

H 0.717710 -4.820218 -0.469103

H -0.843879 -4.715589 -1.288223

H 0.635243 -4.165677 -2.087905

H 2.627411 -1.926689 -1.264157

H 1.833053 1.577576 -0.885387

H 3.557013 3.224102 0.110941

H 4.162412 1.900323 1.098176

H 4.115100 2.022854 -1.951226

H 6.615793 2.080469 -1.746263

H 5.866026 3.457754 -0.932913

H 6.467383 2.090648 0.015209

H 5.989074 -2.121551 -1.044193

H 6.263115 -1.955520 0.713771

H 4.607027 -2.206307 0.074335

H -1.529881 4.742981 -1.259346

H -0.137113 3.935710 -1.995510

H -1.689907 3.101797 -1.956443

YYY-148d-c2 , delta G = 0.1173 kcal/mol, population = 23.34 %

C -6.992467 -0.503947 0.586110

C -7.022239 -0.001056 1.883983

C -5.907794 0.659948 2.390496

C -4.770718 0.815838 1.603521

C -4.728839 0.315024 0.301089

C -5.853711 -0.347328 -0.196350

C -3.475996 0.427311 -0.525517

C -2.608624 -0.840904 -0.439796

C -1.229566 -0.693411 -1.139892

C -0.284635 -0.265899 -0.015210

C 1.158842 -0.699760 -0.278191

C 1.266235 -2.204043 -0.332792

C 0.265064 -2.728544 -1.356185

C -0.806805 -2.022995 -1.750466

C -0.142867 1.237338 0.342180

C 1.201847 1.332340 1.056999

C 1.993447 0.089465 0.716328

N -2.211264 -1.173503 0.921380

C -0.919750 -0.924476 1.230742

O -0.385724 -1.139835 2.308308

C -1.742533 -2.523763 -2.829938

C 0.579177 -4.101296 -1.887132

H -1.290374 0.063288 -1.922757

H 1.404733 -0.350165 -1.288115

O 2.602352 -2.630776 -0.637681

C 2.453636 1.433098 0.227779

C 3.711220 2.048189 0.784314

C 4.954520 1.628774 -0.030471

C 5.000072 0.117321 -0.146756

C 6.239443 2.219411 0.552997

O 4.670887 -0.506610 -1.137241

O 5.417940 -0.465803 0.981664

C 5.478396 -1.910683 0.993887

H 2.554345 -0.428250 1.481572

H 1.206198 1.745925 2.055285

O -0.191864 1.990313 -0.896995

O -0.399435 3.907701 0.266341

C -0.299447 3.329975 -0.790903

C -0.269983 3.982011 -2.143849

H -7.857625 -1.015202 0.182325

H -7.908737 -0.120491 2.493996

H -5.923575 1.057244 3.397813

H -3.906212 1.332982 2.002874

H -5.838170 -0.738719 -1.207388

H -3.728678 0.580734 -1.577393

H -2.893220 1.291506 -0.204560

H -3.178351 -1.670942 -0.856356

H 1.047435 -2.639281 0.646738

H -0.945149 1.598761 0.980892

H -2.856398 -1.522177 1.613962

H -1.196762 -2.965638 -3.663126

H -2.437078 -3.282678 -2.457510

H -2.344215 -1.704970 -3.228799

H 1.399009 -4.070995 -2.609019

H 0.913292 -4.748639 -1.073036

H -0.280859 -4.569129 -2.361339

H 3.079838 -1.923448 -1.096104

H 2.306966 1.612890 -0.832501

H 3.648053 3.139514 0.763697

H 3.845247 1.753129 1.828211

H 4.822551 1.985149 -1.053800

H 6.178404 3.309015 0.546574

H 6.389241 1.889839 1.581080

H 7.113305 1.926255 -0.031178

H 5.834477 -2.172589 1.985497

H 4.492693 -2.332278 0.807340

H 6.176052 -2.259845 0.233757

H -0.507127 5.037966 -2.048322

H 0.726767 3.870051 -2.575051

H -0.976132 3.494862 -2.816268

YYY-148d-c4 , delta G = 0.2278 kcal/mol, population = 19.37 %

C -6.163523 -0.550649 -1.203900

C -5.894853 0.428796 -2.156451

C -4.876315 1.347741 -1.926958

C -4.128480 1.281013 -0.755772

C -4.382912 0.300460 0.203121

C -5.413668 -0.611822 -0.034895

C -3.527692 0.186154 1.437281

C -2.571593 -1.018342 1.359734

C -1.477373 -0.897900 0.262252

C -0.268613 -0.314984 0.995777

C 1.049352 -0.741203 0.349352

C 1.231804 -2.237719 0.397068

C 0.006158 -2.906542 -0.196924

C -1.173864 -2.276918 -0.313313

C -0.097730 1.222950 1.119105

C 1.387825 1.407749 1.427510

C 2.097167 0.149988 0.988872

N -1.765595 -1.166292 2.562839

C -0.459042 -0.841177 2.437312

O 0.382838 -0.918188 3.318991

C -2.358220 -2.919895 -1.003069

C 0.224368 -4.324737 -0.651350

H -1.821468 -0.242642 -0.536697

H 0.973024 -0.490720 -0.713436

O 2.378338 -2.635333 -0.377009

C 2.377891 1.446694 0.289921

C 3.734150 2.081451 0.477799

C 4.697625 1.739107 -0.674168

C 4.645403 0.261153 -1.017000

C 6.139850 2.140128 -0.341787

O 4.834719 -0.649058 -0.236380

O 4.377073 0.068534 -2.317034

C 4.338835 -1.299262 -2.785703

H 2.869683 -0.284099 1.606978

H 1.643646 1.906968 2.351172

O -0.496529 1.799022 -0.152037

O -0.410566 3.867542 0.735503

C -0.579044 3.142284 -0.216820

C -0.882774 3.600020 -1.615715

H -6.959194 -1.265988 -1.371219

H -6.478102 0.476862 -3.067304

H -4.663097 2.116883 -2.658976

H -3.335818 1.996908 -0.581806

H -5.630875 -1.376824 0.701766

H -2.950630 1.100456 1.580793

H -4.157271 0.063298 2.322550

H -3.167512 -1.918164 1.213628

H 1.376350 -2.574571 1.431466

H -0.716000 1.666682 1.895887

H -2.138746 -1.485379 3.444186

H -2.852579 -3.665453 -0.372957

H -3.107820 -2.170167 -1.259592

H -2.062350 -3.420935 -1.924518

H 0.801113 -4.871507 0.098572

H -0.711144 -4.855492 -0.812888

H 0.807452 -4.360757 -1.574606

H 3.149750 -2.148087 -0.051708

H 1.958475 1.554638 -0.705773

H 3.654957 3.170600 0.532106

H 4.164521 1.745131 1.424571

H 4.374992 2.278154 -1.566180

H 6.505922 1.573439 0.515616

H 6.808287 1.961576 -1.185610

H 6.180367 3.202170 -0.094582

H 3.501961 -1.826842 -2.331987

H 4.212110 -1.228843 -3.861801

H 5.271544 -1.806200 -2.543056

H -1.314918 4.597162 -1.590553

H 0.052812 3.636859 -2.178743

H -1.548759 2.904143 -2.123165

YYY-148d-c7-gfn0 , delta G = 0.9469 kcal/mol, population = 5.75 %

C 4.412861 1.909041 1.726298

C 5.592486 1.189904 1.884528

C 5.935953 0.220136 0.946153

C 5.099605 -0.032707 -0.134834

C 3.905376 0.674460 -0.298047

C 3.579179 1.650997 0.642433

C 2.974488 0.358796 -1.440143

C 2.376538 -1.055162 -1.327472

C 1.411095 -1.259307 -0.124028

C 0.018273 -1.026211 -0.708543

C -1.066569 -1.826100 0.017035

C -0.807531 -3.310561 -0.098184

C 0.606653 -3.611265 0.363885

C 1.565445 -2.673918 0.415836

C -0.568642 0.407525 -0.724918

C -2.067704 0.189499 -0.886515

C -2.381280 -1.236959 -0.487965

N 1.516610 -1.383155 -2.455346

C 0.192557 -1.431534 -2.189819

O -0.686460 -1.708498 -2.992543

C 2.939178 -2.966890 0.979303

C 0.845872 -5.042050 0.766935

H 1.642762 -0.543260 0.664350

H -0.968035 -1.596811 1.082233

O -1.708394 -4.056475 0.742502

C -2.928281 -0.102856 0.322866

C -4.397348 0.245060 0.260259

C -4.741886 1.594390 0.934830

C -3.821251 2.682151 0.417030

C -4.693123 1.513481 2.458330

O -2.975952 3.252349 1.070421

O -4.037180 2.937158 -0.883715

C -3.160525 3.907297 -1.498831

H -3.042586 -1.834980 -1.101982

H -2.546303 0.640669 -1.743716

O -0.246913 1.047295 0.535160

O 0.078734 3.033103 -0.484306

C 0.009758 2.371046 0.527072

C 0.183858 2.897176 1.919575

H 4.140474 2.672405 2.444538

H 6.242713 1.386606 2.727550

H 6.857021 -0.338704 1.056271

H 5.378761 -0.788513 -0.859800

H 2.671004 2.224954 0.516292

H 2.171493 1.095861 -1.483259

H 3.512418 0.421293 -2.390354

H 3.196113 -1.771070 -1.283894

H -0.939287 -3.645755 -1.134549

H -0.169286 1.023344 -1.525069

H 1.866421 -1.556881 -3.385646

H 3.561220 -3.537833 0.283270

H 3.470528 -2.039972 1.197027

H 2.879061 -3.540867 1.903903

H 0.351552 -5.716074 0.063831

H 1.904692 -5.290820 0.788840

H 0.420488 -5.256015 1.750401

H -2.595159 -3.956783 0.378250

H -2.459368 0.045075 1.288419

H -4.720263 0.266794 -0.782077

H -4.983472 -0.531187 0.760035

H -5.753962 1.863811 0.621754

H -5.398996 0.759583 2.809990

H -3.697836 1.244154 2.811773

H -4.955997 2.469274 2.911899

H -2.121442 3.602733 -1.388262

H -3.449441 3.933172 -2.545266

H -3.303069 4.885226 -1.040668

H 0.652110 2.156755 2.565580

H 0.767863 3.814114 1.895989

H -0.811432 3.122001 2.307824

YYY-148d-c5 , delta G = 0.9689 kcal/mol, population = 5.54 %

C -7.037952 -0.457008 0.383017

C -7.143667 0.159534 1.626924

C -6.060044 0.866782 2.137753

C -4.878013 0.955472 1.408735

C -4.759835 0.340306 0.161350

C -5.854448 -0.367221 -0.341297

C -3.459971 0.382029 -0.595738

C -2.608117 -0.876534 -0.354327

C -1.194688 -0.796830 -0.994869

C -0.303685 -0.274103 0.132491

C 1.145364 -0.726347 -0.032061

C 1.269923 -2.228294 0.025881

C 0.299336 -2.849866 -0.960331

C -0.754329 -2.181638 -1.456328

C -0.156747 1.253508 0.370087

C 1.136620 1.380548 1.174444

C 1.925350 0.112239 0.962176

N -2.280989 -1.087963 1.049024

C -1.001684 -0.825703 1.397076

O -0.521217 -0.958983 2.512351

C -1.655683 -2.784794 -2.512422

C 0.618220 -4.271849 -1.337033

H -1.210853 -0.115548 -1.846534

H 1.447560 -0.447327 -1.046708

O 2.600649 -2.642066 -0.334851

C 2.460735 1.416171 0.451151

C 3.680141 1.996160 1.125298

C 4.980897 1.643632 0.379417

C 5.014950 0.179470 -0.020804

C 6.219466 1.973609 1.221632

O 4.879543 -0.760961 0.735022

O 5.238948 0.036216 -1.335467

C 5.349411 -1.314447 -1.840645

H 2.425731 -0.365801 1.791674

H 1.069434 1.846185 2.147451

O -0.094084 1.889651 -0.932029

O -0.339154 3.911381 0.030466

C -0.173466 3.235105 -0.957864

C -0.019943 3.756700 -2.358378

H -7.878896 -1.004684 -0.024156

H -8.065186 0.092252 2.191311

H -6.134905 1.352351 3.102828

H -4.037437 1.509376 1.809480

H -5.779149 -0.847024 -1.310699

H -3.649320 0.448604 -1.669823

H -2.890457 1.267949 -0.312123

H -3.163726 -1.738137 -0.722736

H 1.053980 -2.593186 1.038062

H -0.994270 1.686415 0.912154

H -2.958723 -1.384470 1.734679

H -1.084898 -3.306621 -3.279972

H -2.367266 -3.501958 -2.092585

H -2.237780 -2.006362 -3.008891

H -0.221507 -4.773593 -1.812445

H 1.478003 -4.320521 -2.009220

H 0.892332 -4.839786 -0.444730

H 3.228955 -2.186060 0.244977

H 2.417297 1.564534 -0.623603

H 3.617041 3.086106 1.184277

H 3.736877 1.624361 2.151544

H 5.016620 2.220589 -0.545802

H 7.140525 1.794362 0.664681

H 6.197834 3.024666 1.513960

H 6.238231 1.365234 2.127115

H 4.395190 -1.829255 -1.747121

H 5.627747 -1.206916 -2.884631

H 6.118143 -1.858710 -1.294133

H -0.669858 3.210565 -3.041930

H -0.251574 4.817959 -2.383664

H 1.009620 3.601864 -2.687408

YYY-148d-c2-gfn0 , delta G = 1.1496 kcal/mol, population = 4.08 %

C 5.914284 0.272286 0.990664

C 5.559503 1.235230 1.931867

C 4.377657 1.949680 1.769030

C 3.552956 1.693625 0.677824

C 3.890417 0.723774 -0.265619

C 5.086952 0.021502 -0.097732

C 2.969626 0.411488 -1.416958

C 2.390211 -1.011578 -1.327225

C 1.424696 -1.248117 -0.129484

C 0.030165 -1.024264 -0.713972

C -1.049818 -1.842799 -0.000609

C -0.783696 -3.324001 -0.137211

C 0.647219 -3.620273 0.292153

C 1.594463 -2.672669 0.377425

C -0.571775 0.403348 -0.721744

C -2.068519 0.171887 -0.885844

C -2.368689 -1.259332 -0.493864

N 1.537824 -1.335947 -2.461583

C 0.214181 -1.414291 -2.198462

O -0.657737 -1.702277 -3.004063

C 2.969441 -2.969695 0.934486

C 0.914839 -5.061798 0.641797

H 1.646235 -0.544173 0.672634

H -0.945455 -1.617954 1.067826

O -1.729458 -4.091370 0.626234

C -2.922316 -0.135290 0.325277

C -4.396055 0.193824 0.267937

C -4.758179 1.534223 0.950965

C -3.851822 2.636290 0.438887

C -4.707436 1.444767 2.473857

O -3.010719 3.210997 1.094080

O -4.074120 2.897964 -0.859238

C -3.207418 3.878662 -1.471449

H -3.029139 -1.869333 -1.094510

H -2.551842 0.626663 -1.738524

O -0.255641 1.038818 0.542950

O 0.041479 3.035681 -0.463622

C -0.017919 2.366186 0.543371

C 0.148924 2.886501 1.939055

H 6.837421 -0.282484 1.104077

H 6.202934 1.430630 2.780409

H 4.096761 2.708177 2.489180

H 2.643278 2.264323 0.547892

H 5.375620 -0.728242 -0.825237

H 2.157680 1.138841 -1.454699

H 3.511556 0.493847 -2.363415

H 3.219572 -1.716489 -1.291652

H -0.948943 -3.652520 -1.166175

H -0.177548 1.027868 -1.517591

H 1.892816 -1.498455 -3.391896

H 3.600458 -3.505853 0.218979

H 3.489243 -2.045903 1.190562

H 2.912536 -3.582152 1.834463

H 0.593469 -5.303324 1.659056

H 0.351677 -5.718767 -0.023333

H 1.970643 -5.312370 0.556805

H -1.654621 -3.820197 1.550106

H -2.452224 0.016815 1.289913

H -4.720815 0.217282 -0.773724

H -4.970585 -0.593990 0.762879

H -5.773940 1.792280 0.640219

H -3.708659 1.184985 2.824908

H -4.981053 2.394537 2.933797

H -5.404243 0.680460 2.821161

H -3.500709 3.909077 -2.516548

H -3.355263 4.852578 -1.006424

H -2.165711 3.580925 -1.367019

H 0.720759 3.811214 1.920861

H -0.849409 3.096318 2.327867

H 0.626921 2.148894 2.581177

YYY-148d-c5-gfn0 , delta G = 1.1672 kcal/mol, population = 3.96 %

C 6.088064 0.327743 0.766679

C 5.653501 1.136146 1.813739

C 4.392343 1.719687 1.752075

C 3.570413 1.488904 0.653521

C 3.991700 0.675897 -0.398958

C 5.263343 0.101946 -0.329323

C 3.075844 0.367905 -1.553816

C 2.522896 -1.067153 -1.471025

C 1.570399 -1.319484 -0.268310

C 0.171933 -1.092049 -0.842319

C -0.903818 -1.893242 -0.100813

C -0.652731 -3.377902 -0.226452

C 0.785542 -3.686183 0.168016

C 1.743445 -2.747458 0.226735

C -0.424669 0.336925 -0.853432

C -1.921869 0.105989 -1.022482

C -2.225312 -1.308480 -0.580493

N 1.666223 -1.404105 -2.598995

C 0.344736 -1.496238 -2.324252

O -0.529676 -1.802869 -3.119850

C 3.129413 -3.055055 0.749818

C 1.047259 -5.129442 0.515237

H 1.795163 -0.625309 0.539867

H -0.778040 -1.656354 0.962815

O -1.585057 -4.127294 0.570657

C -2.765346 -0.144206 0.201009

C -4.237303 0.197994 0.148495

C -4.550110 1.683628 -0.053276

C -3.932122 2.562759 1.014667

C -6.067043 1.930453 -0.109727

O -3.482679 2.186473 2.075857

O -3.965294 3.854738 0.649151

C -3.405006 4.807019 1.571692

H -2.901878 -1.929346 -1.151370

H -2.391802 0.534958 -1.895652

O -0.074973 0.939581 0.421017

O -0.882446 2.931043 -0.252730

C -0.391913 2.234682 0.606162

C -0.084148 2.675290 2.008364

H 7.071244 -0.124838 0.803172

H 6.295150 1.312949 2.667661

H 4.047057 2.354746 2.558534

H 2.588454 1.942005 0.610865

H 5.611312 -0.527071 -1.140826

H 2.249725 1.079127 -1.579418

H 3.614406 0.469114 -2.499589

H 3.368552 -1.753010 -1.444672

H -0.847048 -3.717636 -1.246706

H -0.029208 0.970314 -1.644010

H 2.016280 -1.576088 -3.529415

H 3.090033 -3.679294 1.642390

H 3.743348 -3.582735 0.013466

H 3.657376 -2.135844 1.007350

H 2.097113 -5.392638 0.399590

H 0.753914 -5.363060 1.542747

H 0.456989 -5.782125 -0.130334

H -1.500549 -3.827300 1.484760

H -2.291752 0.050379 1.154336

H -4.706004 -0.342489 -0.678280

H -4.718568 -0.154258 1.064516

H -4.115356 2.011507 -1.000522

H -6.291122 2.976438 -0.315316

H -6.509673 1.321528 -0.899320

H -6.539176 1.653502 0.835468

H -2.329796 4.658784 1.650910

H -3.618936 5.783321 1.147114

H -3.865451 4.706853 2.553427

H 0.160641 3.735294 2.014861

H -0.984851 2.521937 2.607070

H 0.725322 2.091940 2.442071

YYY-148d-c3 , delta G = 1.2324 kcal/mol, population = 3.55 %

C -4.257404 2.341167 -0.405296

C -4.185279 2.899000 0.866130

C -4.087651 2.066394 1.976247

C -4.059115 0.685844 1.812990

C -4.115695 0.113396 0.539638

C -4.219191 0.960696 -0.564928

C -4.071764 -1.379044 0.361677

C -2.684142 -2.038348 0.471330

C -1.583007 -1.531402 -0.515802

C -0.614478 -0.686028 0.327100

C 0.851712 -0.910989 -0.075406

C 1.244661 -2.361136 0.071904

C 0.311062 -3.186211 -0.808785

C -0.917804 -2.755524 -1.132399

C -0.709814 0.863752 0.305478

C 0.652165 1.323989 0.825599

C 1.622356 0.180937 0.646450

N -2.048981 -1.846413 1.761906

C -0.901552 -1.135320 1.776143

O -0.237590 -0.890230 2.772736

C -1.820080 -3.519564 -2.077965

C 0.878487 -4.494718 -1.286910

H -2.034962 -0.930975 -1.304195

H 0.901636 -0.705081 -1.150227

O 2.621141 -2.580505 -0.260223

C 1.798273 1.450342 -0.139195

C 2.957599 2.366364 0.153936

C 4.197337 2.008343 -0.695405

C 4.511576 0.534078 -0.535839

C 5.388295 2.909952 -0.365554

O 4.224316 -0.322947 -1.349757

O 5.118835 0.267844 0.625095

C 5.442303 -1.114559 0.898988

H 2.322695 -0.074427 1.428639

H 0.659346 1.933065 1.717927

O -0.954330 1.244615 -1.073249

O -1.042227 3.421262 -0.493935

C -1.107432 2.555642 -1.334265

C -1.362444 2.780790 -2.798429

H -4.336230 2.982958 -1.273865

H -4.200243 3.973924 0.990137

H -4.034665 2.491607 2.970608

H -3.993843 0.047023 2.684452

H -4.272161 0.533364 -1.559321

H -4.717365 -1.854754 1.105528

H -4.476096 -1.646684 -0.617038

H -2.842193 -3.106481 0.317285

H 1.153956 -2.683003 1.114579

H -1.523904 1.257325 0.907306

H -2.424863 -2.224846 2.618542

H -1.267484 -3.921077 -2.926682

H -2.322207 -4.360887 -1.590729

H -2.600176 -2.866218 -2.472936

H 1.376417 -5.008776 -0.461215

H 0.113140 -5.156693 -1.685869

H 1.641280 -4.342796 -2.054330

H 2.933138 -1.890488 -0.865091

H 1.547859 1.379787 -1.192846

H 2.690190 3.404076 -0.062693

H 3.218572 2.315796 1.214292

H 3.931111 2.127242 -1.747345

H 5.126857 3.950559 -0.564901

H 5.666004 2.820573 0.684673

H 6.259442 2.657563 -0.972524

H 4.537579 -1.718940 0.912519

H 6.125409 -1.493858 0.140107

H 5.922067 -1.107517 1.872907

H -0.510175 2.426249 -3.380407

H -2.236507 2.210466 -3.114941

H -1.521577 3.839149 -2.985324

YYY-148d-c15-gfn0 , delta G = 1.3636 kcal/mol, population = 2.84 %

C 4.406053 1.905610 1.754292

C 5.589471 1.190785 1.903426

C 5.936153 0.232231 0.954758

C 5.099310 -0.013781 -0.127427

C 3.901341 0.689125 -0.281741

C 3.571913 1.654350 0.669196

C 2.970147 0.380205 -1.425488

C 2.379600 -1.037577 -1.324089

C 1.415439 -1.257993 -0.122494

C 0.020515 -1.030902 -0.704660

C -1.063693 -1.834347 0.019076

C -0.806032 -3.313319 -0.109846

C 0.622148 -3.615787 0.329598

C 1.578628 -2.677780 0.399086

C -0.570057 0.401464 -0.724272

C -2.068657 0.180612 -0.882656

C -2.378797 -1.245003 -0.478294

N 1.522073 -1.364057 -2.453983

C 0.198567 -1.431836 -2.187079

O -0.676302 -1.719016 -2.990284

C 2.953551 -2.971255 0.960323

C 0.867008 -5.052172 0.709549

H 1.642317 -0.548543 0.673204

H -0.960815 -1.612065 1.085911

O -1.764493 -3.977741 0.734891

C -2.920556 -0.111084 0.333994

C -4.391423 0.230213 0.280518

C -4.737966 1.582788 0.947602

C -3.828617 2.671271 0.412393

C -4.675414 1.514669 2.471170

O -2.983008 3.255062 1.053467

O -4.054387 2.910080 -0.889932

C -3.182677 3.872298 -1.523508

H -3.046087 -1.854167 -1.072198

H -2.550938 0.633314 -1.737034

O -0.245806 1.047505 0.532891

O 0.065454 3.031416 -0.494880

C 0.001350 2.372681 0.519144

C 0.171610 2.906737 1.909244

H 4.130991 2.660307 2.480629

H 6.240114 1.382224 2.747346

H 6.860209 -0.323058 1.057737

H 5.381199 -0.760644 -0.860589

H 2.660524 2.224680 0.550579

H 2.164204 1.114294 -1.461246

H 3.506752 0.453273 -2.375708

H 3.203901 -1.748467 -1.285451

H -0.962567 -3.641551 -1.143969

H -0.172300 1.014612 -1.527181

H 1.873274 -1.531768 -3.384808

H 3.579081 -3.530825 0.258309

H 3.480387 -2.044505 1.189486

H 2.895404 -3.555639 1.878616

H 1.926038 -5.295391 0.751654

H 0.425183 -5.292636 1.680256

H 0.408424 -5.721740 -0.025439

H -1.781399 -4.913035 0.505826

H -2.445797 0.042401 1.295879

H -4.722355 0.242827 -0.759470

H -4.969096 -0.545981 0.789859

H -5.754422 1.844072 0.641440

H -5.374157 0.759880 2.834886

H -3.675789 1.252753 2.817980

H -4.939127 2.472731 2.919629

H -3.477628 3.883339 -2.568555

H -3.323011 4.856598 -1.078413

H -2.142709 3.569858 -1.414721

H 0.635523 2.169501 2.561935

H 0.757291 3.822582 1.882322

H -0.824934 3.136222 2.291568

YYY-148d-c8-gfn0 , delta G = 1.7338 kcal/mol, population = 1.52 %

C 6.550132 0.627537 -0.350193

C 6.457349 1.328794 -1.549440

C 5.212403 1.753008 -2.002827

C 4.067435 1.474979 -1.262255

C 4.147912 0.770049 -0.060156

C 5.403519 0.350775 0.385995

C 2.901780 0.409075 0.703186

C 2.420869 -1.014649 0.373897

C 1.056946 -1.380680 1.024865

C 0.027937 -1.044235 -0.053520

C -1.246301 -1.881980 0.070550

C -0.944006 -3.350490 -0.119253

C 0.203021 -3.753374 0.798235

C 1.032328 -2.863268 1.366534

C -0.508294 0.405924 -0.172146

C -1.825558 0.256681 -0.918913

C -2.261614 -1.190616 -0.831684

N 2.128588 -1.208879 -1.039882

C 0.818206 -1.294817 -1.358546

O 0.362570 -1.485513 -2.475961

C 2.083093 -3.284408 2.370049

C 0.322584 -5.236556 1.039068

H 0.904422 -0.789755 1.929140

H -1.578514 -1.778806 1.111163

O -2.111586 -4.153886 0.119229

C -3.081332 -0.127206 -0.168034

C -4.415949 0.285997 -0.742369

C -4.971937 1.596356 -0.134811

C -3.916303 2.683715 -0.189344

C -5.488621 1.407685 1.289109

O -3.369022 3.175520 0.772633

O -3.633842 3.038804 -1.453435

C -2.584744 4.017388 -1.623912

H -2.641336 -1.707369 -1.702082

H -1.942678 0.815308 -1.836208

O -0.689634 0.951459 1.158113

O 0.058990 2.973876 0.491764

C -0.408425 2.258404 1.348760

C -0.764819 2.691517 2.738213

H 7.516271 0.299299 0.012872

H 7.349354 1.546325 -2.123307

H 5.131569 2.302868 -2.932297

H 3.100410 1.810024 -1.617825

H 5.482695 -0.193367 1.320526

H 3.090417 0.451885 1.778712

H 2.111972 1.126851 0.482406

H 3.201163 -1.713444 0.673519

H -0.687414 -3.560158 -1.160407

H 0.171129 1.061243 -0.708495

H 2.844405 -1.253186 -1.749101

H 2.436803 -2.424426 2.941460

H 1.691989 -4.013570 3.079376

H 2.957070 -3.735588 1.890781

H 0.081579 -5.784045 0.126082

H 1.325550 -5.520565 1.352028

H -0.379123 -5.584800 1.802339

H -2.409724 -3.982872 1.021782

H -3.017401 -0.098340 0.913752

H -4.325593 0.397506 -1.824391

H -5.154927 -0.500021 -0.563953

H -5.795202 1.925556 -0.774361

H -6.275155 0.651687 1.298659

H -4.693555 1.087393 1.962431

H -5.899653 2.336664 1.684929

H -2.874332 4.962433 -1.166072

H -1.659726 3.664305 -1.171787

H -2.472323 4.134498 -2.697586

H -0.204529 3.585931 2.999001

H -1.831165 2.925288 2.743218

H -0.580213 1.896381 3.458655

YYY-148d-c6 , delta G = 2.1160 kcal/mol, population = 0.80 %

C -4.191632 2.326121 -0.674770

C -4.227460 2.937137 0.573435

C -4.223488 2.152249 1.722077

C -4.181485 0.766281 1.620076

C -4.129936 0.140381 0.371696

C -4.139679 0.940071 -0.772363

C -4.074627 -1.358606 0.264955

C -2.709081 -2.016806 0.539589

C -1.521712 -1.590686 -0.383510

C -0.630157 -0.677332 0.471061

C 0.859800 -0.902173 0.181408

C 1.280638 -2.325717 0.445132

C 0.403519 -3.245765 -0.381947

C -0.806329 -2.865545 -0.819597

C -0.731618 0.869443 0.353250

C 0.579870 1.367379 0.964310

C 1.565014 0.228019 0.905440

N -2.179364 -1.739742 1.861944

C -1.022880 -1.044388 1.919271

O -0.427770 -0.753053 2.946152

C -1.638671 -3.744847 -1.729907

C 0.998729 -4.594205 -0.684342

H -1.897725 -1.065653 -1.260936

H 0.988664 -0.759551 -0.895675

O 2.647130 -2.536431 0.049505

C 1.810288 1.470898 0.101047

C 2.948901 2.375206 0.504629

C 4.217797 2.119284 -0.329931

C 4.499538 0.633889 -0.461519

C 5.440200 2.823781 0.270416

O 4.639083 -0.139284 0.463905

O 4.598172 0.264187 -1.747365

C 4.911366 -1.122765 -2.011645

H 2.206865 0.014483 1.747977

H 0.507690 2.006764 1.832166

O -0.857558 1.168714 -1.060792

O -0.978446 3.375653 -0.618334

C -0.981530 2.462496 -1.409254

C -1.121821 2.601203 -2.899450

H -4.197535 2.930840 -1.572923

H -4.253214 4.016285 0.650366

H -4.254605 2.619062 2.698580

H -4.187954 0.164161 2.519659

H -4.107408 0.471346 -1.748804

H -4.792578 -1.798782 0.963016

H -4.383039 -1.670415 -0.735422

H -2.869685 -3.091523 0.447839

H 1.185619 -2.568901 1.512043

H -1.596074 1.289345 0.859184

H -2.620200 -2.067165 2.708421

H -1.042443 -4.155965 -2.544712

H -2.089030 -4.589875 -1.201142

H -2.453657 -3.173275 -2.176503

H 0.256646 -5.299592 -1.051297

H 1.801110 -4.518249 -1.421860

H 1.449871 -5.013058 0.218383

H 3.199771 -1.869600 0.483322

H 1.656122 1.385817 -0.970367

H 2.676487 3.426797 0.380909

H 3.173434 2.225860 1.563847

H 4.049439 2.496651 -1.339614

H 5.239121 3.891640 0.370018

H 5.664068 2.422159 1.259798

H 6.321922 2.700928 -0.360711

H 5.045636 -1.184955 -3.087400

H 5.827249 -1.406251 -1.495280

H 4.091197 -1.760074 -1.686886

H -1.987576 2.033622 -3.243222

H -1.237409 3.649038 -3.162309

H -0.241351 2.189742 -3.395328

YYY-148d-c16-gfn0 , delta G = 2.3569 kcal/mol, population = 0.53 %

C 5.962445 0.371124 0.726315

C 5.653186 1.380375 1.634439

C 4.458712 2.079569 1.500478

C 3.576199 1.762600 0.472065

C 3.868154 0.745785 -0.436847

C 5.077893 0.059497 -0.299535

C 2.889200 0.371340 -1.520080

C 2.340309 -1.055136 -1.340907

C 1.440687 -1.254786 -0.086321

C 0.013936 -1.081283 -0.608137

C -1.011723 -1.887608 0.194914

C -0.724004 -3.368233 0.104771

C 0.731898 -3.619921 0.474709

C 1.663003 -2.652601 0.472915

C -0.617525 0.332386 -0.646170

C -2.115574 0.066909 -0.728370

C -2.366078 -1.347870 -0.248678

N 1.437881 -1.442067 -2.415393

C 0.131027 -1.534375 -2.081574

O -0.773930 -1.877201 -2.826468

C 3.068709 -2.900360 0.974410

C 1.044760 -5.039722 0.872876

H 1.689104 -0.512869 0.672851

H -0.854765 -1.618413 1.246661

O -1.615145 -4.119679 0.945427

C -2.887909 -0.190053 0.545658

C -4.365094 0.112669 0.583202

C -4.697126 1.576796 0.951115

C -4.224151 2.492058 -0.164211

C -4.208166 1.966585 2.345242

O -4.697580 2.483143 -1.279773

O -3.226422 3.314183 0.197847

C -2.754200 4.224641 -0.818414

H -3.050406 -1.995228 -0.779104

H -2.648086 0.468279 -1.578490

O -0.270835 1.022685 0.584279

O 0.135698 2.954295 -0.509835

C 0.010768 2.337185 0.523597

C 0.134650 2.927928 1.897740

H 6.894929 -0.172211 0.816293

H 6.341484 1.623427 2.434039

H 4.213324 2.874452 2.193921

H 2.657792 2.323230 0.361124

H 5.332278 -0.725722 -1.001942

H 2.064191 1.084426 -1.541667

H 3.376554 0.422390 -2.497980

H 3.182205 -1.745505 -1.318201

H -0.932874 -3.742033 -0.900402

H -0.268839 0.935653 -1.478467

H 1.749323 -1.642977 -3.353774

H 3.067832 -3.480698 1.897131

H 3.677931 -3.448629 0.249277

H 3.578854 -1.957101 1.172549

H 2.099183 -5.276302 0.742733

H 0.781984 -5.240232 1.915583

H 0.459630 -5.735036 0.268661

H -1.500551 -3.807912 1.852226

H -2.356024 0.014067 1.466757

H -4.813995 -0.117272 -0.385108

H -4.848366 -0.534433 1.320538

H -5.786894 1.662957 0.926228

H -4.523115 2.977426 2.604811

H -4.625438 1.279863 3.084277

H -3.122438 1.928738 2.419737

H -3.570482 4.859526 -1.159196

H -1.976318 4.811907 -0.342660

H -2.341205 3.669940 -1.657847

H -0.868393 3.189118 2.243575

H 0.560414 2.211832 2.598143

H 0.736154 3.832729 1.858324

YYY-148d-c17-gfn0 , delta G = 2.7805 kcal/mol, population = 0.26 %

C 3.051941 2.577009 0.857501

C 2.902778 3.276364 -0.334736

C 3.141539 2.630357 -1.543544

C 3.523676 1.293556 -1.556921

C 3.663820 0.575915 -0.366199

C 3.426102 1.238045 0.839493

C 4.068718 -0.872023 -0.384529

C 2.966577 -1.888153 -0.738385

C 1.705940 -1.909609 0.186427

C 0.576488 -1.258276 -0.624129

C -0.780946 -1.938129 -0.380591

C -0.733939 -3.403487 -0.741602

C 0.379519 -4.064154 0.049130

C 1.417413 -3.364772 0.530963

C 0.213378 0.232021 -0.394369

C -1.180902 0.360498 -1.000369

C -1.797721 -1.017936 -1.045466

N 2.377683 -1.685856 -2.049676

C 1.061736 -1.385209 -2.084773

O 0.404249 -1.224260 -3.102861

C 2.480269 -4.006720 1.398152

C 0.222244 -5.545705 0.260196

H 1.902872 -1.350783 1.100571

H -0.948142 -1.912455 0.699812

O -1.966887 -4.059945 -0.395563

C -2.391936 0.044110 -0.163388

C -3.705394 0.698089 -0.521608

C -3.757139 2.210108 -0.282612

C -3.489092 2.581116 1.162223

C -5.107647 2.796828 -0.726450

O -3.470380 1.818403 2.103927

O -3.294736 3.904636 1.289565

C -3.007083 4.390676 2.612570

H -2.325414 -1.344547 -1.932453

H -1.294711 1.047925 -1.825887

O 0.234254 0.437468 1.042210

O -0.313945 2.608125 0.783303

C -0.057077 1.667890 1.498412

C -0.056612 1.704532 2.999974

H 2.868568 3.072662 1.802539

H 2.597988 4.314686 -0.321989

H 3.030903 3.167587 -2.477154

H 3.714853 0.801981 -2.502327

H 3.537466 0.699521 1.773131

H 4.883317 -1.012857 -1.100870

H 4.462872 -1.157968 0.593165

H 3.444200 -2.868491 -0.720517

H -0.560621 -3.532906 -1.818538

H 0.915214 0.929998 -0.840975

H 2.896141 -1.803493 -2.907358

H 3.093104 -3.245106 1.882423

H 2.039353 -4.620858 2.183530

H 3.155438 -4.649329 0.825808

H 1.143470 -6.012790 0.600945

H -0.565628 -5.762287 0.985267

H -0.078944 -6.026297 -0.673707

H -2.666858 -3.652455 -0.918275

H -2.227176 -0.080761 0.898611

H -3.913741 0.532182 -1.582292

H -4.512016 0.214262 0.035951

H -2.966057 2.688413 -0.865314

H -5.127722 3.879527 -0.609258

H -5.284822 2.560773 -1.776814

H -5.925442 2.369566 -0.141760

H -2.061632 3.980702 2.963036

H -2.937506 5.470204 2.517383

H -3.803536 4.118197 3.303758

H 0.213133 2.702256 3.339457

H -1.070597 1.484032 3.341292

H 0.620978 0.962828 3.417530

### Table S19. Geometry data of conformers of structure 2e.

YYY-148e-c2 , delta G = 0.0000 kcal/mol, population = 50.19 %

C 6.656281 0.444161 1.515757

C 6.521178 -0.408283 2.608327

C 5.400002 -1.225847 2.707286

C 4.419491 -1.188716 1.720563

C 4.543292 -0.337247 0.621542

C 5.673894 0.478393 0.532274

C 3.448839 -0.252431 -0.408609

C 2.496226 0.924328 -0.139592

C 1.265456 0.972705 -1.080879

C 0.174661 0.193439 -0.325448

C -1.196624 0.609434 -0.881867

C -1.506811 2.031552 -0.466735

C -0.368100 2.915640 -0.950576

C 0.833457 2.422536 -1.294814

C 0.150266 -1.360668 -0.428927

C -1.312448 -1.758507 -0.365450

C -2.154391 -0.528527 -0.631481

N 1.847356 0.853158 1.159692

C 0.529223 0.561947 1.143962

O -0.190113 0.557691 2.133399

C 1.906662 3.298053 -1.903744

C -0.704748 4.379666 -1.042914

H 1.503127 0.509622 -2.038451

H -1.069526 0.644674 -1.969921

O -2.733131 2.492002 -1.051408

C -2.198074 -1.189654 0.713894

C -3.450607 -1.935220 1.105119

C -4.526671 -0.998610 1.695500

C -4.884415 0.076336 0.691446

C -5.758158 -1.778674 2.165175

O -4.575954 1.248490 0.789818

O -5.570773 -0.407117 -0.352304

C -5.926662 0.530967 -1.389746

H -3.019044 -0.606185 -1.276252

H -1.567568 -2.723953 -0.781735

O 0.665357 -1.721248 -1.740790

O 1.263769 -3.763166 -0.993591

C 1.196246 -2.951773 -1.888028

C 1.694471 -3.155322 -3.290228

H 7.528817 1.079766 1.428201

H 7.286330 -0.437487 3.373821

H 5.288713 -1.894940 3.551511

H 3.548839 -1.828419 1.801676

H 5.787201 1.142615 -0.317257

H 3.873982 -0.111183 -1.405263

H 2.885628 -1.184268 -0.423817

H 3.075944 1.844722 -0.196616

H -1.593755 2.101665 0.622908

H 0.769683 -1.850670 0.319206

H 2.316372 1.068483 2.026115

H 2.636155 2.690583 -2.443175

H 1.488631 4.013122 -2.611345

H 2.457353 3.869543 -1.150340

H 0.182705 5.009422 -1.047311

H -1.288291 4.598211 -1.940862

H -1.326885 4.671008 -0.194333

H -3.450104 2.193668 -0.469425

H -1.717445 -0.662472 1.525261

H -3.216679 -2.689895 1.860820

H -3.870008 -2.463363 0.244359

H -4.089785 -0.463796 2.540101

H -5.468001 -2.484331 2.945029

H -6.203074 -2.338975 1.342507

H -6.518039 -1.112663 2.577451

H -5.029588 0.933177 -1.858929

H -6.507646 -0.039248 -2.107783

H -6.518711 1.345632 -0.976341

H 2.468776 -2.419835 -3.514177

H 2.097212 -4.158768 -3.395466

H 0.880688 -3.002528 -4.000122

YYY-148e-c1 , delta G = 0.4788 kcal/mol, population = 22.35 %

C 6.468836 0.969311 -0.041210

C 6.541328 0.272552 -1.244603

C 5.575344 -0.682203 -1.542983

C 4.541648 -0.931507 -0.645635

C 4.454001 -0.237425 0.560130

C 5.434423 0.714665 0.851707

C 3.298758 -0.456719 1.502064

C 2.297008 0.708577 1.438352

C 1.454311 0.778494 0.137303

C 0.142367 0.045377 0.476726

C -0.953778 0.535096 -0.484640

C -1.311852 1.971762 -0.167856

C -0.037958 2.796382 -0.261193

C 1.184014 2.240892 -0.210052

C 0.079449 -1.503165 0.324889

C -1.348958 -1.823535 -0.076659

C -1.997258 -0.550577 -0.578115

N 1.255333 0.616494 2.446583

C 0.006762 0.381927 1.989960

O -0.996322 0.402812 2.689752

C 2.438989 3.050444 -0.456458

C -0.253095 4.272891 -0.456060

H 1.984281 0.297954 -0.683399

H -0.479240 0.564741 -1.471841

O -2.269701 2.489415 -1.101495

C -2.507476 -1.211079 0.668242

C -3.851984 -1.894629 0.615678

C -5.018020 -0.909673 0.846875

C -4.976509 0.192428 -0.190145

C -6.369207 -1.630529 0.865723

O -4.663097 1.345887 0.034481

O -5.303132 -0.244962 -1.413206

C -5.250724 0.717824 -2.487203

H -2.608686 -0.581016 -1.469550

H -1.507675 -2.773176 -0.569510

O 0.943407 -1.866307 -0.792386

O 1.030401 -4.001842 -0.074345

C 1.306320 -3.159651 -0.897546

C 2.069645 -3.422071 -2.166037

H 7.220433 1.709921 0.202555

H 7.346829 0.470109 -1.940547

H 5.624961 -1.233644 -2.473793

H 3.792307 -1.672972 -0.883445

H 5.388700 1.260558 1.787299

H 2.791721 -1.394734 1.273989

H 3.664116 -0.531646 2.529536

H 2.852656 1.633294 1.587194

H -1.735547 2.046591 0.839432

H 0.425854 -2.039674 1.205727

H 1.406420 0.839257 3.418901

H 3.280012 2.395180 -0.688258

H 2.312732 3.736408 -1.293780

H 2.728811 3.649371 0.412293

H -1.080250 4.613175 0.170479

H 0.630218 4.855779 -0.202570

H -0.534706 4.501620 -1.486901

H -3.145642 2.221347 -0.781143

H -2.293136 -0.710210 1.600772

H -3.911110 -2.665395 1.388776

H -3.992246 -2.395130 -0.346441

H -4.858638 -0.407678 1.802313

H -6.383292 -2.359159 1.677731

H -6.543010 -2.157917 -0.072533

H -7.192053 -0.931423 1.024179

H -5.595981 0.186289 -3.368403

H -5.900285 1.563669 -2.269088

H -4.229256 1.068543 -2.629890

H 2.913597 -4.076526 -1.953312

H 1.407270 -3.946093 -2.858435

H 2.407560 -2.501967 -2.636166

YYY-148e-c4 , delta G = 0.5083 kcal/mol, population = 21.26 %

C 6.442827 0.784560 1.678609

C 6.315549 0.019861 2.835305

C 5.248376 -0.863545 2.961057

C 4.313183 -0.980156 1.936670

C 4.429790 -0.217477 0.773672

C 5.505728 0.666167 0.658603

C 3.383823 -0.292590 -0.306793

C 2.370197 0.858003 -0.197543

C 1.221150 0.799998 -1.234787

C 0.104232 0.016106 -0.524846

C -1.224119 0.339719 -1.222715

C -1.634698 1.762061 -0.927352

C -0.497753 2.674184 -1.354353

C 0.749211 2.218156 -1.563038

C 0.142858 -1.544202 -0.534252

C -1.306591 -1.997694 -0.585606

C -2.157239 -0.812844 -0.978227

N 1.617513 0.842103 1.046719

C 0.318016 0.490120 0.942090

O -0.482140 0.516411 1.867286

C 1.836530 3.109117 -2.123214

C -0.888890 4.113604 -1.552090

H 1.553427 0.299317 -2.143974

H -1.003504 0.316733 -2.296052

O -2.815455 2.128593 -1.656456

C -2.289248 -1.402818 0.393173

C -3.579238 -2.125255 0.703561

C -4.559702 -1.240244 1.494487

C -4.684566 0.147587 0.896562

C -5.954178 -1.877128 1.579638

O -4.865667 0.396350 -0.279484

O -4.603019 1.099735 1.835830

C -4.777073 2.465405 1.400459

H -2.989447 -0.940502 -1.655657

H -1.489679 -2.992131 -0.970835

O 0.800054 -1.957553 -1.763230

O 1.419018 -3.914648 -0.829142

C 1.405025 -3.162267 -1.776116

C 2.066581 -3.415069 -3.100604

H 7.274154 1.470222 1.570367

H 7.045136 0.109424 3.630265

H 5.143966 -1.464788 3.855714

H 3.484858 -1.671377 2.037891

H 5.611297 1.262767 -0.240589

H 3.851686 -0.229127 -1.292303

H 2.864682 -1.248286 -0.252141

H 2.920469 1.795129 -0.268236

H -1.828149 1.887365 0.145341

H 0.700783 -1.962365 0.300854

H 2.003800 1.129212 1.932925

H 2.638664 2.508056 -2.555753

H 1.456009 3.761334 -2.908297

H 2.289031 3.748358 -1.359043

H -0.029198 4.779576 -1.574782

H -1.457291 4.246391 -2.475726

H -1.544785 4.433177 -0.738817

H -3.547386 1.592968 -1.310620

H -1.877522 -0.837107 1.217018

H -3.392631 -3.019474 1.304799

H -4.051987 -2.459215 -0.224218

H -4.170220 -1.108695 2.504934

H -6.393762 -1.970023 0.585202

H -6.626491 -1.285028 2.202444

H -5.876909 -2.874518 2.015221

H -4.744952 3.062229 2.306463

H -3.972974 2.756293 0.726838

H -5.734817 2.581801 0.895728

H 2.895610 -2.716060 -3.227934

H 2.442050 -4.433924 -3.135789

H 1.362322 -3.242799 -3.914494

YYY-148e-c3 , delta G = 1.5506 kcal/mol, population = 3.65 %

C 6.447858 1.130354 0.334996

C 6.640952 0.479625 -0.880761

C 5.720228 -0.473712 -1.301997

C 4.611974 -0.769819 -0.514181

C 4.405550 -0.123310 0.704258

C 5.339668 0.829683 1.118079

C 3.170669 -0.378536 1.528917

C 2.132543 0.737737 1.320264

C 1.396828 0.692439 -0.044229

C 0.090722 -0.072223 0.239764

C -0.927944 0.298199 -0.847017

C -1.372199 1.732577 -0.684164

C -0.133301 2.610504 -0.719811

C 1.099323 2.117450 -0.511683

C 0.089018 -1.632359 0.206906

C -1.285769 -2.036977 -0.302368

C -1.925027 -0.823553 -0.934949

N 1.016111 0.665425 2.247941

C -0.185201 0.372449 1.705244

O -1.246130 0.425746 2.311854

C 2.339992 2.965189 -0.695896

C -0.393675 4.057032 -1.040922

H 2.008705 0.185774 -0.788410

H -0.370916 0.270525 -1.790355

O -2.248495 2.131681 -1.748745

C -2.514669 -1.412224 0.311001

C -3.855837 -2.094682 0.177848

C -5.010628 -1.180674 0.624500

C -4.903050 0.207752 0.024740

C -6.378204 -1.778461 0.264138

O -4.688328 0.457473 -1.145350

O -5.108189 1.159992 0.944872

C -5.103781 2.528251 0.483588

H -2.493138 -0.921123 -1.849041

H -1.368932 -3.023639 -0.737902

O 1.073684 -2.051896 -0.781760

O 1.155874 -4.111751 0.132045

C 1.495612 -3.330997 -0.726980

C 2.426799 -3.662652 -1.860043

H 7.162320 1.870504 0.673422

H 7.503922 0.711773 -1.491967

H 5.863157 -0.988071 -2.244277

H 3.896576 -1.507290 -0.850823

H 5.197339 1.339963 2.064082

H 2.733155 -1.345444 1.280467

H 3.431031 -0.405816 2.589757

H 2.641543 1.691077 1.455501

H -1.895338 1.863829 0.271407

H 0.358371 -2.082171 1.160246

H 1.078221 0.969465 3.207979

H 3.222622 2.333443 -0.807534

H 2.267166 3.592026 -1.584163

H 2.525436 3.627640 0.154994

H -0.637879 4.190629 -2.097529

H -1.260150 4.412247 -0.477757

H 0.453252 4.694947 -0.797919

H -3.063531 1.611240 -1.664866

H -2.378191 -0.861354 1.230282

H -3.898983 -2.996872 0.794482

H -4.015108 -2.409325 -0.857259

H -4.955148 -1.055500 1.706800

H -7.195685 -1.163290 0.643284

H -6.470727 -2.773980 0.701080

H -6.483869 -1.867781 -0.818366

H -5.341646 3.126797 1.357251

H -4.122978 2.797039 0.095475

H -5.853263 2.666760 -0.294127

H 1.954999 -4.426266 -2.480474

H 2.652071 -2.792913 -2.471685

H 3.345291 -4.088054 -1.455621

YYY-148e-c6 , delta G = 2.0990 kcal/mol, population = 1.45 %

C 6.345175 0.744874 1.690331

C 6.187853 -0.037495 2.831384

C 5.108181 -0.909835 2.922945

C 4.190928 -0.997223 1.879841

C 4.337740 -0.217242 0.731828

C 5.426002 0.655065 0.651098

C 3.312880 -0.270180 -0.369448

C 2.295859 0.878274 -0.264049

C 1.150658 0.814108 -1.305259

C 0.027028 0.038375 -0.594957

C -1.300222 0.373651 -1.294270

C -1.687780 1.798405 -0.964272

C -0.554201 2.698245 -1.431296

C 0.686921 2.231021 -1.650777

C 0.055838 -1.519796 -0.596885

C -1.395765 -1.961202 -0.644109

C -2.241134 -0.782623 -1.074073

N 1.540416 0.860637 0.979016

C 0.242702 0.508901 0.872911

O -0.556797 0.529238 1.800809

C 1.775819 3.103949 -2.235910

C -0.937140 4.137138 -1.645093

H 1.486790 0.302736 -2.207031

H -1.076799 0.367551 -2.366888

O -2.907512 2.176211 -1.613249

C -2.394995 -1.342551 0.304306

C -3.659870 -2.083047 0.672272

C -4.908431 -1.155313 0.801099

C -4.480168 0.194293 1.340358

C -5.685087 -0.998528 -0.504566

O -4.407392 1.220535 0.693947

O -4.146678 0.125878 2.634027

C -3.576669 1.317735 3.221770

H -3.035686 -0.923608 -1.791694

H -1.584767 -2.962085 -1.008768

O 0.703839 -1.945088 -1.827275

O 1.320882 -3.898986 -0.884724

C 1.300221 -3.154164 -1.837495

C 1.936355 -3.425160 -3.170955

H 7.186611 1.421843 1.608770

H 6.904213 0.029814 3.640461

H 4.980559 -1.525374 3.804751

H 3.353533 -1.680705 1.953845

H 5.556890 1.264493 -0.236053

H 3.799664 -0.191002 -1.344495

H 2.791618 -1.226248 -0.341477

H 2.842327 1.817842 -0.333349

H -1.828213 1.911688 0.114949

H 0.613129 -1.940069 0.237480

H 1.926374 1.144164 1.866646

H 2.241811 3.751700 -1.487117

H 2.568730 2.490283 -2.668199

H 1.392769 3.747269 -3.027185

H -0.072052 4.795289 -1.690665

H -1.517216 4.261516 -2.562694

H -1.580420 4.473386 -0.828782

H -3.619429 1.883426 -1.023362

H -1.986291 -0.743783 1.104828

H -3.501579 -2.599248 1.620747

H -3.891160 -2.848203 -0.072775

H -5.566371 -1.598480 1.551873

H -5.984898 -1.979489 -0.875520

H -5.087763 -0.507084 -1.269936

H -6.585373 -0.401165 -0.353450

H -4.254996 2.160507 3.102049

H -3.441035 1.081277 4.272759

H -2.618075 1.530864 2.752596

H 2.371167 -4.420657 -3.175705

H 1.193298 -3.336381 -3.964162

H 2.712494 -2.682327 -3.362820

YYY-148e-c5 , delta G = 2.2659 kcal/mol, population = 1.09 %

C 5.678092 -0.524394 -1.263538

C 6.604440 0.416862 -0.827275

C 6.394899 1.077954 0.380132

C 5.265236 0.799467 1.140568

C 4.326171 -0.141868 0.711751

C 4.548549 -0.798376 -0.498378

C 3.071667 -0.378517 1.511250

C 2.049824 0.747387 1.275816

C 1.346821 0.705013 -0.105343

C 0.026511 -0.047796 0.147014

C -0.964390 0.342721 -0.961829

C -1.399986 1.778360 -0.760261

C -0.148352 2.640801 -0.803196

C 1.076107 2.131921 -0.583716

C 0.009101 -1.605524 0.113336

C -1.354989 -1.991724 -0.431524

C -1.959904 -0.779462 -1.104477

N 0.910956 0.685263 2.176614

C -0.277004 0.390601 1.609383

O -1.349928 0.433510 2.198204

C 2.331433 2.959929 -0.759950

C -0.383840 4.087829 -1.138210

H 1.971221 0.190002 -0.833257

H -0.382683 0.337282 -1.890134

O -2.316386 2.197327 -1.778564

C -2.600778 -1.344276 0.123181

C -3.935822 -2.043029 0.011599

C -5.123350 -1.070583 -0.270050

C -4.871098 0.249939 0.428845

C -5.390740 -0.854379 -1.757945

O -4.544153 1.285074 -0.116943

O -5.017617 0.142858 1.754496

C -4.659527 1.300824 2.542707

H -2.460250 -0.885858 -2.055698

H -1.441682 -2.981888 -0.857609

O 1.015413 -2.037774 -0.848577

O 1.017912 -4.107820 0.045331

C 1.408071 -3.325992 -0.791061

C 2.381682 -3.665521 -1.885589

H 5.833367 -1.046247 -2.199744

H 7.484142 0.631871 -1.420627

H 7.113283 1.808970 0.729913

H 5.109738 1.317743 2.080123

H 3.828965 -1.527003 -0.845182

H 2.627786 -1.340897 1.256232

H 3.308623 -0.405681 2.577570

H 2.562779 1.697172 1.420714

H -1.897166 1.889969 0.208059

H 0.247499 -2.059930 1.072897

H 0.954972 0.975849 3.141877

H 2.282287 3.572353 -1.660143

H 2.510354 3.635490 0.082165

H 3.208168 2.315807 -0.844885

H -1.261143 4.455514 -0.601286

H 0.465306 4.716462 -0.878322

H -0.599459 4.218175 -2.201626

H -3.194333 1.926695 -1.468083

H -2.480116 -0.767323 1.027865

H -4.130002 -2.581298 0.940958

H -3.917868 -2.785537 -0.789837

H -6.014146 -1.503901 0.189905

H -6.273444 -0.231351 -1.909243

H -5.563709 -1.814463 -2.245883

H -4.552249 -0.362928 -2.247169

H -4.903238 1.035876 3.567068

H -3.593201 1.493471 2.442129

H -5.234231 2.168300 2.223323

H 2.594309 -2.811044 -2.522761

H 3.304216 -4.037502 -1.438650

H 1.959568 -4.472045 -2.486381

### Table S20. Geometry data of conformers of structure 2f.

YYY-148f-c4 , delta G = 0.0000 kcal/mol, population = 64.59 %

C 6.607765 0.124587 -0.858011

C 6.588495 1.449201 -1.286919

C 5.499754 2.256717 -0.974520

C 4.436821 1.741302 -0.238884

C 4.444396 0.415175 0.195558

C 5.543144 -0.386386 -0.123977

C 3.264736 -0.152493 0.938902

C 2.336217 -0.950685 0.010489

C 1.042201 -1.487609 0.676654

C -0.020258 -0.401857 0.401632

C -1.400093 -1.055919 0.543924

C -1.657340 -1.983502 -0.625303

C -0.528337 -3.003380 -0.639148

C 0.633164 -2.802940 0.008077

C -0.099799 0.837138 1.353216

C -1.572276 1.089941 1.657120

C -2.394724 -0.046149 1.075939

N 1.778953 -0.146989 -1.065028

C 0.446677 0.068227 -1.002607

O -0.212648 0.567075 -1.901926

C 1.684404 -3.888158 0.099143

C -0.829455 -4.262182 -1.405795

H 1.199446 -1.643687 1.743192

H -1.293646 -1.735234 1.397656

O -2.907885 -2.664164 -0.493571

C -2.620657 1.367759 0.606121

C -2.500047 1.983413 -0.772428

C -3.208113 1.161614 -1.867772

C -4.533503 0.621775 -1.385371

C -3.352672 1.965207 -3.162994

O -4.784740 -0.556245 -1.208046

O -5.430798 1.587929 -1.149489

C -6.719026 1.171927 -0.652268

H -3.198237 -0.479756 1.656380

H -1.755089 1.480683 2.649207

O 0.536787 0.479154 2.610242

O 0.990196 2.643306 3.055123

C 1.037207 1.476529 3.368631

C 1.663195 0.931815 4.620423

H 7.454329 -0.509077 -1.092032

H 7.417997 1.848868 -1.856552

H 5.478294 3.289252 -1.300412

H 3.592034 2.374417 0.004832

H 5.566699 -1.417995 0.209279

H 3.600751 -0.824330 1.732583

H 2.705445 0.654935 1.406534

H 2.918348 -1.763362 -0.421979

H -1.654594 -1.434337 -1.572004

H 0.419892 1.705816 0.953353

H 2.299409 0.105539 -1.891097

H 2.369261 -3.692045 0.926422

H 1.236206 -4.866259 0.270524

H 2.289437 -3.962624 -0.809599

H 0.070914 -4.813825 -1.668265

H -1.488873 -4.923385 -0.838019

H -1.361191 -4.016239 -2.327342

H -3.596579 -2.025662 -0.747636

H -3.511649 1.745182 1.099883

H -1.470085 2.104086 -1.088562

H -2.950222 2.978141 -0.727251

H -2.593516 0.288848 -2.065154

H -3.815611 1.370757 -3.952863

H -2.362979 2.270483 -3.505495

H -3.956378 2.859708 -3.005022

H -7.205549 0.510403 -1.367254

H -6.606768 0.657300 0.300941

H -7.290210 2.086797 -0.527759

H 0.932416 0.344182 5.177230

H 2.488289 0.267681 4.357893

H 2.030062 1.749644 5.234139

YYY-148f-c2 , delta G = 0.4612 kcal/mol, population = 29.63 %

C 6.527574 0.389244 -0.676879

C 6.481416 1.720949 -1.080717

C 5.360066 2.489747 -0.785843

C 4.291880 1.929034 -0.092250

C 4.326220 0.594884 0.316699

C 5.457280 -0.167374 0.014471

C 3.145489 -0.022507 1.017525

C 2.238511 -0.785095 0.039413

C 0.965596 -1.416310 0.662476

C -0.134951 -0.349559 0.476601

C -1.490654 -1.063048 0.584525

C -1.728279 -1.892751 -0.658387

C -0.557527 -2.857134 -0.782984

C 0.598481 -2.680443 -0.119653

C -0.243439 0.807445 1.522766

C -1.715681 0.968034 1.881596

C -2.514225 -0.144118 1.222087

N 1.648013 0.073195 -0.974892

C 0.308068 0.226601 -0.898740

O -0.371504 0.741660 -1.775017

C 1.683521 -3.735353 -0.127583

C -0.816141 -4.046890 -1.667288

H 1.129820 -1.656024 1.712077

H -1.342323 -1.808342 1.374457

O -2.937830 -2.651429 -0.570952

C -2.806612 1.292600 0.888212

C -2.707246 2.048202 -0.410504

C -3.971321 1.941789 -1.305087

C -4.117618 0.540914 -1.856959

C -5.260351 2.369983 -0.598629

O -4.582733 -0.405195 -1.249384

O -3.673185 0.444937 -3.116739

C -3.770437 -0.848741 -3.748663

H -3.282566 -0.661022 1.781672

H -1.887371 1.264860 2.907653

O 0.444716 0.385431 2.733057

O 0.778493 2.530041 3.346244

C 0.896514 1.346560 3.564611

C 1.559074 0.742515 4.769545

H 7.399557 -0.214328 -0.896778

H 7.315115 2.156363 -1.617062

H 5.317458 3.527627 -1.092135

H 3.422611 2.533074 0.139036

H 5.502329 -1.203907 0.329613

H 3.483281 -0.728153 1.780369

H 2.571390 0.753738 1.518274

H 2.847039 -1.543396 -0.451521

H -1.771410 -1.252156 -1.545191

H 0.226217 1.727129 1.179125

H 2.149750 0.385028 -1.792297

H 2.288851 -3.709552 -1.038777

H 2.363411 -3.592154 0.714477

H 1.265747 -4.738147 -0.044831

H -1.436839 -4.790598 -1.161412

H -1.371805 -3.736242 -2.554612

H 0.103386 -4.528234 -1.993863

H -3.662310 -2.027569 -0.741338

H -3.684978 1.604581 1.444667

H -1.846116 1.741874 -0.991738

H -2.584408 3.110468 -0.176211

H -3.798973 2.597106 -2.159636

H -6.092325 2.419991 -1.303279

H -5.129973 3.359497 -0.156879

H -5.533323 1.670198 0.190065

H -3.405122 -0.702775 -4.760281

H -3.155035 -1.578450 -3.226041

H -4.805479 -1.186930 -3.757647

H 1.970867 1.529134 5.395483

H 0.828864 0.163019 5.336756

H 2.350093 0.059572 4.457722

YYY-148f-c6 , delta G = 1.5788 kcal/mol, population = 4.48 %

C 6.558833 -0.968177 -0.059747

C 6.513534 -1.212369 1.309941

C 5.442062 -0.733334 2.056417

C 4.422678 -0.017837 1.436051

C 4.455969 0.233833 0.063791

C 5.537928 -0.251892 -0.674286

C 3.320436 0.937574 -0.633862

C 2.296400 -0.075788 -1.170833

C 1.374870 -0.722114 -0.099796

C 0.070618 0.105001 -0.139826

C -1.047275 -0.771816 0.441075

C -1.394567 -1.884052 -0.527553

C -0.117532 -2.675308 -0.766881

C 1.098961 -2.175486 -0.489796

C -0.032696 1.433556 0.681578

C -1.386584 1.431963 1.385615

C -2.061877 0.092935 1.156274

N 1.316783 0.515397 -2.068196

C 0.032563 0.463537 -1.647468

O -0.933358 0.697631 -2.357192

C 2.347085 -3.027748 -0.578805

C -0.323968 -4.069933 -1.292634

H 1.845718 -0.689325 0.880197

H -0.573726 -1.296057 1.279184

O -2.393944 -2.754650 0.008746

C -2.711929 1.360901 0.665040

C -3.098254 1.796129 -0.732910

C -3.873847 0.716738 -1.514292

C -4.869908 0.000680 -0.633536

C -4.535365 1.300678 -2.765997

O -4.805793 -1.173707 -0.318739

O -5.854475 0.802061 -0.206583

C -6.837443 0.213248 0.669334

H -2.565705 -0.402537 1.975589

H -1.381709 1.912145 2.354528

O 0.990767 1.429960 1.717125

O 0.820161 3.671352 1.906114

C 1.306303 2.624470 2.265020

C 2.345279 2.486004 3.341693

H 7.390884 -1.333597 -0.648837

H 7.307848 -1.769157 1.790858

H 5.398066 -0.917076 3.122829

H 3.589070 0.342929 2.022410

H 5.580875 -0.064320 -1.741289

H 2.826198 1.640800 0.034137

H 3.706745 1.508925 -1.480975

H 2.849437 -0.842052 -1.713048

H -1.754531 -1.480290 -1.479093

H 0.132218 2.313649 0.062729

H 1.518104 0.715062 -3.036613

H 3.164436 -2.572268 -0.017579

H 2.178677 -4.023525 -0.169290

H 2.696236 -3.155323 -1.607945

H -1.128957 -4.074025 -2.030128

H 0.572539 -4.470385 -1.762208

H -0.631580 -4.753828 -0.497365

H -3.245495 -2.296004 -0.096240

H -3.499882 1.626018 1.364205

H -2.240488 2.064775 -1.338991

H -3.725827 2.685608 -0.637298

H -3.158168 -0.040774 -1.819509

H -5.257186 2.075055 -2.503464

H -5.049633 0.529811 -3.342996

H -3.767606 1.743556 -3.401941

H -7.532407 1.013697 0.903908

H -7.350432 -0.605122 0.166786

H -6.360577 -0.158594 1.575029

H 2.306547 1.505460 3.811121

H 3.333710 2.619482 2.896470

H 2.199385 3.268702 4.082811

YYY-148f-c7 , delta G = 2.3105 kcal/mol, population = 1.30 %

C 6.758719 -0.035140 -0.469748

C 6.835074 1.213854 -1.080574

C 5.753341 2.084864 -1.000781

C 4.601617 1.708067 -0.315969

C 4.512763 0.457634 0.297627

C 5.606010 -0.407875 0.212038

C 3.244978 0.023502 0.984206

C 2.372155 -0.844800 0.064949

C 1.045566 -1.352084 0.686196

C -0.008626 -0.296736 0.288010

C -1.392407 -0.956409 0.436538

C -1.598401 -1.941154 -0.696315

C -0.447462 -2.936753 -0.632439

C 0.680433 -2.700617 0.060332

C -0.125283 1.005496 1.138259

C -1.604761 1.267432 1.372188

C -2.413431 0.066391 0.907334

N 1.870978 -0.122578 -1.092199

C 0.533861 0.074125 -1.125489

O -0.065842 0.495419 -2.102884

C 1.733255 -3.771981 0.242564

C -0.694133 -4.227931 -1.365175

H 1.140245 -1.448292 1.767065

H -1.293432 -1.591314 1.324259

O -2.836782 -2.649888 -0.561942

C -2.661670 1.423012 0.304601

C -2.558087 1.818210 -1.146285

C -3.936313 1.741016 -1.863112

C -4.547211 0.386466 -1.575803

C -4.847362 2.921766 -1.528016

O -4.158662 -0.652789 -2.076670

O -5.536989 0.428121 -0.677085

C -6.110904 -0.834620 -0.279847

H -3.191146 -0.337626 1.542205

H -1.814384 1.757247 2.313745

O 0.464524 0.744640 2.443694

O 0.894843 2.938171 2.744015

C 0.932957 1.797557 3.144018

C 1.515723 1.350913 4.454333

H 7.598934 -0.716554 -0.521556

H 7.732633 1.506966 -1.610455

H 5.806003 3.059990 -1.468974

H 3.762956 2.391287 -0.253439

H 5.554122 -1.380231 0.688932

H 3.479127 -0.563251 1.875707

H 2.682357 0.898132 1.302532

H 2.983489 -1.679160 -0.276386

H -1.596462 -1.430036 -1.662509

H 0.406000 1.846360 0.697127

H 2.431578 0.067634 -1.908712

H 2.370765 -3.542762 1.098756

H 1.283537 -4.748142 0.420941

H 2.387249 -3.870067 -0.629362

H -1.326213 -4.902420 -0.781845

H -1.231279 -4.029875 -2.294697

H 0.230247 -4.748048 -1.609431

H -3.485310 -2.144165 -1.076229

H -3.534801 1.872095 0.769442

H -1.846210 1.195024 -1.671439

H -2.206452 2.850425 -1.231941

H -3.734274 1.741124 -2.936240

H -5.809511 2.841138 -2.035456

H -4.368416 3.847304 -1.851373

H -5.042285 2.995082 -0.458368

H -6.928776 -0.582823 0.388028

H -6.477938 -1.376240 -1.149837

H -5.367298 -1.436511 0.241178

H 2.376598 0.706225 4.268836

H 1.825086 2.216457 5.033369

H 0.781958 0.768006 5.011770

### Table S21. Geometry data of conformers of structure 2g.

YYY-148g-c2redo , delta G = 0.0000 kcal/mol, population = 83.31 %

C -4.550784 1.565872 -1.947958

C -5.536381 0.699760 -2.409423

C -5.861971 -0.429696 -1.663171

C -5.200928 -0.691034 -0.468607

C -4.204256 0.167353 0.001766

C -3.891913 1.298723 -0.752024

C -3.443187 -0.163913 1.258240

C -2.436157 -1.304658 1.022978

C -1.244204 -0.926438 0.098771

C -0.131171 -0.515915 1.064204

C 1.264071 -0.766883 0.486262

C 1.478147 -2.246633 0.241000

C 0.333464 -2.768883 -0.622218

C -0.842705 -2.132924 -0.736088

C -0.048370 0.949020 1.563281

C 1.397580 1.109211 2.019103

C 2.213973 -0.011564 1.404794

N -1.748609 -1.705978 2.241931

C -0.444493 -1.358461 2.322502

O 0.311476 -1.627673 3.243497

C -1.920494 -2.602943 -1.689598

C 0.636205 -4.047302 -1.358170

H -1.528934 -0.108475 -0.561366

H 1.272069 -0.306271 -0.504915

O 2.750382 -2.505478 -0.359965

C 2.530007 1.426624 1.068376

C 2.412644 2.062309 -0.298355

C 3.734779 2.012873 -1.086689

C 4.158865 0.572473 -1.267032

C 3.589868 2.692120 -2.452028

O 3.532443 -0.245616 -1.915388

O 5.298073 0.290512 -0.630960

C 5.783986 -1.070371 -0.716482

H 2.910224 -0.579519 2.004507

H 1.533480 1.393865 3.052186

O -0.436859 1.817911 0.466121

O -0.535804 3.576422 1.869475

C -0.629760 3.118261 0.754375

C -0.963664 3.916392 -0.475856

H -4.293615 2.450140 -2.517983

H -6.050262 0.904567 -3.340110

H -6.632782 -1.105943 -2.011481

H -5.462833 -1.572127 0.106146

H -3.123147 1.973152 -0.399507

H -2.918445 0.718070 1.627361

H -4.134514 -0.478208 2.044401

H -2.979993 -2.158436 0.620415

H 1.508634 -2.791391 1.188274

H -0.725928 1.150974 2.390036

H -2.195894 -2.219717 2.986174

H -2.459357 -3.476619 -1.310621

H -2.659451 -1.817454 -1.853635

H -1.504854 -2.876402 -2.659485

H -0.268673 -4.563673 -1.672459

H 1.251408 -3.866738 -2.243794

H 1.210714 -4.720187 -0.718330

H 2.897213 -1.859547 -1.070680

H 3.393299 1.803134 1.609565

H 1.629208 1.603282 -0.897915

H 2.141668 3.115155 -0.185269

H 4.511882 2.514913 -0.508665

H 3.278150 3.728882 -2.317110

H 2.839167 2.180355 -3.055483

H 4.532819 2.689058 -3.001084

H 5.914567 -1.356952 -1.758786

H 6.738118 -1.065394 -0.198987

H 5.083045 -1.746044 -0.230654

H -1.847986 4.522674 -0.281596

H -0.135745 4.596719 -0.682673

H -1.125538 3.278631 -1.341001

YYY-148g-c3 , delta G = 1.0530 kcal/mol, population = 14.06 %

C -6.154315 0.138299 1.820276

C -7.144774 -0.418393 1.017516

C -6.878515 -0.675105 -0.324751

C -5.628396 -0.378803 -0.856683

C -4.625924 0.180264 -0.060431

C -4.905160 0.434945 1.283458

C -3.253648 0.437655 -0.622580

C -2.378601 -0.828168 -0.616835

C -0.907095 -0.564373 -1.040689

C -0.171204 -0.344465 0.282008

C 1.310643 -0.717102 0.192649

C 1.467431 -2.195226 -0.096902

C 0.646602 -2.541104 -1.336097

C -0.357013 -1.771448 -1.785570

C -0.146322 1.067265 0.921671

C 1.061489 1.038719 1.850691

C 1.944693 -0.125209 1.443227

N -2.201682 -1.397621 0.711879

C -0.983242 -1.216627 1.267816

O -0.627399 -1.625727 2.362608

C -1.084055 -2.078441 -3.077248

C 1.066887 -3.810224 -2.028557

H -0.858299 0.316640 -1.681532

H 1.701181 -0.206597 -0.691741

O 2.841101 -2.562125 -0.257130

C 2.472419 1.288401 1.369902

C 2.879949 2.015555 0.108528

C 4.383228 1.869984 -0.194330

C 4.723992 0.403928 -0.340997

C 4.767992 2.631444 -1.466382

O 4.299551 -0.308047 -1.232824

O 5.537792 -0.031825 0.623143

C 5.901656 -1.432587 0.596639

H 2.344912 -0.799268 2.186847

H 0.855899 1.241721 2.891650

O -0.069908 2.043767 -0.148525

O -0.569763 3.695831 1.298040

C -0.298847 3.330487 0.177754

C -0.177814 4.218735 -1.027671

H -6.353949 0.344425 2.864497

H -8.118049 -0.647291 1.432975

H -7.645834 -1.103494 -0.957695

H -5.428094 -0.578768 -1.903363

H -4.137883 0.869744 1.912891

H -3.325726 0.781562 -1.657225

H -2.758381 1.225793 -0.054628

H -2.849817 -1.569658 -1.261356

H 1.122139 -2.790071 0.752625

H -1.046678 1.284002 1.491983

H -2.941782 -1.862665 1.215202

H -1.808251 -2.890785 -2.964127

H -1.634072 -1.203001 -3.427296

H -0.392906 -2.369525 -3.868075

H 1.323818 -4.571632 -1.289415

H 0.283723 -4.208391 -2.670667

H 1.962989 -3.657998 -2.636059

H 3.281613 -1.891361 -0.804856

H 3.127228 1.534618 2.200771

H 2.312458 1.681168 -0.757799

H 2.673359 3.082748 0.223406

H 4.953564 2.256913 0.651256

H 5.838744 2.562141 -1.664626

H 4.510304 3.685891 -1.357113

H 4.232715 2.231454 -2.328461

H 6.370636 -1.679789 -0.354408

H 6.604329 -1.560280 1.413986

H 5.016872 -2.048153 0.745412

H -0.322618 5.255495 -0.737294

H 0.803605 4.091894 -1.486285

H -0.925776 3.934742 -1.769677

YYY-148g-c4 , delta G = 2.0438 kcal/mol, population = 2.64 %

C -4.285957 2.185859 -0.419088

C -4.461656 2.561357 0.907795

C -4.439481 1.590803 1.904075

C -4.243194 0.254813 1.572750

C -4.051585 -0.134090 0.244249

C -4.077444 0.850333 -0.744927

C -3.843254 -1.580491 -0.112423

C -2.446616 -2.167366 0.163412

C -1.236784 -1.492660 -0.561858

C -0.481517 -0.701036 0.517232

C 1.045048 -0.784598 0.347785

C 1.513793 -2.223523 0.420982

C 0.786475 -3.005990 -0.668982

C -0.393631 -2.606995 -1.166003

C -0.701595 0.827978 0.656757

C 0.520499 1.304872 1.438712

C 1.600077 0.249454 1.315023

N -2.044233 -2.096620 1.555796

C -0.961203 -1.341895 1.838962

O -0.479390 -1.194411 2.952466

C -1.062525 -3.321349 -2.321296

C 1.504026 -4.234287 -1.161021

H -1.592717 -0.831928 -1.351422

H 1.254396 -0.468198 -0.676631

O 2.932494 -2.335033 0.297731

C 1.844111 1.646636 0.793071

C 2.086312 2.029786 -0.649319

C 3.584316 2.085599 -1.001318

C 4.204948 0.728841 -0.760104

C 3.795070 2.507116 -2.459108

O 3.903622 -0.275734 -1.379143

O 5.117802 0.746445 0.213068

C 5.755180 -0.509910 0.545939

H 2.140762 -0.093599 2.184986

H 0.308621 1.773052 2.388733

O -0.811794 1.364508 -0.686823

O -1.190025 3.432693 0.124266

C -1.074574 2.677331 -0.811662

C -1.187399 3.061695 -2.260153

H -4.304494 2.935399 -1.200283

H -4.610163 3.602374 1.163645

H -4.578702 1.873139 2.940105

H -4.241557 -0.494321 2.354177

H -3.940258 0.564885 -1.781221

H -4.565662 -2.194173 0.433651

H -4.049528 -1.732571 -1.174272

H -2.500003 -3.216633 -0.129792

H 1.292076 -2.650098 1.403616

H -1.618406 1.080257 1.182792

H -2.521913 -2.597466 2.290204

H -1.840577 -2.695455 -2.761360

H -0.349813 -3.562084 -3.109915

H -1.536668 -4.259175 -2.016440

H 1.941859 -4.773559 -0.318554

H 0.839831 -4.912087 -1.693262

H 2.334125 -3.977667 -1.824659

H 3.228690 -1.771273 -0.436817

H 2.455474 2.240930 1.466375

H 1.589098 1.353310 -1.341747

H 1.674685 3.025627 -0.831612

H 4.078881 2.797344 -0.338918

H 4.855898 2.581398 -2.703870

H 3.337682 3.482470 -2.631718

H 3.336525 1.784642 -3.135501

H 5.021910 -1.199198 0.959669

H 6.214562 -0.944171 -0.340524

H 6.510125 -0.259806 1.284593

H -0.231179 2.895745 -2.759225

H -1.929269 2.435376 -2.755942

H -1.466765 4.108375 -2.342462

### Table S22. Geometry data of conformers of structure 2h.

YYY-148h-c14 , delta G = 0.0000 kcal/mol, population = 35.86 %

C 6.981846 -0.620148 -0.475404

C 7.074098 -0.165015 -1.788098

C 5.996133 0.501719 -2.361384

C 4.833282 0.710595 -1.625824

C 4.729000 0.258466 -0.309427

C 5.817878 -0.410174 0.255436

C 3.449453 0.429543 0.464189

C 2.571444 -0.833101 0.420096

C 1.182064 -0.643270 1.088467

C 0.258316 -0.264993 -0.071014

C -1.191395 -0.677352 0.193242

C -1.309273 -2.176906 0.312917

C -0.327769 -2.658717 1.375580

C 0.741174 -1.940486 1.754268

C 0.132129 1.219902 -0.504109

C -1.205030 1.289957 -1.237054

C -2.010409 0.072386 -0.843039

N 2.195477 -1.224447 -0.931551

C 0.909261 -0.987723 -1.272297

O 0.390064 -1.254861 -2.345450

C 1.655507 -2.393311 2.872385

C -0.659207 -4.002574 1.966347

H 1.234777 0.149658 1.835258

H -1.446445 -0.282637 1.184045

O -2.652674 -2.582083 0.614936

C -2.459320 1.441578 -0.419138

C -3.709908 2.037870 -1.011619

C -4.971809 1.684009 -0.181046

C -5.028871 0.180985 0.013688

C -5.016234 2.415207 1.158941

O -4.677816 -0.410195 1.015388

O -5.488244 -0.437869 -1.081148

C -5.540621 -1.883060 -1.044910

H -2.574043 -0.474440 -1.585591

H -1.198355 1.655953 -2.253680

O 0.172607 2.031491 0.697552

O 0.371391 3.892556 -0.555185

C 0.270042 3.365424 0.528009

C 0.230574 4.080342 1.848624

H 7.818369 -1.135835 -0.020185

H 7.980658 -0.326075 -2.357696

H 6.060207 0.862399 -3.380478

H 3.997113 1.232439 -2.075995

H 5.753799 -0.765071 1.277997

H 3.667218 0.639750 1.514220

H 2.887984 1.279537 0.074234

H 3.125813 -1.648190 0.884149

H -1.076904 -2.656510 -0.642390

H 0.943931 1.543869 -1.151003

H 2.848579 -1.614799 -1.593795

H 2.259820 -1.560224 3.236058

H 1.092732 -2.784879 3.719416

H 2.347633 -3.177635 2.551595

H -1.481493 -3.930420 2.682546

H -0.997551 -4.682211 1.181016

H 0.193658 -4.457677 2.465408

H -3.133123 -1.850415 1.030216

H -2.305129 1.668808 0.629149

H -3.634689 3.127492 -1.058305

H -3.838518 1.679307 -2.034955

H -5.842863 1.964831 -0.776077

H -4.973970 3.492731 0.992912

H -4.181464 2.129580 1.797933

H -5.936613 2.188362 1.698520

H -5.920197 -2.179314 -2.017928

H -4.548236 -2.294059 -0.870326

H -6.216220 -2.211408 -0.256076

H -0.748937 3.937348 2.308674

H 0.976024 3.663500 2.526345

H 0.412114 5.140853 1.698342

YYY-148h-c4-gfn0 , delta G = 0.5547 kcal/mol, population = 14.05 %

C 3.607848 3.475726 1.247993

C 4.963938 3.215241 1.411802

C 5.591926 2.296443 0.574906

C 4.864986 1.638143 -0.410044

C 3.499082 1.882136 -0.576651

C 2.885107 2.813086 0.260613

C 2.703096 1.125925 -1.608649

C 2.615488 -0.376828 -1.286976

C 1.796229 -0.721604 -0.009780

C 0.395206 -1.042639 -0.529971

C -0.343733 -2.051403 0.353526

C 0.397473 -3.368219 0.395865

C 1.837205 -3.118476 0.808125

C 2.425445 -1.918282 0.688857

C -0.637976 0.101540 -0.682456

C -1.981103 -0.615886 -0.714469

C -1.791339 -1.998814 -0.128791

N 1.894660 -1.120021 -2.310276

C 0.667510 -1.563595 -1.959240

O -0.083633 -2.218872 -2.666473

C 3.825781 -1.660081 1.201544

C 2.549628 -4.320437 1.368894

H 1.788397 0.131174 0.668902

H -0.303313 -1.664465 1.376120

O -0.186766 -4.257744 1.366023

C -2.663839 -1.007106 0.577407

C -4.165774 -1.164093 0.582656

C -4.902881 0.086826 1.092614

C -4.432227 1.334859 0.373508

C -6.423646 -0.075185 0.993576

O -3.869464 2.265902 0.908527

O -4.708005 1.301874 -0.938614

C -4.264694 2.434811 -1.717747

H -2.232168 -2.857475 -0.618778

H -2.605844 -0.463691 -1.582584

O -0.521162 0.979622 0.465060

O -0.897186 2.801740 -0.810646

C -0.721613 2.299567 0.277024

C -0.708143 3.040163 1.579869

H 3.110897 4.195597 1.886431

H 5.529325 3.727551 2.179932

H 6.649609 2.093723 0.689087

H 5.365717 0.926638 -1.056430

H 1.834122 3.032178 0.128144

H 1.698884 1.544690 -1.687897

H 3.168679 1.232424 -2.592564

H 3.628091 -0.769870 -1.207642

H 0.371490 -3.857323 -0.585788

H -0.485644 0.697694 -1.577130

H 2.264942 -1.292173 -3.232913

H 3.975640 -2.089884 2.191917

H 4.593380 -2.082064 0.545695

H 4.017734 -0.588964 1.271236

H 2.299650 -5.209368 0.785591

H 3.630678 -4.199060 1.357312

H 2.236414 -4.527030 2.395174

H -1.047141 -4.531137 1.028674

H -2.249847 -0.583896 1.486416

H -4.517205 -1.409737 -0.422637

H -4.447210 -1.998978 1.231192

H -4.627433 0.246615 2.136532

H -6.942890 0.800595 1.386474

H -6.740601 -0.944363 1.572547

H -6.731202 -0.221321 -0.042312

H -4.541102 2.204534 -2.742304

H -4.768130 3.340444 -1.381887

H -3.187707 2.559893 -1.623208

H -1.710938 2.967225 2.005505

H 0.000178 2.597596 2.277955

H -0.475527 4.087827 1.405022

YYY-148h-c9 , delta G = 0.7009 kcal/mol, population = 10.97 %

C 5.988641 -0.067339 1.423198

C 5.482750 0.634717 2.514070

C 4.281871 1.325020 2.388449

C 3.589380 1.305507 1.181776

C 4.081824 0.600189 0.083639

C 5.293320 -0.082120 0.219803

C 3.299633 0.516371 -1.200629

C 2.642373 -0.865983 -1.370388

C 1.501168 -1.157176 -0.355442

C 0.225511 -0.763660 -1.099170

C -0.993889 -1.556750 -0.624977

C -0.805529 -3.034793 -0.879530

C 0.513419 -3.487996 -0.280180

C 1.505929 -2.633523 0.012077

C -0.272548 0.701377 -1.037718

C -1.742642 0.605502 -1.433838

C -2.187907 -0.825884 -1.230245

N 1.949460 -1.005958 -2.643231

C 0.599439 -1.016568 -2.577864

O -0.160891 -1.156476 -3.524135

C 2.773638 -3.084194 0.705420

C 0.616111 -4.970214 -0.037682

H 1.642990 -0.561972 0.545590

H -1.032876 -1.452935 0.463399

O -1.853677 -3.800780 -0.254952

C -2.778718 0.267866 -0.390022

C -4.214229 0.688020 -0.595724

C -4.560463 2.086403 -0.030683

C -4.233478 2.132008 1.449204

C -3.887268 3.230470 -0.782916

O -3.373034 2.811855 1.963113

O -5.011521 1.287257 2.151026

C -4.751878 1.199624 3.566033

H -2.795281 -1.314585 -1.981319

H -2.058226 1.168363 -2.299884

O -0.081887 1.172568 0.320424

O -0.252377 3.306219 -0.380444

C -0.128960 2.506954 0.518983

C -0.036420 2.848357 1.976047

H 6.926522 -0.601816 1.509930

H 6.023373 0.646988 3.452008

H 3.882997 1.879614 3.228763

H 2.654934 1.843524 1.090425

H 5.696184 -0.630340 -0.624282

H 2.534175 1.292922 -1.228371

H 3.959622 0.686829 -2.055085

H 3.422451 -1.623212 -1.302712

H -0.814394 -3.245373 -1.956095

H 0.269515 1.368873 -1.702482

H 2.427985 -1.097938 -3.526694

H 2.560187 -3.769511 1.525414

H 3.460448 -3.596353 0.024763

H 3.310193 -2.228529 1.117351

H 1.645375 -5.293634 0.101360

H 0.036807 -5.272804 0.837743

H 0.198953 -5.516797 -0.886708

H -2.672039 -3.613159 -0.728346

H -2.442463 0.291108 0.641508

H -4.450482 0.687257 -1.662728

H -4.867803 -0.049149 -0.124313

H -5.646325 2.193250 -0.106391

H -4.213893 4.195662 -0.394285

H -4.153520 3.180794 -1.840086

H -2.803647 3.187298 -0.698795

H -4.900398 2.168531 4.040624

H -5.464621 0.474868 3.947682

H -3.731871 0.861072 3.743646

H 0.514335 2.090908 2.530331

H 0.425301 3.826178 2.094185

H -1.056793 2.894734 2.363311

YYY-148h-c11 , delta G = 0.7285 kcal/mol, population = 10.47 %

C 4.860317 1.339481 1.863603

C 5.858333 0.409546 2.135045

C 6.110808 -0.612427 1.223751

C 5.365532 -0.704574 0.053917

C 4.355007 0.218486 -0.225950

C 4.116393 1.241194 0.692011

C 3.506532 0.076043 -1.461870

C 2.547791 -1.124796 -1.364198

C 1.452424 -0.982100 -0.269874

C 0.244240 -0.417244 -1.016916

C -1.078776 -0.824829 -0.369706

C -1.261917 -2.320644 -0.350068

C -0.028964 -2.974824 0.245522

C 1.152231 -2.344867 0.339239

C 0.079004 1.118853 -1.157397

C -1.401723 1.304643 -1.479704

C -2.123960 0.047243 -1.051564

N 1.743943 -1.293386 -2.566251

C 0.437041 -0.967887 -2.448392

O -0.404990 -1.062858 -3.328393

C 2.337918 -2.971615 1.041913

C -0.246212 -4.379889 0.740631

H 1.794636 -0.306301 0.512668

H -1.009237 -0.527788 0.682008

O -2.393629 -2.680586 0.468326

C -2.415956 1.346232 -0.364363

C -3.754206 2.013046 -0.570042

C -4.917130 1.197301 0.038688

C -4.572818 0.838374 1.468514

C -6.235977 1.968731 -0.023942

O -4.572946 1.614187 2.396925

O -4.221057 -0.458333 1.600284

C -3.807799 -0.874855 2.921550

H -2.862389 -0.404192 -1.700524

H -1.646806 1.801522 -2.407222

O 0.464408 1.707448 0.111102

O 0.411572 3.763114 -0.808356

C 0.557487 3.051943 0.158017

C 0.842523 3.526092 1.554825

H 4.659744 2.141890 2.562774

H 6.438220 0.482081 3.046426

H 6.890601 -1.336754 1.423874

H 5.572086 -1.502750 -0.650005

H 3.340859 1.967064 0.484831

H 2.932295 0.988052 -1.628652

H 4.140809 -0.066707 -2.340779

H 3.140850 -2.023399 -1.200978

H -1.434807 -2.700624 -1.365214

H 0.704841 1.552941 -1.933507

H 2.118892 -1.628647 -3.440860

H 3.089568 -2.216600 1.275284

H 2.043908 -3.447275 1.977400

H 2.828271 -3.735002 0.430462

H -0.854852 -4.938695 0.025657

H 0.688995 -4.917315 0.881119

H -0.794691 -4.386775 1.685575

H -3.070598 -1.999020 0.362277

H -2.017940 1.434942 0.641183

H -3.745034 3.011749 -0.126573

H -3.958504 2.136029 -1.636124

H -5.007681 0.265250 -0.519996

H -6.453427 2.247446 -1.056212

H -6.175827 2.878342 0.574350

H -7.066996 1.367568 0.348540

H -3.537219 -1.920711 2.822917

H -2.948878 -0.289517 3.246020

H -4.625735 -0.746888 3.628226

H 1.537561 2.858485 2.061915

H 1.235089 4.539177 1.526383

H -0.092427 3.524513 2.119998

YYY-148h-c1-gfn0 , delta G = 0.7323 kcal/mol, population = 10.40 %

C 5.576853 2.299455 0.592666

C 4.947026 3.223887 1.421963

C 3.592625 3.487591 1.249474

C 2.873344 2.822484 0.261192

C 3.489068 1.885902 -0.568463

C 4.853388 1.638807 -0.393302

C 2.696329 1.128221 -1.602040

C 2.614282 -0.375074 -1.282293

C 1.799659 -0.725185 -0.003243

C 0.398245 -1.049066 -0.520832

C -0.343612 -2.052740 0.366684

C 0.387684 -3.374134 0.416738

C 1.844302 -3.129322 0.788826

C 2.432600 -1.927278 0.681772

C -0.635080 0.093937 -0.682217

C -1.978345 -0.623465 -0.711416

C -1.789952 -2.000794 -0.111960

N 1.894544 -1.121363 -2.303788

C 0.673220 -1.577878 -1.946955

O -0.071285 -2.246824 -2.647225

C 3.838339 -1.681663 1.184328

C 2.572723 -4.339181 1.315919

H 1.793282 0.124577 0.679248

H -0.297898 -1.648674 1.385378

O -0.243341 -4.278748 1.338468

C -2.661215 -1.002320 0.584698

C -4.162719 -1.164402 0.590344

C -4.905676 0.085828 1.093531

C -4.434503 1.331915 0.371735

C -6.425470 -0.081311 0.989699

O -3.867434 2.262018 0.904263

O -4.713362 1.297925 -0.939446

C -4.268327 2.427945 -1.721711

H -2.228423 -2.871712 -0.579186

H -2.602356 -0.475931 -1.580907

O -0.517918 0.979537 0.460556

O -0.901974 2.793149 -0.824763

C -0.723541 2.297658 0.265411

C -0.711821 3.046124 1.563893

H 6.633382 2.094463 0.713273

H 5.509800 3.738279 2.190626

H 3.094395 4.212161 1.881581

H 1.823925 3.044373 0.121542

H 5.355873 0.923562 -1.034135

H 1.691082 1.544035 -1.682010

H 3.162342 1.237103 -2.585487

H 3.628627 -0.763844 -1.205572

H 0.320729 -3.889561 -0.544422

H -0.481071 0.684542 -1.580159

H 2.267086 -1.299572 -3.224309

H 4.597625 -2.087931 0.509010

H 4.032529 -0.612740 1.278746

H 3.999571 -2.138529 2.160843

H 3.650533 -4.251848 1.191160

H 2.370761 -4.508948 2.377490

H 2.238019 -5.235679 0.791298

H -0.243926 -3.861743 2.209554

H -2.249615 -0.566143 1.488940

H -4.512130 -1.416319 -0.414024

H -4.441265 -1.998008 1.241618

H -4.634415 0.250191 2.137906

H -6.728870 -0.231712 -0.046758

H -6.949100 0.793903 1.378099

H -6.741547 -0.949943 1.569959

H -4.766742 3.336115 -1.385188

H -3.190504 2.548604 -1.631408

H -4.549544 2.197420 -2.744886

H -0.004791 2.607918 2.266011

H -0.478534 4.092642 1.383090

H -1.715628 2.976493 1.987663

YYY-148h-c6 , delta G = 0.7656 kcal/mol, population = 9.84 %

C 4.958376 1.472797 1.698828

C 5.968949 0.561955 1.987945

C 6.194691 -0.509432 1.127828

C 5.410538 -0.669553 -0.009028

C 4.387522 0.234306 -0.305531

C 4.175720 1.306521 0.560940

C 3.493298 0.019729 -1.497817

C 2.515201 -1.149052 -1.276295

C 1.459638 -0.901682 -0.161937

C 0.233242 -0.376409 -0.910698

C -1.073352 -0.729183 -0.195241

C -1.260352 -2.224618 -0.112913

C -0.023610 -2.832641 0.539691

C 1.162941 -2.205726 0.565263

C 0.085684 1.146428 -1.169010

C -1.408006 1.343879 -1.417110

C -2.129748 0.139343 -0.857669

N 1.669372 -1.391047 -2.436232

C 0.373456 -1.029764 -2.303999

O -0.496133 -1.165382 -3.151257

C 2.356302 -2.775022 1.303228

C -0.243787 -4.185428 1.161328

H 1.836684 -0.171630 0.552793

H -0.952383 -0.392215 0.841139

O -2.456652 -2.570258 0.601275

C -2.319175 1.489379 -0.228121

C -3.644046 2.197365 -0.348825

C -4.595910 1.854680 0.827397

C -4.694755 0.346872 0.957542

C -4.155819 2.494558 2.142441

O -4.081223 -0.329248 1.758898

O -5.526351 -0.170156 0.044233

C -5.665112 -1.609827 0.024054

H -2.938635 -0.318064 -1.409633

H -1.703307 1.792058 -2.354839

O 0.561794 1.834349 0.017766

O 0.436679 3.809187 -1.058140

C 0.652781 3.177044 -0.050629

C 1.028583 3.765332 1.280114

H 4.779102 2.313117 2.358110

H 6.579396 0.687229 2.873269

H 6.983446 -1.219875 1.342080

H 5.594892 -1.505960 -0.673632

H 3.387747 2.014473 0.340446

H 2.930736 0.928971 -1.714335

H 4.093275 -0.203529 -2.383985

H 3.097503 -2.044951 -1.063464

H -1.395466 -2.651491 -1.111207

H 0.672427 1.497096 -2.014835

H 2.011007 -1.792048 -3.296787

H 3.121303 -2.010773 1.445935

H 2.075989 -3.151939 2.286905

H 2.825373 -3.601952 0.761432

H 0.689480 -4.719468 1.327947

H -0.769607 -4.104409 2.115952

H -0.877157 -4.796884 0.514639

H -2.713501 -1.844735 1.189794

H -1.824806 1.620379 0.727338

H -3.502948 3.281380 -0.360730

H -4.121252 1.924550 -1.292232

H -5.589016 2.221166 0.560798

H -4.094030 3.577251 2.022750

H -3.181891 2.124873 2.461624

H -4.867382 2.279145 2.940555

H -6.357086 -1.818503 -0.786058

H -4.700470 -2.081057 -0.153806

H -6.072768 -1.957602 0.972377

H 1.693631 3.104324 1.832925

H 1.487967 4.739829 1.134629

H 0.117470 3.892433 1.869659

YYY-148h-c4 , delta G = 1.2688 kcal/mol, population = 4.20 %

C 4.281887 1.338963 2.375957

C 5.473285 0.635605 2.518346

C 5.972585 -0.094081 1.442648

C 5.280198 -0.123201 0.237807

C 4.078071 0.572112 0.084981

C 3.592205 1.304839 1.167950

C 3.298556 0.475055 -1.200049

C 2.635365 -0.906279 -1.353090

C 1.492238 -1.180770 -0.335265

C 0.218771 -0.790579 -1.084688

C -1.008905 -1.564034 -0.597663

C -0.841708 -3.047760 -0.831533

C 0.492489 -3.504944 -0.256183

C 1.488528 -2.654771 0.039781

C -0.266446 0.679760 -1.053254

C -1.737987 0.589468 -1.445126

C -2.197035 -0.832257 -1.207767

N 1.942593 -1.061194 -2.624057

C 0.592106 -1.074952 -2.558157

O -0.168035 -1.237462 -3.500135

C 2.752335 -3.115674 0.732637

C 0.600882 -4.989551 -0.019724

H 1.637047 -0.577909 0.560243

H -1.036224 -1.435198 0.491290

O -1.937352 -3.784888 -0.263352

C -2.771523 0.284976 -0.388098

C -4.205003 0.710478 -0.597396

C -4.541906 2.122476 -0.060903

C -4.220652 2.193127 1.418844

C -3.855845 3.246001 -0.832217

O -3.344816 2.861062 1.922527

O -5.023774 1.384660 2.135671

C -4.775307 1.324846 3.553805

H -2.818029 -1.341288 -1.931921

H -2.050644 1.139565 -2.320423

O -0.068409 1.177171 0.295342

O -0.220526 3.297436 -0.449033

C -0.103703 2.515736 0.466435

C -0.007761 2.886798 1.916131

H 3.888474 1.915548 3.203978

H 6.011975 0.659393 3.457181

H 6.903357 -0.638675 1.542158

H 5.678427 -0.692560 -0.594369

H 2.665538 1.853686 1.063346

H 2.537444 1.255146 -1.240064

H 3.961341 0.630572 -2.055197

H 3.412593 -1.665426 -1.274874

H -0.894618 -3.279367 -1.897975

H 0.281314 1.328128 -1.732045

H 2.421129 -1.175176 -3.504881

H 3.294092 -2.265922 1.150111

H 2.533141 -3.805869 1.547307

H 3.436449 -3.628723 0.049831

H 0.125960 -5.291109 0.918273

H 0.086973 -5.533506 -0.814353

H 1.636579 -5.323082 0.007704

H -1.968815 -3.586980 0.681365

H -2.429420 0.332686 0.640883

H -4.444176 0.688908 -1.663438

H -4.862165 -0.013138 -0.110246

H -5.626438 2.236464 -0.143474

H -4.118503 3.178030 -1.889247

H -2.773006 3.194895 -0.743383

H -4.175653 4.221398 -0.463725

H -5.508801 0.628040 3.948101

H -3.765305 0.965243 3.747142

H -4.901964 2.308314 4.004187

H -1.027739 2.953407 2.301441

H 0.534164 2.135181 2.486838

H 0.465421 3.861405 2.013844

YYY-148h-c3 , delta G = 1.5518 kcal/mol, population = 2.60 %

C 4.130350 1.983191 -1.975540

C 4.228516 2.810024 -0.861178

C 4.263587 2.246804 0.409443

C 4.187641 0.867211 0.563861

C 4.082324 0.025813 -0.545064

C 4.063973 0.603293 -1.817251

C 3.997769 -1.465767 -0.373355

C 2.596016 -2.089360 -0.508922

C 1.495230 -1.559530 0.466760

C 0.561263 -0.684547 -0.383526

C -0.915189 -0.872912 -0.001680

C -1.346101 -2.311223 -0.159533

C -0.445135 -3.165920 0.727631

C 0.790052 -2.768454 1.069681

C 0.696849 0.861863 -0.350154

C -0.644290 1.360261 -0.889085

C -1.646270 0.242101 -0.729153

N 1.984090 -1.872232 -1.807004

C 0.855824 -1.131525 -1.831665

O 0.211909 -0.861511 -2.834915

C 1.661879 -3.555584 2.024162

C -1.054464 -4.461370 1.189348

H 1.953371 -0.976190 1.264297

H -0.975224 -0.669819 1.073097

O -2.731476 -2.493667 0.157966

C -1.799370 1.510918 0.061913

C -2.931202 2.457257 -0.244028

C -4.196634 2.147091 0.598768

C -4.542197 0.679675 0.441058

C -4.026125 2.518945 2.070202

O -4.257947 -0.196567 1.233660

O -5.177785 0.446320 -0.713861

C -5.510912 -0.928991 -1.013323

H -2.340328 0.008773 -1.523771

H -0.624173 1.973542 -1.778039

O 0.927055 1.226668 1.035201

O 1.075846 3.405486 0.477265

C 1.104795 2.531458 1.310797

C 1.336002 2.737518 2.781601

H 4.106405 2.412286 -2.969373

H 4.273122 3.884570 -0.981443

H 4.343689 2.883723 1.281421

H 4.212517 0.436060 1.557740

H 3.998717 -0.031508 -2.691707

H 4.644518 -1.955416 -1.107085

H 4.377856 -1.746810 0.611375

H 2.724712 -3.161975 -0.360260

H -1.252998 -2.631385 -1.202526

H 1.531648 1.237235 -0.934746

H 2.362502 -2.253009 -2.661510

H 2.416257 -2.905746 2.472006

H 1.082748 -3.994128 2.835434

H 2.196144 -4.371518 1.527729

H -1.553204 -4.956852 0.352818

H -0.313804 -5.146575 1.595389

H -1.824802 -4.292774 1.945498

H -3.034951 -1.790255 0.752059

H -1.557066 1.427155 1.114959

H -2.636241 3.489340 -0.037508

H -3.181133 2.400001 -1.305440

H -5.021915 2.720795 0.172848

H -4.942680 2.331512 2.631187

H -3.783371 3.579053 2.156717

H -3.229338 1.942024 2.538379

H -6.002646 -0.899469 -1.980782

H -4.609482 -1.537481 -1.050946

H -6.185911 -1.322403 -0.254349

H 1.521624 3.789280 2.980958

H 0.461577 2.403678 3.342626

H 2.185965 2.138738 3.110956

YYY-148h-c3-gfn0 , delta G = 1.8392 kcal/mol, population = 1.60 %

C 4.228755 3.316249 -2.299506

C 5.566175 3.355917 -1.918580

C 5.954567 2.781883 -0.711011

C 5.010181 2.169805 0.105635

C 3.664792 2.122250 -0.267206

C 3.286661 2.703571 -1.478659

C 2.655841 1.400414 0.585189

C 2.675875 -0.117720 0.336036

C 1.546001 -0.888546 1.075582

C 0.421490 -0.979807 0.045300

C -0.484423 -2.192430 0.271716

C 0.292944 -3.479901 0.121631

C 1.544739 -3.418779 0.987294

C 2.042816 -2.270561 1.473234

C -0.582646 0.193372 -0.095563

C -1.798077 -0.433657 -0.762397

C -1.710087 -1.936075 -0.597144

N 2.410597 -0.475088 -1.050778

C 1.195806 -1.015418 -1.292662

O 0.787374 -1.405876 -2.375804

C 3.214243 -2.254546 2.430347

C 2.172614 -4.757643 1.279326

H 1.237112 -0.336914 1.964657

H -0.789455 -2.150999 1.324975

O -0.519743 -4.616984 0.456782

C -2.816926 -1.179644 0.070376

C -4.236104 -1.275366 -0.436550

C -5.164032 -0.203557 0.162382

C -4.568502 1.181766 0.014336

C -6.571874 -0.276401 -0.438744

O -4.240849 1.892269 0.940661

O -4.438865 1.545992 -1.269841

C -3.844217 2.839017 -1.516393

H -1.925654 -2.597170 -1.425023

H -2.134897 0.003097 -1.691340

O -0.885253 0.713384 1.223303

O -0.928958 2.829564 0.439632

C -1.074116 2.044281 1.349283

C -1.500115 2.402567 2.740404

H 3.917153 3.764104 -3.234994

H 6.300085 3.833969 -2.555108

H 6.992734 2.814145 -0.404187

H 5.318607 1.727282 1.046234

H 2.245743 2.678056 -1.778119

H 2.868592 1.562915 1.644688

H 1.658730 1.794719 0.389659

H 3.658902 -0.494223 0.616792

H 0.563474 -3.648610 -0.923368

H -0.190664 1.011716 -0.691735

H 3.068593 -0.310412 -1.797260

H 3.118648 -3.025715 3.194606

H 4.168317 -2.420110 1.921058

H 3.283122 -1.291316 2.938837

H 3.225161 -4.667048 1.541300

H 1.666276 -5.276492 2.098334

H 2.092103 -5.407519 0.406176

H -0.826508 -4.504746 1.365593

H -2.729269 -1.068576 1.146449

H -4.247268 -1.198795 -1.526584

H -4.653718 -2.253880 -0.182124

H -5.226471 -0.365688 1.239953

H -7.233342 0.468772 0.006498

H -7.000884 -1.262799 -0.254246

H -6.543140 -0.110826 -1.516220

H -2.844120 2.882040 -1.089205

H -3.809332 2.937759 -2.597225

H -4.460497 3.624877 -1.081611

H -2.583664 2.277615 2.790323

H -1.039843 1.746102 3.476742

H -1.255128 3.442283 2.943112
